# Placental transcriptome profiling in congenital Chagas disease: gene networks associated with transmission

**DOI:** 10.3389/fcimb.2026.1749307

**Published:** 2026-03-18

**Authors:** Sofia Apodaca, Emiliano E. Campos, Kevin D. Calupiña, Carolina Davies, Raúl H. Lucero, Silvia A. Longhi, Laura Kamenetzky, Maria Paola Zago, Alejandro G. Schijman

**Affiliations:** 1 Laboratorio de Biología Molecular de la Enfermedad de Chagas (LabMECh), Instituto de Investigaciones en Ingeniería Genética y Biología Molecular (INGEBI), Consejo Nacional de Investigaciones Científicas y Técnicas (CONICET), Buenos Aires, Argentina; 2 Unidad de Conocimiento Traslacional del Hospital Público Materno Infantil (UCT-HPMI), Salta, Argentina; 3 Instituto de Patología Experimental, Consejo Nacional de Investigaciones Científicas y Técnicas (CONICET) - Universidad Nacional de Salta, Salta, Argentina; 4 Laboratorio de Genómica y Bioinformática de Patógenos, iB3, Instituto de Biociencias, Biotecnología y Biología traslacional, Departamento de Fisiologia y Biologia Molecular y Celular, Facultad de Ciencias Exactas y Naturales, Universidad de Buenos Aires, Buenos Aires, Argentina; 5 Instituto de Biología de Organismos Marinos (IBIOMAR), Consejo Nacional de Investigaciones Científicas y Técnicas (CONICET), Puerto Madryn, Chubut, Argentina; 6 Instituto de Medicina Regional, Universidad Nacional del Nordeste (UNNE), Resistencia, Chaco, Argentina

**Keywords:** co-expression network analysis, congenital Chagas disease, differential gene expression, gene set enrichment analysis, host-parasite interaction, placenta, RNA-Seq, transcriptomics

## Abstract

Chagas disease, caused by *Trypanosoma cruzi*, affects over seven million people worldwide. Vertical transmission during pregnancy contributes to the urban spread of the disease, including in non-endemic regions. Although the placenta constitutes a critical barrier against fetal infection, the molecular mechanisms underlying congenital transmission remain poorly understood. To identify placental factors associated with transmission, we performed a transcriptomic analysis of placental tissues from deliveries of congenitally infected (M+B+), exposed but uninfected (M+B−), and unexposed/uninfected (M−B−) newborns. Differential gene expression analysis comparing M+B+ and M−B− placentas revealed overexpression of CEMIP (cell migration–inducing hyaluronidase 1), involved in extracellular matrix (ECM) remodeling and intracellular transport, together with ENSG00000304767, a novel long non-coding RNA located intronically within *CEMIP*. In contrast, PRRX1 (paired related homeobox 1), CADM3 (cell adhesion molecule 3), and CDH11 (cadherin 11), genes associated with transcriptional regulation and cell–cell adhesion, were underexpressed. In the M+B− versus M−B− comparison, MIR4300HG, a long non-coding RNA hosting *MIR4300*, was overexpressed, whereas CGB5 (chorionic gonadotropin subunit beta 5), essential for pregnancy maintenance, was underexpressed. Direct comparison between M+B+ and M+B− placentas showed overexpression of the *CEMIP*-associated lncRNA and CGB5, accompanied by downregulation of CADM3, NEGR1, PDPN, and CDH11, implicating altered adhesion and structural pathways in transmission. Overall, these findings indicate that placental cell adhesion and ECM integrity are disrupted in transmitting placentas. Gene set enrichment analysis using the Gene Ontology library revealed alterations of immune-related pathways in both infected mother groups, while highlighting ECM-related processes—particularly collagen organization and metabolism—as key contributors to transmission events. Cell type enrichment analysis showed overrepresentation of extravillous trophoblasts in M+B+ placentas, with the opposite pattern observed in M+B− cases. Conversely, syncytiotrophoblasts and villous cytotrophoblasts were enriched in non-transmitting placentas relative to controls. Immune-associated placental cell types were consistently reduced in both infected groups. Co-expression network analysis further confirmed compromised placental signaling and structural integrity in transmitting cases, identifying ENPP1 and SLC16A10 as central hub genes. Together, these RNA-seq data define key placental transcriptional alterations associated with congenital *T. cruzi* transmission which, upon future experimental validation may provide insights into molecular mechanisms governing fetal protection or susceptibility.

## Introduction

Chagas disease (CD), caused by *Trypanosoma cruzi*, remains a major public health concern in Latin America and is increasingly recognized in non-endemic regions due to migration (World Health Organization, 2024). While vectorial and transfusional routes have declined in prevalence, mother-to-child transmission causing congenital Chagas disease (cCD) has emerged as the predominant mode of new infections in urban and non-endemic settings. An estimated 5% of children born to infected mothers acquire the infection during pregnancy, yet the biological factors determining transmission risk remain poorly understood.

The placenta is the critical interface between maternal and fetal environments, acting as both a physical and immunological barrier against vertical transmission of pathogens (Carlier et al., 2012; Liempi et al., 2016). Despite its protective role, congenital transmission of *T. cruzi* still occurs, indicating that specific alterations in placental structure or function may facilitate the passage of the parasite to the fetus. Among the placental mechanisms to protect the fetus from the infection are fetal production of proinflammatory cytokines (Cuna et al., 2009), fetal natural killer response (Hermann et al., 2006) and epithelial trophoblast turnover (Liempi et al., 2016; Kemmerling et al., 2019; Carlier et al., 2020; Christian et al., 2025). However, little is known about the molecular and cellular changes in the placenta associated with *T. cruzi* transmission.

Given the complexity of host–pathogen interactions during congenital infections, transcriptomic approaches that go beyond single-gene analyses are valuable for understanding broader regulatory dynamics. Gene co-expression network analysis provides a framework to identify groups of coordinately expressed genes (modules) and central hub genes that may play key roles in disease processes (Chen et al., 2008).

Building on a previous study that examined gene expression in placentas from *T. cruzi*-infected mothers who did not transmit cCD compared with non-infected controls (Juiz et al., 2018), the present study sought to further define placental gene expression profiles and gene co-expression networks associated with vertical transmission.

For the first time, we performed an integrative transcriptomic analysis of placental tissues from three clinical groups: congenitally infected newborns born to seropositive women (M+B+), exposed but uninfected newborns (M+B−), and unexposed newborns born to seronegative women (M−B−). This approach aimed to provide new insights into host–parasite interactions at the maternal–fetal interface and identify candidate molecular pathways and markers associated with susceptibility to cCD.

## Materials and methods

### Ethical statement

The study was conducted upon approval of the bioethics committees of the participating institutions, following the principles of the Helsinki declaration, in accordance with resolution 1480/2011 of the Ministerio de Salud from Argentina. Patients were recruited at the Hospital Perrando, Resistencia, Province of Chaco and at the Hospital Público Materno Infantil, Salta, Province of Salta. In all cases, the purpose of the study was explained to the mothers, and informed consent was obtained before sample collection.

### Subjects enrollment and sample collection

We aimed to compare placental RNA expression profiles across three clinical groups. The first group consisted of placentas collected at term from *Trypanosoma cruzi*–seropositive women who delivered congenitally infected newborns (M+B+). The second group included placentas collected at term from seropositive women who delivered non-infected newborns (M+B−). The third group comprised placentas collected at term from seronegative women who delivered non-infected newborns (M−B−). These three groups enabled transcriptional comparisons between M+B+ and M−B−, M+B− and M−B−, as well as between M+B+ and M+B− placentas.

Pregnant women were diagnosed with CD using conventional serological methods performed at their respective healthcare centers as part of routine screening. Standard clinical guidelines also included screening for toxoplasmosis (Durlach et al., 2021), preeclampsia (Poon et al., 2021), premature rupture of membranes (Ubom et al., 2023) and meconium-stained amniotic fluid.

Infants born to seropositive mothers were tested for cCD, following the current diagnostic algorithm. This includes a micromethod assay and/or qPCR to detect parasitemia within the first days of life or between 4 and 8 weeks of age, followed by confirmatory serological testing—using two distinct serological methods—after the infant reaches nine months of age (Schijman et al., 2024).

Fresh normal placentas were obtained immediately after labor from vaginal or cesarean deliveries, kept at 4 °C and processed within the next 3 h. Subsequently, each placenta was dissected: a portion of tissue located at 4 cm from the umbilical cord was extracted, and the middle region was immersed in RNAlater Solution (Applied Biosystems, Foster City, CA), overnight at 4 °C to facilitate tissue penetration. Finally, the samples were stored at -80 °C until RNA extraction.

### RNA extraction

Placental samples preserved in RNAlater Solution were thawed and always kept cold. Between 50 mg and 100 mg of placental tissue were cut and weighed on a Petri dish. The RNeasy Lipid Tissue Mini Kit (Qiagen, Hilden, Germany) was used according to the manufacturer’s instructions. The tissue was disaggregated after adding 1 mL of Qiazol (Qiagen), using the Fisherbrand™ Pellet Pestle™ Cordless Motor with clean Pellet Pestles (Thermo Fisher Scientific, Waltham, Massachusetts, USA). DNA digestion was also performed using an RNase-Free DNase Set (Qiagen) on the same column according to the manufacturer’s instructions. Elution was performed with 50 μL of DEPC-treated water. Aliquots were separated from the RNA obtained to have a first approximation of the concentration; purity of the RNA was assessed using the DS-11 Spectrophotometer (DeNovix, Wilmington, USA) and integrity was verified after electrophoresis in 1% Agarose Gel, with 0.5X TBE buffer. The remaining RNA solution was precipitated by adding 0.1 volumes of Sodium Acetate, pH 5.5, mixing gently and then adding 2 volumes of 100% ethanol and mixing again. The precipitated RNA was stored at -80 °C until shipping.

### Transcriptomic studies

An RNA-seq study was performed using samples from three clinical groups control or uninfected placentas (M−B−, n=7), infected but non-transmitting placentas (negative for cCD or M+B−, n=4), and infected, transmitting placentas (positive for cCD or M+B+, n=5). The preparation of cDNA libraries and sequencing was performed by Macrogen (Seoul, Republic of Korea): samples were used individually to construct the libraries using the TruSeq Stranded Total RNA with Ribo-Zero H/M/R Gold kit (Illumina, San Diego, CA). Sequencing was performed using Illumina NovaSeq 6000 platform with a 101 bp paired-end reads strategy (**Supplementary Table 1**). The RNA-seq data have been submitted to the National Center for Biotechnology Information (NCBI) Sequence Read Archive (SRA) under the BioProject accession number PRJNA1297177.

### RNA sequencing analysis

Raw read quality was initially assessed using FastQC (version 0.11.5; Babraham Bioinformatics, Cambridge, United Kingdom). Subsequently, adapter sequences were removed using Trimmomatic, version 0.36, in paired-end mode (Bolger et al., 2014), and the quality of the trimmed reads was re-evaluated with FastQC. The cleaned reads were then aligned to the human reference genome GRCh38.p14, obtained from Gencode (https://www.gencodegenes.org/human/), using STAR, version 2.5.2b (Dobin et al., 2013) with default parameters, generating coordinate-sorted BAM files. The integrity of these BAM files was verified using Samtools, version 1.3.1, via its flagstat function (Li et al., 2009).

For quantification, featureCounts, version 2.0.1, was employed in paired-end mode (Liao et al., 2014) to generate a count matrix, which was subsequently transformed into a DESeq2 object (dds). A filtering criterion [rowSums(counts(dds) ≥ 3) ≥ 6] was applied to retain only those genes with at least 3 counts in a minimum of 6 samples, thereby minimizing noise from lowly expressed genes. Finally, differential expression analysis was performed using the DESeq2 package, version 1.44.0 (Love et al., 2014), facilitating the identification of differentially expressed genes. DEGs were identified using an adjusted p-value threshold of 0.05 and an absolute log_2_ fold change (|log_2_FC|) greater than 1.

Principal component analysis (PCA) was performed on variance-stabilized counts using the 1,000 most variable genes with PCAtools package, version 3.22 (Blighe and Lun, 2019).

Functional enrichment analysis was conducted using the ClusterProfiler package, version 4.14.6 (Wu et al., 2021), applying thresholds of pvalueCutoff < 0.01 and qvalueCutoff < 0.05. P-value correction was performed using the Benjamini-Hochberg method, and the results were visualized with ggplot2. Considering that the hierarchical structure of Gene Ontology (GO) terms can lead to redundancy in the list of enriched terms, the simplify function from the GOSemSim package was employed with default thresholds to evaluate semantic similarities among GO terms, effectively eliminating closely related terms, while retaining only the most representative ones. Gene set enrichment analysis was performed using the GSEA software, version 4.3.3 (Subramanian et al., 2005), with the MSigDB gene sets (Liberzon et al., 2015), specifically the C5 collection (ontology gene sets, 16,107 gene sets) and the C8 collection (cell type signature gene sets, 840 gene sets), employing 1000 permutations and default settings for all other parameters. For GSEA analysis using the whole expression matrix, an FDR < 25% was considered significant.

### Co-expression gene analysis

Gene co-expression networks were constructed using the WGCNA package version 1.72-1 (Langfelder and Horvath, 2008), on variance-stabilized DESeq2 version 1.44.0 (Love et al., 2014), data. Genes with low expression were removed following the initial filtering criteria described above, and the expression matrix was further filtered to include only genes with variance in the upper quartile to improve network robustness.

Similarities in gene expression between pairs were calculated using Pearson’s correlation coefficient. These were transformed into adjacency matrices using a soft-threshold of power = 14, selected to approximate a scale-free topology according to standard WGCNA criteria (Langfelder and Horvath, 2008). A signed network was constructed, including only positive correlations (genes with similar expression patterns), while negative correlations were downweighted to better reflect biologically relevant co-expression. The topological overlap matrix (TOM) and its dissimilarity (dissTOM) were calculated to assess the interconnectedness of the network.

Modules of co-expressed genes were identified by hierarchical clustering of the dissTOM, followed by dynamic tree cutting (minimum module size = 30 genes). Modules with high eigengene similarity were merged based on a predefined soft-threshold.

Module eigengenes were correlated with clinical traits (M−B−, M+B−, M+B+) to identify phenotype-associated modules. Modules were prioritized for downstream analysis based on the strength, direction, and statistical significance of their eigengene-trait correlations, together with concordance with differential expression patterns and biological relevance. Hub genes were defined as those with high node degree (the number of connections a gene has within the module), and high betweenness centrality (the extent to which a gene acts as a bridge between other genes) within the co-expression network. Functional annotation of selected modules was performed using ClusterProfiler package, version 4.14.6 (Wu et al., 2021), and networks were visualized with Cytoscape version 3.10.0 (Shannon et al., 2003).

## Results

### Enrollment of mother-infant pairs. Placental characterization

**Figure 1:**
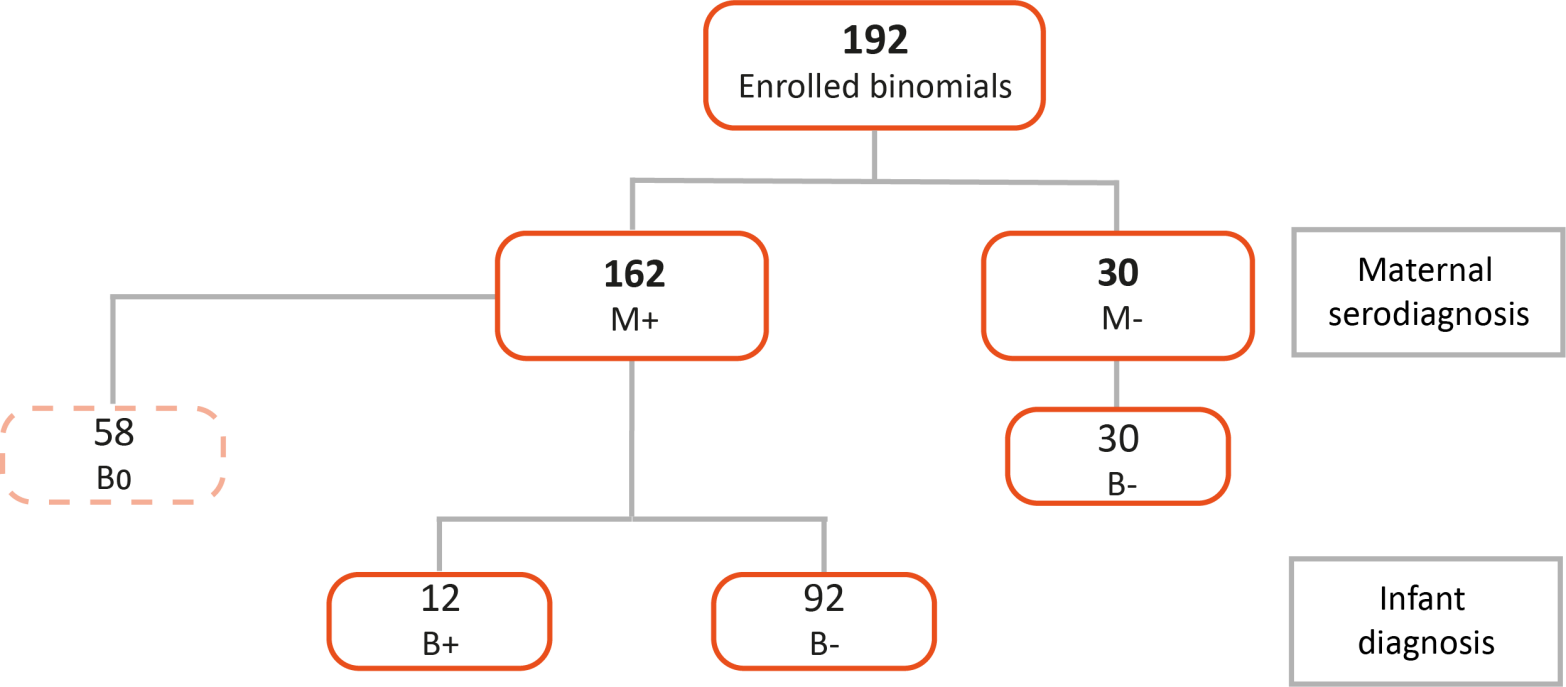
Flowchart of enrolled mother/newborn pairs to characterize placental groups. Mothers that were seropositive for Chagas disease are depicted as M+, while seronegative mothers are designated as M−. B+ corresponds to babies diagnosed positive for cCD, and B- to babies diagnosed as non-infected. Thus, transmitting placentas come from M+B+ pairs, non-transmitting ones come from M+B-, and negative controls from M-B-. B0 refers to babies that did not complete the diagnostic algorithm.

In total, 192 pregnant women were enrolled; 162 of them were diagnosed as positive for CD (M+) and 30 as negative (M−). When mothers tested positive, their babies were diagnosed, resulting in 12 positives (B+) and 92 negatives (B−). 58 newborns that did not complete the diagnostic algorithm (B0) were excluded from the study (**Figure 1**).

The placental samples were selected and processed based on the characteristics of the clinical cases, meaning that the transmitting placentas corresponded to M+B+, the non-transmitting to M+B- and the controls to M−B− dyads. The experimental placental groups were set keeping the tightest equivalence in terms of type of delivery and newborn’s sex, including RIN values of the purified RNAs and the clinical characteristics described in **Table 1**.

**Table 1:** Clinical characteristics of the placental RNA samples used in the transcriptomic analysis.

ID	Clinical group	Mother's age (years)	IgG Toxo	Gestation (weeks)	Pregnancy complications	Delivery	Newborn's sex	RIN RNA
H014	M−B−	20	Positive	40	PE	Cesarean	Male	5.4
H020	M−B−	26	Negative	39	No	Vaginal	Male	4.2
M076	M−B−	25	Negative	40	No	Vaginal	Male	4.4
M038	M−B−	39	Negative	39	No	Vaginal	Female	4.9
21NH	M−B−	21	Positive	39	No	Vaginal	Male	6.1
H013	M−B−	25	Negative	38	No	Cesarean	Female	5.4
H011	M−B−	22	Positive	39	No	Cesarean	Female	5.1
M059	M+B−	30	Negative	40	No	Vaginal	Male	4.7
M108	M+B−	26	Negative	36	PRM	Vaginal	Male	4
M084	M+B−	35	Positive	37	PE	Cesarean	Female	3.2
48QP	M+B−	37	Negative	38	No	Cesarean	Female	4.6
M083	M+B+	43	Positive	37	PE	Cesarean	Male	5.3
M030	M+B+	25	Positive	38	PE	Cesarean	Male	4.7
M072	M+B+	25	Negative	40	Meco	Vaginal	Male	4.5
15II	M+B+	31	Positive	40	No	Vaginal	Female	4.9
M070	M+B+	41	Positive	40	No	Cesarean	Female	4.3
M098	M+B+	28	Negative	34	PRM	Cesarean	Female	4.1

ID, Patient identifier code; IgG Toxo, the mother’s diagnosis of *Toxoplasma gondii* by indirect immunofluorescence assay. Results are reported as Positive or Negative according to a cut-off titer of 1:16. Titers ≥ 1:16 were considered positive. PE, Preeclampsia; PRM, Premature Rupture of Membranes; Meco, Meconium.

Global transcriptional profiles of placental samples were explored by hierarchical clustering based on sample-to-sample distances (**Supplementary Figure S1**). The distance heatmap and associated dendrogram identified distinct transcriptional patterns across samples. Notably, sample M098 displayed a divergent expression profile, characterized by a predominance of overrepresented genes in the heatmap. Importantly, M098 was the only placental sample in the cohort in which intracellular amastigote nests were detected by microscopy, a rare finding in placental tissue (data not shown). Consistent with this observation, pairwise scatter plot comparisons of variance-stabilized (VST) normalized expression values showed reduced concordance between M098 and other samples within the same group (R² < 0.90), whereas comparisons among the remaining M+B+ samples yielded higher correlations (R² > 0.94) (**Supplementary Figure S2**). Collectively, these analyses indicated that M098 represented a biologically and transcriptomically distinct outlier. Accordingly, this sample was excluded from subsequent differential expression analyses.

Principal Component Analysis plots are shown in **Supplementary Figure S3**. PC1 and PC2 accounted for 26.09% and 13.89% of variance in the RNA-seq dataset, and showed overlapping among groups.

### Differentially expressed genes

**Figure 2:**
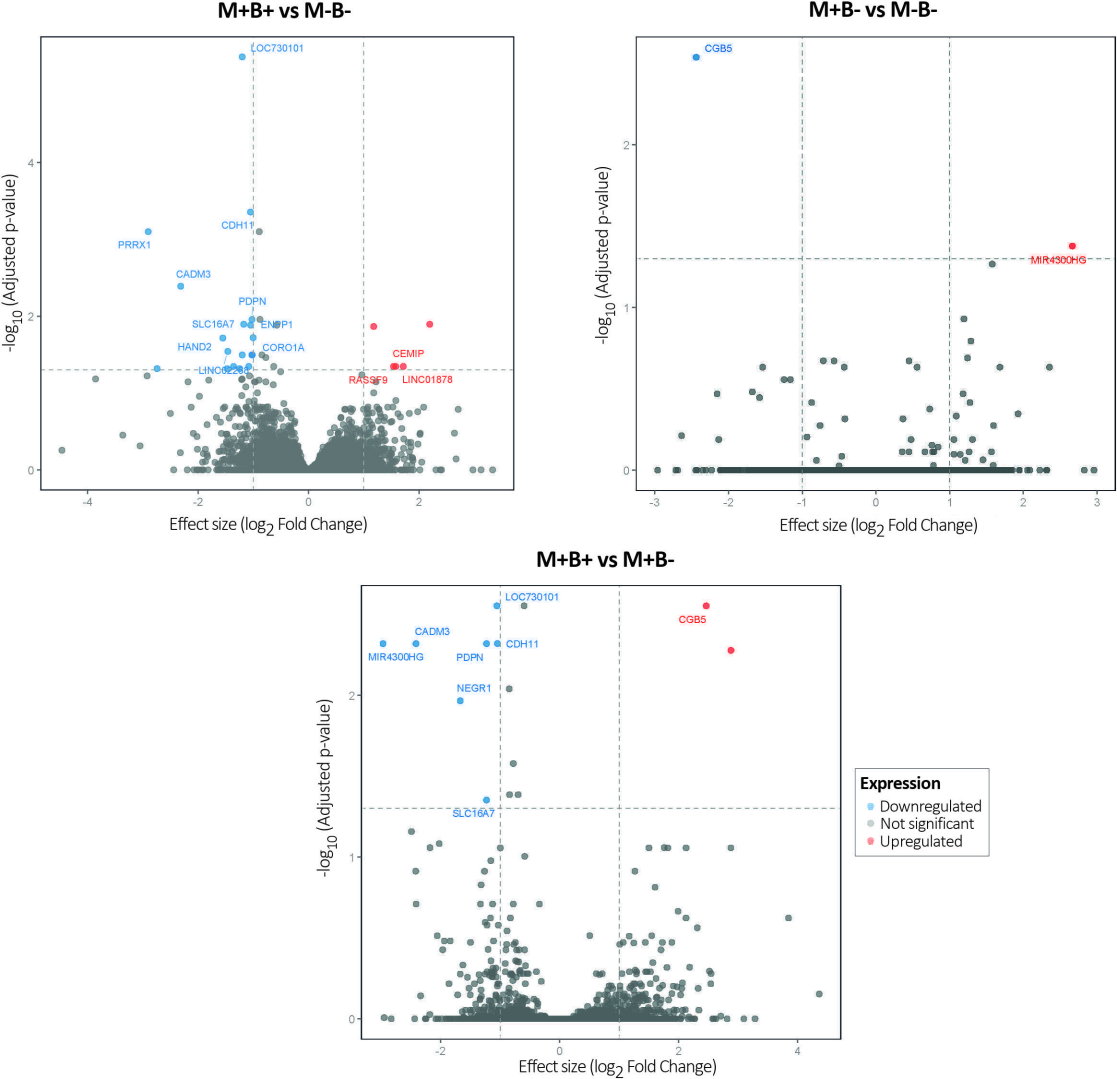
Differentially expressed genes. Volcano plots illustrating DEGs for the comparisons: M+B+ vs. M−B−, M+B− vs. M−B−, and M+B+ vs. M+B−. The y-axis represents the negative logarithm of the adjusted p-value, with a horizontal dashed line indicating the significance threshold (*p*_adj_ = 0.05). The x-axis displays the log_2_ Fold Change, with vertical dashed lines at 1 and -1. Red and blue dots represent significantly upregulated and downregulated genes, respectively (*p*_adj_ < 0.05 and |FC|>1), while grey dots represent genes that did not surpass this cutoff.

**Figure 2** and **Tables 2**–**4** show DEG analysis between M+B+ and M−B−, M+B− and M−B− and M+B+ and M+B−. Only those DEGs with a *p*_adj_ value ≤0.05 and |FC| > 1 are listed.

**Table 2:** Differentially expressed genes in the M+B+ vs. M-B- comparison.

Upregulated				Downregulated			
GID	Gene	log_2_ FC	*p*_adj_	GID	Gene	log_2_ FC	*p*_adj_
ENSG00000304767.1	NTSI to *CEMIP*	2.3287	0.0108	ENSG00000116132.12	*PRRX1*	-2.9373	0.0016
ENSG00000103888.17	*CEMIP*	1.5462	0.0413	ENSG00000162706.13	*CADM3*	-2.4660	0.0004
ENSG00000310416.1	*LOC105379271*	1.2143	0.0099	ENSG00000198848.13	*CES1*	-2.3106	0.0482
ENSG00000232406.9	*EPB41L1-AS1* – lncRNA	1.1499	0.0417	ENSG00000162711.19	*NLRP3*	-1.9107	0.0482
				ENSG00000164107.9	*HAND2*	-1.6862	0.0016
				ENSG00000187672.15	*ERC2*	-1.6512	0.0482
				ENSG00000248174.9	*LINC02268*	-1.4779	0.0465
				ENSG00000070193.5	*FGF10*	-1.4409	0.0288
				ENSG00000291006.1	*LOC730101*	-1.2623	0.0000
				ENSG00000118596.12	*SLC16A7*	-1.1433	0.0309
				ENSG00000141338.15	*ABCA8*	-1.1199	0.0482
				ENSG00000140937.14	*CDH11*	-1.1034	0.0002
				ENSG00000197594.14	*ENPP1*	-1.0990	0.0108
				ENSG00000149090.13	*PAMR1*	-1.0722	0.0288
				ENSG00000117525.14	*F3*	-1.0706	0.0288
				ENSG00000102879.17	*CORO1A*	-1.0625	0.0108

GID: Gene identifier; log_2_ FC: base _2_ logarithm of the fold change; *p*_adj_: adjusted *p*-value; NTSI: Novel Transcript, Sense Intronic.

**Table 3:** Differentially expressed genes in the M+B- vs. M-B- comparison.

Upregulated				Downregulated			
GID	Gene ID	log_2_ FC	*p*_adj_	GID	Gene ID	log_2_ FC	*p*_adj_
ENSG00000245832.8	*MIR4300HG*	2.6632	0.0499	ENSG00000189052.7	*CGB5*	-2.4383	0.0029

GID: Gene identifier; log_2_ FC: base 2 logarithm of the fold change; *p*_adj_: adjusted *p*-value.

**Table 4:** Differentially expressed genes in the M+B+ vs. M+B- comparison.

Upregulated				Downregulated			
GID	Gene ID	log_2_FC	*p*_adj_	GID	Gene ID	log_2_FC	*p*_adj_
ENSG00000304767.1	NTSI to CEMIP	2.8822	0.0052	ENSG00000245832.8	*MI R4300HG*	-2.9724	0.0049
ENSG00000189052.7	*CGB5*	2.4656	0.0037	ENSG00000162706.13	*CADM3*	-2.4160	0.0049
				ENSG00000172260.15	*NEGR1*	-1.6713	0.0117
				ENSG00000162493.17	*PDPN*	-1.2298	0.0049
				ENSG00000291006.1	*LOC730101*	-1.0561	0.0037
				ENSG00000140937.14	*CDH11*	-1.0464	0.0049

GID: Gene identifier; log_2_ FC: base 2 logarithm of the fold change; *p*_adj_: adjusted *p*-value; NTSI: Novel Transcript, Sense Intronic.

**Comparison between M+B+ and M−B−:** Twenty DEGs were identified between both sample groups (**Figure 2**; **Table 2**), 16 were downregulated and 4 upregulated. Among the latter, two corresponded to long non-coding RNAs and one to an uncharacterized gene. The remaining upregulated gene was *CEMIP* (cell migration-inducing hyaluronidase 1), involved in hyaluronan catabolism and in positive regulation of protein phosphorylation and intracellular transport processes, likely mediated through its association with clathrin-coated vesicles during endocytosis (Spataro et al., 2023).

Surprisingly, most DEGs were downregulated in M+B+ placentas compared to M−B− non-infected controls; the highest negative fold change corresponded to *PRRX1* (paired related homeobox 1), a transcriptional coactivator implicated in cell growth and differentiation (Grueneberg et al., 1992). Other DEGs were *CADM3* (cell adhesion molecule 3) (Narita et al., 2011) and *CDH11* (cadherin 11), a calcium-dependent transmembrane adhesion protein (Alimperti and Andreadis, 2015). Genes associated with inflammatory response regulation were also suppressed, including *NLRP3* (NLR family pyrin domain containing 3), a key component of the inflammasome complex that interacts with ASC/PYCARD (Balci and Acar, 2024), and *F3* (coagulation factor III), a membrane-bound glycoprotein that initiates coagulation cascades (Zelaya et al., 2018). In addition, genes involved in trophoblast invasion, proliferation and syncytialization were downregulated, such as *HAND2* (heart and neural crest derivatives expressed 2), a basic helix-loop-helix transcription factor (Mestre-Citrinovitz et al., 2015; Yu et al., 2018; Šućurović et al., 2020) and *FGF10* (fibroblast growth factor 10), which plays critical roles in embryonic development, morphogenesis, and tissue repair (Natanson-Yaron et al., 2007; Rochais and Kelly, 2024).

**Comparison between M+B− and M−B−**: This comparison identified two significantly altered transcripts (**Figure 2**; **Table 3**): *MIR4300*, a long non-coding RNA, upregulated, and *CGB5* (chorionic gonadotropin subunit beta 5) that encodes a protein produced by trophoblastic cells with a key role in stimulating ovarian steroidogenesis (Jameson and Hollenberg, 1993; Rull and Laan, 2005), downregulated.

**Comparison between M+B+ and M+B-**: This comparison showed 8 DEGs: 2 upregulated and 6 downregulated (**Figure 2**; **Table 4**). The former ones included a novel long non-coding RNA located within the intronic region of *CEMIP*, and *CGB5*, above described.

Among the downregulated genes, several are associated with cell adhesion processes: *NEGR1* (neuronal growth regulator 1), predicted to localize to the extracellular region and plasma membrane (Miyata et al., 2000); *PDPN* (podoplanin), a cell-surface glycoprotein involved in cell adhesion, chemotaxis and the maturation of lymphatic and vascular structures (Onak Kandemir et al., 2019); as well as *CDH11* and *CADM3*, described above.

### Functional enrichment analysis

**Figure 3:**
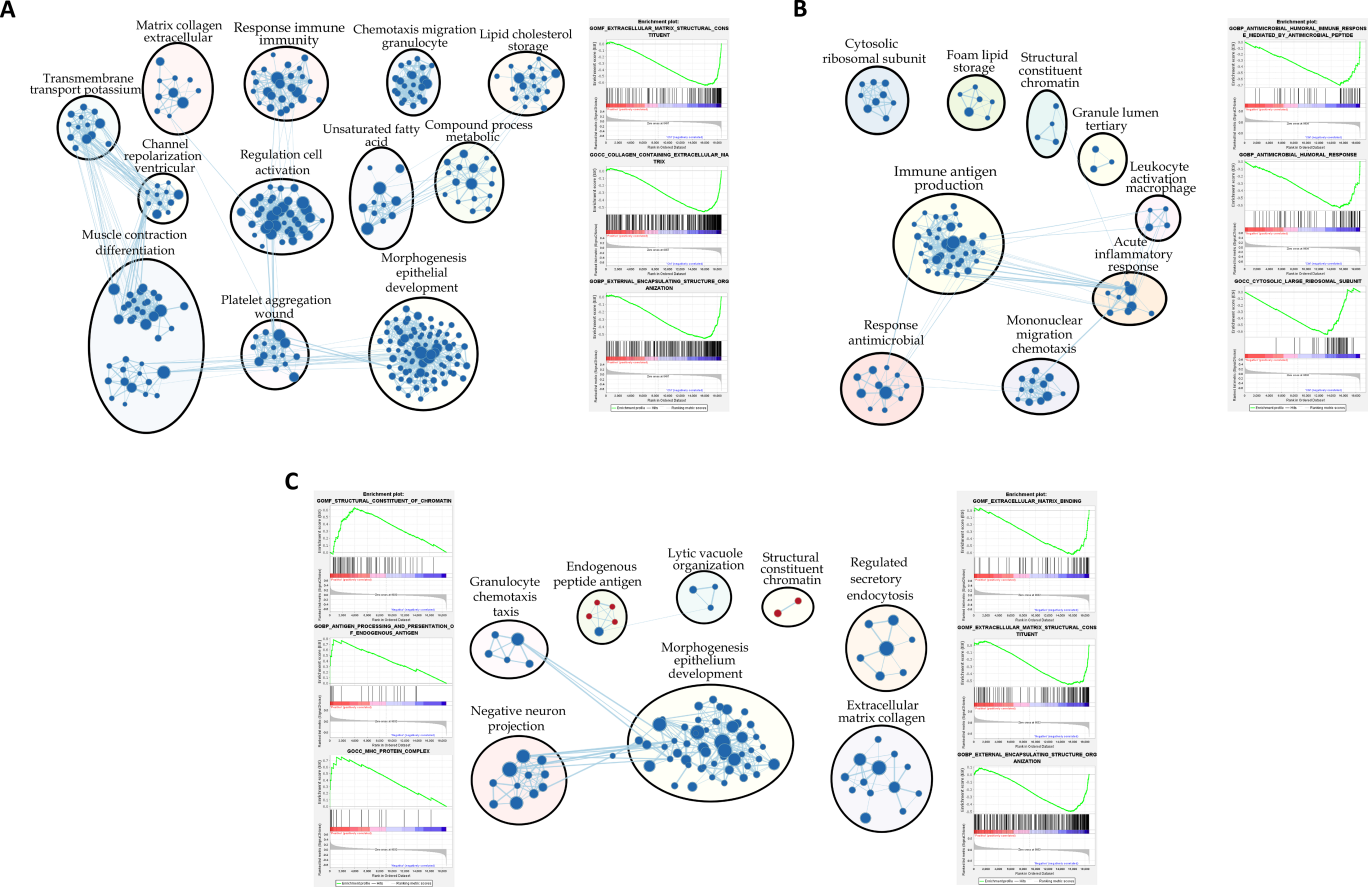
Gene Set enrichment analysis: Gene Ontology (GO) terms. Enrichment maps are shown for **(A)** M+B+ vs. M-B-, **(B)** M+B- vs. M-B-, and **(C)** M+B+ vs. M+B-. The maps display significantly altered (negatively and positively enriched ) GO terms with an FDR < 10%. Node color represents the Normalized Enrichment Score (NES), where red indicates positive enrichment and blue indicates negative enrichment. Related gene sets (clusters) are connected by light blue lines. GSEA results were visualized using Cytoscape. Representative enrichment plots for the three **(A, B)** or six **(C)** most significantly altered biological processes (FDR < 10%) are displayed flanking the maps.

Gene set enrichment analysis (GSEA) using Gene Ontology (GO) M+B+ and M−B− placenta (**Figure 3A**; **Supplementary Table S2**) revealed predominantly downregulated biological processes, particularly those involved in extracellular matrix organization, cell migration, and inflammation linked to chemotaxis. Similarly, in M+B− vs. M−B− comparison (**Figure 3B**; **Supplementary Table S3**), underrepresented pathways were also dominant, including gene sets related to innate immune defense, inflammatory regulation, and pathogen recognition. Finally, comparison between M+B+ and M+B− placental samples (**Figure 3C**; **Supplementary Table S4**) showed a predominance of underrepresented pathways over overrepresented ones. Among the latter, two terms related to the immune system reached an FDR <10% and six reached an FDR <25%, encompassing processes involved in antigen presentation, immune regulation, and intracellular functions such as ER–Golgi vesicular transport and nucleosomal DNA organization. In contrast, underrepresented pathways included thirteen terms with FDR <10% and 37 with FDR <25%, which were mainly associated with embryonic development, extracellular matrix organization (including collagen fibril assembly and basement membrane formation), and innate immune functions such as macrophage chemotaxis, phagosome maturation, and NK cell differentiation.

**Figure 4:**
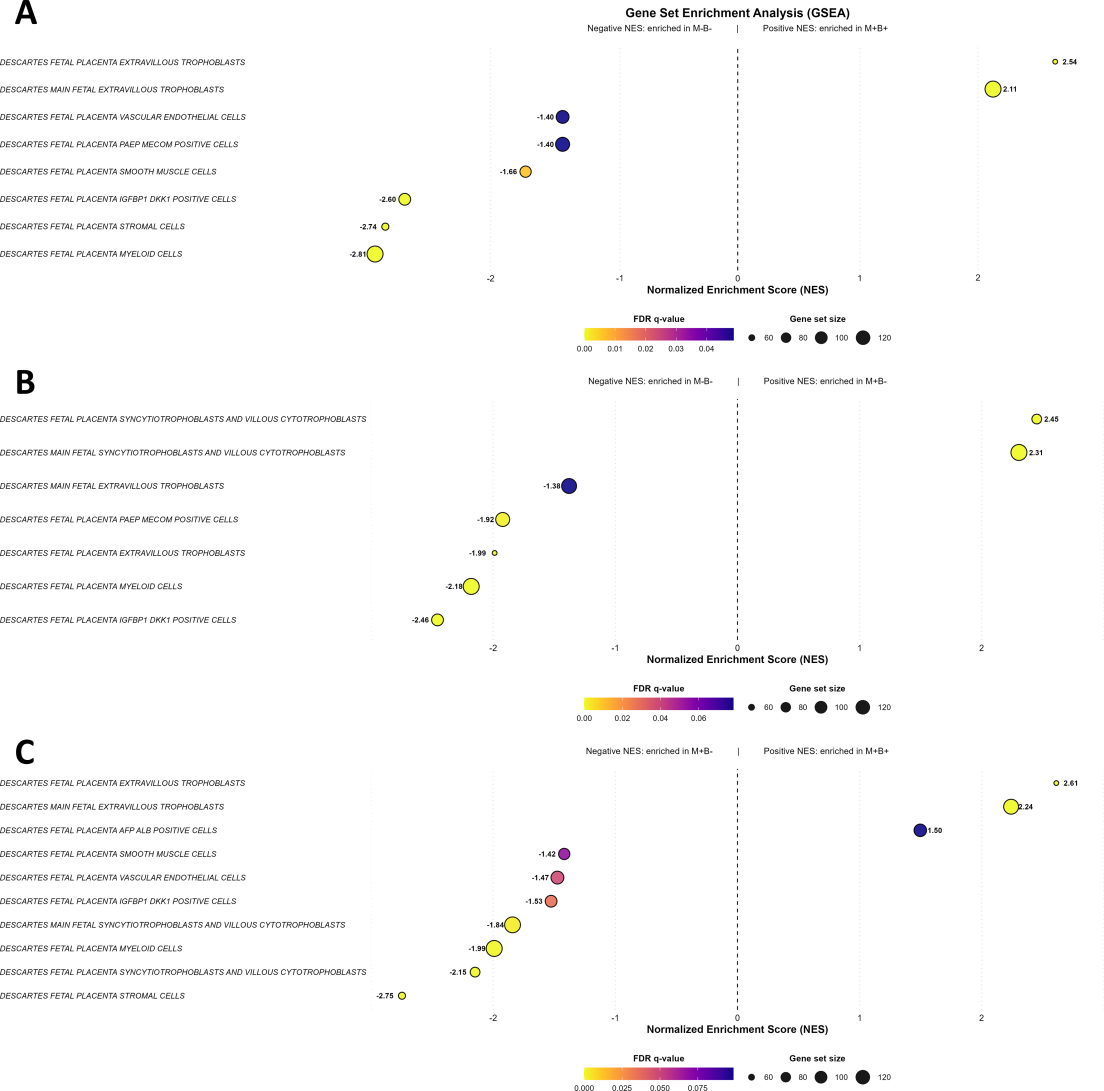
Gene set enrichment analysis: Cell-type signature collection. Dot plots of M+B+ vs.M-B- **(A)**, M+B- vs. M-B- **(B)** and M+B+ vs. M+B- **(C)**. The charts show Gen sets of negatively or positively enriched Cell-type terms with a False Discovery Rate (FDR) < 10% associated to the compared placental groups. The normalized enrichment score (NES) is represented on the x-axis. The FDR values span from 0 (yellow) to 0.1 (blue) and the sizes of the dots indicate those of the Gene sets ranging from 60 to 120.

We have also performed GSEA using the Cell-Type signature collection, in order to identify the cell types active in the different clinical groups (**Figure 4**; **Supplementary Tables S5**-**S7**). This analysis showed that gene sets associated with extravillous trophoblasts were positively enriched in M+B+ placentas in comparison to the other two groups. In contrast, M+B− placental gene sets were negatively enriched when compared to the M−B− group.

When compared to the M−B− controls, both *T. cruzi*-infected gene sets groups (M+) showed negatively enriched cell types related to the placental immune system, regardless of the status of infection transmission. Specifically, negative enrichment was observed in the fetal placenta myeloid cells, placenta PAEP MECOM positive cells and placenta IGFB1 DKK1 positive cells gene sets (**Figure 4**; **Supplementary Tables S5**-**S7**).

### Gene co-expression network analysis

**Figure 5:**
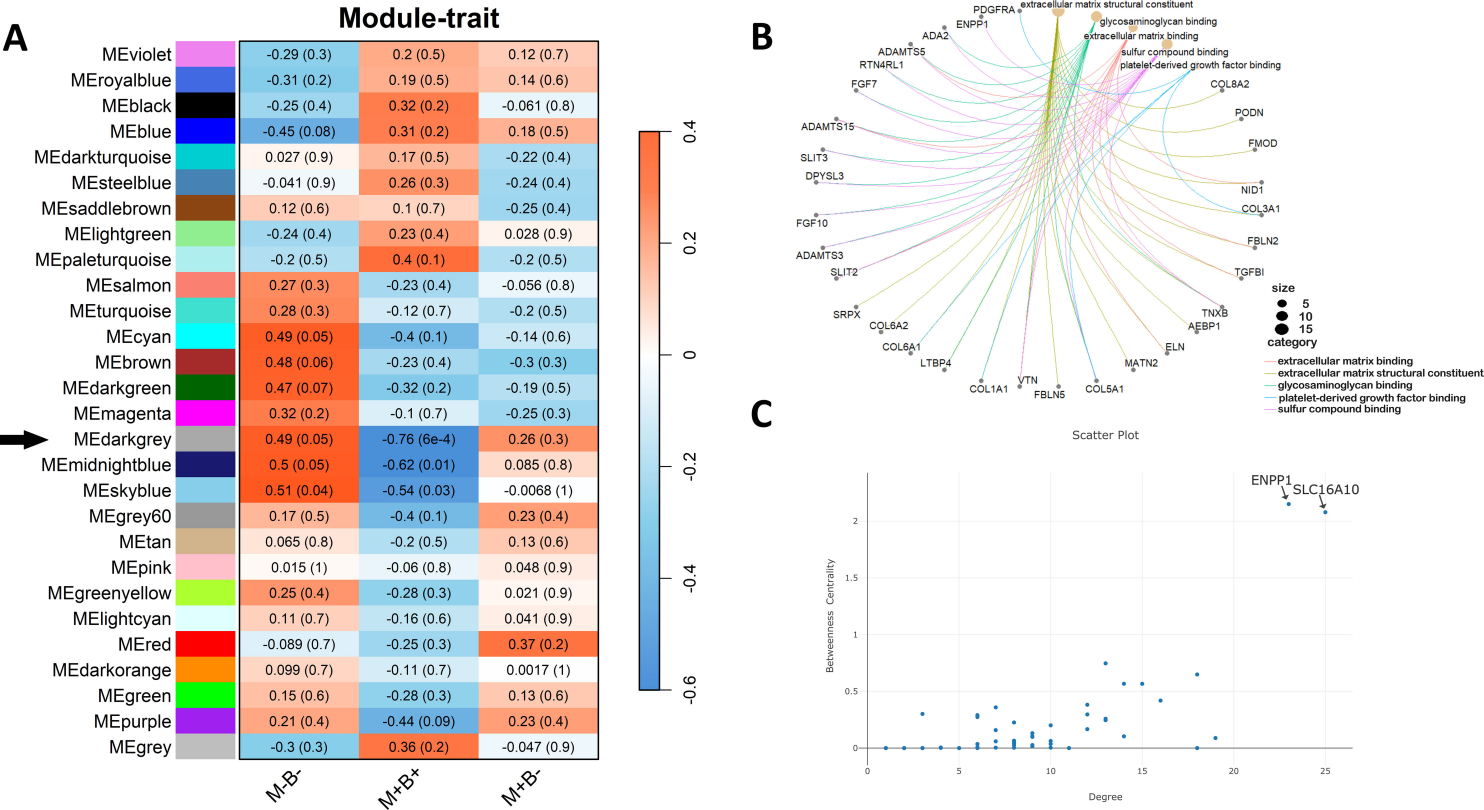
Network analysis of the dark grey module associated with the MB clinical groups. **(A)** Heatmap of module–trait relationships from WGCNA. Colors represent Pearson correlation coefficients between module eigengenes and clinical traits (red, positive correlation; blue, negative correlation). Numbers within each cell indicate the correlation coefficient (r), with the corresponding p-value shown in parentheses. **(B)** Functional enrichment of genes from the dark grey module (Black arrow). **(C)** Hub gene analysis within the module. The circular plot shows functional connections, and the scatter plot highlights key hub genes with high degree of betweenness centrality.

Co-expression network analysis identified three key modules—designated as dark grey, sky blue, and midnight blue—displaying differential associations across the study groups. These modules showed a strong positive correlation with the M-B- group and a strong negative correlation with the M+B+ group (**Figure 5A**). This pattern suggests an opposite transcriptional behavior of the same gene sets depending on the clinical phenotype. Accordingly, functional enrichment analysis of the dark grey module revealed a significant enrichment of genes associated with extracellular matrix organization, glycosaminoglycan binding, and growth factor interactions (**Figure 5B**), suggesting potential disruptions in cell–matrix communication and structural integrity in cases of cCD transmission. These criteria were defined based on established WGCNA strategies (Langfelder and Horvath, 2008) and adapted to the specific characteristics of the analyzed dataset. To prioritize the most biologically relevant module for downstream analyses, a multi-criteria selection strategy was applied. First, module-trait associations were evaluated using Pearson correlation, considering both the strength, direction, and statistical significance of the relationships with clinical groups. In this context, among the associated modules, the dark grey module exhibited a moderate positive correlation with M-B- (r = 0.49, p = 0.05) and a strong, highly significant negative correlation with M+B+ (r = -0.76, p = 6×10^-4^) (**Figure 5A**), reflecting a coherent and biologically meaningful inverse association with the transmission status. Second, module-level differential expression was assessed, prioritizing modules in which a substantial proportion of genes were differentially expressed in the comparison M+B+ vs. M-B-, indicating high internal transcriptional coherence. Consistent with this criterion, the dark grey module showed a pronounced enrichment of downregulated genes, consistent with its negative eigengene-trait correlation. Third, biological relevance was evaluated through Gene Ontology annotation and functional enrichment analyses.

Moreover, two hub genes, *ENPP1* and *SLC16A10*, were identified based on high intramodular connectivity measures following WGCNA recommendations (**Figure 5C**). *ENPP1* (ectonucleotide pyrophosphatase/phosphodiesterase 1) is a transmembrane glycoprotein, member of the ecto-nucleotide pyrophosphatase/phosphodiesterase (ENPP) family that hydrolyzes extracellular ATP (eATP) and generates AMP and pyrophosphate (Ansh et al., 2024) and *SLC16A10* is a solute carrier family 16 member 10 also known as TAT1, is a Na⁺‑independent transporter of aromatic amino acids across the plasma membrane (Ajmeriya et al., 2025). Both genes are expressed in trophoblast layers. As central nodes in the co-expression network, these genes may play pivotal roles in the biological pathways altered during congenital infection.

## Discussion

Although the incidence of vertical transmission of *T. cruzi* is relatively low (Rendell et al., 2015), it remains the main transmission route due to successful vectorial control, transfusion and transplant-related cases (Carlier et al., 2019; Colombo et al., 2021). Congenital CD has declined over recent decades, particularly in the areas of this study. In Chaco Province, the incidence in the period 2014–2015 was 7.1% at Hospital Perrando (Benatar et al., 2021), and in Salta Province approximately 1.6% in 2021 (unpublish data). Due to socioeconomic constraints of this vulnerable population, the cCD diagnostic algorithm could not be achieved in 58 (35.8%) newborns—a limitation previously reported and exacerbated during the COVID-19 pandemic (Gürtler et al., 2003; De Rissio et al., 2010; Danesi et al., 2019). Nevertheless, 12 placental samples were collected from a total of 162 *T.cruzi* infected pregnant women who transmitted cCD to their offsprings.

Placental structure and function rely on tightly regulated processes, including continuous syncytiotrophoblast turnover, vascular remodeling by extravillous trophoblasts, and finely tuned immune regulation to prevent fetal rejection and protect the fetus against infection. These coordinated processes result in a complex, cell type–specific transcriptional landscape (Li et al., 2020).

### Differentially expressed gene analysis

#### M+B+ vs. M−B− comparison

This comparison revealed 20 DEGs, a quarter of them being ncRNAs, emphasizing their emerging relevance in placental gene regulation. This aligns with recent findings that implicate lncRNAs in key trophoblast functions, including proliferation, migration, invasion, and apoptosis (Monteiro et al., 2021; Adu-Gyamfi et al., 2024).

**Structural and functional alterations of the placental barrier**. Among the upregulated transcripts, two were particularly notable: *CEMIP* (cell migration–inducing hyaluronidase 1) and a novel long non-coding RNA (ENSG00000304767) located within an intron of the *CEMIP* gene. *CEMIP* is involved in extracellular matrix remodeling and intracellular trafficking through clathrin-mediated endocytosis, and has been linked to trophoblast migration and invasiveness (Natanson-Yaron et al., 2007; Li et al., 2021; Spataro et al., 2023; Rochais and Kelly, 2024). The coordinated upregulation of *CEMIP* and its intronic lncRNA suggests a potential cis-regulatory interaction that may contribute to placental remodeling processes associated with congenital *T. cruzi* transmission.

Notably, the intronic transcript ENSG00000304767 maps to a genomic region enriched in enhancer-associated epigenetic marks and overlaps the predicted enhancer ENSR15_BL2T2, which has been implicated in the regulation of *CEMIP* (**Supplementary Figure 4**). Its genomic localization and expression profile are consistent with a potential role as an enhancer RNA, capable of modulating *CEMIP* transcription or that of nearby genes, although direct experimental validation will be required. The coordinated overexpression of ENSG00000304767 and *CEMIP* observed in this study supports the existence of a cis-regulatory lncRNA–host gene axis that may influence villous extracellular matrix (ECM) organization. Altered hyaluronan turnover and stabilization of high–molecular-weight hyaluronan could modify the physical and biochemical properties of the trophoblast and villous stroma, with potential consequences for trophoblast migration, vascular remodeling, and leukocyte trafficking. Such ECM remodeling may generate localized changes in barrier integrity or immune cell composition that could facilitate *T. cruzi* traversal of the placental barrier, while also possibly reflecting a compensatory host response to chronic infection aimed at reinforcing placental defense. Dissecting the ENSG00000304767–*CEMIP* regulatory axis in trophoblast-based models may therefore provide mechanistic insight into how ECM dynamics shape susceptibility to congenital transmission.

The involvement of structural pathways in congenital transmission is supported by the fact that several genes associated with cell adhesion were downregulated. These included CADM3 and CDH11. CADM3 encodes a calcium-independent adhesion molecule capable of both homophilic and heterophilic interactions via nectin dimerization that participates in the formation and organization of adherens and tight junctions in epithelial barriers (Narita et al., 2011; Yamada et al., 2013). Although CADM3 has been linked to preeclampsia (Ashar-Patel et al., 2017; Gao et al., 2023), its specific function in placental tissue remains poorly characterized. Nevertheless, its established role in junctional organization suggests that reduced CADM3 expression in M+B+ placentas (this study) might be associated with weakened cell–cell adhesion within the trophoblast layer and/or the fetal endothelium. Such junctional loosening could facilitate paracellular passage of the parasites.

CDH11, a type II classical cadherin, is expressed in syncytiotrophoblasts and extravillous cytotrophoblasts and plays a calcium-dependent role in cell–cell adhesion. It contributes to the epithelial–mesenchymal transition required for anchoring to the decidua (MacCalman et al., 1996; Kokkinos et al., 2010). Notably, CDH11 expression increases during trophoblast syncytialization, and its overexpression has been shown to suppress proliferation and enhance differentiation (Getsios and MacCalman, 2003). Reduced CDH11 expression in M+B+ placentas may therefore impair normal syncytiotrophoblast formation or trophoblast–decidua interactions, leading to focal defects in barrier continuity or abnormal villous anchoring, providing routes of entry that favor *T. cruzi* passage to the fetal compartment. PRRX1 (paired related homeobox 1) exhibited the highest downregulation. Its protein acts as a transcriptional co-activator, boosting the DNA-binding capacity of serum response factor, which in turn activates genes in response to growth and differentiation signals (Grueneberg et al., 1992). PRRX1 also promotes the expression of TGFβ3 and fibrillar collagen genes (Jiang and Stefanovic, 2008) and regulates mRNA expression of COL6A3 (collagen type VI alpha 3 chain) in adipose cells (Dankel et al., 2020), indicating that its function is closely related to the extracellular matrix.

Evidence from *in vitro* (Luján et al., 2004; Díaz-Luján et al., 2012; Fretes and Kemmerling, 2012) and histopathological studies of placentas from seropositive women (Mezzano et al., 2022) indicates that *T. cruzi* infection induces placental tissue damage, including syncytiotrophoblast detachment, which may facilitate parasite invasion. Other studies reported destruction of the syncytiotrophoblast and villous stroma, along with selective disruption of the basal lamina and collagen I organization (Duaso et al., 2012). In the present study, downregulation of genes involved in trophoblast function, such as HAND2 and FGF10, suggests impairment of epithelial turnover and extravillous trophoblast proliferation and invasion —the main defense mechanisms reported to protect against infection (Liempi et al., 2014; Liempi et al., 2016). HAND2 encodes a transcription factor implicated in trophoblast invasion, proliferation and syncytialization (Mestre-Citrinovitz et al., 2015; Yu et al., 2018; Šućurović et al., 2020), in addition to its role in regulating the timing and progression of parturition (Wu et al., 2018; Nikolakopoulou and Turco, 2021). In fact, dysregulation of HAND2 expression or signaling has been associated with preterm birth and preeclampsia (Wu et al., 2020; Zhu et al., 2025).

LINC02268 (ENSG00000248174) was reported to co-express itself with other genes and is potentially involved in activation of IL-6/JAK/STAT3 pathways in cancer (Wei et al., 2021). GeneCards predictions suggest it could regulate HAND2 (among others); interestingly, HAND2 appeared downregulated in our study (**Table 2**).

Notably FGF10 is involved in embryonic development with a complex spatiotemporal expression pattern, and in adults its continuous expression has functions in the regulation of homeostasis and tissue repair (Rochais and Kelly, 2024). In the placenta, FGF10 promotes invasion and outgrowth of trophoblasts (Natanson-Yaron et al., 2007), and its expression was found in decidual cells and in cytotrophoblasts of the cytotrophoblast columns during all three trimesters, suggesting a function in the maternal-fetal interface (Anteby et al., 2005).

**Alteration of the innate immune responses at the placental interface**. Downregulated NLRP3 and F3 evidenced an altered immune status compared to controls. Notably, the inflammasome activation by NLRP3 has been reported as essential to control *Leishmania*, *Toxoplasma gondii* and *T. cruzi* infections. On the contrary, inactivation of this pathway inhibits the differentiation of T-cell responses and killing of *T. cruzi* by macrophages, resulting in greater parasite burdens (Alonaizan, 2024). Additionally, parasite–macrophage interaction may trigger TF release, promoting coagulation, inflammation, and frequent thrombotic events in CD patients (Choudhuri and Garg, 2022). In our study, the low expression of NLRP3 suggests limited inflammation, which in turn would favor the development of the infection and consequently congenital transmission.

Importantly, cell type–based enrichment analyses indicate that the reduction of innate immune–related placental cell signatures is a shared feature of placentas from *T. cruzi–*infected mothers, regardless of congenital transmission outcome. This suggests that immune attenuation represents a baseline placental adaptation to chronic maternal infection, whereas additional structural and trophoblast-specific alterations may be required to allow parasite transmission to the fetus.

#### M+B− vs. M−B− comparison

**MicroRNA-associated regulation and trophoblast hormonal dysfunction**. RNA-seq analysis identified only two DEGs, MIR4300HG, a long non-coding RNA that hosts MIR4300 upregulated (FC = 2.66), and CGB5 downregulated. Although the function of MIR4300 remains unclear, it is known that microRNAs broadly regulate gene expression and biological processes (Jorge et al., 2021), and while the mature microRNA was not directly detectable due to its small size, its expression is potentially occurring. Using miRWalk 3.0, CGB5 was predicted as a potential MIR4300 target, with binding sites in its coding region, suggesting possible post-transcriptional repression. CGB5 encodes the β-subunit of human chorionic gonadotropin (β-hCG), a hormone predominantly expressed in trophoblasts through CGB3, CGB5, or CGB8 (Rull and Laan, 2005). Its underexpression is consistent with previous findings (Juiz et al., 2018) in non-transmitting placentas. It has been shown that β-hCG production was increased in human placental chorionic villus explants and *in vitro* using the human trophoblast cell line BeWo infected with the Ypsilon strain (classified as discrete typing unit Tc II) (Liempi et al., 2014; Carrillo et al., 2016). In contrast, human chorionic villi explants challenged with an isolate from a cCD case (Lucky, Tc II/VI) and the Tulahuen strain (Tc VI) showed a decrease in β-hCG production (Triquell et al., 2018). Since β-hCG is considered an indicator of the vitality of syncytiotrophoblast (Cole, 2010), our transcriptomic data suggest a functional alteration of the placental barrier.

#### M+B+ vs M+B− comparison

**Alterations in trophoblast differentiation, cell adhesion, and placental barrier integrity**. This comparison revealed that in M+B+ the long non-coding RNA intronic to CEMIP gene as well as CGB5 were upregulated while CADM3, NEGR1, PDPN, and CDH11, among others, were downregulated. As previously mentioned, CADM3 is related to cell adhesion, as well as NEGR1 and PDPN, while CDH11 is involved in trophoblast differentiation. NEGR1 is a membrane-bound protein involved in intercellular adhesion, widely studied in the brain which is also expressed in the placenta, where its specific function remains unknown (Breton et al., 2020). Podoplanin, PDPN, is a cell-surface receptor implicated in cell adhesion, chemotaxis, and lymphatic and vascular maturation (Quintanilla et al., 2019). In human placentas, it is mainly expressed in decidual cells and chorionic villous stromal cells, during pregnancy (Onak Kandemir et al., 2019). Interestingly, the authors propose that low levels of PDPN could cause inflammation in the villi, at least in the context of molar pregnancies (Onak Kandemir et al., 2019). These results suggest that differences between parasite transmission and non-transmission in infected placentas might rely on trophoblast differentiation and functionality, cell adhesion, and ECM integrity.

### Gene set enrichment analysis

#### M+B+ vs. M-B- comparison

GSEA showed that most negatively enriched terms in the M+B+ group are related to the extracellular matrix, particularly collagen organization and metabolism. Consistently, analysis of downregulated DEGs highlighted pathways associated with cell adhesion and extracellular matrix organization, processes that have been previously linked to trophoblast detachment and ECM reorganization in response to *T. cruzi* infection (Luján et al., 2004; Díaz-Luján et al., 2012; Duaso et al., 2012; Fretes and Kemmerling, 2012; Mezzano et al., 2022).

Gene sets associated with metallopeptidase activity—enzymes that degrade proteins through a metal ion in their active site—were also underrepresented. This finding supports previous evidence linking two MMP2 gene polymorphisms with the occurrence of cCD (Juiz et al., 2016). Polymorphisms that reduce metalloproteinase transcription may impair the placental response to infection, given the role of these enzymes in ECM degradation, epitope processing and modulation of immune and inflammatory responses (Geurts et al., 2012; Smigiel and Parks, 2017). Our study suggests reduced leukocyte migration by the enrichment of terms such as GOBP_LEUKOCYTE_CHEMOTAXIS and GOBP_MYELOID_LEUKOCYTE_MIGRATION. In turn, this impairment may be caused by decreased metalloproteinase activity and ECM remodeling, as effective leukocyte infiltration requires both matrix degradation and the generation of chemotactic signals derived from metalloproteinase-mediated proteolysis (Geurts et al., 2012). Finally, placentas from M+B+ mothers showed an overall underrepresentation of the immune response compared to M-B-. Several gene sets related to innate and adaptive immune responses had negative NES values, and those related to the inflammatory response were particularly diminished, potentially facilitating *T. cruzi* placental transmission (Benizio et al., 2023).

#### M+B- vs. M-B- comparison

GSEA revealed that the gene sets most prominently affected were related to the humoral immune response, particularly those mediated by antimicrobial peptides, which overlapped with signatures previously reported (Juiz et al., 2018). Our findings differ from those reported by Juiz et al. for the M+B− group. This discrepancy may reflect several factors known to influence placental transcriptomic profiles, including differences in study population characteristics such as sample size and geographic origin. The latter may be associated with the circulation of distinct *Trypanosoma cruzi* genotypes as well as with differences in host genetic background, both key components of the host–parasite interaction. In fact, Juiz et al. analyzed pooled placental samples rather than individual placentas and included women from a different endemic region, potentially associated with different *T. cruzi* populations. Importantly, different *T. cruzi* strains may display distinct levels of virulence and placental tissue tropism, which could further modulate placental responses. This notion is supported by previous studies that demonstrated strain-dependent differences in placental infection and host responses using both an *in vivo* murine model (Juiz et al., 2017) and a 3D human trophoblast cell line model (Apodaca et al., 2024). Both parasite strain and parasite burden have been shown to influence placental transcriptional responses. In line with this, transcriptomic analyses of human chorionic villus explants experimentally infected with the *T. cruzi* Y strain at varying parasite concentrations and incubation times revealed that immune-related pathways can be either positively or negatively enriched depending on parasite load and stage of infection, underscoring the dynamic and context-dependent nature of the placental immune response to *T. cruzi* (Castillo et al., 2018).

#### M+B+ vs. M+B- comparison

GSEA revealed increased antigen processing and presentation in M+B+ placentas, while pathways linked to the innate immune response and ECM organization and collagen-related processes were reduced. This nuanced immune-related pattern may reflect two distinct aspects of the placental response to *T. cruzi*. On the one hand, the positive enrichment of pathways involved in antigen processing and presentation in the M+B+ group may indicate the physical presence of the parasite within placental tissue. On the other hand, pathways associated with innate immune responses—including macrophage activity, NK cell differentiation, and phagosome maturation—were negatively enriched, together with processes related to extracellular matrix organization and integrity. Collectively, this transcriptional profile may reflect a compromised placental barrier, facilitating parasite transmission from mother to fetus. Nevertheless, further studies will be required to elucidate the complex interplay between these regulatory mechanisms.

Cell type enrichment analysis indicated decreased syncytiotrophoblast and cytotrophoblast signatures in transmitting cases, suggesting impaired epithelial renewal, a process thought to protect against vertical transmission (Liempi et al., 2016). Additionally, gene sets related to development and morphogenesis were significantly downregulated in the transmitting group. This aligns with clinical observations of shorter gestation in infected newborns as well as low birth weight, Apgar scores < 7, and increased rates of premature membrane rupture in babies born to seropositive mothers compared to controls (Torrico et al., 2004). GSEA using the Cell-type signature collection identified differential expression patterns in villous, extravillous and syncytiotrophoblasts, suggesting that shifts in specific trophoblast populations might be associated with susceptibility or resistance to transplacental transmission (**Figure 4**, **Supplementary Tables S5**-**S7**).

### Gene co-expression network analysis

Chen et al (Chen et al., 2008). demonstrated the utility of performing Gene co-expression analysis in metabolic syndrome, where co-expression networks revealed biologically relevant modules perturbed by disease-associated loci. Although applied in a different biological context, their findings underscore the value of network-based strategies that can also be applied to study placental responses to infection. In our study, 3 key modules (dark grey, sky blue and midnight blue) showed correlations with the clinical groups. Among them, the dark grey module was prioritized for further analysis because it showed the strongest concordance between co-expression patterns, differential expression and biological function. Notably, the eigengene expression of this module exhibited an inverse correlation with the occurrence of *T. cruzi* vertical transmission, indicating that genes within this module are upregulated in controls and downregulated in M+B+ (Langfelder and Horvath, 2008; Apodaca et al., 2024). Furthermore, a substantial proportion of DEGs between M+B+ and M-B-placentas (10 out of 16) overlapped with genes in this module. Collectively, these results suggest the existence of a coordinated transcriptional repression program linked to congenital *T. cruzi* transmission (Carlier et al., 2020). Additionally, functional enrichment analysis of the dark grey module revealed a significant overrepresentation of genes involved in ECM organization, glycosaminoglycan binding, and growth factor interactions, suggesting a disruption in cell-matrix communication and structural integrity in transmitting placentas, confirming the GSEA results (Nde et al., 2012). Finally, two transcripts were identified as hub genes: ENPP1 and SLC16A10. The protein encoded by ENPP1 has recently been associated with the innate immune response, among other functions, and it has been suggested that its variants are linked to different disease phenotypes (Ansh et al., 2024). Similarly, downregulation of SLC16A10 has been proposed to be linked to gestational disorders (Ajmeriya et al., 2025). These 2 genes deserve validation and investigation as potential biomarkers of transmission risk, especially taking into account that ENPP1 was also significantly downregulated in the DEGs analysis.

## Study limitations

It should be noted that all inferences drawn in this study are based exclusively on bioinformatic analyses of transcriptomic data.

Principal component analysis showed overlap among the clinical groups. This unclear separation has been also reported in previous placental transcriptomic studies (Lien et al., 2021; Xin et al., 2023; Akram et al., 2025) and is consistent with a low number of DEGs observed. In human placenta, a large proportion of global gene expression variance is driven by inter-individual differences and intra-tissue variability, which often exceed the variance explained by group-level factors (Hughes et al., 2015). In this context, unsupervised analyses such as PCA of placental bulk RNA-seq data often capture broad sources of variability related to tissue heterogeneity, cellular composition and additional biological or environmental factors (Yong and Chan, 2020) and do not necessarily maximize separation by specific clinical phenotypes (Yong and Chan, 2020). As discussed in guidelines for good practice in RNA-seq analysis (Dawadi et al., 2025), biologically relevant differences in complex human tissues are often more effectively identified using targeted differential expression and network-based approaches, rather than unsupervised global variance alone (Conesa et al., 2016).

The gene expression patterns and pathway signatures identified in our study are consistent with alterations in trophoblast–extracellular matrix (ECM) interactions and placental tissue remodeling in cases associated with congenital transmission. However, in the absence of histopathological or immunohistochemical validation, these findings should be interpreted as transcriptome-based inferences rather than direct evidence of trophoblast detachment or structural tissue damage. Notably, the pathways highlighted here are concordant with previous reports describing architectural alterations in *T. cruzi*-infected placentas from transmitting cases (Duaso et al., 2012), supporting the biological plausibility of the proposed mechanisms and underscoring the need for future studies integrating transcriptomics with experimental validation.

Among potential confounding variables, both the route of delivery and prior exposure to *Toxoplasma gondii* warrant consideration. The route of delivery was included as a selection criterion, and efforts were made to ensure comparable proportions of vaginal and cesarean deliveries across the three clinical groups. However, inclusion in the RNA-seq analysis ultimately depended on RNA quality metrics (e.g., RIN values), resulting in a limited number of samples per group and precluding a robust statistical assessment of delivery route effects on gene expression.

Regarding *T. gondii* coinfection, IgG seropositivity reflects past exposure, and no cases of active infection during pregnancy were identified. As shown in **Table 1**, all clinical groups included both seropositive and seronegative individuals, although an even distribution could not be achieved due to the mentioned above sample availability and quality constraints. To further assess the potential impact of prior *T. gondii* exposure on placental transcriptional profiles, IgG serostatus was included as a clinical trait in the co-expression network analysis. No significant associations were observed between *T. gondii* IgG status and any gene co-expression module (p < 0.05), and this variable was therefore excluded from the main module–trait heatmap (**Figure 5**).

## Conclusions

This transcriptomic study is the first to compare placental tissues from *T. cruzi*-infected mothers who transmitted cCD, those who did not, and non-infected controls. Although direct causal links between these transcriptomic alterations and congenital transmission remain to be demonstrated, their coordinated dysregulation observed in this study suggests a model in which subtle impairments across immune–metabolic balance, ECM architecture, and junctional integrity contribute to creating a microenvironment more permissive to *T. cruzi* passage.

Through an integrated analytical strategy, we identified both coding and non-coding genes with potential regulatory functions—most notably CEMIP and the intronic long non-coding RNA ENSG00000304767—alongside several genes implicated in cell adhesion, suggesting a possible compromise of the placental barrier integrity during transmission. GSEA revealed distinct functional alterations across groups, with transmission cases showing marked changes in extracellular matrix-related pathways and in key trophoblast cell types. These findings were further confirmed by co-expression network analysis, which highlighted ENPP1 and SLC16A10 as hub genes potentially involved in vertical transmission mechanisms.

Together, these results provide new molecular insights into placental biology in congenital *T. cruzi* transmission and identify candidate pathways for future functional and translational studies. These pathways warrant mechanistic investigation using trophoblast cell systems, three-dimensional placental models, and/or ex vivo villous explants, and may ultimately guide efforts to identify biomarkers of placental susceptibility to *T. cruzi* transmission.

## Data Availability

The datasets presented in this study can be found in online repositories. The names of the repository/repositories and accession number(s) can be found in the article/**Supplementary Material**.
