# Supplementary figures and images for: Placental transcriptome profiling in congenital Chagas disease: gene networks associated with transmission
Source: Front Cell Infect Microbiol. 2026 Mar 18;16:1749307. doi: 10.3389/fcimb.2026.1749307 (PMC13038943; doi:10.3389/fcimb.2026.1749307)

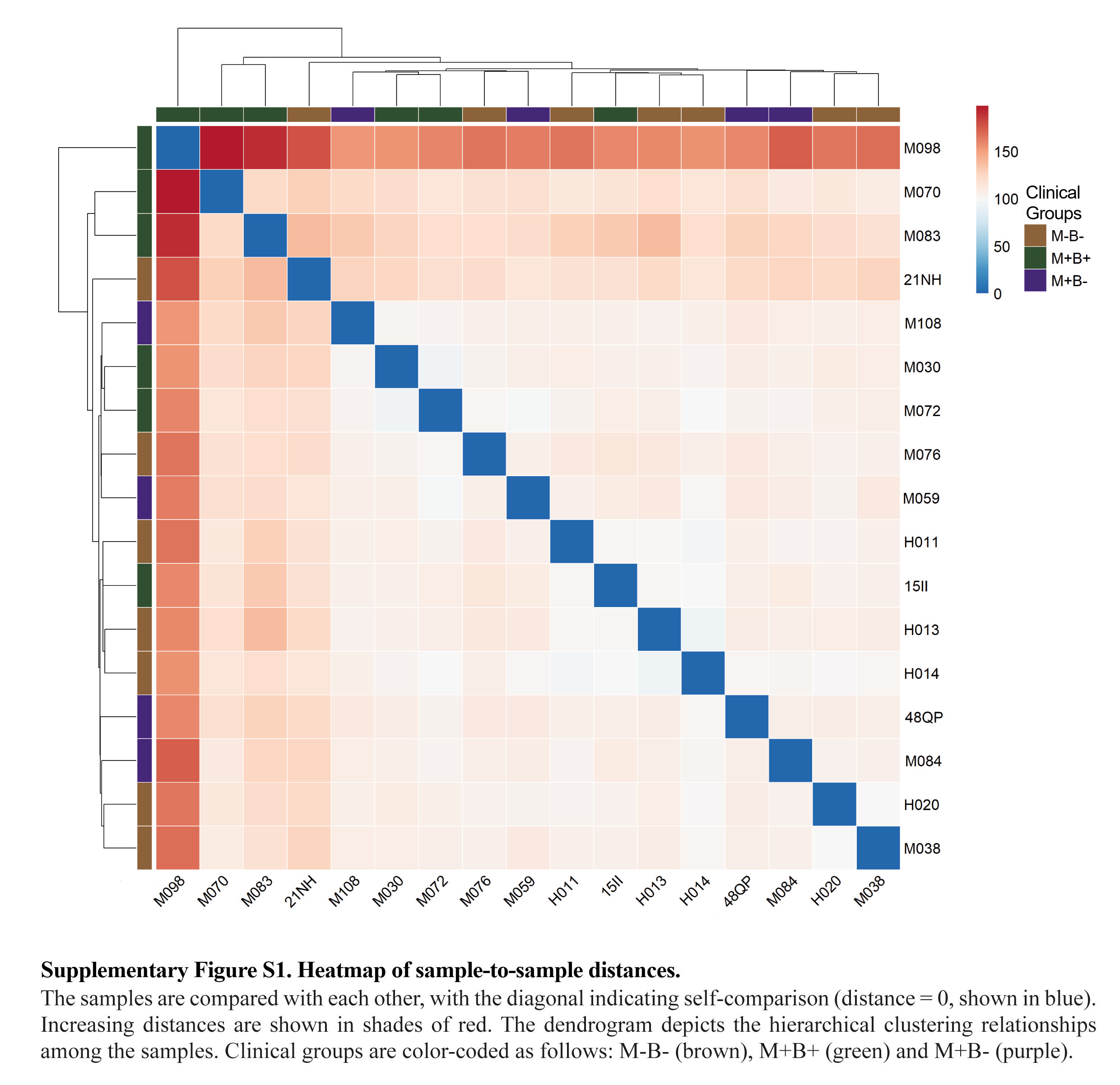

Supplement: Supplementary file 1 [file Image1.tif]

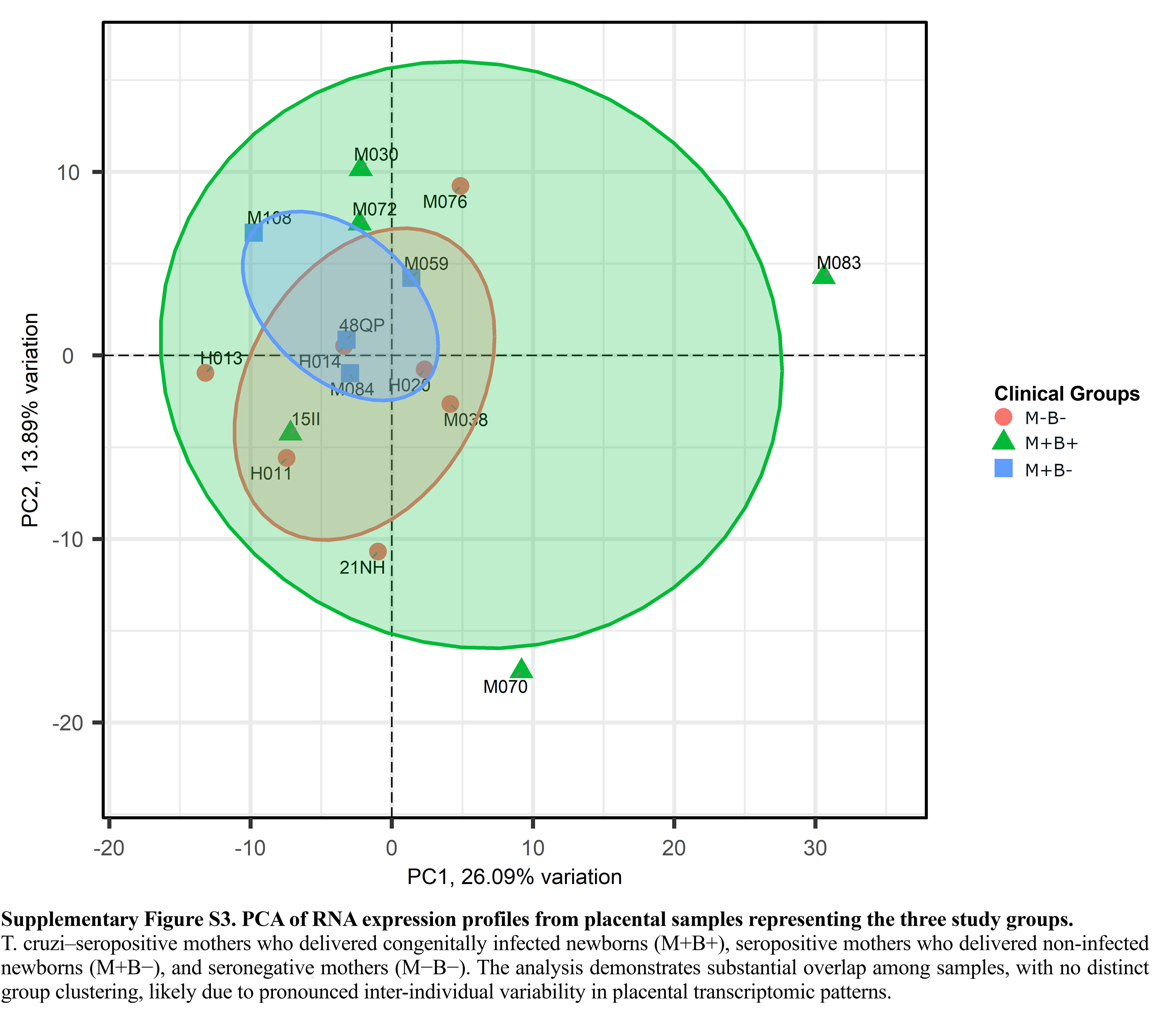

Supplement: Supplementary file 3 [file Image3.tiff]

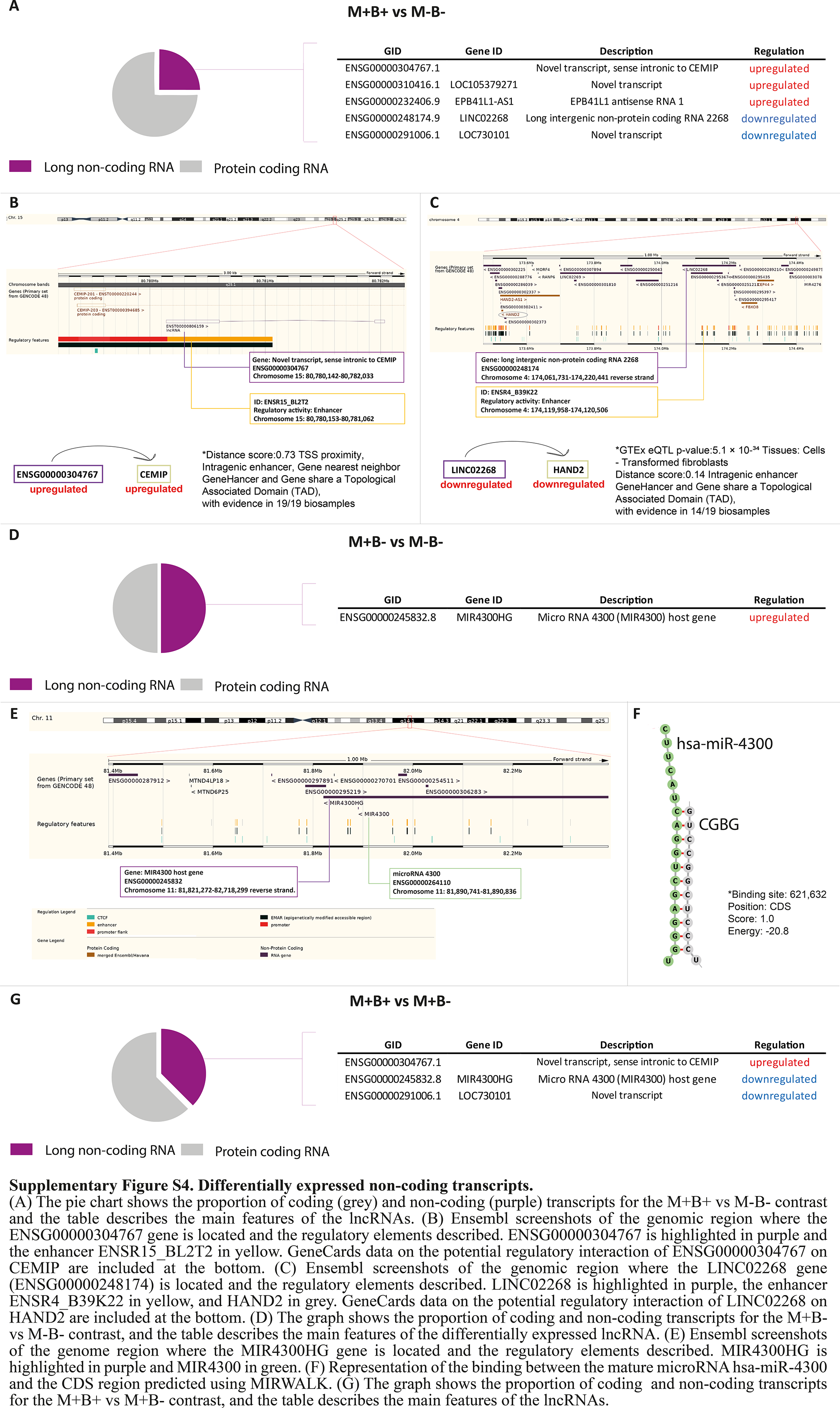

Supplement: Supplementary file 4 [file Image4.tiff]
