# Supplementary material for: Placental transcriptome profiling in congenital Chagas disease: gene networks associated with transmission
Source: Front Cell Infect Microbiol. 2026 Mar 18;16:1749307. doi: 10.3389/fcimb.2026.1749307 (PMC13038943; doi:10.3389/fcimb.2026.1749307)
Supplement: Supplementary file 5 [file Table1.pdf]

Supplementary Table 1. STAR mapping statistics summary for samples

Sample	Total Reads	Uniquely Mapped	Uniquely Mapped %	Multi Mapped	Multi Mapped %	Too Many Loci	Too Many Loci %	Unmapped Mismatches %	Unmapped Short %	Unmapped Other %	Chimeric Reads	Chimeric Reads %
**H014**	23,294,899	21,157,820	90.83	1,394,280	5.99	1,627	0.01	0	3.09	0.09	0	0
**M030**	21,872,157	19,606,764	89.64	1,308,025	5.98	1,916	0.01	0	4.26	0.11	0	0
**M083**	19,840,441	17,964,341	90.54	1,168,770	5.89	1,294	0.01	0	3.45	0.11	0	0
**H020**	20,545,855	18,832,852	91.66	1,090,196	5.31	1,194	0.01	0	2.92	0.10	0	0
**M076**	25,613,649	23,354,862	91.18	1,202,921	4.70	1,201	0.00	0	4.01	0.11	0	0
**21NH**	19,465,447	17,297,276	88.86	1,487,903	7.64	691	0.00	0	3.40	0.09	0	0
**M059**	21,222,720	19,277,441	90.83	1,156,406	5.45	1,543	0.01	0	3.59	0.12	0	0
**M108**	20,236,307	18,285,512	90.36	938,447	4.64	1,055	0.01	0	4.89	0.11	0	0
**M072**	26,693,881	24,035,311	90.04	1,472,070	5.51	1,706	0.01	0	4.31	0.13	0	0
**M038**	21,307,557	19,377,888	90.94	1,032,666	4.85	1,786	0.01	0	4.09	0.11	0	0
**16GB**	27,517,981	24,856,712	90.33	2,096,610	7.62	1,839	0.01	0	1.93	0.11	0	0
**15II**	22,045,664	19,796,371	89.80	1,542,724	7.00	1,259	0.01	0	3.11	0.09	0	0
**H011**	20,457,803	18,523,642	90.55	1,090,095	5.33	1,019	0.00	0	4.03	0.10	0	0
**H013**	19,867,503	18,162,935	91.42	934,957	4.71	837	0.00	0	3.77	0.09	0	0
**48QP**	19,050,881	17,158,638	90.07	1,106,355	5.81	1,692	0.01	0	4.00	0.12	0	0
**M084**	21,468,928	19,444,226	90.57	1,155,119	5.38	1,855	0.01	0	3.94	0.11	0	0
**M070**	21,672,834	19,537,691	90.15	1,150,001	5.31	1,200	0.01	0	4.44	0.10	0	0
**M098**	28,252,605	26,098,670	92.38	1,030,154	3.65	1,514	0.01	0	3.85	0.12	0	0
