# Supplementary material for: Placental transcriptome profiling in congenital Chagas disease: gene networks associated with transmission
Source: Front Cell Infect Microbiol. 2026 Mar 18;16:1749307. doi: 10.3389/fcimb.2026.1749307 (PMC13038943; doi:10.3389/fcimb.2026.1749307)
Supplement: Supplementary file 6 [file Table2.docx]

**Supplementary Table 2. GSEA results using GO library for M+B+ vs M-B- clinical groups.**

| **Gene sets enriched in M+B+** | **NES** | **FDR q-val** |
| --- | --- | --- |
| GOMF_OLFACTORY_RECEPTOR_ACTIVITY | 1.91 | 0.224 |
| GOMF_CYTOSKELETAL_MOTOR_ACTIVITY | 1.87 | 0.241 |
| GOBP_SENSORY_PERCEPTION_OF_SMELL | 1.85 | 0.211 |

| Gene sets enriched in M-B- | NES | FDR q-val |
| --- | --- | --- |
| GOMF_EXTRACELLULAR_MATRIX_STRUCTURAL_CONSTITUENT | -2.66 | 0.000 |
| GOCC_COLLAGEN_CONTAINING_EXTRACELLULAR_MATRIX | -2.58 | 0.000 |
| GOBP_EXTERNAL_ENCAPSULATING_STRUCTURE_ORGANIZATION | -2.48 | 0.000 |
| GOBP_LEUKOCYTE_CHEMOTAXIS | -2.44 | 0.000 |
| GOCC_EXTERNAL_ENCAPSULATING_STRUCTURE | -2.43 | 0.000 |
| GOBP_COLLAGEN_FIBRIL_ORGANIZATION | -2.41 | 0.000 |
| GOBP_CELL_CHEMOTAXIS | -2.40 | 0.000 |
| GOCC_COLLAGEN_TRIMER | -2.39 | 0.000 |
| GOBP_TAXIS | -2.37 | 0.000 |
| GOCC_ENDOPLASMIC_RETICULUM_LUMEN | -2.36 | 0.000 |
| GOBP_REGULATION_OF_EXTRACELLULAR_MATRIX_ORGANIZATION | -2.35 | 0.000 |
| GOBP_VASCULAR_ASSOCIATED_SMOOTH_MUSCLE_CELL_PROLIFERATION | -2.35 | 0.000 |
| GOBP_POSITIVE_REGULATION_OF_CHEMOTAXIS | -2.33 | 0.000 |
| GOMF_EXTRACELLULAR_MATRIX_BINDING | -2.33 | 0.000 |
| GOBP_EXTRACELLULAR_MATRIX_ASSEMBLY | -2.33 | 0.000 |
| GOBP_CELL_JUNCTION_DISASSEMBLY | -2.33 | 0.000 |
| GOBP_REGULATION_OF_CHEMOTAXIS | -2.28 | 0.000 |
| GOBP_MYELOID_LEUKOCYTE_MIGRATION | -2.28 | 0.000 |
| GOBP_REGULATION_OF_PLATELET_ACTIVATION | -2.25 | 0.000 |
| GOBP_REGULATION_OF_ANIMAL_ORGAN_MORPHOGENESIS | -2.25 | 0.000 |
| GOMF_HEPARIN_BINDING | -2.24 | 0.000 |
| GOBP_POSITIVE_REGULATION_OF_ENDOCYTOSIS | -2.24 | 0.000 |
| GOBP_MACROPHAGE_ACTIVATION | -2.24 | 0.000 |
| GOBP_LEUKOCYTE_MIGRATION | -2.22 | 0.000 |
| GOMF_GLYCOSAMINOGLYCAN_BINDING | -2.22 | 0.000 |
| GOBP_MONONUCLEAR_CELL_MIGRATION | -2.22 | 0.000 |
| GOBP_REGULATION_OF_LEUKOCYTE_MIGRATION | -2.22 | 0.000 |
| GOBP_CARDIAC_MUSCLE_CELL_ACTION_POTENTIAL_INVOLVED_IN_CONTRACTION | -2.20 | 0.000 |
| GOBP_REGULATION_OF_PHAGOCYTOSIS | -2.20 | 0.000 |
| GOBP_REGULATION_OF_LEUKOCYTE_CHEMOTAXIS | -2.19 | 0.000 |
| GOBP_GRANULOCYTE_CHEMOTAXIS | -2.18 | 0.000 |
| GOBP_DEVELOPMENTAL_INDUCTION | -2.18 | 0.000 |
| GOMF_COLLAGEN_BINDING | -2.18 | 0.000 |
| GOBP_SMOOTH_MUSCLE_CELL_PROLIFERATION | -2.18 | 0.000 |
| GOBP_ACUTE_INFLAMMATORY_RESPONSE | -2.17 | 0.000 |
| GOBP_NEGATIVE_REGULATION_OF_VASCULAR_ASSOCIATED_SMOOTH_MUSCLE_CELL_PROLIFERATION | -2.17 | 0.000 |
| GOCC_ENDOCYTIC_VESICLE_MEMBRANE | -2.16 | 0.000 |
| GOMF_INTEGRIN_BINDING | -2.16 | 0.000 |
| GOBP_APOPTOTIC_CELL_CLEARANCE | -2.15 | 0.000 |
| GOMF_CHEMOATTRACTANT_ACTIVITY | -2.15 | 0.000 |
| GOMF_PROTEOGLYCAN_BINDING | -2.15 | 0.000 |
| GOBP_REGULATION_OF_CARDIOCYTE_DIFFERENTIATION | -2.15 | 0.000 |
| GOBP_CIRCULATORY_SYSTEM_PROCESS | -2.15 | 0.001 |
| GOBP_HEART_PROCESS | -2.14 | 0.001 |
| GOBP_REGULATION_OF_MONONUCLEAR_CELL_MIGRATION | -2.14 | 0.001 |
| GOBP_CHEMOKINE_PRODUCTION | -2.14 | 0.001 |
| GOBP_POSITIVE_REGULATION_OF_ANIMAL_ORGAN_MORPHOGENESIS | -2.14 | 0.001 |
| GOBP_GRANULOCYTE_MIGRATION | -2.14 | 0.001 |
| GOBP_POSITIVE_CHEMOTAXIS | -2.14 | 0.001 |
| GOBP_REGULATION_OF_ACUTE_INFLAMMATORY_RESPONSE | -2.13 | 0.001 |
| GOBP_REGULATION_OF_PLATELET_AGGREGATION | -2.13 | 0.001 |
| GOBP_ORGAN_INDUCTION | -2.13 | 0.001 |
| GOBP_NEGATIVE_REGULATION_OF_SMOOTH_MUSCLE_CELL_PROLIFERATION | -2.13 | 0.001 |
| GOBP_PHAGOCYTOSIS_ENGULFMENT | -2.12 | 0.001 |
| GOBP_MYELOID_LEUKOCYTE_ACTIVATION | -2.12 | 0.001 |
| GOBP_POSITIVE_REGULATION_OF_ERK1_AND_ERK2_CASCADE | -2.12 | 0.001 |
| GOBP_DIGESTIVE_SYSTEM_DEVELOPMENT | -2.12 | 0.001 |
| GOBP_PLATELET_AGGREGATION | -2.11 | 0.001 |
| GOBP_PLATELET_ACTIVATION | -2.11 | 0.001 |
| GOBP_SPECIFICATION_OF_ANIMAL_ORGAN_IDENTITY | -2.11 | 0.001 |
| GOMF_CARGO_RECEPTOR_ACTIVITY | -2.11 | 0.001 |
| GOBP_POSITIVE_REGULATION_OF_SMOOTH_MUSCLE_CELL_PROLIFERATION | -2.11 | 0.001 |
| GOBP_CELL_RECOGNITION | -2.11 | 0.001 |
| GOMF_SULFUR_COMPOUND_BINDING | -2.11 | 0.001 |
| GOBP_DETECTION_OF_BIOTIC_STIMULUS | -2.11 | 0.001 |
| GOBP_MACROPHAGE_MIGRATION | -2.10 | 0.001 |
| GOBP_CARDIAC_MUSCLE_CELL_ACTION_POTENTIAL | -2.10 | 0.001 |
| GOBP_MESENCHYMAL_CELL_PROLIFERATION | -2.10 | 0.001 |
| GOBP_AORTIC_VALVE_MORPHOGENESIS | -2.10 | 0.001 |
| GOBP_GENITALIA_DEVELOPMENT | -2.10 | 0.001 |
| GOBP_COMPLEMENT_ACTIVATION_CLASSICAL_PATHWAY | -2.10 | 0.001 |
| GOBP_COMPLEMENT_ACTIVATION | -2.10 | 0.001 |
| GOBP_PHAGOCYTOSIS | -2.10 | 0.001 |
| GOBP_CARDIAC_MUSCLE_CELL_CONTRACTION | -2.09 | 0.001 |
| GOBP_NEGATIVE_REGULATION_OF_LOCOMOTION | -2.09 | 0.001 |
| GOMF_EXTRACELLULAR_MATRIX_STRUCTURAL_CONSTITUENT_CONFERRING_TENSILE_STRENGTH | -2.09 | 0.001 |
| GOBP_POSITIVE_REGULATION_OF_STEM_CELL_PROLIFERATION | -2.09 | 0.001 |
| GOBP_SEMI_LUNAR_VALVE_DEVELOPMENT | -2.09 | 0.001 |
| GOBP_POSITIVE_REGULATION_OF_LEUKOCYTE_MIGRATION | -2.09 | 0.001 |
| GOBP_REGULATION_OF_ANIMAL_ORGAN_FORMATION | -2.09 | 0.001 |
| GOBP_CHOLESTEROL_STORAGE | -2.08 | 0.001 |
| GOBP_POSITIVE_REGULATION_OF_POTASSIUM_ION_TRANSPORT | -2.08 | 0.001 |
| GOBP_CARDIOCYTE_DIFFERENTIATION | -2.08 | 0.001 |
| GOBP_HEART_VALVE_MORPHOGENESIS | -2.08 | 0.001 |
| GOBP_HEART_VALVE_DEVELOPMENT | -2.08 | 0.001 |
| GOBP_POSITIVE_REGULATION_OF_VASCULAR_ASSOCIATED_SMOOTH_MUSCLE_CELL_PROLIFERATION | -2.08 | 0.001 |
| GOBP_PHENOL_CONTAINING_COMPOUND_BIOSYNTHETIC_PROCESS | -2.07 | 0.001 |
| GOCC_CLATHRIN_COATED_ENDOCYTIC_VESICLE_MEMBRANE | -2.07 | 0.001 |
| GOBP_CELL_ADHESION_MEDIATED_BY_INTEGRIN | -2.07 | 0.001 |
| GOBP_CARDIAC_MUSCLE_CELL_DIFFERENTIATION | -2.07 | 0.001 |
| GOCC_PROTEIN_COMPLEX_INVOLVED_IN_CELL_MATRIX_ADHESION | -2.07 | 0.001 |
| GOBP_REGULATION_OF_ENDOCYTOSIS | -2.06 | 0.001 |
| GOBP_RESPIRATORY_SYSTEM_DEVELOPMENT | -2.06 | 0.001 |
| GOBP_POSITIVE_REGULATION_OF_LIPID_LOCALIZATION | -2.06 | 0.001 |
| GOBP_CELL_FATE_DETERMINATION | -2.06 | 0.001 |
| GOBP_POSITIVE_REGULATION_OF_LEUKOCYTE_CHEMOTAXIS | -2.06 | 0.001 |
| GOBP_EMBRYONIC_DIGESTIVE_TRACT_DEVELOPMENT | -2.06 | 0.001 |
| GOCC_MAIN_AXON | -2.05 | 0.002 |
| GOBP_CHONDROCYTE_DIFFERENTIATION | -2.05 | 0.002 |
| GOBP_BASEMENT_MEMBRANE_ORGANIZATION | -2.05 | 0.002 |
| GOBP_ADENYLATE_CYCLASE_MODULATING_G_PROTEIN_COUPLED_RECEPTOR_SIGNALING_PATHWAY | -2.05 | 0.002 |
| GOBP_VENTRICULAR_CARDIAC_MUSCLE_CELL_MEMBRANE_REPOLARIZATION | -2.05 | 0.002 |
| GOBP_FOAM_CELL_DIFFERENTIATION | -2.05 | 0.002 |
| GOMF_SCAVENGER_RECEPTOR_ACTIVITY | -2.04 | 0.002 |
| GOBP_POSITIVE_REGULATION_OF_FIBROBLAST_PROLIFERATION | -2.04 | 0.002 |
| GOBP_CARDIAC_VENTRICLE_DEVELOPMENT | -2.04 | 0.002 |
| GOBP_POSITIVE_REGULATION_OF_PHAGOCYTOSIS | -2.04 | 0.002 |
| GOBP_PHENOL_CONTAINING_COMPOUND_METABOLIC_PROCESS | -2.04 | 0.002 |
| GOBP_POSITIVE_REGULATION_OF_MIRNA_TRANSCRIPTION | -2.04 | 0.002 |
| GOBP_POSITIVE_REGULATION_OF_MACROPHAGE_ACTIVATION | -2.04 | 0.002 |
| GOBP_ODONTOGENESIS | -2.04 | 0.002 |
| GOBP_NEUTROPHIL_CHEMOTAXIS | -2.04 | 0.002 |
| GOBP_RESPONSE_TO_XENOBIOTIC_STIMULUS | -2.04 | 0.002 |
| GOBP_MONOCYTE_CHEMOTAXIS | -2.04 | 0.002 |
| GOBP_LUNG_EPITHELIUM_DEVELOPMENT | -2.04 | 0.002 |
| GOBP_POSITIVE_REGULATION_OF_PROTEIN_DEPOLYMERIZATION | -2.04 | 0.002 |
| GOBP_CARDIAC_CONDUCTION | -2.03 | 0.002 |
| GOBP_CELL_MIGRATION_INVOLVED_IN_HEART_DEVELOPMENT | -2.03 | 0.002 |
| GOBP_NEGATIVE_REGULATION_OF_SMOOTH_MUSCLE_CELL_MIGRATION | -2.03 | 0.002 |
| GOBP_STRIATED_MUSCLE_TISSUE_DEVELOPMENT | -2.03 | 0.002 |
| GOBP_REGULATION_OF_COAGULATION | -2.03 | 0.002 |
| GOBP_MEMBRANE_INVAGINATION | -2.03 | 0.002 |
| GOBP_REGULATION_OF_HOMOTYPIC_CELL_CELL_ADHESION | -2.03 | 0.002 |
| GOBP_POSITIVE_REGULATION_OF_ACUTE_INFLAMMATORY_RESPONSE | -2.03 | 0.002 |
| GOBP_ACTIN_MEDIATED_CELL_CONTRACTION | -2.03 | 0.002 |
| GOBP_MUSCLE_CELL_PROLIFERATION | -2.03 | 0.002 |
| GOBP_POSITIVE_REGULATION_OF_CHEMOKINE_PRODUCTION | -2.02 | 0.002 |
| GOBP_REGULATION_OF_ODONTOGENESIS | -2.02 | 0.002 |
| GOBP_EPITHELIAL_CELL_DIFFERENTIATION_INVOLVED_IN_KIDNEY_DEVELOPMENT | -2.02 | 0.002 |
| GOBP_MALE_GENITALIA_DEVELOPMENT | -2.02 | 0.002 |
| GOMF_CYTOKINE_ACTIVITY | -2.02 | 0.002 |
| GOBP_MESENCHYME_MORPHOGENESIS | -2.02 | 0.002 |
| GOBP_BRANCHING_INVOLVED_IN_SALIVARY_GLAND_MORPHOGENESIS | -2.02 | 0.002 |
| GOBP_POSITIVE_REGULATION_OF_EXTRACELLULAR_MATRIX_ORGANIZATION | -2.02 | 0.002 |
| GOBP_REGULATION_OF_MACROPHAGE_MIGRATION | -2.01 | 0.002 |
| GOBP_STRIATED_MUSCLE_CONTRACTION | -2.01 | 0.002 |
| GOCC_PLATELET_ALPHA_GRANULE | -2.01 | 0.002 |
| GOBP_HEMOSTASIS | -2.01 | 0.002 |
| GOCC_PLATELET_ALPHA_GRANULE_LUMEN | -2.01 | 0.002 |
| GOBP_TISSUE_HOMEOSTASIS | -2.01 | 0.002 |
| GOBP_REGULATION_OF_SYSTEM_PROCESS | -2.01 | 0.002 |
| GOBP_REGULATION_OF_CARDIAC_MUSCLE_CELL_DIFFERENTIATION | -2.01 | 0.002 |
| GOBP_ORGANIC_HYDROXY_COMPOUND_BIOSYNTHETIC_PROCESS | -2.00 | 0.002 |
| GOBP_ICOSANOID_BIOSYNTHETIC_PROCESS | -2.00 | 0.003 |
| GOBP_CALCIUM_MEDIATED_SIGNALING | -2.00 | 0.003 |
| GOBP_POSITIVE_REGULATION_OF_INFLAMMATORY_RESPONSE | -2.00 | 0.003 |
| GOBP_POSITIVE_REGULATION_OF_VASCULATURE_DEVELOPMENT | -2.00 | 0.003 |
| GOBP_PHAGOSOME_MATURATION | -2.00 | 0.003 |
| GOCC_EXTERNAL_SIDE_OF_PLASMA_MEMBRANE | -2.00 | 0.003 |
| GOBP_HEART_MORPHOGENESIS | -1.99 | 0.003 |
| GOBP_MUSCLE_CELL_DIFFERENTIATION | -1.99 | 0.003 |
| GOBP_HUMORAL_IMMUNE_RESPONSE_MEDIATED_BY_CIRCULATING_IMMUNOGLOBULIN | -1.99 | 0.003 |
| GOBP_POSITIVE_REGULATION_OF_MIRNA_METABOLIC_PROCESS | -1.99 | 0.003 |
| GOBP_DEFENSE_RESPONSE_TO_GRAM_POSITIVE_BACTERIUM | -1.99 | 0.003 |
| GOBP_TISSUE_MORPHOGENESIS | -1.99 | 0.003 |
| GOBP_NEGATIVE_REGULATION_OF_SECRETION | -1.99 | 0.003 |
| GOCC_PROTEIN_COMPLEX_INVOLVED_IN_CELL_ADHESION | -1.99 | 0.003 |
| GOBP_APPENDAGE_MORPHOGENESIS | -1.99 | 0.003 |
| GOBP_MORPHOGENESIS_OF_AN_EPITHELIUM | -1.99 | 0.003 |
| GOBP_CELL_CELL_SIGNALING_INVOLVED_IN_CARDIAC_CONDUCTION | -1.99 | 0.003 |
| GOBP_CORONARY_VASCULATURE_DEVELOPMENT | -1.99 | 0.003 |
| GOBP_HUMORAL_IMMUNE_RESPONSE | -1.99 | 0.003 |
| GOBP_NEUTROPHIL_HOMEOSTASIS | -1.99 | 0.003 |
| GOBP_REGULATION_OF_HEART_CONTRACTION | -1.98 | 0.003 |
| GOBP_MESENCHYME_DEVELOPMENT | -1.98 | 0.003 |
| GOBP_CELLULAR_RESPONSE_TO_LIPOPROTEIN_PARTICLE_STIMULUS | -1.98 | 0.003 |
| GOBP_NEGATIVE_REGULATION_OF_MONONUCLEAR_CELL_MIGRATION | -1.98 | 0.003 |
| GOBP_CARDIAC_MUSCLE_CELL_MEMBRANE_REPOLARIZATION | -1.98 | 0.003 |
| GOBP_ORGANIC_HYDROXY_COMPOUND_CATABOLIC_PROCESS | -1.98 | 0.003 |
| GOBP_POSITIVE_REGULATION_OF_MAPK_CASCADE | -1.98 | 0.003 |
| GOBP_CARDIAC_MUSCLE_CONTRACTION | -1.98 | 0.003 |
| GOBP_DIGESTIVE_TRACT_MORPHOGENESIS | -1.98 | 0.003 |
| GOBP_POSITIVE_REGULATION_OF_ORGAN_GROWTH | -1.97 | 0.003 |
| GOBP_CELL_COMMUNICATION_INVOLVED_IN_CARDIAC_CONDUCTION | -1.97 | 0.003 |
| GOBP_TISSUE_MIGRATION | -1.97 | 0.003 |
| GOMF_G_PROTEIN_COUPLED_RECEPTOR_BINDING | -1.97 | 0.003 |
| GOBP_CELL_FATE_COMMITMENT_INVOLVED_IN_FORMATION_OF_PRIMARY_GERM_LAYER | -1.97 | 0.003 |
| GOBP_INTERLEUKIN_4_PRODUCTION | -1.97 | 0.003 |
| GOBP_RESPIRATORY_BURST | -1.97 | 0.003 |
| GOBP_COLLAGEN_METABOLIC_PROCESS | -1.97 | 0.003 |
| GOBP_PLASMA_LIPOPROTEIN_PARTICLE_CLEARANCE | -1.97 | 0.003 |
| GOBP_CARDIAC_CHAMBER_MORPHOGENESIS | -1.97 | 0.003 |
| GOBP_CARDIAC_CHAMBER_DEVELOPMENT | -1.97 | 0.003 |
| GOCC_SECONDARY_LYSOSOME | -1.97 | 0.003 |
| GOBP_SEGMENTATION | -1.97 | 0.003 |
| GOCC_FILOPODIUM | -1.97 | 0.003 |
| GOBP_B_CELL_MEDIATED_IMMUNITY | -1.96 | 0.003 |
| GOBP_CONNECTIVE_TISSUE_DEVELOPMENT | -1.96 | 0.003 |
| GOBP_REGULATION_OF_MYELOID_LEUKOCYTE_MEDIATED_IMMUNITY | -1.96 | 0.003 |
| GOBP_PERICARDIUM_DEVELOPMENT | -1.96 | 0.003 |
| GOBP_POSITIVE_REGULATION_OF_MYELOID_LEUKOCYTE_MEDIATED_IMMUNITY | -1.96 | 0.003 |
| GOBP_VASCULAR_PROCESS_IN_CIRCULATORY_SYSTEM | -1.96 | 0.003 |
| GOMF_VOLTAGE_GATED_SODIUM_CHANNEL_ACTIVITY | -1.95 | 0.004 |
| GOBP_POSITIVE_REGULATION_OF_G_PROTEIN_COUPLED_RECEPTOR_SIGNALING_PATHWAY | -1.95 | 0.004 |
| GOCC_BASEMENT_MEMBRANE | -1.95 | 0.004 |
| GOBP_SMOOTH_MUSCLE_CELL_DIFFERENTIATION | -1.95 | 0.004 |
| GOBP_ADRENAL_GLAND_DEVELOPMENT | -1.95 | 0.004 |
| GOBP_AMINE_METABOLIC_PROCESS | -1.95 | 0.004 |
| GOBP_NEUTROPHIL_MIGRATION | -1.94 | 0.004 |
| GOBP_CATECHOL_CONTAINING_COMPOUND_METABOLIC_PROCESS | -1.94 | 0.004 |
| GOBP_KERATINOCYTE_MIGRATION | -1.94 | 0.004 |
| GOBP_MUSCLE_CELL_MIGRATION | -1.94 | 0.004 |
| GOBP_SMOOTH_MUSCLE_CELL_MIGRATION | -1.94 | 0.004 |
| GOBP_REGULATION_OF_POTASSIUM_ION_TRANSPORT | -1.94 | 0.004 |
| GOBP_REGULATION_OF_PROTEIN_DEPOLYMERIZATION | -1.94 | 0.004 |
| GOBP_RESPONSE_TO_WOUNDING | -1.94 | 0.004 |
| GOBP_CELL_DIFFERENTIATION_INVOLVED_IN_KIDNEY_DEVELOPMENT | -1.94 | 0.004 |
| GOBP_RESPONSE_TO_KETONE | -1.94 | 0.004 |
| GOMF_SIGNALING_RECEPTOR_REGULATOR_ACTIVITY | -1.94 | 0.004 |
| GOBP_CARTILAGE_DEVELOPMENT | -1.94 | 0.004 |
| GOMF_OPSONIN_BINDING | -1.94 | 0.004 |
| GOBP_RENAL_SYSTEM_DEVELOPMENT | -1.94 | 0.004 |
| GOBP_EPITHELIAL_TUBE_BRANCHING_INVOLVED_IN_LUNG_MORPHOGENESIS | -1.94 | 0.004 |
| GOBP_MORPHOGENESIS_OF_A_BRANCHING_STRUCTURE | -1.94 | 0.005 |
| GOBP_POSITIVE_REGULATION_OF_CYTOSOLIC_CALCIUM_ION_CONCENTRATION | -1.93 | 0.005 |
| GOBP_OUTFLOW_TRACT_MORPHOGENESIS | -1.93 | 0.005 |
| GOBP_NATURAL_KILLER_CELL_DIFFERENTIATION | -1.93 | 0.005 |
| GOBP_REGULATION_OF_CHOLESTEROL_STORAGE | -1.93 | 0.005 |
| GOBP_PROXIMAL_DISTAL_PATTERN_FORMATION | -1.93 | 0.005 |
| GOBP_MUSCLE_CONTRACTION | -1.93 | 0.005 |
| GOBP_LUNG_MORPHOGENESIS | -1.93 | 0.005 |
| GOBP_HYPERSENSITIVITY | -1.93 | 0.005 |
| GOBP_REGULATION_OF_BLOOD_CIRCULATION | -1.93 | 0.005 |
| GOBP_ROOF_OF_MOUTH_DEVELOPMENT | -1.93 | 0.005 |
| GOBP_PLATELET_DERIVED_GROWTH_FACTOR_RECEPTOR_SIGNALING_PATHWAY | -1.93 | 0.005 |
| GOBP_CELL_FATE_COMMITMENT | -1.93 | 0.005 |
| GOBP_REGULATION_OF_MACROPHAGE_DERIVED_FOAM_CELL_DIFFERENTIATION | -1.93 | 0.005 |
| GOBP_POSITIVE_REGULATION_OF_LIPID_STORAGE | -1.93 | 0.005 |
| GOBP_BIOGENIC_AMINE_METABOLIC_PROCESS | -1.93 | 0.005 |
| GOBP_SODIUM_ION_HOMEOSTASIS | -1.93 | 0.005 |
| GOBP_NEGATIVE_REGULATION_OF_LEUKOCYTE_MIGRATION | -1.92 | 0.005 |
| GOBP_REGULATION_OF_HEART_RATE_BY_CARDIAC_CONDUCTION | -1.92 | 0.005 |
| GOBP_APPENDAGE_DEVELOPMENT | -1.92 | 0.005 |
| GOBP_SOMITOGENESIS | -1.92 | 0.005 |
| GOBP_NEGATIVE_CHEMOTAXIS | -1.92 | 0.005 |
| GOBP_NEGATIVE_REGULATION_OF_EXOCYTOSIS | -1.92 | 0.005 |
| GOBP_ERK1_AND_ERK2_CASCADE | -1.92 | 0.005 |
| GOBP_CELL_ACTIVATION_INVOLVED_IN_IMMUNE_RESPONSE | -1.92 | 0.005 |
| GOBP_RESPONSE_TO_TRANSFORMING_GROWTH_FACTOR_BETA | -1.92 | 0.005 |
| GOCC_ENDOPLASMIC_RETICULUM_PROTEIN_CONTAINING_COMPLEX | -1.91 | 0.005 |
| GOBP_MYELOID_LEUKOCYTE_MEDIATED_IMMUNITY | -1.91 | 0.006 |
| GOBP_ORGANIC_HYDROXY_COMPOUND_METABOLIC_PROCESS | -1.91 | 0.006 |
| GOBP_CHOLESTEROL_EFFLUX | -1.91 | 0.006 |
| GOBP_ENDOCARDIAL_CUSHION_DEVELOPMENT | -1.91 | 0.006 |
| GOBP_UNSATURATED_FATTY_ACID_BIOSYNTHETIC_PROCESS | -1.91 | 0.006 |
| GOBP_REGULATION_OF_VENTRICULAR_CARDIAC_MUSCLE_CELL_MEMBRANE_REPOLARIZATION | -1.91 | 0.006 |
| GOMF_STRUCTURAL_CONSTITUENT_OF_RIBOSOME | -1.91 | 0.006 |
| GOBP_PITUITARY_GLAND_DEVELOPMENT | -1.91 | 0.006 |
| GOBP_POSITIVE_REGULATION_OF_NEUROBLAST_PROLIFERATION | -1.91 | 0.006 |
| GOBP_AMEBOIDAL_TYPE_CELL_MIGRATION | -1.91 | 0.006 |
| GOBP_HOMOTYPIC_CELL_CELL_ADHESION | -1.90 | 0.006 |
| GOBP_HOMOPHILIC_CELL_ADHESION_VIA_PLASMA_MEMBRANE_ADHESION_MOLECULES | -1.90 | 0.006 |
| GOBP_NEGATIVE_REGULATION_OF_REGULATED_SECRETORY_PATHWAY | -1.90 | 0.006 |
| GOBP_NEGATIVE_REGULATION_OF_COAGULATION | -1.90 | 0.006 |
| GOBP_REGULATION_OF_CYTOSOLIC_CALCIUM_ION_CONCENTRATION | -1.90 | 0.006 |
| GOBP_REGULATION_OF_LIPID_LOCALIZATION | -1.90 | 0.006 |
| GOBP_PULMONARY_VALVE_MORPHOGENESIS | -1.90 | 0.006 |
| GOBP_REGULATION_OF_VESICLE_MEDIATED_TRANSPORT | -1.90 | 0.006 |
| GOBP_SECONDARY_METABOLIC_PROCESS | -1.90 | 0.006 |
| GOBP_MUSCLE_TISSUE_DEVELOPMENT | -1.90 | 0.006 |
| GOBP_MUSCLE_SYSTEM_PROCESS | -1.90 | 0.006 |
| GOBP_POSITIVE_REGULATION_OF_EPITHELIAL_CELL_MIGRATION | -1.90 | 0.006 |
| GOBP_POSITIVE_REGULATION_OF_POTASSIUM_ION_TRANSMEMBRANE_TRANSPORT | -1.90 | 0.006 |
| GOBP_REGULATION_OF_CELL_ACTIVATION | -1.90 | 0.006 |
| GOBP_REGULATION_OF_CARDIAC_MUSCLE_CELL_MEMBRANE_REPOLARIZATION | -1.90 | 0.006 |
| GOBP_EXTRACELLULAR_MATRIX_DISASSEMBLY | -1.89 | 0.007 |
| GOCC_ENDOCYTIC_VESICLE | -1.89 | 0.007 |
| GOCC_BLOOD_MICROPARTICLE | -1.89 | 0.007 |
| GOBP_POSITIVE_REGULATION_OF_VIRAL_LIFE_CYCLE | -1.89 | 0.007 |
| GOBP_CELL_SUBSTRATE_ADHESION | -1.89 | 0.007 |
| GOMF_TRANSMEMBRANE_TRANSPORTER_BINDING | -1.89 | 0.007 |
| GOBP_AMYLOID_BETA_CLEARANCE | -1.89 | 0.007 |
| GOBP_HETEROTYPIC_CELL_CELL_ADHESION | -1.89 | 0.007 |
| GOBP_REGULATION_OF_LIPID_TRANSPORT | -1.89 | 0.007 |
| GOBP_POSITIVE_REGULATION_OF_RESPONSE_TO_CYTOKINE_STIMULUS | -1.89 | 0.007 |
| GOBP_FEMALE_GENITALIA_DEVELOPMENT | -1.89 | 0.007 |
| GOBP_ENDOCRINE_SYSTEM_DEVELOPMENT | -1.88 | 0.007 |
| GOBP_ADENYLATE_CYCLASE_ACTIVATING_G_PROTEIN_COUPLED_RECEPTOR_SIGNALING_PATHWAY | -1.88 | 0.007 |
| GOBP_POSITIVE_REGULATION_OF_CYTOKINE_PRODUCTION | -1.88 | 0.007 |
| GOBP_PROTEIN_LIPID_COMPLEX_ORGANIZATION | -1.88 | 0.007 |
| GOBP_RESPONSE_TO_PURINE_CONTAINING_COMPOUND | -1.88 | 0.007 |
| GOBP_NEUROINFLAMMATORY_RESPONSE | -1.88 | 0.008 |
| GOBP_LYMPH_VESSEL_DEVELOPMENT | -1.88 | 0.008 |
| GOBP_PROTEIN_CONTAINING_COMPLEX_REMODELING | -1.88 | 0.008 |
| GOCC_SPECIFIC_GRANULE_MEMBRANE | -1.88 | 0.008 |
| GOBP_REGULATION_OF_GRANULOCYTE_CHEMOTAXIS | -1.88 | 0.008 |
| GOBP_RESPONSE_TO_ALCOHOL | -1.88 | 0.008 |
| GOBP_REGULATION_OF_MONOCYTE_CHEMOTAXIS | -1.88 | 0.008 |
| GOBP_RESPONSE_TO_BACTERIUM | -1.88 | 0.008 |
| GOBP_STEROID_METABOLIC_PROCESS | -1.88 | 0.008 |
| GOBP_REGULATION_OF_WOUND_HEALING | -1.88 | 0.008 |
| GOBP_ENDOTHELIAL_CELL_MIGRATION | -1.88 | 0.008 |
| GOBP_SALIVARY_GLAND_DEVELOPMENT | -1.88 | 0.008 |
| GOBP_POSITIVE_REGULATION_OF_MONONUCLEAR_CELL_MIGRATION | -1.87 | 0.008 |
| GOBP_WOUND_HEALING | -1.87 | 0.008 |
| GOBP_POSITIVE_REGULATION_OF_HEMOPOIESIS | -1.87 | 0.008 |
| GOBP_POSITIVE_REGULATION_OF_INTERLEUKIN_4_PRODUCTION | -1.87 | 0.008 |
| GOBP_CELLULAR_RESPONSE_TO_VASCULAR_ENDOTHELIAL_GROWTH_FACTOR_STIMULUS | -1.87 | 0.008 |
| GOBP_POSITIVE_REGULATION_OF_T_CELL_PROLIFERATION | -1.87 | 0.008 |
| GOBP_ADAPTIVE_IMMUNE_RESPONSE | -1.87 | 0.008 |
| GOBP_REGULATION_OF_MEMBRANE_REPOLARIZATION | -1.87 | 0.008 |
| GOBP_COLLAGEN_BIOSYNTHETIC_PROCESS | -1.87 | 0.008 |
| GOBP_REGULATION_OF_MACROPHAGE_ACTIVATION | -1.87 | 0.008 |
| GOBP_INFLAMMASOME_MEDIATED_SIGNALING_PATHWAY | -1.87 | 0.009 |
| GOBP_CELL_CELL_ADHESION_VIA_PLASMA_MEMBRANE_ADHESION_MOLECULES | -1.87 | 0.009 |
| GOBP_ACUTE_INFLAMMATORY_RESPONSE_TO_ANTIGENIC_STIMULUS | -1.87 | 0.009 |
| GOCC_MEMBRANE_MICRODOMAIN | -1.87 | 0.009 |
| GOBP_STRIATED_MUSCLE_CELL_DIFFERENTIATION | -1.87 | 0.009 |
| GOBP_LEUKOCYTE_ACTIVATION_INVOLVED_IN_INFLAMMATORY_RESPONSE | -1.87 | 0.009 |
| GOBP_CD4_POSITIVE_ALPHA_BETA_T_CELL_PROLIFERATION | -1.86 | 0.009 |
| GOBP_CELLULAR_RESPONSE_TO_XENOBIOTIC_STIMULUS | -1.86 | 0.009 |
| GOBP_MONOATOMIC_ION_HOMEOSTASIS | -1.86 | 0.009 |
| GOMF_PATTERN_RECOGNITION_RECEPTOR_ACTIVITY | -1.86 | 0.009 |
| GOBP_HEART_FORMATION | -1.86 | 0.009 |
| GOBP_MYELOID_CELL_ACTIVATION_INVOLVED_IN_IMMUNE_RESPONSE | -1.86 | 0.009 |
| GOBP_LYMPHOCYTE_ACTIVATION_INVOLVED_IN_IMMUNE_RESPONSE | -1.86 | 0.009 |
| GOBP_ORGANIC_HYDROXY_COMPOUND_TRANSPORT | -1.86 | 0.009 |
| GOBP_REGULATION_OF_STEM_CELL_PROLIFERATION | -1.86 | 0.009 |
| GOBP_POSITIVE_REGULATION_OF_OSTEOBLAST_DIFFERENTIATION | -1.86 | 0.009 |
| GOBP_AXIS_ELONGATION | -1.86 | 0.010 |
| GOBP_REGULATION_OF_FIBROBLAST_PROLIFERATION | -1.86 | 0.010 |
| GOBP_REGULATION_OF_MORPHOGENESIS_OF_AN_EPITHELIUM | -1.86 | 0.010 |
| GOBP_GLUCOCORTICOID_METABOLIC_PROCESS | -1.86 | 0.010 |
| GOBP_POSITIVE_REGULATION_OF_ENDOTHELIAL_CELL_PROLIFERATION | -1.86 | 0.010 |
| GOBP_SKELETAL_SYSTEM_DEVELOPMENT | -1.85 | 0.010 |
| GOBP_MONOAMINE_TRANSPORT | -1.85 | 0.010 |
| GOBP_PULMONARY_VALVE_DEVELOPMENT | -1.85 | 0.010 |
| GOBP_CELLULAR_ALDEHYDE_METABOLIC_PROCESS | -1.85 | 0.010 |
| GOBP_RESPONSE_TO_GONADOTROPIN | -1.85 | 0.010 |
| GOBP_GLIAL_CELL_ACTIVATION | -1.85 | 0.010 |
| GOBP_NEGATIVE_REGULATION_OF_CELL_KILLING | -1.85 | 0.010 |
| GOBP_MESENCHYMAL_CELL_DIFFERENTIATION | -1.85 | 0.010 |
| GOBP_REGULATION_OF_POSITIVE_CHEMOTAXIS | -1.85 | 0.010 |
| GOBP_PHAGOLYSOSOME_ASSEMBLY | -1.85 | 0.010 |
| GOBP_ACTION_POTENTIAL | -1.85 | 0.010 |
| GOBP_VASODILATION | -1.85 | 0.010 |
| GOBP_NEGATIVE_REGULATION_OF_PLATELET_ACTIVATION | -1.85 | 0.010 |
| GOBP_REGULATION_OF_BODY_FLUID_LEVELS | -1.85 | 0.010 |
| GOBP_POSITIVE_REGULATION_OF_LEUKOCYTE_PROLIFERATION | -1.85 | 0.010 |
| GOBP_POSITIVE_REGULATION_OF_LIPID_TRANSPORT | -1.85 | 0.010 |
| GOBP_MONOATOMIC_ANION_HOMEOSTASIS | -1.85 | 0.010 |
| GOBP_CELLULAR_RESPONSE_TO_CAMP | -1.85 | 0.010 |
| GOMF_SERINE_HYDROLASE_ACTIVITY | -1.85 | 0.010 |
| GOBP_NEGATIVE_REGULATION_OF_CHEMOTAXIS | -1.85 | 0.010 |
| GOBP_REGULATION_OF_PLASMA_LIPOPROTEIN_PARTICLE_LEVELS | -1.85 | 0.011 |
| GOBP_MYOBLAST_DIFFERENTIATION | -1.85 | 0.011 |
| GOBP_LEUKOCYTE_HOMEOSTASIS | -1.84 | 0.011 |
| GOBP_POSITIVE_REGULATION_OF_CELL_ACTIVATION | -1.84 | 0.011 |
| GOCC_RIBOSOMAL_SUBUNIT | -1.84 | 0.011 |
| GOBP_GLUCOSAMINE_CONTAINING_COMPOUND_METABOLIC_PROCESS | -1.84 | 0.011 |
| GOBP_CELLULAR_RESPONSE_TO_ACID_CHEMICAL | -1.84 | 0.011 |
| GOMF_3_5_CYCLIC_AMP_PHOSPHODIESTERASE_ACTIVITY | -1.84 | 0.011 |
| GOBP_NEGATIVE_REGULATION_OF_TRANSPORT | -1.84 | 0.011 |
| GOBP_HETEROPHILIC_CELL_CELL_ADHESION_VIA_PLASMA_MEMBRANE_CELL_ADHESION_MOLECULES | -1.84 | 0.011 |
| GOBP_REGULATION_OF_MUSCLE_CONTRACTION | -1.84 | 0.011 |
| GOBP_REGULATION_OF_BLOOD_PRESSURE | -1.84 | 0.011 |
| GOBP_MORPHOGENESIS_OF_AN_EPITHELIAL_SHEET | -1.84 | 0.011 |
| GOBP_OSTEOBLAST_DIFFERENTIATION | -1.84 | 0.011 |
| GOBP_AMINO_SUGAR_METABOLIC_PROCESS | -1.84 | 0.011 |
| GOCC_TERTIARY_GRANULE | -1.84 | 0.011 |
| GOMF_SULFOTRANSFERASE_ACTIVITY | -1.84 | 0.011 |
| GOMF_OXIDOREDUCTASE_ACTIVITY_ACTING_ON_THE_CH_NH2_GROUP_OF_DONORS_OXYGEN_AS_ACCEPTOR | -1.84 | 0.011 |
| GOBP_REGULATION_OF_MYOBLAST_DIFFERENTIATION | -1.84 | 0.011 |
| GOBP_ENDOTHELIAL_CELL_APOPTOTIC_PROCESS | -1.84 | 0.011 |
| GOBP_ACTIN_FILAMENT_BASED_MOVEMENT | -1.84 | 0.011 |
| GOBP_EPITHELIAL_TUBE_MORPHOGENESIS | -1.84 | 0.011 |
| GOBP_REGULATION_OF_CHONDROCYTE_DIFFERENTIATION | -1.83 | 0.011 |
| GOCC_STRIATED_MUSCLE_THIN_FILAMENT | -1.83 | 0.012 |
| GOBP_REGULATION_OF_HEART_RATE | -1.83 | 0.012 |
| GOBP_ANIMAL_ORGAN_FORMATION | -1.83 | 0.012 |
| GOBP_MACROPHAGE_CHEMOTAXIS | -1.83 | 0.012 |
| GOBP_FIBROBLAST_PROLIFERATION | -1.83 | 0.012 |
| GOMF_LIPOPOLYSACCHARIDE_BINDING | -1.83 | 0.012 |
| GOBP_FORMATION_OF_PRIMARY_GERM_LAYER | -1.83 | 0.012 |
| GOBP_RESPONSE_TO_NUTRIENT | -1.83 | 0.012 |
| GOBP_ADENYLATE_CYCLASE_INHIBITING_G_PROTEIN_COUPLED_RECEPTOR_SIGNALING_PATHWAY | -1.83 | 0.012 |
| GOBP_MORPHOGENESIS_OF_AN_EPITHELIAL_FOLD | -1.83 | 0.012 |
| GOBP_LEUKOCYTE_DEGRANULATION | -1.83 | 0.012 |
| GOBP_METANEPHROS_DEVELOPMENT | -1.83 | 0.012 |
| GOBP_ACTIVATION_OF_PHOSPHOLIPASE_C_ACTIVITY | -1.83 | 0.012 |
| GOBP_PROTEIN_LIPID_COMPLEX_ASSEMBLY | -1.83 | 0.012 |
| GOBP_REGULATION_OF_INSULIN_LIKE_GROWTH_FACTOR_RECEPTOR_SIGNALING_PATHWAY | -1.83 | 0.012 |
| GOBP_POSITIVE_REGULATION_OF_STEM_CELL_DIFFERENTIATION | -1.83 | 0.012 |
| GOBP_MEMBRANE_REPOLARIZATION | -1.83 | 0.012 |
| GOBP_DETECTION_OF_EXTERNAL_BIOTIC_STIMULUS | -1.83 | 0.012 |
| GOBP_ANTERIOR_POSTERIOR_PATTERN_SPECIFICATION | -1.83 | 0.012 |
| GOBP_POSITIVE_REGULATION_OF_NOTCH_SIGNALING_PATHWAY | -1.83 | 0.012 |
| GOCC_SARCOLEMMA | -1.83 | 0.012 |
| GOBP_POSITIVE_REGULATION_OF_PROTEIN_CONTAINING_COMPLEX_DISASSEMBLY | -1.82 | 0.012 |
| GOMF_GROWTH_FACTOR_RECEPTOR_BINDING | -1.82 | 0.012 |
| GOBP_POSITIVE_REGULATION_OF_HEART_GROWTH | -1.82 | 0.012 |
| GOBP_N_ACETYLGLUCOSAMINE_METABOLIC_PROCESS | -1.82 | 0.012 |
| GOBP_UTERUS_DEVELOPMENT | -1.82 | 0.012 |
| GOBP_OSSIFICATION | -1.82 | 0.012 |
| GOBP_VENTRICULAR_CARDIAC_MUSCLE_CELL_ACTION_POTENTIAL | -1.82 | 0.012 |
| GOBP_NEUTRAL_LIPID_CATABOLIC_PROCESS | -1.82 | 0.013 |
| GOBP_KIDNEY_EPITHELIUM_DEVELOPMENT | -1.82 | 0.013 |
| GOBP_ANTIGEN_PROCESSING_AND_PRESENTATION | -1.82 | 0.012 |
| GOBP_REGULATION_OF_SYSTEMIC_ARTERIAL_BLOOD_PRESSURE_BY_CIRCULATORY_RENIN_ANGIOTENSIN | -1.82 | 0.013 |
| GOBP_PATTERN_SPECIFICATION_PROCESS | -1.82 | 0.013 |
| GOBP_POSITIVE_REGULATION_OF_CHOLESTEROL_EFFLUX | -1.82 | 0.013 |
| GOCC_PLASMA_MEMBRANE_RAFT | -1.82 | 0.013 |
| GOMF_FRIZZLED_BINDING | -1.82 | 0.013 |
| GOBP_REGULATION_OF_POTASSIUM_ION_TRANSMEMBRANE_TRANSPORTER_ACTIVITY | -1.82 | 0.013 |
| GOBP_POSITIVE_REGULATION_OF_CELL_ADHESION | -1.82 | 0.013 |
| GOBP_BRANCHING_MORPHOGENESIS_OF_AN_EPITHELIAL_TUBE | -1.81 | 0.013 |
| GOBP_EMBRYONIC_APPENDAGE_MORPHOGENESIS | -1.81 | 0.013 |
| GOBP_HINDLIMB_MORPHOGENESIS | -1.81 | 0.013 |
| GOBP_INORGANIC_ION_HOMEOSTASIS | -1.81 | 0.013 |
| GOBP_REGULATION_OF_MIRNA_TRANSCRIPTION | -1.81 | 0.013 |
| GOBP_LEUKOCYTE_MEDIATED_IMMUNITY | -1.81 | 0.013 |
| GOBP_REGULATION_OF_MUSCLE_CELL_DIFFERENTIATION | -1.81 | 0.013 |
| GOBP_POSITIVE_REGULATION_OF_MYELOID_LEUKOCYTE_DIFFERENTIATION | -1.81 | 0.013 |
| GOBP_REGULATION_OF_CELL_CELL_ADHESION | -1.81 | 0.014 |
| GOBP_REGULATION_OF_MIRNA_METABOLIC_PROCESS | -1.81 | 0.014 |
| GOBP_POSITIVE_REGULATION_OF_BONE_RESORPTION | -1.81 | 0.014 |
| GOBP_NEGATIVE_REGULATION_OF_WOUND_HEALING | -1.81 | 0.014 |
| GOBP_IMMUNE_EFFECTOR_PROCESS | -1.81 | 0.014 |
| GOBP_NEGATIVE_REGULATION_OF_ENDOTHELIAL_CELL_APOPTOTIC_PROCESS | -1.81 | 0.014 |
| GOBP_POSITIVE_REGULATION_OF_COAGULATION | -1.81 | 0.014 |
| GOBP_POSITIVE_REGULATION_OF_MESENCHYMAL_CELL_PROLIFERATION | -1.81 | 0.014 |
| GOBP_RESPONSE_TO_ORGANOPHOSPHORUS | -1.81 | 0.014 |
| GOBP_REGULATION_OF_RENAL_SYSTEM_PROCESS | -1.81 | 0.014 |
| GOBP_NEGATIVE_REGULATION_OF_EXTRINSIC_APOPTOTIC_SIGNALING_PATHWAY | -1.81 | 0.014 |
| GOCC_IMMUNOLOGICAL_SYNAPSE | -1.81 | 0.014 |
| GOBP_REGULATION_OF_STRIATED_MUSCLE_CONTRACTION | -1.81 | 0.014 |
| GOBP_REGULATION_OF_SUPEROXIDE_METABOLIC_PROCESS | -1.81 | 0.014 |
| GOBP_GLAND_DEVELOPMENT | -1.81 | 0.014 |
| GOBP_REGULATION_OF_VASCULATURE_DEVELOPMENT | -1.81 | 0.014 |
| GOBP_CARDIAC_CELL_DEVELOPMENT | -1.80 | 0.014 |
| GOBP_INFLAMMATORY_RESPONSE_TO_ANTIGENIC_STIMULUS | -1.80 | 0.014 |
| GOBP_MAMMARY_GLAND_ALVEOLUS_DEVELOPMENT | -1.80 | 0.014 |
| GOMF_IMMUNE_RECEPTOR_ACTIVITY | -1.80 | 0.014 |
| GOBP_PHYSIOLOGICAL_CARDIAC_MUSCLE_HYPERTROPHY | -1.80 | 0.014 |
| GOMF_INSULIN_LIKE_GROWTH_FACTOR_BINDING | -1.80 | 0.014 |
| GOMF_INTRAMOLECULAR_OXIDOREDUCTASE_ACTIVITY | -1.80 | 0.014 |
| GOBP_REGULATION_OF_MUSCLE_SYSTEM_PROCESS | -1.80 | 0.014 |
| GOBP_TRIGLYCERIDE_CATABOLIC_PROCESS | -1.80 | 0.015 |
| GOBP_REGULATION_OF_FATTY_ACID_TRANSPORT | -1.80 | 0.015 |
| GOBP_NEGATIVE_REGULATION_OF_EPITHELIAL_CELL_APOPTOTIC_PROCESS | -1.80 | 0.015 |
| GOBP_GLOMERULUS_DEVELOPMENT | -1.80 | 0.015 |
| GOBP_ADAPTIVE_IMMUNE_RESPONSE_BASED_ON_SOMATIC_RECOMBINATION_OF_IMMUNE_RECEPTORS_BUILT_FROM_IMMUNOGLOBULIN_SUPERFAMILY_DOMAINS | -1.80 | 0.015 |
| GOCC_COMPLEX_OF_COLLAGEN_TRIMERS | -1.80 | 0.015 |
| GOMF_GROWTH_FACTOR_ACTIVITY | -1.80 | 0.015 |
| GOBP_POSITIVE_REGULATION_OF_CELL_CELL_ADHESION | -1.80 | 0.015 |
| GOBP_REGULATION_OF_HEART_GROWTH | -1.80 | 0.015 |
| GOBP_POSITIVE_REGULATION_OF_CARDIAC_MUSCLE_CELL_PROLIFERATION | -1.80 | 0.015 |
| GOBP_ODONTOGENESIS_OF_DENTIN_CONTAINING_TOOTH | -1.79 | 0.015 |
| GOMF_EXOGENOUS_PROTEIN_BINDING | -1.79 | 0.015 |
| GOBP_NEURON_PROJECTION_EXTENSION_INVOLVED_IN_NEURON_PROJECTION_GUIDANCE | -1.79 | 0.015 |
| GOBP_REGULATION_OF_SYSTEMIC_ARTERIAL_BLOOD_PRESSURE_MEDIATED_BY_A_CHEMICAL_SIGNAL | -1.79 | 0.015 |
| GOBP_NEGATIVE_REGULATION_OF_MUSCLE_CELL_DIFFERENTIATION | -1.79 | 0.015 |
| GOBP_ALCOHOL_CATABOLIC_PROCESS | -1.79 | 0.015 |
| GOBP_RENAL_SYSTEM_VASCULATURE_DEVELOPMENT | -1.79 | 0.016 |
| GOCC_CLATHRIN_COATED_ENDOCYTIC_VESICLE | -1.79 | 0.016 |
| GOBP_VASCULAR_ENDOTHELIAL_GROWTH_FACTOR_PRODUCTION | -1.79 | 0.016 |
| GOBP_NEGATIVE_REGULATION_OF_NUCLEOCYTOPLASMIC_TRANSPORT | -1.79 | 0.016 |
| GOBP_MUSCLE_CELL_DEVELOPMENT | -1.79 | 0.016 |
| GOCC_PLASMA_MEMBRANE_PROTEIN_COMPLEX | -1.79 | 0.016 |
| GOBP_EPIBOLY | -1.79 | 0.016 |
| GOBP_RESPONSE_TO_HEPATOCYTE_GROWTH_FACTOR | -1.79 | 0.016 |
| GOBP_POSITIVE_REGULATION_OF_RECEPTOR_INTERNALIZATION | -1.79 | 0.016 |
| GOBP_TRIGLYCERIDE_METABOLIC_PROCESS | -1.79 | 0.016 |
| GOBP_LENS_DEVELOPMENT_IN_CAMERA_TYPE_EYE | -1.79 | 0.016 |
| GOBP_MOLTING_CYCLE_PROCESS | -1.79 | 0.016 |
| GOBP_ESTABLISHMENT_OR_MAINTENANCE_OF_MONOPOLAR_CELL_POLARITY | -1.78 | 0.016 |
| GOBP_MULTICELLULAR_ORGANISMAL_RESPONSE_TO_STRESS | -1.78 | 0.016 |
| GOBP_RESPONSE_TO_MOLECULE_OF_BACTERIAL_ORIGIN | -1.78 | 0.016 |
| GOBP_INTERLEUKIN_2_PRODUCTION | -1.78 | 0.016 |
| GOMF_G_PROTEIN_ALPHA_SUBUNIT_BINDING | -1.78 | 0.016 |
| GOBP_UNSATURATED_FATTY_ACID_METABOLIC_PROCESS | -1.78 | 0.016 |
| GOBP_DENDRITIC_CELL_DIFFERENTIATION | -1.78 | 0.016 |
| GOBP_CATECHOLAMINE_TRANSPORT | -1.78 | 0.016 |
| GOBP_T_CELL_ACTIVATION | -1.78 | 0.016 |
| GOBP_NEGATIVE_REGULATION_OF_IMMUNE_EFFECTOR_PROCESS | -1.78 | 0.016 |
| GOBP_GLIAL_CELL_APOPTOTIC_PROCESS | -1.78 | 0.016 |
| GOBP_ENDOCRINE_PROCESS | -1.78 | 0.017 |
| GOBP_CELL_KILLING | -1.78 | 0.016 |
| GOBP_ENDODERM_DEVELOPMENT | -1.78 | 0.017 |
| GOBP_POSITIVE_REGULATION_OF_RECEPTOR_MEDIATED_ENDOCYTOSIS | -1.78 | 0.017 |
| GOBP_ICOSANOID_METABOLIC_PROCESS | -1.78 | 0.017 |
| GOBP_RECEPTOR_MEDIATED_ENDOCYTOSIS | -1.78 | 0.017 |
| GOBP_SECONDARY_METABOLITE_BIOSYNTHETIC_PROCESS | -1.78 | 0.017 |
| GOBP_GAMMA_AMINOBUTYRIC_ACID_TRANSPORT | -1.78 | 0.017 |
| GOBP_ARTERY_MORPHOGENESIS | -1.78 | 0.017 |
| GOBP_POSITIVE_REGULATION_OF_MONOATOMIC_ION_TRANSMEMBRANE_TRANSPORT | -1.78 | 0.017 |
| GOBP_ORGANIC_ACID_BIOSYNTHETIC_PROCESS | -1.78 | 0.017 |
| GOBP_ALCOHOL_METABOLIC_PROCESS | -1.78 | 0.017 |
| GOBP_MAMMARY_GLAND_DEVELOPMENT | -1.78 | 0.017 |
| GOBP_REGULATION_OF_EPITHELIAL_CELL_MIGRATION | -1.78 | 0.017 |
| GOBP_VERY_LONG_CHAIN_FATTY_ACID_METABOLIC_PROCESS | -1.78 | 0.017 |
| GOBP_REGULATION_OF_LEUKOCYTE_PROLIFERATION | -1.78 | 0.017 |
| GOBP_PHOSPHOLIPASE_C_ACTIVATING_G_PROTEIN_COUPLED_RECEPTOR_SIGNALING_PATHWAY | -1.78 | 0.017 |
| GOBP_ANTIGEN_PROCESSING_AND_PRESENTATION_OF_PEPTIDE_ANTIGEN | -1.78 | 0.017 |
| GOBP_CELL_DIFFERENTIATION_INVOLVED_IN_METANEPHROS_DEVELOPMENT | -1.77 | 0.017 |
| GOBP_POSITIVE_REGULATION_OF_PHOSPHATASE_ACTIVITY | -1.77 | 0.017 |
| GOCC_RIBOSOME | -1.77 | 0.017 |
| GOBP_CARDIAC_VENTRICLE_MORPHOGENESIS | -1.77 | 0.017 |
| GOBP_STEROL_TRANSPORT | -1.77 | 0.017 |
| GOBP_NEGATIVE_REGULATION_OF_CELL_JUNCTION_ASSEMBLY | -1.77 | 0.017 |
| GOCC_VESICLE_LUMEN | -1.77 | 0.017 |
| GOBP_BONE_RESORPTION | -1.77 | 0.017 |
| GOMF_CYTOKINE_BINDING | -1.77 | 0.017 |
| GOBP_REGULATION_OF_INFLAMMATORY_RESPONSE | -1.77 | 0.017 |
| GOBP_NEGATIVE_REGULATION_OF_FAT_CELL_DIFFERENTIATION | -1.77 | 0.017 |
| GOBP_POSITIVE_REGULATION_OF_NEURON_PROJECTION_DEVELOPMENT | -1.77 | 0.017 |
| GOBP_REGULATION_OF_BONE_RESORPTION | -1.77 | 0.017 |
| GOBP_MONONUCLEAR_CELL_DIFFERENTIATION | -1.77 | 0.017 |
| GOBP_STRIATED_MUSCLE_CELL_DEVELOPMENT | -1.77 | 0.018 |
| GOBP_ENDODERM_FORMATION | -1.77 | 0.018 |
| GOBP_NEURON_INTRINSIC_APOPTOTIC_SIGNALING_PATHWAY_IN_RESPONSE_TO_OXIDATIVE_STRESS | -1.77 | 0.018 |
| GOBP_CELL_CELL_RECOGNITION | -1.77 | 0.018 |
| GOBP_NEURON_RECOGNITION | -1.77 | 0.018 |
| GOBP_POSITIVE_REGULATION_OF_B_CELL_MEDIATED_IMMUNITY | -1.77 | 0.018 |
| GOBP_POSITIVE_REGULATION_OF_SYNCYTIUM_FORMATION_BY_PLASMA_MEMBRANE_FUSION | -1.77 | 0.018 |
| GOBP_REGULATION_OF_B_CELL_ACTIVATION | -1.77 | 0.018 |
| GOCC_PHAGOCYTIC_VESICLE | -1.77 | 0.018 |
| GOBP_MONOCARBOXYLIC_ACID_BIOSYNTHETIC_PROCESS | -1.77 | 0.018 |
| GOBP_OLEFINIC_COMPOUND_BIOSYNTHETIC_PROCESS | -1.77 | 0.018 |
| GOBP_REGULATION_OF_MACROPHAGE_CHEMOTAXIS | -1.77 | 0.018 |
| GOBP_REGULATION_OF_OSTEOBLAST_DIFFERENTIATION | -1.77 | 0.018 |
| GOBP_PROSTATE_GLAND_DEVELOPMENT | -1.76 | 0.018 |
| GOBP_RESPONSE_TO_AXON_INJURY | -1.76 | 0.018 |
| GOMF_OXIDOREDUCTASE_ACTIVITY_ACTING_ON_THE_CH_NH2_GROUP_OF_DONORS | -1.76 | 0.018 |
| GOBP_VASCULAR_TRANSPORT | -1.76 | 0.018 |
| GOBP_DOPAMINE_METABOLIC_PROCESS | -1.76 | 0.018 |
| GOMF_AMIDE_BINDING | -1.76 | 0.018 |
| GOCC_PROTEIN_LIPID_COMPLEX | -1.76 | 0.018 |
| GOBP_POSITIVE_REGULATION_OF_MONOATOMIC_ION_TRANSPORT | -1.76 | 0.018 |
| GOBP_TUMOR_NECROSIS_FACTOR_SUPERFAMILY_CYTOKINE_PRODUCTION | -1.76 | 0.018 |
| GOBP_MYOBLAST_FUSION | -1.76 | 0.018 |
| GOBP_CARDIAC_SEPTUM_MORPHOGENESIS | -1.76 | 0.018 |
| GOBP_APOPTOTIC_PROCESS_INVOLVED_IN_DEVELOPMENT | -1.76 | 0.018 |
| GOMF_CHEMOKINE_BINDING | -1.76 | 0.018 |
| GOBP_PYRIDINE_NUCLEOTIDE_BIOSYNTHETIC_PROCESS | -1.76 | 0.019 |
| GOBP_EXOCRINE_SYSTEM_DEVELOPMENT | -1.76 | 0.019 |
| GOBP_LEUKOCYTE_PROLIFERATION | -1.76 | 0.019 |
| GOBP_RESPONSE_TO_PEPTIDE | -1.76 | 0.019 |
| GOBP_REGULATION_OF_MESENCHYMAL_CELL_PROLIFERATION | -1.76 | 0.019 |
| GOBP_OLEFINIC_COMPOUND_METABOLIC_PROCESS | -1.76 | 0.019 |
| GOBP_REGULATION_OF_G_PROTEIN_COUPLED_RECEPTOR_SIGNALING_PATHWAY | -1.76 | 0.019 |
| GOBP_REGULATION_OF_LEUKOCYTE_DIFFERENTIATION | -1.76 | 0.019 |
| GOBP_POLYSACCHARIDE_BIOSYNTHETIC_PROCESS | -1.76 | 0.019 |
| GOBP_SYNCYTIUM_FORMATION | -1.75 | 0.019 |
| GOBP_POSITIVE_REGULATION_OF_NEURAL_PRECURSOR_CELL_PROLIFERATION | -1.75 | 0.019 |
| GOBP_REGULATION_OF_NITRIC_OXIDE_METABOLIC_PROCESS | -1.75 | 0.019 |
| GOBP_CRANIAL_SKELETAL_SYSTEM_DEVELOPMENT | -1.75 | 0.020 |
| GOBP_GLOMERULAR_EPITHELIUM_DEVELOPMENT | -1.75 | 0.020 |
| GOBP_T_CELL_PROLIFERATION | -1.75 | 0.020 |
| GOBP_REGENERATION | -1.75 | 0.020 |
| GOBP_RESPONSE_TO_TOXIC_SUBSTANCE | -1.75 | 0.020 |
| GOBP_REGULATION_OF_T_CELL_PROLIFERATION | -1.75 | 0.020 |
| GOBP_INTRACELLULAR_SODIUM_ION_HOMEOSTASIS | -1.75 | 0.020 |
| GOBP_STEM_CELL_PROLIFERATION | -1.75 | 0.020 |
| GOBP_RESPONSE_TO_PAIN | -1.75 | 0.020 |
| GOBP_POTASSIUM_ION_HOMEOSTASIS | -1.75 | 0.020 |
| GOBP_REGULATION_OF_PEPTIDYL_TYROSINE_PHOSPHORYLATION | -1.75 | 0.020 |
| GOBP_DEVELOPMENTAL_PIGMENTATION | -1.75 | 0.020 |
| GOBP_PEPTIDYL_TYROSINE_MODIFICATION | -1.75 | 0.020 |
| GOBP_DEFENSE_RESPONSE_TO_BACTERIUM | -1.75 | 0.020 |
| GOBP_POSITIVE_REGULATION_OF_WOUND_HEALING | -1.75 | 0.021 |
| GOBP_ATRIAL_CARDIAC_MUSCLE_CELL_TO_AV_NODE_CELL_COMMUNICATION | -1.75 | 0.021 |
| GOBP_LYMPHOCYTE_CHEMOTAXIS | -1.75 | 0.021 |
| GOBP_DENDRITIC_CELL_CHEMOTAXIS | -1.75 | 0.021 |
| GOBP_CATECHOLAMINE_SECRETION | -1.75 | 0.021 |
| GOBP_REACTIVE_NITROGEN_SPECIES_METABOLIC_PROCESS | -1.75 | 0.021 |
| GOBP_EPITHELIAL_TO_MESENCHYMAL_TRANSITION | -1.75 | 0.021 |
| GOBP_POSITIVE_REGULATION_OF_NEURON_DIFFERENTIATION | -1.74 | 0.021 |
| GOBP_ACTIN_FILAMENT_DEPOLYMERIZATION | -1.74 | 0.021 |
| GOBP_INTERLEUKIN_6_PRODUCTION | -1.74 | 0.021 |
| GOBP_POSITIVE_REGULATION_OF_MACROPHAGE_MIGRATION | -1.74 | 0.021 |
| GOBP_NEURON_PROJECTION_GUIDANCE | -1.74 | 0.021 |
| GOBP_REGULATION_OF_SYSTEMIC_ARTERIAL_BLOOD_PRESSURE | -1.74 | 0.021 |
| GOBP_ANTIGEN_PROCESSING_AND_PRESENTATION_OF_PEPTIDE_OR_POLYSACCHARIDE_ANTIGEN_VIA_MHC_CLASS_II | -1.74 | 0.021 |
| GOBP_VASCULAR_ASSOCIATED_SMOOTH_MUSCLE_CELL_MIGRATION | -1.74 | 0.021 |
| GOBP_DOPAMINERGIC_NEURON_DIFFERENTIATION | -1.74 | 0.021 |
| GOBP_ANTIGEN_PROCESSING_AND_PRESENTATION_OF_EXOGENOUS_ANTIGEN | -1.74 | 0.021 |
| GOBP_STEROID_BIOSYNTHETIC_PROCESS | -1.74 | 0.021 |
| GOBP_ANTIGEN_PROCESSING_AND_PRESENTATION_OF_EXOGENOUS_PEPTIDE_ANTIGEN | -1.74 | 0.021 |
| GOMF_PHOSPHATIDYLETHANOLAMINE_BINDING | -1.74 | 0.021 |
| GOBP_RESPONSE_TO_RETINOIC_ACID | -1.74 | 0.021 |
| GOCC_CYTOSOLIC_RIBOSOME | -1.74 | 0.021 |
| GOMF_ALDEHYDE_DEHYDROGENASE_NAD_P_PLUS_ACTIVITY | -1.74 | 0.021 |
| GOBP_REGULATION_OF_ANATOMICAL_STRUCTURE_SIZE | -1.74 | 0.021 |
| GOBP_B_CELL_RECEPTOR_SIGNALING_PATHWAY | -1.74 | 0.021 |
| GOBP_STEROID_CATABOLIC_PROCESS | -1.74 | 0.021 |
| GOBP_NEURON_MATURATION | -1.74 | 0.021 |
| GOBP_ARTERY_DEVELOPMENT | -1.74 | 0.021 |
| GOBP_MIRNA_METABOLIC_PROCESS | -1.74 | 0.021 |
| GOBP_SOMITE_DEVELOPMENT | -1.74 | 0.022 |
| GOBP_POSITIVE_REGULATION_OF_MACROPHAGE_CHEMOTAXIS | -1.74 | 0.022 |
| GOBP_POSITIVE_REGULATION_OF_MUSCLE_HYPERTROPHY | -1.74 | 0.022 |
| GOBP_POSITIVE_REGULATION_OF_PHOSPHATIDYLINOSITOL_3_KINASE_PROTEIN_KINASE_B_SIGNAL_TRANSDUCTION | -1.74 | 0.022 |
| GOCC_T_TUBULE | -1.74 | 0.022 |
| GOBP_REGULATION_OF_CELL_SUBSTRATE_ADHESION | -1.74 | 0.022 |
| GOBP_TOLL_LIKE_RECEPTOR_SIGNALING_PATHWAY | -1.73 | 0.022 |
| GOBP_ENDOCARDIAL_CUSHION_MORPHOGENESIS | -1.73 | 0.022 |
| GOMF_TRANSMEMBRANE_RECEPTOR_PROTEIN_KINASE_ACTIVITY | -1.73 | 0.022 |
| GOMF_WNT_PROTEIN_BINDING | -1.73 | 0.022 |
| GOBP_POSITIVE_REGULATION_OF_LEUKOCYTE_CELL_CELL_ADHESION | -1.73 | 0.022 |
| GOBP_CELLULAR_RESPONSE_TO_ALCOHOL | -1.73 | 0.022 |
| GOBP_GASTRULATION | -1.73 | 0.022 |
| GOBP_CELLULAR_RESPONSE_TO_AMYLOID_BETA | -1.73 | 0.023 |
| GOMF_OXIDOREDUCTASE_ACTIVITY_ACTING_ON_THE_ALDEHYDE_OR_OXO_GROUP_OF_DONORS | -1.73 | 0.023 |
| GOBP_OUTFLOW_TRACT_SEPTUM_MORPHOGENESIS | -1.73 | 0.023 |
| GOBP_LYMPHOCYTE_MEDIATED_IMMUNITY | -1.73 | 0.023 |
| GOBP_MACROPHAGE_ACTIVATION_INVOLVED_IN_IMMUNE_RESPONSE | -1.73 | 0.023 |
| GOBP_TISSUE_REMODELING | -1.73 | 0.023 |
| GOMF_TRANSPORTER_REGULATOR_ACTIVITY | -1.73 | 0.023 |
| GOBP_EMBRYONIC_HINDLIMB_MORPHOGENESIS | -1.73 | 0.023 |
| GOBP_REGULATION_OF_CHOLESTEROL_EFFLUX | -1.73 | 0.023 |
| GOBP_POSITIVE_REGULATION_OF_B_CELL_ACTIVATION | -1.73 | 0.023 |
| GOBP_SUPEROXIDE_ANION_GENERATION | -1.73 | 0.023 |
| GOBP_T_CELL_ACTIVATION_INVOLVED_IN_IMMUNE_RESPONSE | -1.73 | 0.023 |
| GOBP_POSITIVE_REGULATION_OF_STRIATED_MUSCLE_CELL_DIFFERENTIATION | -1.73 | 0.023 |
| GOBP_EPITHELIAL_CELL_APOPTOTIC_PROCESS | -1.73 | 0.023 |
| GOBP_ENDOTHELIAL_CELL_PROLIFERATION | -1.73 | 0.023 |
| GOCC_PERINUCLEAR_ENDOPLASMIC_RETICULUM | -1.73 | 0.023 |
| GOBP_POTASSIUM_ION_TRANSPORT | -1.73 | 0.023 |
| GOMF_CYCLIC_NUCLEOTIDE_PHOSPHODIESTERASE_ACTIVITY | -1.72 | 0.024 |
| GOBP_REGULATION_OF_ACTIN_FILAMENT_BASED_MOVEMENT | -1.72 | 0.024 |
| GOBP_RESPONSE_TO_AMYLOID_BETA | -1.72 | 0.024 |
| GOCC_CATION_CHANNEL_COMPLEX | -1.72 | 0.024 |
| GOBP_CALCIUM_DEPENDENT_CELL_CELL_ADHESION_VIA_PLASMA_MEMBRANE_CELL_ADHESION_MOLECULES | -1.72 | 0.024 |
| GOBP_NEGATIVE_REGULATION_OF_PHAGOCYTOSIS | -1.72 | 0.024 |
| GOBP_CELLULAR_COMPONENT_MAINTENANCE | -1.72 | 0.025 |
| GOBP_PRIMARY_ALCOHOL_METABOLIC_PROCESS | -1.72 | 0.025 |
| GOBP_PIGMENTATION | -1.72 | 0.025 |
| GOBP_LEUKOCYTE_CELL_CELL_ADHESION | -1.72 | 0.025 |
| GOBP_REGULATION_OF_LIPID_STORAGE | -1.72 | 0.025 |
| GOBP_CORONARY_VASCULATURE_MORPHOGENESIS | -1.72 | 0.025 |
| GOBP_SYNAPSE_ASSEMBLY | -1.72 | 0.025 |
| GOBP_ALPHA_BETA_T_CELL_PROLIFERATION | -1.72 | 0.025 |
| GOBP_NEUROBLAST_PROLIFERATION | -1.72 | 0.025 |
| GOBP_NEGATIVE_REGULATION_OF_FATTY_ACID_METABOLIC_PROCESS | -1.72 | 0.025 |
| GOCC_VACUOLAR_LUMEN | -1.72 | 0.025 |
| GOBP_STEM_CELL_DIFFERENTIATION | -1.72 | 0.025 |
| GOBP_REGULATION_OF_ORGAN_GROWTH | -1.72 | 0.025 |
| GOBP_MAINTENANCE_OF_BLOOD_BRAIN_BARRIER | -1.72 | 0.025 |
| GOBP_REGULATION_OF_CELL_ADHESION_MEDIATED_BY_INTEGRIN | -1.72 | 0.025 |
| GOCC_LYSOSOMAL_LUMEN | -1.72 | 0.025 |
| GOBP_CELLULAR_RESPONSE_TO_CALCIUM_ION | -1.72 | 0.025 |
| GOBP_POSITIVE_REGULATION_OF_PHOSPHORYLATION | -1.72 | 0.025 |
| GOBP_TRICARBOXYLIC_ACID_CYCLE | -1.72 | 0.025 |
| GOBP_REGULATION_OF_RESPONSE_TO_WOUNDING | -1.72 | 0.025 |
| GOCC_FICOLIN_1_RICH_GRANULE_MEMBRANE | -1.72 | 0.025 |
| GOBP_VENTRICULAR_SEPTUM_DEVELOPMENT | -1.71 | 0.025 |
| GOBP_TYPE_II_INTERFERON_PRODUCTION | -1.71 | 0.025 |
| GOBP_POSITIVE_REGULATION_OF_EPITHELIAL_CELL_PROLIFERATION | -1.71 | 0.025 |
| GOBP_REGULATION_OF_ALCOHOL_BIOSYNTHETIC_PROCESS | -1.71 | 0.025 |
| GOBP_INTERLEUKIN_1_PRODUCTION | -1.71 | 0.025 |
| GOBP_REGULATION_OF_CELL_CELL_ADHESION_MEDIATED_BY_CADHERIN | -1.71 | 0.025 |
| GOBP_SMALL_MOLECULE_BIOSYNTHETIC_PROCESS | -1.71 | 0.025 |
| GOBP_REGULATION_OF_LIPASE_ACTIVITY | -1.71 | 0.025 |
| GOBP_REGULATION_OF_MACROPHAGE_DIFFERENTIATION | -1.71 | 0.026 |
| GOBP_EPIDERMIS_MORPHOGENESIS | -1.71 | 0.026 |
| GOBP_ZYMOGEN_ACTIVATION | -1.71 | 0.026 |
| GOBP_REGULATION_OF_PHOSPHOLIPASE_ACTIVITY | -1.71 | 0.026 |
| GOBP_LYMPH_VESSEL_MORPHOGENESIS | -1.71 | 0.026 |
| GOMF_TRANSMEMBRANE_RECEPTOR_PROTEIN_TYROSINE_KINASE_ACTIVITY | -1.71 | 0.026 |
| GOCC_EARLY_ENDOSOME | -1.71 | 0.025 |
| GOBP_REGULATION_OF_STEROL_TRANSPORT | -1.71 | 0.026 |
| GOBP_REGULATION_OF_LYMPHOCYTE_ACTIVATION | -1.71 | 0.026 |
| GOBP_REGULATION_OF_SYNCYTIUM_FORMATION_BY_PLASMA_MEMBRANE_FUSION | -1.71 | 0.026 |
| GOBP_REGULATION_OF_PROTEIN_CONTAINING_COMPLEX_DISASSEMBLY | -1.71 | 0.026 |
| GOBP_CELLULAR_RESPONSE_TO_KETONE | -1.71 | 0.026 |
| GOBP_BONE_MINERALIZATION | -1.71 | 0.026 |
| GOCC_SMALL_RIBOSOMAL_SUBUNIT | -1.71 | 0.026 |
| GOBP_SUBSTRATE_DEPENDENT_CELL_MIGRATION | -1.71 | 0.026 |
| GOBP_REGULATION_OF_PLATELET_DERIVED_GROWTH_FACTOR_RECEPTOR_SIGNALING_PATHWAY | -1.71 | 0.026 |
| GOBP_GLYCEROLIPID_CATABOLIC_PROCESS | -1.71 | 0.026 |
| GOBP_NEGATIVE_REGULATION_OF_G_PROTEIN_COUPLED_RECEPTOR_SIGNALING_PATHWAY | -1.71 | 0.026 |
| GOMF_COMPLEMENT_BINDING | -1.71 | 0.026 |
| GOBP_AMINOGLYCAN_METABOLIC_PROCESS | -1.71 | 0.026 |
| GOBP_POSITIVE_REGULATION_OF_INTERLEUKIN_1_BETA_PRODUCTION | -1.71 | 0.026 |
| GOBP_CELLULAR_RESPONSE_TO_LIPID | -1.71 | 0.026 |
| GOBP_LIPID_CATABOLIC_PROCESS | -1.71 | 0.026 |
| GOBP_CELL_JUNCTION_MAINTENANCE | -1.71 | 0.026 |
| GOBP_RESPONSE_TO_MECHANICAL_STIMULUS | -1.71 | 0.026 |
| GOBP_FEEDING_BEHAVIOR | -1.70 | 0.026 |
| GOBP_CELLULAR_RESPONSE_TO_OXIDATIVE_STRESS | -1.70 | 0.026 |
| GOBP_REGULATION_OF_NEUROBLAST_PROLIFERATION | -1.70 | 0.026 |
| GOBP_LEUKOCYTE_DIFFERENTIATION | -1.70 | 0.026 |
| GOMF_GROWTH_FACTOR_BINDING | -1.70 | 0.026 |
| GOCC_LARGE_RIBOSOMAL_SUBUNIT | -1.70 | 0.026 |
| GOBP_BRANCH_ELONGATION_OF_AN_EPITHELIUM | -1.70 | 0.026 |
| GOBP_RENAL_ABSORPTION | -1.70 | 0.027 |
| GOBP_NITRIC_OXIDE_SYNTHASE_BIOSYNTHETIC_PROCESS | -1.70 | 0.027 |
| GOBP_CARDIAC_SEPTUM_DEVELOPMENT | -1.70 | 0.027 |
| GOBP_REGULATION_OF_SUPEROXIDE_ANION_GENERATION | -1.70 | 0.027 |
| GOBP_REGULATION_OF_TUBE_SIZE | -1.70 | 0.027 |
| GOBP_POSITIVE_REGULATION_OF_INTERLEUKIN_1_PRODUCTION | -1.70 | 0.027 |
| GOBP_RESPONSE_TO_FIBROBLAST_GROWTH_FACTOR | -1.70 | 0.027 |
| GOBP_NEGATIVE_REGULATION_OF_CELL_DIFFERENTIATION | -1.70 | 0.027 |
| GOBP_MUSCLE_ORGAN_DEVELOPMENT | -1.70 | 0.027 |
| GOBP_IMMUNE_RESPONSE_REGULATING_CELL_SURFACE_RECEPTOR_SIGNALING_PATHWAY | -1.70 | 0.027 |
| GOBP_POSITIVE_REGULATION_OF_ENDOTHELIAL_CELL_MIGRATION | -1.70 | 0.027 |
| GOBP_REGULATION_OF_MONOATOMIC_ION_TRANSMEMBRANE_TRANSPORT | -1.70 | 0.027 |
| GOBP_REGULATION_OF_PHOSPHOLIPASE_C_ACTIVITY | -1.70 | 0.027 |
| GOBP_GLAND_MORPHOGENESIS | -1.70 | 0.027 |
| GOBP_REGULATION_OF_STEROID_METABOLIC_PROCESS | -1.70 | 0.027 |
| GOBP_NEGATIVE_REGULATION_OF_PROTEIN_LOCALIZATION_TO_NUCLEUS | -1.70 | 0.027 |
| GOBP_PIGMENT_CELL_DIFFERENTIATION | -1.70 | 0.027 |
| GOBP_POSITIVE_REGULATION_OF_VASCULAR_ENDOTHELIAL_GROWTH_FACTOR_PRODUCTION | -1.70 | 0.027 |
| GOBP_HEART_GROWTH | -1.70 | 0.027 |
| GOBP_REGULATION_OF_SODIUM_ION_TRANSMEMBRANE_TRANSPORT | -1.70 | 0.027 |
| GOBP_SENSORY_PERCEPTION_OF_TEMPERATURE_STIMULUS | -1.70 | 0.027 |
| GOBP_RESPONSE_TO_ESTROGEN | -1.70 | 0.027 |
| GOCC_BASOLATERAL_PLASMA_MEMBRANE | -1.70 | 0.027 |
| GOBP_RENAL_SYSTEM_PROCESS_INVOLVED_IN_REGULATION_OF_SYSTEMIC_ARTERIAL_BLOOD_PRESSURE | -1.70 | 0.027 |
| GOBP_REGULATION_OF_STRIATED_MUSCLE_CELL_DIFFERENTIATION | -1.70 | 0.027 |
| GOBP_EMBRYONIC_HEART_TUBE_DEVELOPMENT | -1.70 | 0.027 |
| GOBP_LIPID_HOMEOSTASIS | -1.70 | 0.027 |
| GOBP_NEGATIVE_REGULATION_OF_SIGNAL_TRANSDUCTION_IN_ABSENCE_OF_LIGAND | -1.70 | 0.027 |
| GOBP_CELLULAR_RESPONSE_TO_ESTRADIOL_STIMULUS | -1.70 | 0.027 |
| GOBP_REGULATION_OF_HEMOPOIESIS | -1.70 | 0.027 |
| GOBP_PROSTANOID_BIOSYNTHETIC_PROCESS | -1.70 | 0.027 |
| GOMF_AMYLOID_BETA_BINDING | -1.70 | 0.027 |
| GOBP_NOREPINEPHRINE_TRANSPORT | -1.70 | 0.027 |
| GOBP_ESTABLISHMENT_OF_TISSUE_POLARITY | -1.70 | 0.027 |
| GOBP_REGULATION_OF_INFLAMMATORY_RESPONSE_TO_ANTIGENIC_STIMULUS | -1.70 | 0.027 |
| GOBP_POSITIVE_REGULATION_OF_SMAD_PROTEIN_SIGNAL_TRANSDUCTION | -1.70 | 0.027 |
| GOBP_ALCOHOL_BIOSYNTHETIC_PROCESS | -1.69 | 0.027 |
| GOCC_CHITOSOME | -1.69 | 0.027 |
| GOBP_REGULATION_OF_EXTRINSIC_APOPTOTIC_SIGNALING_PATHWAY_IN_ABSENCE_OF_LIGAND | -1.69 | 0.027 |
| GOBP_DITERPENOID_METABOLIC_PROCESS | -1.69 | 0.027 |
| GOBP_EYE_MORPHOGENESIS | -1.69 | 0.028 |
| GOBP_NEGATIVE_REGULATION_OF_SUPRAMOLECULAR_FIBER_ORGANIZATION | -1.69 | 0.028 |
| GOBP_POSITIVE_REGULATION_OF_PRODUCTION_OF_MOLECULAR_MEDIATOR_OF_IMMUNE_RESPONSE | -1.69 | 0.028 |
| GOBP_MEMBRANE_DEPOLARIZATION_DURING_CARDIAC_MUSCLE_CELL_ACTION_POTENTIAL | -1.69 | 0.028 |
| GOBP_POSITIVE_REGULATION_OF_PHOSPHOLIPASE_ACTIVITY | -1.69 | 0.028 |
| GOBP_MEMBRANE_REPOLARIZATION_DURING_ACTION_POTENTIAL | -1.69 | 0.028 |
| GOBP_NEGATIVE_REGULATION_OF_LIPID_METABOLIC_PROCESS | -1.69 | 0.028 |
| GOBP_INDOLE_CONTAINING_COMPOUND_METABOLIC_PROCESS | -1.69 | 0.028 |
| GOMF_UDP_GLYCOSYLTRANSFERASE_ACTIVITY | -1.69 | 0.028 |
| GOBP_POSITIVE_REGULATION_OF_T_CELL_CYTOKINE_PRODUCTION | -1.69 | 0.028 |
| GOBP_VENTRICULAR_SEPTUM_MORPHOGENESIS | -1.69 | 0.029 |
| GOBP_RETINA_VASCULATURE_DEVELOPMENT_IN_CAMERA_TYPE_EYE | -1.69 | 0.029 |
| GOMF_STEROL_BINDING | -1.69 | 0.029 |
| GOBP_LUNG_ALVEOLUS_DEVELOPMENT | -1.69 | 0.029 |
| GOBP_POSITIVE_REGULATION_OF_INTERLEUKIN_2_PRODUCTION | -1.69 | 0.029 |
| GOBP_PHAGOCYTOSIS_RECOGNITION | -1.69 | 0.029 |
| GOBP_REGULATION_OF_INFLAMMASOME_MEDIATED_SIGNALING_PATHWAY | -1.69 | 0.029 |
| GOBP_MESONEPHROS_DEVELOPMENT | -1.69 | 0.029 |
| GOBP_REGULATION_OF_MORPHOGENESIS_OF_A_BRANCHING_STRUCTURE | -1.69 | 0.029 |
| GOMF_ATP_DEPENDENT_PROTEIN_FOLDING_CHAPERONE | -1.69 | 0.029 |
| GOCC_FICOLIN_1_RICH_GRANULE | -1.68 | 0.029 |
| GOMF_CORECEPTOR_ACTIVITY | -1.68 | 0.029 |
| GOBP_REGULATION_OF_EPITHELIAL_CELL_APOPTOTIC_PROCESS | -1.68 | 0.029 |
| GOBP_ACROSOME_REACTION | -1.68 | 0.029 |
| GOBP_FAT_CELL_DIFFERENTIATION | -1.68 | 0.029 |
| GOBP_REGULATION_OF_RECEPTOR_INTERNALIZATION | -1.68 | 0.029 |
| GOBP_CELLULAR_RESPONSE_TO_CHEMICAL_STRESS | -1.68 | 0.029 |
| GOBP_POSITIVE_REGULATION_OF_NITRIC_OXIDE_METABOLIC_PROCESS | -1.68 | 0.029 |
| GOBP_POSITIVE_REGULATION_OF_INTERLEUKIN_10_PRODUCTION | -1.68 | 0.029 |
| GOBP_ACTIVATION_OF_IMMUNE_RESPONSE | -1.68 | 0.029 |
| GOBP_CELL_CELL_ADHESION_MEDIATED_BY_CADHERIN | -1.68 | 0.030 |
| GOBP_RESPONSE_TO_COCAINE | -1.68 | 0.030 |
| GOBP_CGMP_MEDIATED_SIGNALING | -1.68 | 0.030 |
| GOMF_CYTOKINE_RECEPTOR_BINDING | -1.68 | 0.030 |
| GOBP_NEURON_FATE_COMMITMENT | -1.68 | 0.030 |
| GOBP_POSITIVE_REGULATION_OF_MUSCLE_CELL_DIFFERENTIATION | -1.68 | 0.030 |
| GOBP_POTASSIUM_ION_IMPORT_ACROSS_PLASMA_MEMBRANE | -1.68 | 0.030 |
| GOBP_CAMP_MEDIATED_SIGNALING | -1.68 | 0.030 |
| GOBP_REGULATION_OF_CHOLESTEROL_METABOLIC_PROCESS | -1.68 | 0.030 |
| GOBP_SENSORY_SYSTEM_DEVELOPMENT | -1.68 | 0.030 |
| GOBP_LOW_DENSITY_LIPOPROTEIN_PARTICLE_CLEARANCE | -1.68 | 0.030 |
| GOBP_EMBRYONIC_MORPHOGENESIS | -1.68 | 0.030 |
| GOBP_PYRIDINE_CONTAINING_COMPOUND_BIOSYNTHETIC_PROCESS | -1.68 | 0.030 |
| GOBP_GLIAL_CELL_MIGRATION | -1.68 | 0.030 |
| GOBP_ORGAN_GROWTH | -1.68 | 0.030 |
| GOBP_ADRENERGIC_RECEPTOR_SIGNALING_PATHWAY | -1.68 | 0.030 |
| GOBP_REPRODUCTIVE_SYSTEM_DEVELOPMENT | -1.68 | 0.030 |
| GOBP_REGULATION_OF_RECEPTOR_MEDIATED_ENDOCYTOSIS | -1.68 | 0.030 |
| GOBP_INTERLEUKIN_8_PRODUCTION | -1.68 | 0.030 |
| GOBP_I_KAPPAB_PHOSPHORYLATION | -1.68 | 0.030 |
| GOBP_REGULATION_OF_DNA_BINDING_TRANSCRIPTION_FACTOR_ACTIVITY | -1.68 | 0.030 |
| GOBP_BLOOD_VESSEL_ENDOTHELIAL_CELL_MIGRATION | -1.68 | 0.030 |
| GOBP_RESPONSE_TO_CATECHOLAMINE | -1.68 | 0.030 |
| GOBP_CANONICAL_WNT_SIGNALING_PATHWAY | -1.68 | 0.030 |
| GOBP_NEGATIVE_REGULATION_OF_RESPONSE_TO_EXTERNAL_STIMULUS | -1.68 | 0.030 |
| GOBP_OVULATION_CYCLE | -1.68 | 0.030 |
| GOBP_CELLULAR_RESPONSE_TO_INORGANIC_SUBSTANCE | -1.68 | 0.031 |
| GOBP_POSITIVE_REGULATION_OF_CELL_DEVELOPMENT | -1.68 | 0.031 |
| GOBP_SODIUM_ION_TRANSMEMBRANE_TRANSPORT | -1.68 | 0.031 |
| GOBP_FATTY_ACYL_COA_BIOSYNTHETIC_PROCESS | -1.67 | 0.031 |
| GOBP_REGULATION_OF_B_CELL_DIFFERENTIATION | -1.67 | 0.031 |
| GOBP_POSITIVE_REGULATION_OF_IMMUNOGLOBULIN_PRODUCTION | -1.67 | 0.031 |
| GOMF_PROTEIN_FOLDING_CHAPERONE_BINDING | -1.67 | 0.031 |
| GOBP_SYNAPSE_ORGANIZATION | -1.67 | 0.031 |
| GOBP_POSITIVE_REGULATION_OF_MONOCYTE_CHEMOTAXIS | -1.67 | 0.031 |
| GOBP_POSITIVE_REGULATION_OF_PEPTIDYL_TYROSINE_PHOSPHORYLATION | -1.67 | 0.031 |
| GOBP_GRANULOCYTE_ACTIVATION | -1.67 | 0.031 |
| GOBP_CENTRAL_NERVOUS_SYSTEM_PROJECTION_NEURON_AXONOGENESIS | -1.67 | 0.031 |
| GOMF_SOLUTE_PROTON_SYMPORTER_ACTIVITY | -1.67 | 0.031 |
| GOBP_POSITIVE_REGULATION_OF_TUMOR_NECROSIS_FACTOR_SUPERFAMILY_CYTOKINE_PRODUCTION | -1.67 | 0.031 |
| GOBP_VASCULAR_ENDOTHELIAL_GROWTH_FACTOR_RECEPTOR_SIGNALING_PATHWAY | -1.67 | 0.031 |
| GOBP_TISSUE_REGENERATION | -1.67 | 0.031 |
| GOBP_LYMPH_NODE_DEVELOPMENT | -1.67 | 0.031 |
| GOBP_POSITIVE_REGULATION_OF_HUMORAL_IMMUNE_RESPONSE | -1.67 | 0.031 |
| GOBP_ESTABLISHMENT_OF_EPITHELIAL_CELL_APICAL_BASAL_POLARITY | -1.67 | 0.031 |
| GOBP_C21_STEROID_HORMONE_METABOLIC_PROCESS | -1.67 | 0.031 |
| GOBP_REGULATION_OF_T_CELL_ACTIVATION | -1.67 | 0.031 |
| GOBP_REGULATION_OF_CELL_SIZE | -1.67 | 0.031 |
| GOBP_RESPONSE_TO_VITAMIN | -1.67 | 0.031 |
| GOCC_PROTEASOME_COMPLEX | -1.67 | 0.031 |
| GOBP_CELL_MATRIX_ADHESION | -1.67 | 0.031 |
| GOBP_REGULATION_OF_VASCULAR_ENDOTHELIAL_GROWTH_FACTOR_RECEPTOR_SIGNALING_PATHWAY | -1.67 | 0.031 |
| GOBP_HOMEOSTASIS_OF_NUMBER_OF_CELLS_WITHIN_A_TISSUE | -1.67 | 0.031 |
| GOCC_PIGMENT_GRANULE | -1.67 | 0.031 |
| GOBP_ALPHA_BETA_T_CELL_ACTIVATION | -1.67 | 0.031 |
| GOBP_MUCOPOLYSACCHARIDE_METABOLIC_PROCESS | -1.67 | 0.032 |
| GOBP_REGULATION_OF_STEM_CELL_DIFFERENTIATION | -1.67 | 0.032 |
| GOBP_NEGATIVE_REGULATION_OF_CELL_ACTIVATION | -1.67 | 0.032 |
| GOBP_MEMBRANE_DEPOLARIZATION_DURING_ACTION_POTENTIAL | -1.67 | 0.032 |
| GOBP_TRANSFORMING_GROWTH_FACTOR_BETA_RECEPTOR_SIGNALING_PATHWAY | -1.67 | 0.032 |
| GOCC_CAVEOLA | -1.67 | 0.032 |
| GOBP_MIDBRAIN_DEVELOPMENT | -1.67 | 0.032 |
| GOBP_VENTRICULAR_CARDIAC_MUSCLE_TISSUE_DEVELOPMENT | -1.67 | 0.032 |
| GOBP_REGULATION_OF_SYSTEMIC_ARTERIAL_BLOOD_PRESSURE_BY_HORMONE | -1.67 | 0.032 |
| GOBP_POSITIVE_REGULATION_OF_DNA_BINDING_TRANSCRIPTION_FACTOR_ACTIVITY | -1.67 | 0.032 |
| GOBP_LYMPHANGIOGENESIS | -1.67 | 0.032 |
| GOBP_FC_GAMMA_RECEPTOR_SIGNALING_PATHWAY | -1.67 | 0.032 |
| GOCC_SMOOTH_ENDOPLASMIC_RETICULUM | -1.67 | 0.032 |
| GOBP_REGULATION_OF_EXTRINSIC_APOPTOTIC_SIGNALING_PATHWAY | -1.67 | 0.032 |
| GOBP_EPITHELIAL_CELL_PROLIFERATION | -1.66 | 0.032 |
| GOBP_FATTY_ACID_METABOLIC_PROCESS | -1.66 | 0.032 |
| GOCC_INTERCALATED_DISC | -1.66 | 0.033 |
| GOBP_MYELOID_DENDRITIC_CELL_ACTIVATION | -1.66 | 0.033 |
| GOBP_POSITIVE_REGULATION_OF_PROTEIN_CONTAINING_COMPLEX_ASSEMBLY | -1.66 | 0.033 |
| GOBP_REGULATION_OF_SMAD_PROTEIN_SIGNAL_TRANSDUCTION | -1.66 | 0.033 |
| GOBP_REGULATION_OF_MONOATOMIC_ION_TRANSPORT | -1.66 | 0.033 |
| GOCC_TERTIARY_GRANULE_MEMBRANE | -1.66 | 0.033 |
| GOBP_B_CELL_ACTIVATION | -1.66 | 0.033 |
| GOBP_CELL_COMMUNICATION_BY_ELECTRICAL_COUPLING | -1.66 | 0.033 |
| GOMF_TAU_PROTEIN_BINDING | -1.66 | 0.033 |
| GOBP_POSITIVE_REGULATION_OF_GLIAL_CELL_PROLIFERATION | -1.66 | 0.033 |
| GOBP_NEPHRON_DEVELOPMENT | -1.66 | 0.033 |
| GOMF_CALCIUM_ION_BINDING | -1.66 | 0.034 |
| GOBP_NEURON_MIGRATION | -1.66 | 0.034 |
| GOBP_TERTIARY_ALCOHOL_METABOLIC_PROCESS | -1.66 | 0.034 |
| GOBP_EMBRYONIC_DIGIT_MORPHOGENESIS | -1.66 | 0.034 |
| GOBP_RESPONSE_TO_ESTRADIOL | -1.66 | 0.034 |
| GOBP_LUNG_CELL_DIFFERENTIATION | -1.66 | 0.034 |
| GOBP_ENDOCARDIAL_CUSHION_FORMATION | -1.66 | 0.034 |
| GOBP_REGULATION_OF_NEURON_PROJECTION_DEVELOPMENT | -1.66 | 0.034 |
| GOBP_IMMUNE_SYSTEM_DEVELOPMENT | -1.66 | 0.034 |
| GOBP_CHONDROITIN_SULFATE_PROTEOGLYCAN_METABOLIC_PROCESS | -1.66 | 0.034 |
| GOBP_CELL_AGGREGATION | -1.66 | 0.034 |
| GOBP_REGULATION_OF_STEROID_BIOSYNTHETIC_PROCESS | -1.66 | 0.034 |
| GOBP_WNT_SIGNALING_PATHWAY | -1.66 | 0.034 |
| GOBP_RESPONSE_TO_DEXAMETHASONE | -1.66 | 0.034 |
| GOBP_NEGATIVE_REGULATION_OF_AMINE_TRANSPORT | -1.66 | 0.034 |
| GOBP_SMAD_PROTEIN_SIGNAL_TRANSDUCTION | -1.66 | 0.034 |
| GOBP_PROTEIN_DEPOLYMERIZATION | -1.66 | 0.034 |
| GOMF_PROTEIN_LIPID_COMPLEX_BINDING | -1.66 | 0.034 |
| GOBP_NEGATIVE_REGULATION_OF_LIPID_CATABOLIC_PROCESS | -1.65 | 0.034 |
| GOBP_REGULATION_OF_LIPID_METABOLIC_PROCESS | -1.65 | 0.034 |
| GOBP_NEUTRAL_LIPID_METABOLIC_PROCESS | -1.65 | 0.034 |
| GOBP_LYTIC_VACUOLE_ORGANIZATION | -1.65 | 0.034 |
| GOBP_POSITIVE_REGULATION_OF_NUCLEAR_DIVISION | -1.65 | 0.035 |
| GOBP_CENTRAL_NERVOUS_SYSTEM_NEURON_AXONOGENESIS | -1.65 | 0.035 |
| GOBP_RESPONSE_TO_TUMOR_NECROSIS_FACTOR | -1.65 | 0.035 |
| GOBP_FOREBRAIN_DEVELOPMENT | -1.65 | 0.035 |
| GOBP_ENDOTHELIUM_DEVELOPMENT | -1.65 | 0.035 |
| GOBP_NEGATIVE_REGULATION_OF_CYTOKINE_PRODUCTION | -1.65 | 0.035 |
| GOCC_INTEGRIN_COMPLEX | -1.65 | 0.035 |
| GOBP_RESPONSE_TO_CALCIUM_ION | -1.65 | 0.035 |
| GOBP_POSITIVE_REGULATION_OF_ALPHA_BETA_T_CELL_ACTIVATION | -1.65 | 0.035 |
| GOBP_REGULATION_OF_SMOOTH_MUSCLE_CONTRACTION | -1.65 | 0.035 |
| GOCC_PHAGOCYTIC_VESICLE_MEMBRANE | -1.65 | 0.035 |
| GOBP_PLASMINOGEN_ACTIVATION | -1.65 | 0.035 |
| GOBP_RECEPTOR_INTERNALIZATION | -1.65 | 0.035 |
| GOCC_SPECIFIC_GRANULE | -1.65 | 0.035 |
| GOBP_C21_STEROID_HORMONE_BIOSYNTHETIC_PROCESS | -1.65 | 0.036 |
| GOBP_POSITIVE_REGULATION_OF_TYPE_2_IMMUNE_RESPONSE | -1.65 | 0.036 |
| GOBP_NEGATIVE_REGULATION_OF_TYPE_II_INTERFERON_PRODUCTION | -1.65 | 0.036 |
| GOBP_EMBRYONIC_PLACENTA_MORPHOGENESIS | -1.65 | 0.036 |
| GOBP_REPRODUCTIVE_BEHAVIOR | -1.65 | 0.036 |
| GOBP_LYMPHOCYTE_MIGRATION | -1.65 | 0.036 |
| GOBP_TERPENOID_METABOLIC_PROCESS | -1.65 | 0.036 |
| GOBP_T_CELL_CHEMOTAXIS | -1.65 | 0.036 |
| GOBP_CARDIAC_CONDUCTION_SYSTEM_DEVELOPMENT | -1.65 | 0.036 |
| GOMF_TRANSFERASE_ACTIVITY_TRANSFERRING_SULPHUR_CONTAINING_GROUPS | -1.65 | 0.036 |
| GOBP_REGULATION_OF_ACTION_POTENTIAL | -1.65 | 0.036 |
| GOBP_LABYRINTHINE_LAYER_MORPHOGENESIS | -1.65 | 0.036 |
| GOBP_SYMBIONT_ENTRY_INTO_HOST | -1.64 | 0.036 |
| GOBP_REGULATION_OF_SODIUM_ION_TRANSMEMBRANE_TRANSPORTER_ACTIVITY | -1.64 | 0.036 |
| GOBP_FATTY_ACID_BIOSYNTHETIC_PROCESS | -1.64 | 0.036 |
| GOMF_SODIUM_CHANNEL_ACTIVITY | -1.64 | 0.036 |
| GOBP_METANEPHRIC_NEPHRON_DEVELOPMENT | -1.64 | 0.037 |
| GOCC_ORGANELLAR_RIBOSOME | -1.64 | 0.037 |
| GOBP_T_CELL_SELECTION | -1.64 | 0.037 |
| GOBP_POSITIVE_REGULATION_OF_LYMPHOCYTE_DIFFERENTIATION | -1.64 | 0.037 |
| GOBP_CELLULAR_RESPONSE_TO_ORGANIC_CYCLIC_COMPOUND | -1.64 | 0.037 |
| GOBP_REGULATION_OF_CELLULAR_EXTRAVASATION | -1.64 | 0.037 |
| GOCC_CELL_BODY | -1.64 | 0.037 |
| GOBP_ANATOMICAL_STRUCTURE_MATURATION | -1.64 | 0.037 |
| GOBP_EMBRYONIC_PATTERN_SPECIFICATION | -1.64 | 0.037 |
| GOBP_POSITIVE_REGULATION_OF_REACTIVE_OXYGEN_SPECIES_METABOLIC_PROCESS | -1.64 | 0.037 |
| GOBP_DIENCEPHALON_DEVELOPMENT | -1.64 | 0.037 |
| GOBP_REGULATION_OF_SYSTEMIC_ARTERIAL_BLOOD_PRESSURE_BY_RENIN_ANGIOTENSIN | -1.64 | 0.037 |
| GOBP_AXON_DEVELOPMENT | -1.64 | 0.037 |
| GOCC_PROTEASOME_ACCESSORY_COMPLEX | -1.64 | 0.037 |
| GOBP_POSITIVE_REGULATION_OF_TYPE_II_INTERFERON_PRODUCTION | -1.64 | 0.037 |
| GOBP_RESPONSE_TO_MINERALOCORTICOID | -1.64 | 0.037 |
| GOBP_NEGATIVE_REGULATION_OF_STRIATED_MUSCLE_CELL_DIFFERENTIATION | -1.64 | 0.037 |
| GOBP_REGULATION_OF_COLLAGEN_METABOLIC_PROCESS | -1.64 | 0.037 |
| GOBP_LENS_FIBER_CELL_DIFFERENTIATION | -1.64 | 0.037 |
| GOBP_POSITIVE_REGULATION_OF_INFLAMMASOME_MEDIATED_SIGNALING_PATHWAY | -1.64 | 0.037 |
| GOBP_RESPONSE_TO_FLUID_SHEAR_STRESS | -1.64 | 0.037 |
| GOBP_POSITIVE_REGULATION_OF_SYNAPSE_ASSEMBLY | -1.64 | 0.037 |
| GOBP_SMOOTH_MUSCLE_CONTRACTION | -1.64 | 0.037 |
| GOBP_NEGATIVE_REGULATION_OF_CELL_ADHESION | -1.64 | 0.037 |
| GOBP_PROSTANOID_METABOLIC_PROCESS | -1.64 | 0.037 |
| GOBP_REGULATION_OF_CELLULAR_RESPONSE_TO_GROWTH_FACTOR_STIMULUS | -1.64 | 0.037 |
| GOBP_CELLULAR_RESPONSE_TO_ALKALOID | -1.64 | 0.037 |
| GOBP_REGULATION_OF_IMMUNE_EFFECTOR_PROCESS | -1.64 | 0.037 |
| GOBP_CHONDROITIN_SULFATE_PROTEOGLYCAN_BIOSYNTHETIC_PROCESS | -1.64 | 0.037 |
| GOBP_RESPONSE_TO_ETHANOL | -1.64 | 0.037 |
| GOBP_INTERLEUKIN_10_PRODUCTION | -1.64 | 0.037 |
| GOBP_REGULATION_OF_NEUTROPHIL_MIGRATION | -1.64 | 0.037 |
| GOBP_REGULATION_OF_DEFENSE_RESPONSE_TO_BACTERIUM | -1.64 | 0.038 |
| GOBP_SEX_DIFFERENTIATION | -1.64 | 0.038 |
| GOBP_REGULATION_OF_PH | -1.64 | 0.038 |
| GOBP_EMBRYONIC_ORGAN_DEVELOPMENT | -1.64 | 0.038 |
| GOBP_EPITHELIAL_TUBE_FORMATION | -1.64 | 0.038 |
| GOBP_CELL_VOLUME_HOMEOSTASIS | -1.63 | 0.038 |
| GOBP_REGULATION_OF_VASCULAR_PERMEABILITY | -1.63 | 0.038 |
| GOBP_NEGATIVE_REGULATION_OF_BLOOD_PRESSURE | -1.63 | 0.038 |
| GOBP_REGULATION_OF_MEMBRANE_POTENTIAL | -1.63 | 0.038 |
| GOBP_CALCIUM_ION_HOMEOSTASIS | -1.63 | 0.038 |
| GOBP_INNERVATION | -1.63 | 0.038 |
| GOBP_INORGANIC_ION_IMPORT_ACROSS_PLASMA_MEMBRANE | -1.63 | 0.038 |
| GOCC_BASAL_PART_OF_CELL | -1.63 | 0.038 |
| GOBP_DEFENSE_RESPONSE_TO_GRAM_NEGATIVE_BACTERIUM | -1.63 | 0.039 |
| GOCC_ENDOPLASMIC_RETICULUM_GOLGI_INTERMEDIATE_COMPARTMENT | -1.63 | 0.039 |
| GOBP_REGULATION_OF_CARDIAC_MUSCLE_CELL_PROLIFERATION | -1.63 | 0.039 |
| GOBP_NEUTROPHIL_ACTIVATION_INVOLVED_IN_IMMUNE_RESPONSE | -1.63 | 0.039 |
| GOBP_FAT_SOLUBLE_VITAMIN_METABOLIC_PROCESS | -1.63 | 0.039 |
| GOBP_GLUCAN_BIOSYNTHETIC_PROCESS | -1.63 | 0.039 |
| GOBP_REGULATION_OF_KETONE_BIOSYNTHETIC_PROCESS | -1.63 | 0.039 |
| GOBP_LONG_CHAIN_FATTY_ACYL_COA_BIOSYNTHETIC_PROCESS | -1.63 | 0.039 |
| GOBP_NEGATIVE_REGULATION_OF_IMMUNE_SYSTEM_PROCESS | -1.63 | 0.039 |
| GOBP_IMMUNE_RESPONSE_REGULATING_SIGNALING_PATHWAY | -1.63 | 0.039 |
| GOBP_FOREBRAIN_CELL_MIGRATION | -1.63 | 0.039 |
| GOBP_STEROID_HORMONE_BIOSYNTHETIC_PROCESS | -1.63 | 0.039 |
| GOBP_NEGATIVE_REGULATION_OF_RESPONSE_TO_WOUNDING | -1.63 | 0.039 |
| GOCC_DENSE_CORE_GRANULE | -1.63 | 0.039 |
| GOBP_CHAPERONE_MEDIATED_PROTEIN_FOLDING | -1.63 | 0.039 |
| GOBP_NEGATIVE_REGULATION_OF_CHONDROCYTE_DIFFERENTIATION | -1.63 | 0.039 |
| GOBP_MACROPHAGE_DIFFERENTIATION | -1.63 | 0.039 |
| GOBP_ANDROGEN_METABOLIC_PROCESS | -1.63 | 0.039 |
| GOBP_REGULATION_OF_FAT_CELL_DIFFERENTIATION | -1.63 | 0.039 |
| GOBP_EMBRYONIC_FORELIMB_MORPHOGENESIS | -1.63 | 0.039 |
| GOBP_CHAPERONE_COFACTOR_DEPENDENT_PROTEIN_REFOLDING | -1.63 | 0.039 |
| GOBP_CD4_POSITIVE_ALPHA_BETA_T_CELL_ACTIVATION | -1.63 | 0.039 |
| GOBP_MYOBLAST_PROLIFERATION | -1.63 | 0.040 |
| GOBP_POSITIVE_REGULATION_OF_DEFENSE_RESPONSE | -1.63 | 0.040 |
| GOBP_POSITIVE_REGULATION_OF_MYELOID_CELL_DIFFERENTIATION | -1.63 | 0.040 |
| GOBP_MAINTENANCE_OF_LOCATION | -1.63 | 0.040 |
| GOBP_CARDIAC_NEURAL_CREST_CELL_DIFFERENTIATION_INVOLVED_IN_HEART_DEVELOPMENT | -1.63 | 0.040 |
| GOBP_MORPHOGENESIS_OF_A_POLARIZED_EPITHELIUM | -1.62 | 0.040 |
| GOCC_PRESYNAPTIC_MEMBRANE | -1.62 | 0.040 |
| GOBP_POSITIVE_REGULATION_OF_SMOOTH_MUSCLE_CELL_MIGRATION | -1.62 | 0.040 |
| GOBP_FORELIMB_MORPHOGENESIS | -1.62 | 0.041 |
| GOMF_STEROL_TRANSFER_ACTIVITY | -1.62 | 0.041 |
| GOBP_MHC_CLASS_II_BIOSYNTHETIC_PROCESS | -1.62 | 0.041 |
| GOBP_CELLULAR_RESPONSE_TO_TOXIC_SUBSTANCE | -1.62 | 0.041 |
| GOBP_NEURON_CELLULAR_HOMEOSTASIS | -1.62 | 0.041 |
| GOBP_SYMPATHETIC_NERVOUS_SYSTEM_DEVELOPMENT | -1.62 | 0.041 |
| GOBP_RESPONSE_TO_LAMINAR_FLUID_SHEAR_STRESS | -1.62 | 0.041 |
| GOBP_NEGATIVE_REGULATION_OF_TUMOR_NECROSIS_FACTOR_SUPERFAMILY_CYTOKINE_PRODUCTION | -1.62 | 0.041 |
| GOBP_POSITIVE_REGULATION_OF_KINASE_ACTIVITY | -1.62 | 0.041 |
| GOBP_MOLTING_CYCLE | -1.62 | 0.042 |
| GOMF_SODIUM_CHANNEL_REGULATOR_ACTIVITY | -1.62 | 0.042 |
| GOBP_RESPONSE_TO_ACID_CHEMICAL | -1.62 | 0.042 |
| GOBP_REGULATION_OF_MUSCLE_HYPERTROPHY | -1.62 | 0.042 |
| GOCC_CALCIUM_CHANNEL_COMPLEX | -1.62 | 0.042 |
| GOBP_MONOCARBOXYLIC_ACID_METABOLIC_PROCESS | -1.62 | 0.042 |
| GOBP_SKELETAL_MUSCLE_CELL_DIFFERENTIATION | -1.62 | 0.042 |
| GOBP_RESPONSE_TO_ELECTRICAL_STIMULUS | -1.62 | 0.042 |
| GOBP_AMINE_BIOSYNTHETIC_PROCESS | -1.62 | 0.042 |
| GOBP_T_CELL_MIGRATION | -1.62 | 0.042 |
| GOCC_GOLGI_LUMEN | -1.62 | 0.042 |
| GOCC_APICAL_DENDRITE | -1.62 | 0.042 |
| GOBP_REGULATION_OF_ENDOTHELIAL_CELL_MIGRATION | -1.62 | 0.042 |
| GOBP_MACROPHAGE_CYTOKINE_PRODUCTION | -1.62 | 0.042 |
| GOBP_POSITIVE_REGULATION_OF_TRANSFERASE_ACTIVITY | -1.62 | 0.043 |
| GOBP_RESPONSE_TO_AMPHETAMINE | -1.62 | 0.043 |
| GOBP_LYMPHOCYTE_DIFFERENTIATION | -1.62 | 0.043 |
| GOBP_NEGATIVE_REGULATION_OF_EPITHELIAL_CELL_DIFFERENTIATION | -1.62 | 0.043 |
| GOBP_REGULATION_OF_PRODUCTION_OF_MOLECULAR_MEDIATOR_OF_IMMUNE_RESPONSE | -1.61 | 0.043 |
| GOBP_REGULATION_OF_VESICLE_FUSION | -1.61 | 0.043 |
| GOBP_IMPORT_ACROSS_PLASMA_MEMBRANE | -1.61 | 0.043 |
| GOBP_CELLULAR_RESPONSE_TO_PROSTAGLANDIN_STIMULUS | -1.61 | 0.043 |
| GOBP_LIPID_STORAGE | -1.61 | 0.043 |
| GOBP_DOPAMINE_TRANSPORT | -1.61 | 0.043 |
| GOBP_POSITIVE_REGULATION_OF_CHONDROCYTE_DIFFERENTIATION | -1.61 | 0.044 |
| GOBP_REGULATION_OF_LEUKOCYTE_DEGRANULATION | -1.61 | 0.044 |
| GOBP_REGULATED_EXOCYTOSIS | -1.61 | 0.044 |
| GOBP_REGULATION_OF_CELLULAR_COMPONENT_SIZE | -1.61 | 0.044 |
| GOMF_STEROID_BINDING | -1.61 | 0.044 |
| GOMF_PHOSPHOLIPASE_BINDING | -1.61 | 0.044 |
| GOBP_PERIPHERAL_NERVOUS_SYSTEM_DEVELOPMENT | -1.61 | 0.044 |
| GOBP_NEPHRON_EPITHELIUM_DEVELOPMENT | -1.61 | 0.045 |
| GOBP_REGULATION_OF_B_CELL_MEDIATED_IMMUNITY | -1.61 | 0.045 |
| GOCC_CELL_CELL_JUNCTION | -1.61 | 0.045 |
| GOBP_REGULATION_OF_NEUTROPHIL_CHEMOTAXIS | -1.61 | 0.045 |
| GOBP_RESPONSE_TO_INORGANIC_SUBSTANCE | -1.61 | 0.045 |
| GOBP_LIPID_IMPORT_INTO_CELL | -1.61 | 0.046 |
| GOBP_POSITIVE_REGULATION_OF_STEROL_TRANSPORT | -1.61 | 0.046 |
| GOBP_POSITIVE_REGULATION_OF_IMMUNE_EFFECTOR_PROCESS | -1.61 | 0.046 |
| GOBP_POSITIVE_REGULATION_OF_RESPONSE_TO_WOUNDING | -1.61 | 0.046 |
| GOBP_B_CELL_HOMEOSTASIS | -1.60 | 0.046 |
| GOMF_STEROID_DEHYDROGENASE_ACTIVITY | -1.60 | 0.046 |
| GOBP_POSITIVE_REGULATION_OF_TRANSPORTER_ACTIVITY | -1.60 | 0.046 |
| GOBP_REGULATION_OF_IMMUNOGLOBULIN_PRODUCTION | -1.60 | 0.046 |
| GOMF_HEXOSYLTRANSFERASE_ACTIVITY | -1.60 | 0.046 |
| GOCC_PLASMA_MEMBRANE_SIGNALING_RECEPTOR_COMPLEX | -1.60 | 0.047 |
| GOBP_REGULATION_OF_LYMPHOCYTE_DIFFERENTIATION | -1.60 | 0.046 |
| GOBP_REGULATION_OF_CELL_FATE_SPECIFICATION | -1.60 | 0.046 |
| GOBP_ORGANIC_ACID_TRANSPORT | -1.60 | 0.047 |
| GOBP_REGULATION_OF_SECRETION | -1.60 | 0.047 |
| GOBP_PEPTIDE_METABOLIC_PROCESS | -1.60 | 0.047 |
| GOBP_SPHINGOLIPID_METABOLIC_PROCESS | -1.60 | 0.047 |
| GOCC_PROTEASOME_CORE_COMPLEX | -1.60 | 0.047 |
| GOBP_NEURON_APOPTOTIC_PROCESS | -1.60 | 0.047 |
| GOBP_SENSORY_ORGAN_DEVELOPMENT | -1.60 | 0.047 |
| GOBP_NEURON_PROJECTION_REGENERATION | -1.60 | 0.047 |
| GOBP_POSITIVE_REGULATION_OF_CARTILAGE_DEVELOPMENT | -1.60 | 0.047 |
| GOMF_LOW_DENSITY_LIPOPROTEIN_PARTICLE_BINDING | -1.60 | 0.047 |
| GOBP_STEROL_BIOSYNTHETIC_PROCESS | -1.60 | 0.047 |
| GOBP_POSITIVE_REGULATION_OF_MAP_KINASE_ACTIVITY | -1.60 | 0.047 |
| GOBP_RESPONSE_TO_CARBOHYDRATE | -1.60 | 0.048 |
| GOBP_RECEPTOR_CATABOLIC_PROCESS | -1.60 | 0.048 |
| GOBP_RESPONSE_TO_INTERLEUKIN_7 | -1.60 | 0.048 |
| GOBP_MAMMARY_GLAND_EPITHELIUM_DEVELOPMENT | -1.60 | 0.048 |
| GOBP_GLYCOPROTEIN_BIOSYNTHETIC_PROCESS | -1.60 | 0.048 |
| GOBP_REGULATION_OF_CALCIUM_MEDIATED_SIGNALING | -1.60 | 0.048 |
| GOBP_RESPONSE_TO_METAL_ION | -1.60 | 0.048 |
| GOBP_POSITIVE_REGULATION_OF_TRANSMEMBRANE_TRANSPORT | -1.60 | 0.048 |
| GOBP_PRODUCTION_OF_MOLECULAR_MEDIATOR_OF_IMMUNE_RESPONSE | -1.60 | 0.048 |
| GOBP_STRIATED_MUSCLE_CELL_PROLIFERATION | -1.59 | 0.049 |
| GOCC_MITOCHONDRIAL_LARGE_RIBOSOMAL_SUBUNIT | -1.59 | 0.049 |
| GOCC_RECEPTOR_COMPLEX | -1.59 | 0.049 |
| GOBP_IMMUNOGLOBULIN_PRODUCTION_INVOLVED_IN_IMMUNOGLOBULIN_MEDIATED_IMMUNE_RESPONSE | -1.59 | 0.049 |
| GOBP_RESPONSE_TO_CAMP | -1.59 | 0.049 |
| GOBP_CHONDROITIN_SULFATE_METABOLIC_PROCESS | -1.59 | 0.049 |
| GOBP_LIPID_LOCALIZATION | -1.59 | 0.049 |
| GOBP_SUPEROXIDE_METABOLIC_PROCESS | -1.59 | 0.049 |
| GOBP_FLUID_TRANSPORT | -1.59 | 0.049 |
| GOBP_INTRACELLULAR_GLUCOSE_HOMEOSTASIS | -1.59 | 0.049 |
| GOBP_NEGATIVE_REGULATION_OF_PROTEIN_CONTAINING_COMPLEX_DISASSEMBLY | -1.59 | 0.049 |
| GOBP_POSITIVE_REGULATION_OF_INTERLEUKIN_6_PRODUCTION | -1.59 | 0.049 |
| GOMF_WNT_RECEPTOR_ACTIVITY | -1.59 | 0.050 |
| GOBP_POSITIVE_REGULATION_OF_WNT_SIGNALING_PATHWAY | -1.59 | 0.050 |
| GOBP_NEGATIVE_REGULATION_OF_SMALL_MOLECULE_METABOLIC_PROCESS | -1.59 | 0.050 |
| GOCC_SYNAPTIC_MEMBRANE | -1.59 | 0.050 |
| GOBP_REGULATION_OF_LEUKOCYTE_MEDIATED_IMMUNITY | -1.59 | 0.050 |
| GOBP_REGULATION_OF_CELLULAR_RESPONSE_TO_VASCULAR_ENDOTHELIAL_GROWTH_FACTOR_STIMULUS | -1.59 | 0.050 |
| GOCC_CELL_CELL_CONTACT_ZONE | -1.59 | 0.050 |
| GOMF_METALLOEXOPEPTIDASE_ACTIVITY | -1.59 | 0.050 |
| GOBP_CHRONIC_INFLAMMATORY_RESPONSE | -1.59 | 0.050 |
| GOBP_INOSITOL_PHOSPHATE_METABOLIC_PROCESS | -1.59 | 0.050 |
| GOBP_NEGATIVE_REGULATION_OF_NEURON_PROJECTION_DEVELOPMENT | -1.59 | 0.050 |
| GOBP_REGULATION_OF_SUPRAMOLECULAR_FIBER_ORGANIZATION | -1.59 | 0.050 |
| GOBP_MORPHOGENESIS_OF_EMBRYONIC_EPITHELIUM | -1.59 | 0.051 |
| GOBP_PHOSPHATIDYLINOSITOL_3_KINASE_PROTEIN_KINASE_B_SIGNAL_TRANSDUCTION | -1.59 | 0.051 |
| GOBP_REGULATION_OF_B_CELL_PROLIFERATION | -1.59 | 0.051 |
| GOBP_T_CELL_DIFFERENTIATION | -1.59 | 0.051 |
| GOBP_REGULATION_OF_KERATINOCYTE_PROLIFERATION | -1.59 | 0.051 |
| GOBP_REGULATION_OF_EPITHELIAL_CELL_DIFFERENTIATION | -1.59 | 0.051 |
| GOBP_CELL_MATURATION | -1.59 | 0.051 |
| GOBP_NEGATIVE_REGULATION_OF_INTERLEUKIN_1_PRODUCTION | -1.59 | 0.051 |
| GOBP_REGULATION_OF_MAP_KINASE_ACTIVITY | -1.59 | 0.051 |
| GOBP_EMBRYONIC_SKELETAL_SYSTEM_DEVELOPMENT | -1.58 | 0.051 |
| GOBP_MELANOCYTE_DIFFERENTIATION | -1.58 | 0.051 |
| GOBP_REGULATION_OF_GROWTH | -1.58 | 0.051 |
| GOMF_HEAT_SHOCK_PROTEIN_BINDING | -1.58 | 0.051 |
| GOBP_RESPONSE_TO_STEROID_HORMONE | -1.58 | 0.052 |
| GOBP_LONG_CHAIN_FATTY_ACID_METABOLIC_PROCESS | -1.58 | 0.052 |
| GOBP_EXPORT_ACROSS_PLASMA_MEMBRANE | -1.58 | 0.052 |
| GOBP_RESPONSE_TO_AMINE | -1.58 | 0.052 |
| GOBP_REGULATION_OF_PROTEIN_AUTOPHOSPHORYLATION | -1.58 | 0.052 |
| GOBP_PURINE_CONTAINING_COMPOUND_TRANSMEMBRANE_TRANSPORT | -1.58 | 0.052 |
| GOBP_MAINTENANCE_OF_PROTEIN_LOCATION | -1.58 | 0.052 |
| GOMF_EXOPEPTIDASE_ACTIVITY | -1.58 | 0.052 |
| GOCC_SECRETORY_GRANULE_MEMBRANE | -1.58 | 0.052 |
| GOBP_SPECIFICATION_OF_SYMMETRY | -1.58 | 0.052 |
| GOBP_SENSORY_ORGAN_MORPHOGENESIS | -1.58 | 0.052 |
| GOBP_POSITIVE_REGULATION_OF_MITOTIC_NUCLEAR_DIVISION | -1.58 | 0.052 |
| GOBP_NEGATIVE_REGULATION_OF_ACTIN_FILAMENT_DEPOLYMERIZATION | -1.58 | 0.052 |
| GOBP_ORGANIC_CATION_TRANSPORT | -1.58 | 0.052 |
| GOBP_CELLULAR_RESPONSE_TO_MOLECULE_OF_BACTERIAL_ORIGIN | -1.58 | 0.052 |
| GOBP_NEUTROPHIL_MEDIATED_IMMUNITY | -1.58 | 0.052 |
| GOBP_RESPONSE_TO_INTERLEUKIN_1 | -1.58 | 0.052 |
| GOBP_TRANSLATIONAL_TERMINATION | -1.58 | 0.052 |
| GOMF_NUCLEOTIDE_RECEPTOR_ACTIVITY | -1.58 | 0.053 |
| GOBP_OLFACTORY_LOBE_DEVELOPMENT | -1.58 | 0.053 |
| GOCC_FILOPODIUM_MEMBRANE | -1.58 | 0.053 |
| GOBP_B_CELL_ACTIVATION_INVOLVED_IN_IMMUNE_RESPONSE | -1.58 | 0.053 |
| GOBP_POSITIVE_REGULATION_OF_ALPHA_BETA_T_CELL_PROLIFERATION | -1.58 | 0.053 |
| GOMF_MANNOSE_BINDING | -1.58 | 0.053 |
| GOBP_POSITIVE_REGULATION_OF_OSTEOCLAST_DIFFERENTIATION | -1.58 | 0.053 |
| GOBP_TUBE_FORMATION | -1.58 | 0.053 |
| GOCC_VACUOLAR_MEMBRANE | -1.58 | 0.054 |
| GOBP_BILE_ACID_BIOSYNTHETIC_PROCESS | -1.58 | 0.054 |
| GOBP_ZINC_ION_TRANSPORT | -1.58 | 0.054 |
| GOBP_SODIUM_ION_TRANSPORT | -1.58 | 0.054 |
| GOBP_POSITIVE_REGULATION_OF_EPITHELIAL_TO_MESENCHYMAL_TRANSITION | -1.58 | 0.054 |
| GOBP_REGULATION_OF_DOPAMINE_SECRETION | -1.58 | 0.054 |
| GOBP_RESPONSE_TO_CHEMOKINE | -1.57 | 0.054 |
| GOBP_MEMBRANE_LIPID_METABOLIC_PROCESS | -1.57 | 0.054 |
| GOBP_REGULATION_OF_HUMORAL_IMMUNE_RESPONSE | -1.57 | 0.054 |
| GOBP_RESPONSE_TO_PEPTIDE_HORMONE | -1.57 | 0.054 |
| GOBP_REGULATION_OF_TRANSMEMBRANE_TRANSPORT | -1.57 | 0.054 |
| GOBP_PROTEIN_KINASE_A_SIGNALING | -1.57 | 0.054 |
| GOBP_VASCULAR_ASSOCIATED_SMOOTH_MUSCLE_CONTRACTION | -1.57 | 0.055 |
| GOBP_TOLL_LIKE_RECEPTOR_2_SIGNALING_PATHWAY | -1.57 | 0.055 |
| GOBP_POSITIVE_REGULATION_OF_SPROUTING_ANGIOGENESIS | -1.57 | 0.056 |
| GOBP_EXCITATORY_SYNAPSE_ASSEMBLY | -1.57 | 0.056 |
| GOBP_RESPONSE_TO_PROSTAGLANDIN | -1.57 | 0.056 |
| GOBP_REGULATION_OF_SMOOTHENED_SIGNALING_PATHWAY | -1.57 | 0.056 |
| GOBP_POSITIVE_REGULATION_OF_VASCULAR_PERMEABILITY | -1.57 | 0.056 |
| GOBP_REGULATION_OF_TISSUE_REMODELING | -1.57 | 0.056 |
| GOMF_TUMOR_NECROSIS_FACTOR_RECEPTOR_BINDING | -1.57 | 0.056 |
| GOBP_LONG_CHAIN_FATTY_ACID_TRANSPORT | -1.57 | 0.056 |
| GOBP_PEPTIDE_CATABOLIC_PROCESS | -1.57 | 0.056 |
| GOMF_LOW_DENSITY_LIPOPROTEIN_PARTICLE_RECEPTOR_BINDING | -1.57 | 0.057 |
| GOBP_ORGANIC_ANION_TRANSPORT | -1.57 | 0.057 |
| GOCC_LAMELLIPODIUM_MEMBRANE | -1.57 | 0.057 |
| GOCC_CLATHRIN_COATED_VESICLE_MEMBRANE | -1.57 | 0.057 |
| GOBP_NEGATIVE_REGULATION_OF_INTERLEUKIN_6_PRODUCTION | -1.57 | 0.057 |
| GOBP_HORMONE_METABOLIC_PROCESS | -1.57 | 0.057 |
| GOBP_AMINOGLYCAN_BIOSYNTHETIC_PROCESS | -1.57 | 0.057 |
| GOBP_POSITIVE_REGULATION_OF_CALCIUM_MEDIATED_SIGNALING | -1.57 | 0.058 |
| GOBP_AXONAL_FASCICULATION | -1.56 | 0.058 |
| GOMF_OXYSTEROL_BINDING | -1.56 | 0.058 |
| GOMF_STEROID_DEHYDROGENASE_ACTIVITY_ACTING_ON_THE_CH_OH_GROUP_OF_DONORS_NAD_OR_NADP_AS_ACCEPTOR | -1.56 | 0.058 |
| GOMF_LIPOPROTEIN_PARTICLE_RECEPTOR_BINDING | -1.56 | 0.058 |
| GOBP_DETECTION_OF_MECHANICAL_STIMULUS_INVOLVED_IN_SENSORY_PERCEPTION | -1.56 | 0.057 |
| GOMF_OXIDOREDUCTASE_ACTIVITY_ACTING_ON_THE_CH_CH_GROUP_OF_DONORS | -1.56 | 0.057 |
| GOBP_EMBRYONIC_CRANIAL_SKELETON_MORPHOGENESIS | -1.56 | 0.058 |
| GOBP_SPHINGOLIPID_BIOSYNTHETIC_PROCESS | -1.56 | 0.058 |
| GOCC_GUANYL_NUCLEOTIDE_EXCHANGE_FACTOR_COMPLEX | -1.56 | 0.058 |
| GOBP_CELLULAR_RESPONSE_TO_BIOTIC_STIMULUS | -1.56 | 0.058 |
| GOBP_IMMUNOGLOBULIN_PRODUCTION | -1.56 | 0.058 |
| GOBP_REGULATION_OF_MEMBRANE_DEPOLARIZATION | -1.56 | 0.059 |
| GOBP_POSITIVE_REGULATION_OF_PROTEIN_AUTOPHOSPHORYLATION | -1.56 | 0.059 |
| GOMF_PASSIVE_TRANSMEMBRANE_TRANSPORTER_ACTIVITY | -1.56 | 0.059 |
| GOBP_RESPONSE_TO_LEUKEMIA_INHIBITORY_FACTOR | -1.56 | 0.059 |
| GOBP_MONOCYTE_CHEMOTACTIC_PROTEIN_1_PRODUCTION | -1.56 | 0.059 |
| GOBP_REGULATION_OF_NERVOUS_SYSTEM_DEVELOPMENT | -1.56 | 0.059 |
| GOBP_DOPAMINE_RECEPTOR_SIGNALING_PATHWAY | -1.56 | 0.059 |
| GOBP_SKELETAL_MUSCLE_CELL_PROLIFERATION | -1.56 | 0.059 |
| GOBP_MESODERMAL_CELL_DIFFERENTIATION | -1.56 | 0.060 |
| GOCC_CYTOSOLIC_SMALL_RIBOSOMAL_SUBUNIT | -1.56 | 0.060 |
| GOBP_ISOPRENOID_METABOLIC_PROCESS | -1.56 | 0.060 |
| GOCC_APICAL_PART_OF_CELL | -1.56 | 0.060 |
| GOMF_PHOSPHATIDYLINOSITOL_3_KINASE_BINDING | -1.56 | 0.060 |
| GOBP_EXOCYTOSIS | -1.56 | 0.060 |
| GOBP_POSITIVE_REGULATION_OF_CATION_CHANNEL_ACTIVITY | -1.56 | 0.060 |
| GOBP_POSITIVE_REGULATION_OF_ATP_DEPENDENT_ACTIVITY | -1.56 | 0.060 |
| GOBP_ACTIVATION_OF_PROTEIN_KINASE_B_ACTIVITY | -1.56 | 0.060 |
| GOBP_POSITIVE_REGULATION_OF_STEROID_METABOLIC_PROCESS | -1.56 | 0.061 |
| GOBP_INTERLEUKIN_6_MEDIATED_SIGNALING_PATHWAY | -1.56 | 0.061 |
| GOBP_REGULATION_OF_REGULATED_SECRETORY_PATHWAY | -1.55 | 0.061 |
| GOBP_SKELETAL_MUSCLE_ORGAN_DEVELOPMENT | -1.55 | 0.061 |
| GOBP_CELL_MORPHOGENESIS_INVOLVED_IN_NEURON_DIFFERENTIATION | -1.55 | 0.061 |
| GOBP_REGULATION_OF_FIBROBLAST_APOPTOTIC_PROCESS | -1.55 | 0.061 |
| GOBP_RHYTHMIC_PROCESS | -1.55 | 0.061 |
| GOBP_POSITIVE_REGULATION_OF_CYTOSKELETON_ORGANIZATION | -1.55 | 0.061 |
| GOBP_POSITIVE_REGULATION_OF_ADAPTIVE_IMMUNE_RESPONSE | -1.55 | 0.061 |
| GOBP_WNT_SIGNALING_PATHWAY_PLANAR_CELL_POLARITY_PATHWAY | -1.55 | 0.061 |
| GOBP_REGULATION_OF_ALPHA_BETA_T_CELL_ACTIVATION | -1.55 | 0.061 |
| GOBP_EMBRYO_IMPLANTATION | -1.55 | 0.061 |
| GOBP_FC_RECEPTOR_SIGNALING_PATHWAY | -1.55 | 0.061 |
| GOMF_SPHINGOLIPID_BINDING | -1.55 | 0.061 |
| GOBP_DISRUPTION_OF_ANATOMICAL_STRUCTURE_IN_ANOTHER_ORGANISM | -1.55 | 0.062 |
| GOBP_REGULATION_OF_MEMBRANE_PROTEIN_ECTODOMAIN_PROTEOLYSIS | -1.55 | 0.062 |
| GOMF_CHEMOKINE_RECEPTOR_BINDING | -1.55 | 0.062 |
| GOBP_REGULATION_OF_LIPID_BIOSYNTHETIC_PROCESS | -1.55 | 0.062 |
| GOBP_CELL_JUNCTION_ASSEMBLY | -1.55 | 0.063 |
| GOMF_PROTEIN_TYROSINE_KINASE_ACTIVATOR_ACTIVITY | -1.55 | 0.063 |
| GOBP_NEURAL_TUBE_DEVELOPMENT | -1.55 | 0.063 |
| GOMF_PROTEIN_TYROSINE_KINASE_ACTIVITY | -1.55 | 0.063 |
| GOBP_MAINTENANCE_OF_SYNAPSE_STRUCTURE | -1.55 | 0.063 |
| GOBP_INTEGRIN_MEDIATED_SIGNALING_PATHWAY | -1.55 | 0.063 |
| GOBP_REGULATION_OF_ACTIN_FILAMENT_BASED_PROCESS | -1.55 | 0.063 |
| GOBP_REGULATION_OF_FATTY_ACID_BIOSYNTHETIC_PROCESS | -1.55 | 0.063 |
| GOBP_CD8_POSITIVE_ALPHA_BETA_T_CELL_ACTIVATION | -1.55 | 0.063 |
| GOBP_BODY_MORPHOGENESIS | -1.55 | 0.063 |
| GOBP_PROGRAMMED_CELL_DEATH_INVOLVED_IN_CELL_DEVELOPMENT | -1.55 | 0.063 |
| GOBP_REGULATION_OF_EPITHELIAL_CELL_PROLIFERATION | -1.55 | 0.063 |
| GOBP_SYNAPTIC_MEMBRANE_ADHESION | -1.55 | 0.063 |
| GOBP_CHONDROITIN_SULFATE_BIOSYNTHETIC_PROCESS | -1.55 | 0.063 |
| GOBP_MITOCHONDRIAL_TRANSLATION | -1.55 | 0.063 |
| GOBP_PROTEIN_LOCALIZATION_TO_ENDOPLASMIC_RETICULUM | -1.55 | 0.063 |
| GOBP_ALPHA_BETA_T_CELL_DIFFERENTIATION | -1.55 | 0.063 |
| GOBP_HOMEOSTASIS_OF_NUMBER_OF_CELLS | -1.55 | 0.064 |
| GOBP_STEM_CELL_DEVELOPMENT | -1.55 | 0.064 |
| GOBP_MYELOID_CELL_DIFFERENTIATION | -1.55 | 0.064 |
| GOBP_LIPOPROTEIN_LOCALIZATION | -1.55 | 0.064 |
| GOBP_POSITIVE_REGULATION_OF_BLOOD_PRESSURE | -1.55 | 0.064 |
| GOBP_AMIDE_METABOLIC_PROCESS | -1.55 | 0.064 |
| GOBP_RENAL_TUBULE_DEVELOPMENT | -1.55 | 0.064 |
| GOBP_VACUOLAR_LOCALIZATION | -1.55 | 0.064 |
| GOMF_GLYCOSYLTRANSFERASE_ACTIVITY | -1.55 | 0.064 |
| GOBP_REGULATION_OF_EXTRACELLULAR_MATRIX_ASSEMBLY | -1.55 | 0.064 |
| GOBP_POSITIVE_REGULATION_OF_B_CELL_PROLIFERATION | -1.55 | 0.064 |
| GOBP_NEGATIVE_REGULATION_OF_POTASSIUM_ION_TRANSMEMBRANE_TRANSPORT | -1.54 | 0.064 |
| GOBP_REGULATION_OF_CANONICAL_WNT_SIGNALING_PATHWAY | -1.54 | 0.064 |
| GOBP_TRANSPORT_OF_VIRUS | -1.54 | 0.064 |
| GOBP_CARDIAC_MUSCLE_TISSUE_MORPHOGENESIS | -1.54 | 0.064 |
| GOBP_HEMATOPOIETIC_OR_LYMPHOID_ORGAN_DEVELOPMENT | -1.54 | 0.064 |
| GOMF_G_PROTEIN_COUPLED_RECEPTOR_ACTIVITY | -1.54 | 0.065 |
| GOMF_AMIDE_TRANSMEMBRANE_TRANSPORTER_ACTIVITY | -1.54 | 0.065 |
| GOBP_RESPONSE_TO_ANGIOTENSIN | -1.54 | 0.065 |
| GOBP_REGULATION_OF_RESPONSE_TO_CYTOKINE_STIMULUS | -1.54 | 0.065 |
| GOBP_UROGENITAL_SYSTEM_DEVELOPMENT | -1.54 | 0.065 |
| GOBP_RENAL_FILTRATION | -1.54 | 0.065 |
| GOBP_REGULATION_OF_TRIGLYCERIDE_METABOLIC_PROCESS | -1.54 | 0.065 |
| GOBP_CAMERA_TYPE_EYE_MORPHOGENESIS | -1.54 | 0.065 |
| GOBP_DORSAL_VENTRAL_PATTERN_FORMATION | -1.54 | 0.065 |
| GOBP_AUTONOMIC_NERVOUS_SYSTEM_DEVELOPMENT | -1.54 | 0.065 |
| GOBP_GLYCOPROTEIN_METABOLIC_PROCESS | -1.54 | 0.065 |
| GOBP_POST_ANAL_TAIL_MORPHOGENESIS | -1.54 | 0.065 |
| GOBP_EMBRYONIC_ORGAN_MORPHOGENESIS | -1.54 | 0.066 |
| GOBP_PHOSPHOLIPID_CATABOLIC_PROCESS | -1.54 | 0.065 |
| GOBP_POSITIVE_REGULATION_OF_G1_S_TRANSITION_OF_MITOTIC_CELL_CYCLE | -1.54 | 0.065 |
| GOBP_ACUTE_PHASE_RESPONSE | -1.54 | 0.066 |
| GOBP_RESPONSE_TO_CORTICOSTEROID | -1.54 | 0.066 |
| GOBP_CELLULAR_RESPONSE_TO_STEROL | -1.54 | 0.066 |
| GOBP_NEGATIVE_REGULATION_OF_INTERLEUKIN_12_PRODUCTION | -1.54 | 0.066 |
| GOBP_AMINE_TRANSPORT | -1.54 | 0.066 |
| GOCC_LATE_ENDOSOME_MEMBRANE | -1.54 | 0.066 |
| GOBP_CELLULAR_RESPONSE_TO_HORMONE_STIMULUS | -1.54 | 0.066 |
| GOBP_CYTOKINE_PRODUCTION_INVOLVED_IN_IMMUNE_RESPONSE | -1.54 | 0.066 |
| GOBP_REGULATION_OF_KINASE_ACTIVITY | -1.54 | 0.066 |
| GOBP_SIGNAL_RELEASE | -1.54 | 0.066 |
| GOCC_ENDOPLASMIC_RETICULUM_GOLGI_INTERMEDIATE_COMPARTMENT_MEMBRANE | -1.54 | 0.066 |
| GOCC_NEUROMUSCULAR_JUNCTION | -1.54 | 0.066 |
| GOBP_NEGATIVE_REGULATION_OF_LEUKOCYTE_MEDIATED_IMMUNITY | -1.54 | 0.067 |
| GOBP_CELLULAR_DEFENSE_RESPONSE | -1.54 | 0.067 |
| GOMF_METALLOENDOPEPTIDASE_ACTIVITY | -1.54 | 0.067 |
| GOMF_PDZ_DOMAIN_BINDING | -1.53 | 0.067 |
| GOBP_BIOMINERAL_TISSUE_DEVELOPMENT | -1.53 | 0.067 |
| GOBP_DEVELOPMENTAL_MATURATION | -1.53 | 0.067 |
| GOBP_ALDEHYDE_BIOSYNTHETIC_PROCESS | -1.53 | 0.067 |
| GOBP_NEGATIVE_REGULATION_OF_B_CELL_ACTIVATION | -1.53 | 0.067 |
| GOCC_MONOATOMIC_ION_CHANNEL_COMPLEX | -1.53 | 0.067 |
| GOCC_AZUROPHIL_GRANULE | -1.53 | 0.067 |
| GOBP_CYTOPLASMIC_TRANSLATION | -1.53 | 0.067 |
| GOBP_REGULATION_OF_HORMONE_LEVELS | -1.53 | 0.067 |
| GOBP_BIOLOGICAL_PROCESS_INVOLVED_IN_INTERACTION_WITH_HOST | -1.53 | 0.067 |
| GOBP_MYELOID_LEUKOCYTE_DIFFERENTIATION | -1.53 | 0.068 |
| GOBP_REGULATION_OF_SYNAPSE_ASSEMBLY | -1.53 | 0.068 |
| GOBP_BONE_REMODELING | -1.53 | 0.068 |
| GOMF_PROTEIN_TYROSINE_PHOSPHATASE_ACTIVITY | -1.53 | 0.068 |
| GOBP_FIBROBLAST_GROWTH_FACTOR_RECEPTOR_SIGNALING_PATHWAY | -1.53 | 0.068 |
| GOBP_GLIOGENESIS | -1.53 | 0.068 |
| GOBP_NEGATIVE_REGULATION_OF_INTRACELLULAR_TRANSPORT | -1.53 | 0.068 |
| GOMF_FATTY_ACID_DERIVATIVE_BINDING | -1.53 | 0.068 |
| GOBP_MONOCARBOXYLIC_ACID_TRANSPORT | -1.53 | 0.068 |
| GOBP_NEGATIVE_REGULATION_OF_ACTIN_FILAMENT_POLYMERIZATION | -1.53 | 0.069 |
| GOCC_ENDOPEPTIDASE_COMPLEX | -1.53 | 0.069 |
| GOBP_PROTEOGLYCAN_METABOLIC_PROCESS | -1.53 | 0.069 |
| GOMF_CARBOXYPEPTIDASE_ACTIVITY | -1.53 | 0.069 |
| GOBP_CELL_SURFACE_RECEPTOR_PROTEIN_SERINE_THREONINE_KINASE_SIGNALING_PATHWAY | -1.53 | 0.069 |
| GOBP_ENDOTHELIAL_CELL_CHEMOTAXIS | -1.53 | 0.070 |
| GOBP_REGULATION_OF_WNT_SIGNALING_PATHWAY | -1.53 | 0.070 |
| GOBP_CELLULAR_LIPID_CATABOLIC_PROCESS | -1.53 | 0.070 |
| GOBP_REGULATION_OF_BONE_MINERALIZATION | -1.53 | 0.070 |
| GOBP_POSITIVE_REGULATION_OF_CELL_CYCLE_G1_S_PHASE_TRANSITION | -1.53 | 0.070 |
| GOBP_NEGATIVE_REGULATION_OF_INTRACELLULAR_PROTEIN_TRANSPORT | -1.53 | 0.070 |
| GOBP_POSITIVE_REGULATION_OF_LEUKOCYTE_ADHESION_TO_VASCULAR_ENDOTHELIAL_CELL | -1.53 | 0.070 |
| GOBP_POSITIVE_REGULATION_OF_CELL_SUBSTRATE_ADHESION | -1.53 | 0.070 |
| GOBP_ACID_SECRETION | -1.53 | 0.070 |
| GOBP_RESPONSE_TO_OXIDATIVE_STRESS | -1.53 | 0.070 |
| GOBP_MYELOID_CELL_HOMEOSTASIS | -1.53 | 0.070 |
| GOBP_REGULATION_OF_CARDIAC_MUSCLE_CELL_ACTION_POTENTIAL | -1.52 | 0.071 |
| GOBP_CELLULAR_KETONE_METABOLIC_PROCESS | -1.52 | 0.071 |
| GOBP_NEGATIVE_REGULATION_OF_STEROID_METABOLIC_PROCESS | -1.52 | 0.071 |
| GOBP_NUCLEOTIDE_TRANSMEMBRANE_TRANSPORT | -1.52 | 0.071 |
| GOBP_FIBROBLAST_APOPTOTIC_PROCESS | -1.52 | 0.071 |
| GOCC_MYOFILAMENT | -1.52 | 0.071 |
| GOBP_POSITIVE_T_CELL_SELECTION | -1.52 | 0.071 |
| GOBP_REGULATION_OF_NEUROINFLAMMATORY_RESPONSE | -1.52 | 0.071 |
| GOMF_ATPASE_REGULATOR_ACTIVITY | -1.52 | 0.071 |
| GOBP_NEGATIVE_REGULATION_OF_VASCULAR_PERMEABILITY | -1.52 | 0.071 |
| GOBP_REGULATION_OF_MYELOID_CELL_DIFFERENTIATION | -1.52 | 0.071 |
| GOBP_ESTROUS_CYCLE | -1.52 | 0.071 |
| GOBP_DE_NOVO_PROTEIN_FOLDING | -1.52 | 0.071 |
| GOMF_OXIDOREDUCTASE_ACTIVITY_ACTING_ON_THE_CH_CH_GROUP_OF_DONORS_NAD_OR_NADP_AS_ACCEPTOR | -1.52 | 0.071 |
| GOBP_REGULATION_OF_PROTEIN_CONTAINING_COMPLEX_ASSEMBLY | -1.52 | 0.071 |
| GOBP_ANTERIOR_POSTERIOR_AXIS_SPECIFICATION | -1.52 | 0.072 |
| GOMF_RECEPTOR_SERINE_THREONINE_KINASE_BINDING | -1.52 | 0.072 |
| GOBP_NOTCH_SIGNALING_PATHWAY | -1.52 | 0.072 |
| GOBP_REGULATION_OF_LIPID_CATABOLIC_PROCESS | -1.52 | 0.072 |
| GOMF_PROTEIN_CARRIER_CHAPERONE | -1.52 | 0.072 |
| GOBP_PROTEIN_MATURATION | -1.52 | 0.072 |
| GOMF_PROTEIN_FOLDING_CHAPERONE | -1.52 | 0.072 |
| GOBP_REGULATION_OF_CELL_KILLING | -1.52 | 0.072 |
| GOBP_POSITIVE_REGULATION_OF_LEUKOCYTE_MEDIATED_IMMUNITY | -1.52 | 0.072 |
| GOBP_CEREBELLAR_CORTEX_MORPHOGENESIS | -1.52 | 0.072 |
| GOBP_STEROID_HORMONE_SECRETION | -1.52 | 0.072 |
| GOBP_ESTROGEN_METABOLIC_PROCESS | -1.52 | 0.072 |
| GOBP_POSITIVE_REGULATION_OF_CD4_POSITIVE_ALPHA_BETA_T_CELL_ACTIVATION | -1.52 | 0.072 |
| GOBP_RESPONSE_TO_VITAMIN_D | -1.52 | 0.072 |
| GOBP_REGULATION_OF_FIBROBLAST_MIGRATION | -1.52 | 0.072 |
| GOBP_PROSTATE_GLAND_MORPHOGENESIS | -1.52 | 0.072 |
| GOBP_INTRINSIC_APOPTOTIC_SIGNALING_PATHWAY_IN_RESPONSE_TO_OXIDATIVE_STRESS | -1.52 | 0.072 |
| GOBP_XENOBIOTIC_METABOLIC_PROCESS | -1.52 | 0.073 |
| GOBP_REGULATION_OF_REACTIVE_OXYGEN_SPECIES_METABOLIC_PROCESS | -1.52 | 0.073 |
| GOBP_ESTABLISHMENT_OF_CELL_POLARITY | -1.52 | 0.073 |
| GOBP_REGULATION_OF_AMINE_METABOLIC_PROCESS | -1.52 | 0.073 |
| GOBP_POSITIVE_REGULATION_OF_MYOBLAST_DIFFERENTIATION | -1.52 | 0.073 |
| GOBP_CALCIUM_ION_TRANSPORT | -1.52 | 0.073 |
| GOBP_REGULATION_OF_CALCIUM_ION_IMPORT | -1.52 | 0.073 |
| GOBP_NEUROMUSCULAR_SYNAPTIC_TRANSMISSION | -1.52 | 0.073 |
| GOBP_FC_EPSILON_RECEPTOR_SIGNALING_PATHWAY | -1.52 | 0.073 |
| GOBP_POSITIVE_REGULATION_OF_MACROPHAGE_CYTOKINE_PRODUCTION | -1.52 | 0.073 |
| GOMF_FIBRONECTIN_BINDING | -1.52 | 0.073 |
| GOBP_FEAR_RESPONSE | -1.52 | 0.073 |
| GOBP_NEGATIVE_REGULATION_OF_CELL_GROWTH | -1.52 | 0.073 |
| GOMF_METALLOPEPTIDASE_ACTIVITY | -1.51 | 0.074 |
| GOBP_REGULATION_OF_ORGANIC_ACID_TRANSPORT | -1.51 | 0.074 |
| GOBP_RESPONSE_TO_FUNGUS | -1.51 | 0.074 |
| GOBP_P38MAPK_CASCADE | -1.51 | 0.074 |
| GOBP_TROPHOBLAST_CELL_MIGRATION | -1.51 | 0.074 |
| GOBP_REGULATION_OF_OSSIFICATION | -1.51 | 0.074 |
| GOMF_E_BOX_BINDING | -1.51 | 0.074 |
| GOBP_B_CELL_PROLIFERATION | -1.51 | 0.075 |
| GOBP_REGULATION_OF_MICROTUBULE_DEPOLYMERIZATION | -1.51 | 0.075 |
| GOBP_RECEPTOR_METABOLIC_PROCESS | -1.51 | 0.075 |
| GOBP_NEGATIVE_REGULATION_OF_PEPTIDYL_SERINE_PHOSPHORYLATION | -1.51 | 0.075 |
| GOBP_ARACHIDONIC_ACID_METABOLIC_PROCESS | -1.51 | 0.075 |
| GOBP_POLARIZED_EPITHELIAL_CELL_DIFFERENTIATION | -1.51 | 0.075 |
| GOBP_NITRIC_OXIDE_MEDIATED_SIGNAL_TRANSDUCTION | -1.51 | 0.075 |
| GOBP_POSITIVE_REGULATION_OF_LIPASE_ACTIVITY | -1.51 | 0.075 |
| GOCC_CYTOSOLIC_LARGE_RIBOSOMAL_SUBUNIT | -1.51 | 0.075 |
| GOMF_CHEMOKINE_ACTIVITY | -1.51 | 0.075 |
| GOBP_POSITIVE_REGULATION_OF_NERVOUS_SYSTEM_DEVELOPMENT | -1.51 | 0.076 |
| GOBP_ACYLGLYCEROL_HOMEOSTASIS | -1.51 | 0.076 |
| GOBP_DEVELOPMENTAL_GROWTH_INVOLVED_IN_MORPHOGENESIS | -1.51 | 0.076 |
| GOBP_T_CELL_DIFFERENTIATION_INVOLVED_IN_IMMUNE_RESPONSE | -1.51 | 0.076 |
| GOBP_CELLULAR_RESPONSE_TO_CARBOHYDRATE_STIMULUS | -1.51 | 0.076 |
| GOBP_ANTIGEN_PROCESSING_AND_PRESENTATION_OF_EXOGENOUS_PEPTIDE_ANTIGEN_VIA_MHC_CLASS_II | -1.51 | 0.076 |
| GOBP_LOCOMOTORY_BEHAVIOR | -1.51 | 0.076 |
| GOBP_DICARBOXYLIC_ACID_METABOLIC_PROCESS | -1.51 | 0.076 |
| GOBP_POSITIVE_REGULATION_OF_CYTOKINE_PRODUCTION_INVOLVED_IN_IMMUNE_RESPONSE | -1.51 | 0.076 |
| GOBP_NEGATIVE_REGULATION_OF_NATURAL_KILLER_CELL_MEDIATED_IMMUNITY | -1.51 | 0.077 |
| GOBP_SMOOTHENED_SIGNALING_PATHWAY | -1.51 | 0.076 |
| GOBP_NEURAL_PRECURSOR_CELL_PROLIFERATION | -1.51 | 0.076 |
| GOMF_NOTCH_BINDING | -1.51 | 0.077 |
| GOCC_FICOLIN_1_RICH_GRANULE_LUMEN | -1.51 | 0.077 |
| GOBP_POSITIVE_REGULATION_OF_BLOOD_VESSEL_ENDOTHELIAL_CELL_MIGRATION | -1.51 | 0.077 |
| GOMF_ENDOPEPTIDASE_ACTIVITY | -1.51 | 0.077 |
| GOBP_VASCULAR_ENDOTHELIAL_GROWTH_FACTOR_SIGNALING_PATHWAY | -1.51 | 0.077 |
| GOBP_DETECTION_OF_MECHANICAL_STIMULUS | -1.51 | 0.077 |
| GOBP_REGULATION_OF_MYOBLAST_FUSION | -1.51 | 0.077 |
| GOBP_REGULATION_OF_CELL_GROWTH | -1.51 | 0.077 |
| GOBP_REGULATION_OF_CELLULAR_KETONE_METABOLIC_PROCESS | -1.51 | 0.077 |
| GOBP_POSITIVE_REGULATION_OF_PROTEIN_KINASE_ACTIVITY | -1.50 | 0.078 |
| GOBP_CELLULAR_COMPONENT_DISASSEMBLY | -1.50 | 0.078 |
| GOBP_ENDOCRINE_PANCREAS_DEVELOPMENT | -1.50 | 0.078 |
| GOBP_CHAPERONE_MEDIATED_PROTEIN_COMPLEX_ASSEMBLY | -1.50 | 0.078 |
| GOBP_REGULATION_OF_P38MAPK_CASCADE | -1.50 | 0.079 |
| GOBP_REGULATION_OF_COMPLEMENT_ACTIVATION | -1.50 | 0.079 |
| GOBP_REGULATION_OF_SMOOTH_MUSCLE_CELL_DIFFERENTIATION | -1.50 | 0.079 |
| GOBP_CYTOKINE_MEDIATED_SIGNALING_PATHWAY | -1.50 | 0.079 |
| GOBP_POSITIVE_REGULATION_OF_CANONICAL_WNT_SIGNALING_PATHWAY | -1.50 | 0.079 |
| GOBP_VITAMIN_METABOLIC_PROCESS | -1.50 | 0.079 |
| GOBP_SINGLE_FERTILIZATION | -1.50 | 0.079 |
| GOBP_REGULATION_OF_ACTIN_FILAMENT_LENGTH | -1.50 | 0.079 |
| GOMF_O_ACYLTRANSFERASE_ACTIVITY | -1.50 | 0.079 |
| GOCC_VOLTAGE_GATED_CALCIUM_CHANNEL_COMPLEX | -1.50 | 0.079 |
| GOBP_NEGATIVE_REGULATION_OF_CELL_SUBSTRATE_ADHESION | -1.50 | 0.079 |
| GOBP_TEMPERATURE_HOMEOSTASIS | -1.50 | 0.079 |
| GOBP_DEVELOPMENTAL_CELL_GROWTH | -1.50 | 0.080 |
| GOBP_POSITIVE_REGULATION_OF_P38MAPK_CASCADE | -1.50 | 0.080 |
| GOCC_CYTOCHROME_COMPLEX | -1.50 | 0.080 |
| GOMF_MONOATOMIC_ANION_MONOATOMIC_CATION_SYMPORTER_ACTIVITY | -1.50 | 0.080 |
| GOBP_NEGATIVE_REGULATION_OF_AXONOGENESIS | -1.50 | 0.080 |
| GOBP_POSITIVE_REGULATION_OF_INTERLEUKIN_8_PRODUCTION | -1.50 | 0.081 |
| GOBP_NEGATIVE_REGULATION_OF_CELL_DEVELOPMENT | -1.50 | 0.081 |
| GOBP_FERTILIZATION | -1.50 | 0.081 |
| GOBP_SULFUR_COMPOUND_BIOSYNTHETIC_PROCESS | -1.50 | 0.081 |
| GOBP_NEGATIVE_REGULATION_OF_NERVOUS_SYSTEM_DEVELOPMENT | -1.50 | 0.081 |
| GOBP_NEGATIVE_REGULATION_OF_CELL_CELL_ADHESION | -1.50 | 0.081 |
| GOBP_DETOXIFICATION | -1.50 | 0.081 |
| GOBP_REGULATION_OF_MONOCYTE_DIFFERENTIATION | -1.50 | 0.081 |
| GOMF_PHOSPHORIC_ESTER_HYDROLASE_ACTIVITY | -1.50 | 0.081 |
| GOBP_RETINAL_GANGLION_CELL_AXON_GUIDANCE | -1.50 | 0.082 |
| GOCC_TRANSLATION_PREINITIATION_COMPLEX | -1.50 | 0.082 |
| GOBP_CD4_POSITIVE_ALPHA_BETA_T_CELL_DIFFERENTIATION | -1.50 | 0.082 |
| GOBP_NEGATIVE_REGULATION_OF_HORMONE_SECRETION | -1.49 | 0.082 |
| GOBP_NEGATIVE_REGULATION_OF_PROTEIN_POLYMERIZATION | -1.49 | 0.082 |
| GOBP_NEGATIVE_REGULATION_OF_FATTY_ACID_BIOSYNTHETIC_PROCESS | -1.49 | 0.082 |
| GOBP_EMBRYONIC_HEART_TUBE_MORPHOGENESIS | -1.49 | 0.082 |
| GOMF_MRNA_5_UTR_BINDING | -1.49 | 0.083 |
| GOBP_REGULATION_OF_PEPTIDYL_SERINE_PHOSPHORYLATION | -1.49 | 0.083 |
| GOMF_HYDROLASE_ACTIVITY_ACTING_ON_GLYCOSYL_BONDS | -1.49 | 0.083 |
| GOBP_REGULATION_OF_RESPONSE_TO_OXIDATIVE_STRESS | -1.49 | 0.083 |
| GOBP_RESPONSE_TO_REACTIVE_OXYGEN_SPECIES | -1.49 | 0.083 |
| GOBP_MATING_BEHAVIOR | -1.49 | 0.083 |
| GOCC_COP9_SIGNALOSOME | -1.49 | 0.083 |
| GOBP_REGULATION_OF_ADAPTIVE_IMMUNE_RESPONSE | -1.49 | 0.083 |
| GOBP_CALCIUM_ION_IMPORT | -1.49 | 0.083 |
| GOBP_VASCULOGENESIS | -1.49 | 0.083 |
| GOCC_EARLY_ENDOSOME_MEMBRANE | -1.49 | 0.083 |
| GOCC_POSTSYNAPTIC_MEMBRANE | -1.49 | 0.084 |
| GOBP_REGULATION_OF_MUSCLE_ADAPTATION | -1.49 | 0.084 |
| GOBP_POSITIVE_REGULATION_OF_TRANSMEMBRANE_RECEPTOR_PROTEIN_SERINE_THREONINE_KINASE_SIGNALING_PATHWAY | -1.49 | 0.084 |
| GOBP_EAR_DEVELOPMENT | -1.49 | 0.084 |
| GOBP_POSITIVE_REGULATION_OF_NON_CANONICAL_NF_KAPPAB_SIGNAL_TRANSDUCTION | -1.49 | 0.084 |
| GOMF_METAL_ION_TRANSMEMBRANE_TRANSPORTER_ACTIVITY | -1.49 | 0.084 |
| GOBP_RESPONSE_TO_TESTOSTERONE | -1.49 | 0.084 |
| GOBP_LEUKOCYTE_MEDIATED_CYTOTOXICITY | -1.49 | 0.084 |
| GOBP_AXIS_SPECIFICATION | -1.49 | 0.084 |
| GOBP_SMOOTH_MUSCLE_TISSUE_DEVELOPMENT | -1.49 | 0.084 |
| GOBP_BIOLOGICAL_PROCESS_INVOLVED_IN_SYMBIOTIC_INTERACTION | -1.49 | 0.084 |
| GOBP_MEMBRANE_LIPID_BIOSYNTHETIC_PROCESS | -1.49 | 0.084 |
| GOBP_NEGATIVE_REGULATION_OF_LIPID_TRANSPORT | -1.49 | 0.085 |
| GOMF_ALCOHOL_BINDING | -1.49 | 0.085 |
| GOBP_POSITIVE_REGULATION_OF_FAT_CELL_DIFFERENTIATION | -1.49 | 0.085 |
| GOBP_RESPONSE_TO_INTERLEUKIN_17 | -1.49 | 0.085 |
| GOBP_RENAL_SYSTEM_PROCESS | -1.49 | 0.085 |
| GOBP_ASTROCYTE_DEVELOPMENT | -1.49 | 0.085 |
| GOBP_FEMALE_SEX_DIFFERENTIATION | -1.49 | 0.085 |
| GOBP_POSITIVE_REGULATION_OF_VIRAL_PROCESS | -1.49 | 0.085 |
| GOBP_ADENYLATE_CYCLASE_ACTIVATING_ADRENERGIC_RECEPTOR_SIGNALING_PATHWAY | -1.48 | 0.086 |
| GOBP_GLYCOSPHINGOLIPID_METABOLIC_PROCESS | -1.48 | 0.086 |
| GOBP_EPITHELIAL_CELL_DEVELOPMENT | -1.48 | 0.086 |
| GOBP_REACTIVE_OXYGEN_SPECIES_METABOLIC_PROCESS | -1.48 | 0.087 |
| GOMF_PHOSPHORUS_OXYGEN_LYASE_ACTIVITY | -1.48 | 0.087 |
| GOBP_MAST_CELL_ACTIVATION | -1.48 | 0.087 |
| GOBP_RESPONSE_TO_ACTIVITY | -1.48 | 0.087 |
| GOBP_CRANIOFACIAL_SUTURE_MORPHOGENESIS | -1.48 | 0.087 |
| GOBP_NEGATIVE_REGULATION_OF_GROWTH | -1.48 | 0.087 |
| GOBP_ACTIN_POLYMERIZATION_OR_DEPOLYMERIZATION | -1.48 | 0.088 |
| GOBP_CARDIAC_MUSCLE_CELL_PROLIFERATION | -1.48 | 0.088 |
| GOBP_NEGATIVE_REGULATION_OF_PROTEIN_CONTAINING_COMPLEX_ASSEMBLY | -1.48 | 0.088 |
| GOBP_REGULATION_OF_ISOTYPE_SWITCHING | -1.48 | 0.088 |
| GOBP_DETECTION_OF_TEMPERATURE_STIMULUS | -1.48 | 0.088 |
| GOBP_DOPAMINE_SECRETION | -1.48 | 0.088 |
| GOBP_CD4_POSITIVE_ALPHA_BETA_T_CELL_CYTOKINE_PRODUCTION | -1.48 | 0.088 |
| GOBP_TRANSFORMING_GROWTH_FACTOR_BETA_PRODUCTION | -1.48 | 0.088 |
| GOBP_PYROPTOSIS | -1.48 | 0.088 |
| GOBP_REGULATION_OF_EXOCYTOSIS | -1.48 | 0.088 |
| GOCC_LATE_ENDOSOME | -1.48 | 0.088 |
| GOBP_POSITIVE_REGULATION_OF_CYSTEINE_TYPE_ENDOPEPTIDASE_ACTIVITY | -1.48 | 0.088 |
| GOBP_REGULATION_OF_DEVELOPMENTAL_GROWTH | -1.48 | 0.088 |
| GOBP_CALCIUM_ION_TRANSMEMBRANE_TRANSPORT | -1.48 | 0.088 |
| GOBP_ESTABLISHMENT_OF_PROTEIN_LOCALIZATION_TO_ENDOPLASMIC_RETICULUM | -1.48 | 0.089 |
| GOMF_BETA_CATENIN_BINDING | -1.48 | 0.089 |
| GOBP_REGULATION_OF_TRANSMEMBRANE_RECEPTOR_PROTEIN_SERINE_THREONINE_KINASE_SIGNALING_PATHWAY | -1.48 | 0.089 |
| GOMF_PHOSPHATASE_ACTIVITY | -1.48 | 0.089 |
| GOBP_NADP_METABOLIC_PROCESS | -1.48 | 0.089 |
| GOBP_FATTY_ACID_DERIVATIVE_BIOSYNTHETIC_PROCESS | -1.48 | 0.089 |
| GOBP_CELLULAR_RESPONSE_TO_REACTIVE_OXYGEN_SPECIES | -1.48 | 0.089 |
| GOBP_REGULATION_OF_INTEGRIN_MEDIATED_SIGNALING_PATHWAY | -1.48 | 0.089 |
| GOBP_REGULATION_OF_CARTILAGE_DEVELOPMENT | -1.48 | 0.089 |
| GOBP_VASOCONSTRICTION | -1.48 | 0.089 |
| GOBP_CELL_COMMUNICATION_BY_ELECTRICAL_COUPLING_INVOLVED_IN_CARDIAC_CONDUCTION | -1.48 | 0.089 |
| GOBP_CYTOPLASMIC_TRANSLATIONAL_INITIATION | -1.48 | 0.089 |
| GOBP_POSITIVE_REGULATION_OF_MICROTUBULE_POLYMERIZATION_OR_DEPOLYMERIZATION | -1.48 | 0.089 |
| GOBP_NEGATIVE_REGULATION_OF_CYTOSKELETON_ORGANIZATION | -1.48 | 0.089 |
| GOBP_POSITIVE_REGULATION_OF_OSSIFICATION | -1.48 | 0.090 |
| GOBP_POSITIVE_REGULATION_OF_ALPHA_BETA_T_CELL_DIFFERENTIATION | -1.48 | 0.090 |
| GOBP_REGULATION_OF_BIOMINERAL_TISSUE_DEVELOPMENT | -1.48 | 0.090 |
| GOBP_REGULATION_OF_AXON_EXTENSION_INVOLVED_IN_AXON_GUIDANCE | -1.48 | 0.090 |
| GOBP_NEGATIVE_REGULATION_OF_LIPID_LOCALIZATION | -1.48 | 0.090 |
| GOBP_AMIDE_TRANSPORT | -1.47 | 0.090 |
| GOBP_RESPONSE_TO_PROGESTERONE | -1.47 | 0.090 |
| GOBP_NEGATIVE_REGULATION_OF_MICROTUBULE_POLYMERIZATION_OR_DEPOLYMERIZATION | -1.47 | 0.090 |
| GOBP_MAMMARY_GLAND_EPITHELIAL_CELL_DIFFERENTIATION | -1.47 | 0.090 |
| GOBP_MAINTENANCE_OF_LOCATION_IN_CELL | -1.47 | 0.090 |
| GOBP_FACE_DEVELOPMENT | -1.47 | 0.090 |
| GOMF_CYTOKINE_RECEPTOR_ACTIVITY | -1.47 | 0.090 |
| GOBP_REGULATION_OF_TRANSPORTER_ACTIVITY | -1.47 | 0.090 |
| GOBP_POSITIVE_REGULATION_OF_CELLULAR_COMPONENT_BIOGENESIS | -1.47 | 0.090 |
| GOBP_SYNAPTIC_TRANSMISSION_GABAERGIC | -1.47 | 0.090 |
| GOBP_RESPONSE_TO_ISCHEMIA | -1.47 | 0.091 |
| GOBP_REGULATION_OF_BONE_REMODELING | -1.47 | 0.091 |
| GOBP_RESPONSE_TO_ALKALOID | -1.47 | 0.091 |
| GOMF_SMAD_BINDING | -1.47 | 0.091 |
| GOBP_REGULATION_OF_POLYSACCHARIDE_BIOSYNTHETIC_PROCESS | -1.47 | 0.091 |
| GOBP_REGULATION_OF_OXIDATIVE_STRESS_INDUCED_INTRINSIC_APOPTOTIC_SIGNALING_PATHWAY | -1.47 | 0.091 |
| GOMF_ABC_TYPE_TRANSPORTER_ACTIVITY | -1.47 | 0.092 |
| GOBP_REGULATION_OF_ENDOTHELIAL_CELL_CHEMOTAXIS | -1.47 | 0.092 |
| GOBP_NEGATIVE_REGULATION_OF_POTASSIUM_ION_TRANSPORT | -1.47 | 0.092 |
| GOBP_RESPONSE_TO_HYDROPEROXIDE | -1.47 | 0.092 |
| GOCC_PEPTIDASE_COMPLEX | -1.47 | 0.092 |
| GOBP_BONE_DEVELOPMENT | -1.47 | 0.093 |
| GOBP_CELLULAR_COMPONENT_ASSEMBLY_INVOLVED_IN_MORPHOGENESIS | -1.47 | 0.093 |
| GOBP_NEURAL_CREST_CELL_DIFFERENTIATION | -1.47 | 0.093 |
| GOBP_CELLULAR_EXTRAVASATION | -1.47 | 0.093 |
| GOBP_LABYRINTHINE_LAYER_DEVELOPMENT | -1.47 | 0.093 |
| GOBP_REGULATION_OF_NEUROGENESIS | -1.47 | 0.093 |
| GOBP_REGULATION_OF_NEURAL_PRECURSOR_CELL_PROLIFERATION | -1.47 | 0.093 |
| GOBP_REGULATION_OF_ATP_DEPENDENT_ACTIVITY | -1.47 | 0.093 |
| GOBP_GLIAL_CELL_DIFFERENTIATION | -1.47 | 0.093 |
| GOBP_RELAXATION_OF_MUSCLE | -1.47 | 0.093 |
| GOBP_REGULATION_OF_LEUKOCYTE_ADHESION_TO_VASCULAR_ENDOTHELIAL_CELL | -1.47 | 0.093 |
| GOBP_NEGATIVE_REGULATION_OF_B_CELL_PROLIFERATION | -1.47 | 0.093 |
| GOBP_SPHINGOMYELIN_METABOLIC_PROCESS | -1.47 | 0.093 |
| GOMF_NON_MEMBRANE_SPANNING_PROTEIN_TYROSINE_KINASE_ACTIVITY | -1.47 | 0.093 |
| GOBP_REGULATION_OF_GLIAL_CELL_MIGRATION | -1.47 | 0.094 |
| GOBP_MUSCLE_ADAPTATION | -1.47 | 0.094 |
| GOBP_REGULATION_OF_SPROUTING_ANGIOGENESIS | -1.47 | 0.094 |
| GOBP_NEGATIVE_REGULATION_OF_MUSCLE_CONTRACTION | -1.47 | 0.094 |
| GOBP_NEGATIVE_REGULATION_OF_PROTEIN_MODIFICATION_PROCESS | -1.47 | 0.094 |
| GOBP_NEGATIVE_REGULATION_OF_LIPID_BIOSYNTHETIC_PROCESS | -1.46 | 0.094 |
| GOBP_NEGATIVE_REGULATION_OF_STEM_CELL_PROLIFERATION | -1.46 | 0.094 |
| GOMF_LIPOPROTEIN_PARTICLE_RECEPTOR_ACTIVITY | -1.46 | 0.095 |
| GOBP_FACE_MORPHOGENESIS | -1.46 | 0.095 |
| GOBP_PROTEIN_FOLDING | -1.46 | 0.095 |
| GOBP_DETECTION_OF_STIMULUS_INVOLVED_IN_SENSORY_PERCEPTION_OF_PAIN | -1.46 | 0.096 |
| GOBP_NEGATIVE_REGULATION_OF_CANONICAL_WNT_SIGNALING_PATHWAY | -1.46 | 0.096 |
| GOBP_HEAD_MORPHOGENESIS | -1.46 | 0.096 |
| GOBP_STEROL_METABOLIC_PROCESS | -1.46 | 0.096 |
| GOBP_REGULATION_OF_CARDIAC_MUSCLE_CONTRACTION | -1.46 | 0.096 |
| GOMF_SODIUM_ION_TRANSMEMBRANE_TRANSPORTER_ACTIVITY | -1.46 | 0.096 |
| GOCC_EUKARYOTIC_48S_PREINITIATION_COMPLEX | -1.46 | 0.096 |
| GOBP_TYPE_B_PANCREATIC_CELL_DIFFERENTIATION | -1.46 | 0.096 |
| GOBP_GAMMA_AMINOBUTYRIC_ACID_SIGNALING_PATHWAY | -1.46 | 0.096 |
| GOMF_HISTONE_KINASE_ACTIVITY | -1.46 | 0.096 |
| GOBP_INNATE_IMMUNE_RESPONSE_ACTIVATING_CELL_SURFACE_RECEPTOR_SIGNALING_PATHWAY | -1.46 | 0.096 |
| GOBP_STORE_OPERATED_CALCIUM_ENTRY | -1.46 | 0.096 |
| GOBP_MYOTUBE_DIFFERENTIATION | -1.46 | 0.097 |
| GOBP_REGULATION_OF_HORMONE_BIOSYNTHETIC_PROCESS | -1.46 | 0.097 |
| GOCC_SODIUM_CHANNEL_COMPLEX | -1.46 | 0.097 |
| GOBP_POSITIVE_REGULATION_OF_NF_KAPPAB_TRANSCRIPTION_FACTOR_ACTIVITY | -1.46 | 0.097 |
| GOBP_REGULATION_OF_GLYCOGEN_BIOSYNTHETIC_PROCESS | -1.46 | 0.097 |
| GOMF_PROTEIN_DISULFIDE_ISOMERASE_ACTIVITY | -1.46 | 0.098 |
| GOBP_REGULATION_OF_CARDIAC_MUSCLE_CONTRACTION_BY_CALCIUM_ION_SIGNALING | -1.46 | 0.098 |
| GOMF_PROTEASE_BINDING | -1.46 | 0.098 |
| GOMF_MAGNESIUM_ION_TRANSMEMBRANE_TRANSPORTER_ACTIVITY | -1.46 | 0.098 |
| GOBP_REGULATION_OF_CELL_JUNCTION_ASSEMBLY | -1.46 | 0.098 |
| GOBP_MULTI_MULTICELLULAR_ORGANISM_PROCESS | -1.46 | 0.098 |
| GOBP_POSITIVE_REGULATION_OF_ISOTYPE_SWITCHING | -1.46 | 0.098 |
| GOBP_MAST_CELL_ACTIVATION_INVOLVED_IN_IMMUNE_RESPONSE | -1.46 | 0.098 |
| GOBP_VENTRICULAR_CARDIAC_MUSCLE_TISSUE_MORPHOGENESIS | -1.46 | 0.099 |
| GOBP_REGULATION_OF_NEURON_MIGRATION | -1.46 | 0.099 |
| GOBP_NEGATIVE_REGULATION_OF_REPRODUCTIVE_PROCESS | -1.46 | 0.099 |
| GOBP_PHOSPHATIDYLCHOLINE_METABOLIC_PROCESS | -1.45 | 0.099 |
| GOBP_CELL_GROWTH | -1.45 | 0.100 |
| GOBP_CELLULAR_RESPONSE_TO_RETINOIC_ACID | -1.45 | 0.100 |
| GOBP_T_CELL_DIFFERENTIATION_IN_THYMUS | -1.45 | 0.100 |
| GOBP_PROTEIN_DEPHOSPHORYLATION | -1.45 | 0.100 |
| GOBP_PURINERGIC_NUCLEOTIDE_RECEPTOR_SIGNALING_PATHWAY | -1.45 | 0.100 |
| GOBP_VASCULAR_ASSOCIATED_SMOOTH_MUSCLE_CELL_DIFFERENTIATION | -1.45 | 0.100 |
| GOBP_NEGATIVE_REGULATION_OF_AXON_EXTENSION | -1.45 | 0.100 |
| GOMF_POLYSACCHARIDE_BINDING | -1.45 | 0.100 |
| GOBP_REGULATION_OF_CHOLESTEROL_BIOSYNTHETIC_PROCESS | -1.45 | 0.100 |
| GOBP_FIBROBLAST_MIGRATION | -1.45 | 0.100 |
| GOBP_NEURONAL_STEM_CELL_POPULATION_MAINTENANCE | -1.45 | 0.100 |
| GOBP_ODONTOBLAST_DIFFERENTIATION | -1.45 | 0.100 |
| GOCC_AZUROPHIL_GRANULE_LUMEN | -1.45 | 0.100 |
| GOBP_PROTEIN_PROCESSING | -1.45 | 0.101 |
| GOMF_ALCOHOL_DEHYDROGENASE_NAD_P_PLUS_ACTIVITY | -1.45 | 0.101 |
| GOBP_MAGNESIUM_ION_TRANSPORT | -1.45 | 0.101 |
| GOBP_VASCULAR_ENDOTHELIAL_CELL_PROLIFERATION | -1.45 | 0.101 |
| GOBP_MUSCLE_ORGAN_MORPHOGENESIS | -1.45 | 0.101 |
| GOBP_EXTRINSIC_APOPTOTIC_SIGNALING_PATHWAY | -1.45 | 0.101 |
| GOBP_REGULATION_OF_EXIT_FROM_MITOSIS | -1.45 | 0.101 |
| GOCC_GLUTAMATERGIC_SYNAPSE | -1.45 | 0.101 |
| GOBP_COLLAGEN_CATABOLIC_PROCESS | -1.45 | 0.101 |
| GOBP_PEPTIDYL_TYROSINE_DEPHOSPHORYLATION | -1.45 | 0.101 |
| GOBP_CELLULAR_RESPONSE_TO_NUTRIENT | -1.45 | 0.101 |
| GOMF_PHOSPHATIDYLINOSITOL_4_5_BISPHOSPHATE_BINDING | -1.45 | 0.102 |
| GOBP_GLUTATHIONE_METABOLIC_PROCESS | -1.45 | 0.102 |
| GOBP_REGULATION_OF_T_CELL_MIGRATION | -1.45 | 0.102 |
| GOBP_ESTABLISHMENT_OR_MAINTENANCE_OF_BIPOLAR_CELL_POLARITY | -1.45 | 0.102 |
| GOBP_KIDNEY_MORPHOGENESIS | -1.45 | 0.102 |
| GOBP_REGULATION_OF_CELLULAR_RESPONSE_TO_TRANSFORMING_GROWTH_FACTOR_BETA_STIMULUS | -1.45 | 0.102 |
| GOBP_NEURAL_TUBE_PATTERNING | -1.45 | 0.102 |
| GOBP_EMBRYONIC_SKELETAL_SYSTEM_MORPHOGENESIS | -1.45 | 0.102 |
| GOBP_TELENCEPHALON_DEVELOPMENT | -1.45 | 0.102 |
| GOBP_T_CELL_CYTOKINE_PRODUCTION | -1.45 | 0.102 |
| GOBP_REGULATION_OF_ACTIN_FILAMENT_ORGANIZATION | -1.45 | 0.103 |
| GOBP_KETONE_BIOSYNTHETIC_PROCESS | -1.45 | 0.102 |
| GOBP_MITOCHONDRIAL_CALCIUM_ION_HOMEOSTASIS | -1.45 | 0.103 |
| GOBP_REGULATION_OF_SYNAPTIC_TRANSMISSION_GLUTAMATERGIC | -1.45 | 0.103 |
| GOCC_ENDOCYTIC_VESICLE_LUMEN | -1.45 | 0.103 |
| GOBP_POSITIVE_REGULATION_OF_CD4_POSITIVE_ALPHA_BETA_T_CELL_DIFFERENTIATION | -1.45 | 0.102 |
| GOBP_NEGATIVE_REGULATION_OF_APOPTOTIC_SIGNALING_PATHWAY | -1.45 | 0.103 |
| GOMF_SOLUTE_MONOATOMIC_CATION_SYMPORTER_ACTIVITY | -1.45 | 0.103 |
| GOMF_HYDROLASE_ACTIVITY_HYDROLYZING_N_GLYCOSYL_COMPOUNDS | -1.45 | 0.103 |
| GOMF_LIGAND_GATED_CALCIUM_CHANNEL_ACTIVITY | -1.45 | 0.103 |
| GOBP_REGULATION_OF_EPITHELIAL_TO_MESENCHYMAL_TRANSITION | -1.45 | 0.103 |
| GOBP_LIPOSACCHARIDE_METABOLIC_PROCESS | -1.45 | 0.103 |
| GOBP_SKIN_EPIDERMIS_DEVELOPMENT | -1.45 | 0.103 |
| GOMF_ATPASE_ACTIVATOR_ACTIVITY | -1.44 | 0.104 |
| GOBP_GLYCOSPHINGOLIPID_BIOSYNTHETIC_PROCESS | -1.44 | 0.103 |
| GOBP_ECTODERM_DEVELOPMENT | -1.44 | 0.104 |
| GOBP_CARBOHYDRATE_METABOLIC_PROCESS | -1.44 | 0.104 |
| GOMF_CARBOHYDRATE_BINDING | -1.44 | 0.104 |
| GOBP_REGULATION_OF_CALCIUM_ION_TRANSPORT | -1.44 | 0.104 |
| GOBP_REGULATION_OF_CD4_POSITIVE_ALPHA_BETA_T_CELL_ACTIVATION | -1.44 | 0.104 |
| GOBP_SECRETION_BY_TISSUE | -1.44 | 0.104 |
| GOBP_RESPONSE_TO_HYDROGEN_PEROXIDE | -1.44 | 0.104 |
| GOBP_REGULATION_OF_PEPTIDASE_ACTIVITY | -1.44 | 0.105 |
| GOCC_MITOCHONDRIAL_SMALL_RIBOSOMAL_SUBUNIT | -1.44 | 0.105 |
| GOBP_ASTROCYTE_DIFFERENTIATION | -1.44 | 0.105 |
| GOBP_PROTEOGLYCAN_BIOSYNTHETIC_PROCESS | -1.44 | 0.105 |
| GOBP_WOUND_HEALING_SPREADING_OF_EPIDERMAL_CELLS | -1.44 | 0.105 |
| GOBP_SIGNALING_RECEPTOR_LIGAND_PRECURSOR_PROCESSING | -1.44 | 0.105 |
| GOBP_CELL_PROLIFERATION_INVOLVED_IN_KIDNEY_DEVELOPMENT | -1.44 | 0.106 |
| GOMF_LAMININ_BINDING | -1.44 | 0.106 |
| GOBP_CIRCADIAN_REGULATION_OF_GENE_EXPRESSION | -1.44 | 0.106 |
| GOMF_ATPASE_COUPLED_TRANSMEMBRANE_TRANSPORTER_ACTIVITY | -1.44 | 0.107 |
| GOBP_RESPONSE_TO_STEROL | -1.44 | 0.107 |
| GOBP_POSITIVE_REGULATION_OF_CALCIUM_ION_TRANSPORT | -1.44 | 0.107 |
| GOMF_MYOSIN_BINDING | -1.44 | 0.107 |
| GOMF_CHOLESTEROL_BINDING | -1.44 | 0.107 |
| GOBP_NEGATIVE_REGULATION_OF_INTERLEUKIN_2_PRODUCTION | -1.44 | 0.107 |
| GOBP_ESTABLISHMENT_OF_EPITHELIAL_CELL_POLARITY | -1.44 | 0.107 |
| GOBP_THYMIC_T_CELL_SELECTION | -1.44 | 0.108 |
| GOBP_PROTEIN_TRANSMEMBRANE_TRANSPORT | -1.44 | 0.108 |
| GOBP_OOCYTE_DIFFERENTIATION | -1.44 | 0.108 |
| GOBP_PROTEIN_CONTAINING_COMPLEX_DISASSEMBLY | -1.44 | 0.108 |
| GOBP_NEGATIVE_REGULATION_OF_FIBROBLAST_GROWTH_FACTOR_RECEPTOR_SIGNALING_PATHWAY | -1.44 | 0.108 |
| GOCC_ACTIN_BASED_CELL_PROJECTION | -1.44 | 0.108 |
| GOBP_POSITIVE_REGULATION_OF_CELL_JUNCTION_ASSEMBLY | -1.44 | 0.108 |
| GOBP_REGULATION_OF_ENDOCRINE_PROCESS | -1.44 | 0.108 |
| GOBP_MALE_SEX_DIFFERENTIATION | -1.44 | 0.109 |
| GOBP_BARBED_END_ACTIN_FILAMENT_CAPPING | -1.43 | 0.109 |
| GOBP_SKIN_DEVELOPMENT | -1.43 | 0.109 |
| GOBP_SKELETAL_SYSTEM_MORPHOGENESIS | -1.43 | 0.109 |
| GOBP_SKELETAL_MUSCLE_TISSUE_REGENERATION | -1.43 | 0.109 |
| GOBP_ORGANELLE_FUSION | -1.43 | 0.109 |
| GOBP_PHOSPHOLIPID_METABOLIC_PROCESS | -1.43 | 0.109 |
| GOBP_AMIDE_BIOSYNTHETIC_PROCESS | -1.43 | 0.109 |
| GOBP_REGULATION_OF_LYMPHOCYTE_MEDIATED_IMMUNITY | -1.43 | 0.109 |
| GOBP_RESPONSE_TO_TOPOLOGICALLY_INCORRECT_PROTEIN | -1.43 | 0.109 |
| GOBP_SULFUR_COMPOUND_METABOLIC_PROCESS | -1.43 | 0.109 |
| GOBP_EMBRYONIC_EYE_MORPHOGENESIS | -1.43 | 0.110 |
| GOCC_DENDRITIC_TREE | -1.43 | 0.110 |
| GOCC_TRANSPORTER_COMPLEX | -1.43 | 0.110 |
| GOBP_SPLEEN_DEVELOPMENT | -1.43 | 0.110 |
| GOBP_MYELOID_CELL_APOPTOTIC_PROCESS | -1.43 | 0.110 |
| GOBP_POSITIVE_REGULATION_OF_BLOOD_CIRCULATION | -1.43 | 0.110 |
| GOBP_REGULATION_OF_CATION_CHANNEL_ACTIVITY | -1.43 | 0.110 |
| GOBP_RNA_DECAPPING | -1.43 | 0.110 |
| GOBP_REGULATION_OF_CATECHOLAMINE_METABOLIC_PROCESS | -1.43 | 0.110 |
| GOCC_AZUROPHIL_GRANULE_MEMBRANE | -1.43 | 0.111 |
| GOBP_POSITIVE_REGULATION_OF_PROTEIN_SERINE_THREONINE_KINASE_ACTIVITY | -1.43 | 0.111 |
| GOBP_NEGATIVE_REGULATION_OF_BIOMINERAL_TISSUE_DEVELOPMENT | -1.43 | 0.111 |
| GOBP_LIPOPOLYSACCHARIDE_MEDIATED_SIGNALING_PATHWAY | -1.43 | 0.112 |
| GOBP_POSITIVE_REGULATION_OF_CANONICAL_NF_KAPPAB_SIGNAL_TRANSDUCTION | -1.43 | 0.112 |
| GOBP_POSITIVE_REGULATION_OF_DEPHOSPHORYLATION | -1.43 | 0.112 |
| GOMF_INTRACELLULARLY_GATED_CALCIUM_CHANNEL_ACTIVITY | -1.43 | 0.112 |
| GOBP_NEGATIVE_REGULATION_OF_WNT_SIGNALING_PATHWAY | -1.43 | 0.112 |
| GOBP_NEGATIVE_REGULATION_OF_PHOSPHORYLATION | -1.43 | 0.112 |
| GOBP_RENAL_TUBULAR_SECRETION | -1.43 | 0.112 |
| GOBP_POSITIVE_REGULATION_OF_MYELOID_LEUKOCYTE_CYTOKINE_PRODUCTION_INVOLVED_IN_IMMUNE_RESPONSE | -1.43 | 0.112 |
| GOBP_NEGATIVE_REGULATION_OF_INTERLEUKIN_1_BETA_PRODUCTION | -1.43 | 0.112 |
| GOBP_CELLULAR_RESPONSE_TO_CHOLESTEROL | -1.43 | 0.113 |
| GOMF_ACTIVE_TRANSMEMBRANE_TRANSPORTER_ACTIVITY | -1.43 | 0.113 |
| GOBP_NEGATIVE_REGULATION_OF_DNA_BINDING_TRANSCRIPTION_FACTOR_ACTIVITY | -1.43 | 0.113 |
| GOBP_REGULATION_OF_NEURON_APOPTOTIC_PROCESS | -1.43 | 0.113 |
| GOBP_POSITIVE_REGULATION_OF_GROWTH | -1.43 | 0.113 |
| GOBP_PALLIUM_DEVELOPMENT | -1.43 | 0.113 |
| GOMF_G_PROTEIN_COUPLED_AMINE_RECEPTOR_ACTIVITY | -1.43 | 0.113 |
| GOBP_GLANDULAR_EPITHELIAL_CELL_DEVELOPMENT | -1.43 | 0.113 |
| GOBP_DEFENSE_RESPONSE_TO_FUNGUS | -1.43 | 0.113 |
| GOBP_POSITIVE_REGULATION_OF_SODIUM_ION_TRANSPORT | -1.43 | 0.114 |
| GOBP_STEROL_HOMEOSTASIS | -1.42 | 0.114 |
| GOBP_DENDRITIC_CELL_MIGRATION | -1.42 | 0.114 |
| GOCC_AXON | -1.42 | 0.114 |
| GOMF_PROTEIN_KINASE_C_BINDING | -1.42 | 0.115 |
| GOMF_PHOSPHOTRANSFERASE_ACTIVITY_FOR_OTHER_SUBSTITUTED_PHOSPHATE_GROUPS | -1.42 | 0.115 |
| GOBP_CELL_MIGRATION_INVOLVED_IN_SPROUTING_ANGIOGENESIS | -1.42 | 0.115 |
| GOBP_NEGATIVE_REGULATION_OF_LYMPHOCYTE_ACTIVATION | -1.42 | 0.115 |
| GOBP_NON_CANONICAL_WNT_SIGNALING_PATHWAY | -1.42 | 0.116 |
| GOMF_OXIDOREDUCTASE_ACTIVITY_ACTING_ON_CH_OH_GROUP_OF_DONORS | -1.42 | 0.116 |
| GOBP_NEGATIVE_REGULATION_OF_CHEMOKINE_PRODUCTION | -1.42 | 0.116 |
| GOBP_REGULATION_OF_LYMPHOCYTE_MIGRATION | -1.42 | 0.116 |
| GOMF_POTASSIUM_CHANNEL_REGULATOR_ACTIVITY | -1.42 | 0.116 |
| GOBP_RESPONSE_TO_MUSCLE_ACTIVITY | -1.42 | 0.116 |
| GOBP_MESODERM_DEVELOPMENT | -1.42 | 0.117 |
| GOMF_MONOCARBOXYLIC_ACID_TRANSMEMBRANE_TRANSPORTER_ACTIVITY | -1.42 | 0.117 |
| GOBP_ENTEROENDOCRINE_CELL_DIFFERENTIATION | -1.42 | 0.117 |
| GOBP_REGULATION_OF_NEURON_DIFFERENTIATION | -1.42 | 0.117 |
| GOBP_NEGATIVE_REGULATION_OF_DEVELOPMENTAL_GROWTH | -1.42 | 0.117 |
| GOBP_METANEPHROS_MORPHOGENESIS | -1.42 | 0.117 |
| GOBP_INCLUSION_BODY_ASSEMBLY | -1.42 | 0.117 |
| GOBP_MORPHOGENESIS_OF_AN_ENDOTHELIUM | -1.42 | 0.117 |
| GOMF_INTRACELLULARLY_LIGAND_GATED_MONOATOMIC_ION_CHANNEL_ACTIVITY | -1.42 | 0.117 |
| GOBP_REGULATION_OF_SODIUM_ION_TRANSPORT | -1.42 | 0.117 |
| GOBP_POSITIVE_REGULATION_OF_CELLULAR_RESPONSE_TO_TRANSFORMING_GROWTH_FACTOR_BETA_STIMULUS | -1.42 | 0.117 |
| GOBP_PROTEIN_REFOLDING | -1.42 | 0.118 |
| GOMF_TRANSLATION_INITIATION_FACTOR_ACTIVITY | -1.42 | 0.118 |
| GOMF_ORGANIC_ANION_TRANSMEMBRANE_TRANSPORTER_ACTIVITY | -1.42 | 0.118 |
| GOMF_FATTY_ACID_BINDING | -1.42 | 0.118 |
| GOBP_ENDOCRINE_HORMONE_SECRETION | -1.42 | 0.118 |
| GOBP_ANTIMICROBIAL_HUMORAL_RESPONSE | -1.42 | 0.118 |
| GOBP_ORGANELLE_MEMBRANE_FUSION | -1.42 | 0.119 |
| GOBP_CELLULAR_RESPONSE_TO_ABIOTIC_STIMULUS | -1.42 | 0.119 |
| GOBP_NEGATIVE_REGULATION_OF_PHOSPHORUS_METABOLIC_PROCESS | -1.42 | 0.119 |
| GOBP_DIGESTION | -1.42 | 0.120 |
| GOBP_CELLULAR_RESPONSE_TO_ANGIOTENSIN | -1.41 | 0.120 |
| GOBP_POSITIVE_REGULATION_OF_TRANSCRIPTION_REGULATORY_REGION_DNA_BINDING | -1.41 | 0.120 |
| GOBP_MANNOSYLATION | -1.41 | 0.121 |
| GOBP_POSITIVE_REGULATION_OF_BONE_MINERALIZATION | -1.41 | 0.121 |
| GOBP_HEAT_GENERATION | -1.41 | 0.121 |
| GOBP_REGULATION_OF_PROTEIN_SERINE_THREONINE_KINASE_ACTIVITY | -1.41 | 0.121 |
| GOBP_REGULATION_OF_CARDIAC_CONDUCTION | -1.41 | 0.122 |
| GOCC_PERIKARYON | -1.41 | 0.122 |
| GOMF_PHOSPHOPROTEIN_PHOSPHATASE_ACTIVITY | -1.41 | 0.122 |
| GOBP_NEUROTRANSMITTER_TRANSPORT | -1.41 | 0.122 |
| GOBP_ORGANIC_ACID_TRANSMEMBRANE_TRANSPORT | -1.41 | 0.122 |
| GOBP_MESODERM_MORPHOGENESIS | -1.41 | 0.122 |
| GOCC_CORTICAL_CYTOSKELETON | -1.41 | 0.122 |
| GOMF_MOLECULAR_SEQUESTERING_ACTIVITY | -1.41 | 0.122 |
| GOMF_ENDOPEPTIDASE_REGULATOR_ACTIVITY | -1.41 | 0.122 |
| GOBP_POSITIVE_REGULATION_OF_SECRETION | -1.41 | 0.122 |
| GOBP_REGULATION_OF_MYELOID_LEUKOCYTE_DIFFERENTIATION | -1.41 | 0.122 |
| GOBP_REGULATION_OF_PROTEOLYSIS | -1.41 | 0.123 |
| GOMF_TRANSLATION_FACTOR_ACTIVITY_RNA_BINDING | -1.41 | 0.123 |
| GOCC_MICROBODY_MEMBRANE | -1.41 | 0.123 |
| GOCC_EUKARYOTIC_TRANSLATION_INITIATION_FACTOR_3_COMPLEX | -1.41 | 0.123 |
| GOBP_REACTIVE_OXYGEN_SPECIES_BIOSYNTHETIC_PROCESS | -1.41 | 0.123 |
| GOBP_CELLULAR_RESPONSE_TO_INTERLEUKIN_1 | -1.41 | 0.123 |
| GOBP_SENSORY_PERCEPTION_OF_PAIN | -1.41 | 0.123 |
| GOBP_ORGANOPHOSPHATE_CATABOLIC_PROCESS | -1.41 | 0.123 |
| GOMF_PROTEIN_TYROSINE_SERINE_THREONINE_PHOSPHATASE_ACTIVITY | -1.41 | 0.123 |
| GOBP_INTRACELLULAR_ZINC_ION_HOMEOSTASIS | -1.41 | 0.123 |
| GOCC_GOLGI_CISTERNA_MEMBRANE | -1.41 | 0.124 |
| GOBP_INORGANIC_ANION_TRANSPORT | -1.41 | 0.124 |
| GOBP_POSITIVE_REGULATION_OF_CELL_DIVISION | -1.41 | 0.124 |
| GOBP_CARDIAC_EPITHELIAL_TO_MESENCHYMAL_TRANSITION | -1.41 | 0.124 |
| GOBP_POSITIVE_REGULATION_OF_SMOOTHENED_SIGNALING_PATHWAY | -1.41 | 0.124 |
| GOBP_RELEASE_OF_SEQUESTERED_CALCIUM_ION_INTO_CYTOSOL_BY_ENDOPLASMIC_RETICULUM | -1.41 | 0.124 |
| GOBP_REGULATION_OF_CELL_FATE_COMMITMENT | -1.41 | 0.125 |
| GOMF_PHOSPHATIDYLINOSITOL_BISPHOSPHATE_BINDING | -1.41 | 0.125 |
| GOBP_REGULATION_OF_DIGESTIVE_SYSTEM_PROCESS | -1.41 | 0.125 |
| GOBP_NEGATIVE_REGULATION_OF_EPITHELIAL_CELL_PROLIFERATION | -1.41 | 0.125 |
| GOBP_POSITIVE_REGULATION_OF_DNA_BIOSYNTHETIC_PROCESS | -1.41 | 0.125 |
| GOBP_MUSCLE_CELL_APOPTOTIC_PROCESS | -1.40 | 0.125 |
| GOBP_POSITIVE_REGULATION_OF_LEUKOCYTE_DEGRANULATION | -1.40 | 0.126 |
| GOMF_CERAMIDE_BINDING | -1.40 | 0.126 |
| GOBP_SPROUTING_ANGIOGENESIS | -1.40 | 0.126 |
| GOBP_CARBOHYDRATE_HOMEOSTASIS | -1.40 | 0.126 |
| GOBP_NEGATIVE_REGULATION_OF_CATALYTIC_ACTIVITY | -1.40 | 0.126 |
| GOMF_TRANSCRIPTION_REGULATOR_INHIBITOR_ACTIVITY | -1.40 | 0.126 |
| GOBP_MYELOID_LEUKOCYTE_CYTOKINE_PRODUCTION | -1.40 | 0.126 |
| GOBP_SOMATIC_DIVERSIFICATION_OF_IMMUNOGLOBULINS | -1.40 | 0.126 |
| GOBP_MOTOR_NEURON_AXON_GUIDANCE | -1.40 | 0.127 |
| GOMF_ORGANIC_CATION_TRANSMEMBRANE_TRANSPORTER_ACTIVITY | -1.40 | 0.127 |
| GOBP_REGULATION_OF_TRANS_SYNAPTIC_SIGNALING | -1.40 | 0.127 |
| GOBP_MITOCHONDRIAL_ELECTRON_TRANSPORT_CYTOCHROME_C_TO_OXYGEN | -1.40 | 0.127 |
| GOBP_POSITIVE_REGULATION_OF_NON_CANONICAL_WNT_SIGNALING_PATHWAY | -1.40 | 0.128 |
| GOMF_ISOMERASE_ACTIVITY | -1.40 | 0.128 |
| GOBP_EMBRYONIC_CAMERA_TYPE_EYE_DEVELOPMENT | -1.40 | 0.128 |
| GOBP_MONOCYTE_DIFFERENTIATION | -1.40 | 0.128 |
| GOBP_MIDDLE_EAR_MORPHOGENESIS | -1.40 | 0.128 |
| GOMF_PHOSPHOLIPID_BINDING | -1.40 | 0.128 |
| GOBP_NEGATIVE_REGULATION_OF_EPITHELIAL_CELL_MIGRATION | -1.40 | 0.128 |
| GOBP_TOLERANCE_INDUCTION | -1.40 | 0.128 |
| GOBP_REGULATION_OF_CELLULAR_RESPONSE_TO_OXIDATIVE_STRESS | -1.40 | 0.128 |
| GOBP_HISTONE_MRNA_METABOLIC_PROCESS | -1.40 | 0.128 |
| GOBP_POSITIVE_REGULATION_OF_HYDROLASE_ACTIVITY | -1.40 | 0.128 |
| GOCC_CYTOSOLIC_REGION | -1.40 | 0.129 |
| GOBP_INTERLEUKIN_17_PRODUCTION | -1.40 | 0.129 |
| GOBP_ANTIGEN_PROCESSING_AND_PRESENTATION_OF_PEPTIDE_ANTIGEN_VIA_MHC_CLASS_I | -1.40 | 0.129 |
| GOCC_SARCOPLASM | -1.40 | 0.129 |
| GOBP_CARDIAC_RIGHT_VENTRICLE_MORPHOGENESIS | -1.40 | 0.129 |
| GOCC_PLATELET_DENSE_GRANULE | -1.40 | 0.129 |
| GOMF_SECONDARY_ACTIVE_TRANSMEMBRANE_TRANSPORTER_ACTIVITY | -1.40 | 0.129 |
| GOMF_MONOATOMIC_CATION_CHANNEL_ACTIVITY | -1.40 | 0.129 |
| GOMF_CYTOSKELETAL_ANCHOR_ACTIVITY | -1.40 | 0.129 |
| GOMF_ZINC_ION_TRANSMEMBRANE_TRANSPORTER_ACTIVITY | -1.40 | 0.129 |
| GOBP_INOSITOL_PHOSPHATE_BIOSYNTHETIC_PROCESS | -1.40 | 0.130 |
| GOMF_NUCLEOSIDE_DIPHOSPHATE_PHOSPHATASE_ACTIVITY | -1.40 | 0.130 |
| GOMF_CYCLASE_ACTIVITY | -1.40 | 0.130 |
| GOBP_REGULATION_OF_NEURON_PROJECTION_REGENERATION | -1.40 | 0.130 |
| GOBP_RESPONSE_TO_OSMOTIC_STRESS | -1.40 | 0.131 |
| GOBP_CARBOHYDRATE_BIOSYNTHETIC_PROCESS | -1.40 | 0.131 |
| GOBP_INTERLEUKIN_12_PRODUCTION | -1.39 | 0.131 |
| GOBP_HORMONE_TRANSPORT | -1.39 | 0.131 |
| GOBP_EMBRYONIC_CAMERA_TYPE_EYE_MORPHOGENESIS | -1.39 | 0.131 |
| GOBP_POSITIVE_REGULATION_OF_PEPTIDASE_ACTIVITY | -1.39 | 0.131 |
| GOCC_SYNAPTIC_CLEFT | -1.39 | 0.131 |
| GOBP_POSITIVE_REGULATION_OF_PATTERN_RECOGNITION_RECEPTOR_SIGNALING_PATHWAY | -1.39 | 0.131 |
| GOBP_REGULATION_OF_T_CELL_DIFFERENTIATION_IN_THYMUS | -1.39 | 0.132 |
| GOBP_REGULATION_OF_REPRODUCTIVE_PROCESS | -1.39 | 0.132 |
| GOBP_CELLULAR_OXIDANT_DETOXIFICATION | -1.39 | 0.132 |
| GOCC_CLATHRIN_COATED_VESICLE | -1.39 | 0.132 |
| GOBP_CELLULAR_RESPONSE_TO_FLUID_SHEAR_STRESS | -1.39 | 0.132 |
| GOBP_POSITIVE_REGULATION_OF_NEUROGENESIS | -1.39 | 0.132 |
| GOCC_RESPIRATORY_CHAIN_COMPLEX_IV | -1.39 | 0.133 |
| GOBP_MYELIN_ASSEMBLY | -1.39 | 0.133 |
| GOBP_TYPE_B_PANCREATIC_CELL_DEVELOPMENT | -1.39 | 0.133 |
| GOBP_LAMELLIPODIUM_MORPHOGENESIS | -1.39 | 0.133 |
| GOCC_GTPASE_COMPLEX | -1.39 | 0.133 |
| GOBP_NEGATIVE_REGULATION_OF_CELL_PROJECTION_ORGANIZATION | -1.39 | 0.133 |
| GOBP_REGULATION_OF_TOLL_LIKE_RECEPTOR_SIGNALING_PATHWAY | -1.39 | 0.133 |
| GOBP_POSITIVE_REGULATION_OF_VASCULAR_ENDOTHELIAL_CELL_PROLIFERATION | -1.39 | 0.133 |
| GOBP_DEVELOPMENT_OF_PRIMARY_SEXUAL_CHARACTERISTICS | -1.39 | 0.133 |
| GOBP_NEGATIVE_REGULATION_OF_NF_KAPPAB_TRANSCRIPTION_FACTOR_ACTIVITY | -1.39 | 0.133 |
| GOBP_GLOMERULAR_MESANGIUM_DEVELOPMENT | -1.39 | 0.133 |
| GOBP_RESPONSE_TO_INTERLEUKIN_6 | -1.39 | 0.133 |
| GOBP_PROTEIN_TRANSMEMBRANE_IMPORT_INTO_INTRACELLULAR_ORGANELLE | -1.39 | 0.133 |
| GOBP_BLASTODERM_SEGMENTATION | -1.39 | 0.133 |
| GOBP_RESPIRATORY_GASEOUS_EXCHANGE_BY_RESPIRATORY_SYSTEM | -1.39 | 0.133 |
| GOMF_PEPTIDE_BINDING | -1.39 | 0.133 |
| GOBP_MEMBRANE_PROTEIN_ECTODOMAIN_PROTEOLYSIS | -1.39 | 0.134 |
| GOBP_REGULATION_OF_RELEASE_OF_SEQUESTERED_CALCIUM_ION_INTO_CYTOSOL_BY_SARCOPLASMIC_RETICULUM | -1.39 | 0.134 |
| GOBP_MAMMARY_GLAND_EPITHELIAL_CELL_PROLIFERATION | -1.39 | 0.134 |
| GOBP_MYELOID_DENDRITIC_CELL_DIFFERENTIATION | -1.39 | 0.134 |
| GOBP_INNER_EAR_MORPHOGENESIS | -1.39 | 0.134 |
| GOBP_REGULATION_OF_LIPOPOLYSACCHARIDE_MEDIATED_SIGNALING_PATHWAY | -1.39 | 0.134 |
| GOBP_REGULATION_OF_PHOSPHATASE_ACTIVITY | -1.39 | 0.134 |
| GOBP_NEGATIVE_REGULATION_OF_NEURON_APOPTOTIC_PROCESS | -1.39 | 0.135 |
| GOBP_RESPONSE_TO_ATP | -1.39 | 0.135 |
| GOMF_PHOSPHOTYROSINE_RESIDUE_BINDING | -1.39 | 0.135 |
| GOBP_GLYCEROLIPID_METABOLIC_PROCESS | -1.39 | 0.135 |
| GOBP_NEGATIVE_REGULATION_OF_MYOBLAST_DIFFERENTIATION | -1.39 | 0.136 |
| GOBP_POSITIVE_REGULATION_OF_RESPONSE_TO_BIOTIC_STIMULUS | -1.39 | 0.136 |
| GOBP_NEURAL_TUBE_FORMATION | -1.39 | 0.136 |
| GOBP_AXON_EXTENSION | -1.39 | 0.136 |
| GOBP_VACUOLE_ORGANIZATION | -1.39 | 0.136 |
| GOBP_VENTRAL_SPINAL_CORD_DEVELOPMENT | -1.39 | 0.136 |
| GOBP_SEQUESTERING_OF_CALCIUM_ION | -1.39 | 0.136 |
| GOBP_SECONDARY_PALATE_DEVELOPMENT | -1.38 | 0.137 |
| GOMF_CALCIUM_ION_TRANSMEMBRANE_TRANSPORTER_ACTIVITY | -1.38 | 0.137 |
| GOMF_GATED_CHANNEL_ACTIVITY | -1.38 | 0.138 |
| GOBP_REGULATION_OF_STEROID_HORMONE_SECRETION | -1.38 | 0.138 |
| GOBP_PRESYNAPSE_ORGANIZATION | -1.38 | 0.138 |
| GOCC_ENDOLYSOSOME | -1.38 | 0.140 |
| GOMF_ORGANIC_ACID_TRANSMEMBRANE_TRANSPORTER_ACTIVITY | -1.38 | 0.140 |
| GOBP_RESPONSE_TO_ALDOSTERONE | -1.38 | 0.140 |
| GOMF_MONOATOMIC_CATION_TRANSMEMBRANE_TRANSPORTER_ACTIVITY | -1.38 | 0.140 |
| GOBP_GLYCOSYLATION | -1.38 | 0.141 |
| GOBP_SUBSTRATE_ADHESION_DEPENDENT_CELL_SPREADING | -1.38 | 0.141 |
| GOBP_LIPOPROTEIN_CATABOLIC_PROCESS | -1.38 | 0.141 |
| GOBP_CANONICAL_NF_KAPPAB_SIGNAL_TRANSDUCTION | -1.38 | 0.141 |
| GOBP_PHOSPHOLIPID_BIOSYNTHETIC_PROCESS | -1.38 | 0.141 |
| GOMF_TRANSMEMBRANE_RECEPTOR_PROTEIN_SERINE_THREONINE_KINASE_BINDING | -1.38 | 0.141 |
| GOBP_KERATINOCYTE_PROLIFERATION | -1.38 | 0.141 |
| GOMF_PHOSPHORIC_DIESTER_HYDROLASE_ACTIVITY | -1.38 | 0.141 |
| GOBP_KERATAN_SULFATE_METABOLIC_PROCESS | -1.38 | 0.141 |
| GOBP_POLYSACCHARIDE_METABOLIC_PROCESS | -1.38 | 0.141 |
| GOBP_RELAXATION_OF_CARDIAC_MUSCLE | -1.38 | 0.142 |
| GOBP_MATURE_B_CELL_DIFFERENTIATION | -1.38 | 0.142 |
| GOBP_CELLULAR_RESPONSE_TO_PEPTIDE_HORMONE_STIMULUS | -1.38 | 0.142 |
| GOBP_REGULATION_OF_PRESYNAPSE_ORGANIZATION | -1.38 | 0.142 |
| GOBP_PHARYNGEAL_SYSTEM_DEVELOPMENT | -1.38 | 0.142 |
| GOBP_POSITIVE_REGULATION_OF_MITOTIC_CELL_CYCLE | -1.38 | 0.142 |
| GOBP_SUBSTANTIA_NIGRA_DEVELOPMENT | -1.38 | 0.142 |
| GOBP_NEUROEPITHELIAL_CELL_DIFFERENTIATION | -1.38 | 0.143 |
| GOBP_T_CELL_LINEAGE_COMMITMENT | -1.38 | 0.143 |
| GOBP_B_CELL_DIFFERENTIATION | -1.38 | 0.143 |
| GOBP_INSULIN_LIKE_GROWTH_FACTOR_RECEPTOR_SIGNALING_PATHWAY | -1.38 | 0.143 |
| GOBP_NEGATIVE_REGULATION_OF_INFLAMMATORY_RESPONSE | -1.38 | 0.143 |
| GOMF_TRANSPORTER_INHIBITOR_ACTIVITY | -1.37 | 0.144 |
| GOBP_PLACENTA_BLOOD_VESSEL_DEVELOPMENT | -1.37 | 0.144 |
| GOCC_COATED_VESICLE | -1.37 | 0.144 |
| GOBP_REGULATION_OF_CELL_MATRIX_ADHESION | -1.37 | 0.144 |
| GOMF_UBIQUITIN_SPECIFIC_PROTEASE_BINDING | -1.37 | 0.144 |
| GOBP_CELLULAR_RESPONSE_TO_DEXAMETHASONE_STIMULUS | -1.37 | 0.144 |
| GOBP_POSITIVE_REGULATION_OF_GLIOGENESIS | -1.37 | 0.145 |
| GOBP_MEMBRANE_DEPOLARIZATION | -1.37 | 0.145 |
| GOBP_REGULATION_OF_VASOCONSTRICTION | -1.37 | 0.145 |
| GOBP_REGULATION_OF_ENDOTHELIAL_CELL_DIFFERENTIATION | -1.37 | 0.145 |
| GOBP_SKELETAL_MUSCLE_CONTRACTION | -1.37 | 0.145 |
| GOBP_MUSCLE_HYPERTROPHY | -1.37 | 0.145 |
| GOMF_DNA_BINDING_TRANSCRIPTION_REPRESSOR_ACTIVITY | -1.37 | 0.145 |
| GOBP_NEGATIVE_REGULATION_OF_IMMUNE_RESPONSE | -1.37 | 0.145 |
| GOMF_ACETYLGALACTOSAMINYLTRANSFERASE_ACTIVITY | -1.37 | 0.145 |
| GOBP_POLYOL_METABOLIC_PROCESS | -1.37 | 0.145 |
| GOCC_TERTIARY_GRANULE_LUMEN | -1.37 | 0.145 |
| GOBP_RETINA_DEVELOPMENT_IN_CAMERA_TYPE_EYE | -1.37 | 0.145 |
| GOBP_POSITIVE_REGULATION_OF_SIGNALING_RECEPTOR_ACTIVITY | -1.37 | 0.145 |
| GOBP_NEGATIVE_REGULATION_OF_DEFENSE_RESPONSE | -1.37 | 0.145 |
| GOMF_ADRENERGIC_RECEPTOR_BINDING | -1.37 | 0.146 |
| GOBP_MULTICELLULAR_ORGANISMAL_MOVEMENT | -1.37 | 0.146 |
| GOMF_PEPTIDASE_ACTIVITY | -1.37 | 0.147 |
| GOBP_PEPTIDYL_THREONINE_DEPHOSPHORYLATION | -1.37 | 0.147 |
| GOBP_SARCOPLASMIC_RETICULUM_CALCIUM_ION_TRANSPORT | -1.37 | 0.148 |
| GOBP_PROTEIN_IMPORT_INTO_MITOCHONDRIAL_MATRIX | -1.37 | 0.148 |
| GOBP_CYTOPLASMIC_SEQUESTERING_OF_PROTEIN | -1.37 | 0.148 |
| GOBP_EATING_BEHAVIOR | -1.37 | 0.148 |
| GOBP_RESPONSE_TO_NUTRIENT_LEVELS | -1.37 | 0.148 |
| GOBP_PROTEIN_O_LINKED_MANNOSYLATION | -1.37 | 0.148 |
| GOBP_CHONDROCYTE_PROLIFERATION | -1.37 | 0.149 |
| GOBP_POSITIVE_REGULATION_OF_JUN_KINASE_ACTIVITY | -1.37 | 0.149 |
| GOBP_POSITIVE_REGULATION_BY_HOST_OF_VIRAL_PROCESS | -1.37 | 0.149 |
| GOBP_PROTEIN_MATURATION_BY_IRON_SULFUR_CLUSTER_TRANSFER | -1.37 | 0.149 |
| GOBP_POSITIVE_REGULATION_OF_MUSCLE_CONTRACTION | -1.37 | 0.150 |
| GOCC_GOLGI_APPARATUS_SUBCOMPARTMENT | -1.37 | 0.150 |
| GOBP_CELLULAR_RESPONSE_TO_OSMOTIC_STRESS | -1.36 | 0.151 |
| GOMF_METALLOCARBOXYPEPTIDASE_ACTIVITY | -1.36 | 0.151 |
| GOBP_INTEGRATED_STRESS_RESPONSE_SIGNALING | -1.36 | 0.152 |
| GOBP_POSITIVE_REGULATION_OF_PROTEOLYSIS | -1.36 | 0.152 |
| GOMF_CELL_ADHESION_MEDIATOR_ACTIVITY | -1.36 | 0.152 |
| GOBP_EAR_MORPHOGENESIS | -1.36 | 0.152 |
| GOBP_NEGATIVE_REGULATION_OF_CELLULAR_RESPONSE_TO_GROWTH_FACTOR_STIMULUS | -1.36 | 0.153 |
| GOBP_CELLULAR_RESPONSE_TO_VITAMIN_D | -1.36 | 0.153 |
| GOBP_MEMBRANE_FUSION | -1.36 | 0.153 |
| GOBP_NON_CANONICAL_NF_KAPPAB_SIGNAL_TRANSDUCTION | -1.36 | 0.153 |
| GOBP_NERVE_DEVELOPMENT | -1.36 | 0.154 |
| GOBP_PROTEIN_NEDDYLATION | -1.36 | 0.154 |
| GOMF_SYMPORTER_ACTIVITY | -1.36 | 0.154 |
| GOBP_REGULATION_OF_MAST_CELL_DEGRANULATION | -1.36 | 0.154 |
| GOMF_MANGANESE_ION_BINDING | -1.36 | 0.154 |
| GOBP_REGULATION_OF_CARDIAC_MUSCLE_CONTRACTION_BY_REGULATION_OF_THE_RELEASE_OF_SEQUESTERED_CALCIUM_ION | -1.36 | 0.154 |
| GOBP_XENOBIOTIC_TRANSPORT | -1.36 | 0.154 |
| GOBP_POSITIVE_REGULATION_OF_TELOMERASE_RNA_LOCALIZATION_TO_CAJAL_BODY | -1.36 | 0.155 |
| GOBP_CALCIUM_ION_TRANSMEMBRANE_IMPORT_INTO_CYTOSOL | -1.36 | 0.155 |
| GOBP_CYCLIC_NUCLEOTIDE_METABOLIC_PROCESS | -1.36 | 0.155 |
| GOBP_ATRIOVENTRICULAR_VALVE_DEVELOPMENT | -1.36 | 0.155 |
| GOBP_NEGATIVE_REGULATION_OF_ENDOPLASMIC_RETICULUM_STRESS_INDUCED_INTRINSIC_APOPTOTIC_SIGNALING_PATHWAY | -1.36 | 0.156 |
| GOBP_ACTIVATION_OF_INNATE_IMMUNE_RESPONSE | -1.36 | 0.156 |
| GOBP_CARBOHYDRATE_DERIVATIVE_CATABOLIC_PROCESS | -1.36 | 0.156 |
| GOBP_POSITIVE_REGULATION_OF_VASOCONSTRICTION | -1.36 | 0.157 |
| GOBP_POSITIVE_REGULATION_OF_MITOCHONDRIAL_FISSION | -1.36 | 0.157 |
| GOBP_NUCLEAR_PORE_ORGANIZATION | -1.36 | 0.157 |
| GOBP_REGULATION_OF_RELEASE_OF_SEQUESTERED_CALCIUM_ION_INTO_CYTOSOL | -1.36 | 0.157 |
| GOBP_NEGATIVE_REGULATION_OF_PROTEIN_LOCALIZATION | -1.36 | 0.157 |
| GOBP_STEM_CELL_DIVISION | -1.36 | 0.157 |
| GOBP_REGULATION_OF_AEROBIC_RESPIRATION | -1.36 | 0.157 |
| GOCC_SCHAFFER_COLLATERAL_CA1_SYNAPSE | -1.36 | 0.157 |
| GOBP_CELL_FATE_SPECIFICATION | -1.36 | 0.157 |
| GOBP_REGULATION_OF_CYSTEINE_TYPE_ENDOPEPTIDASE_ACTIVITY_INVOLVED_IN_APOPTOTIC_PROCESS | -1.35 | 0.157 |
| GOBP_PROTEIN_COMPLEX_OLIGOMERIZATION | -1.35 | 0.158 |
| GOBP_SEX_CHROMOSOME_DOSAGE_COMPENSATION | -1.35 | 0.158 |
| GOBP_POSITIVE_REGULATION_OF_CELL_PROJECTION_ORGANIZATION | -1.35 | 0.158 |
| GOBP_REGULATION_OF_MUSCLE_TISSUE_DEVELOPMENT | -1.35 | 0.158 |
| GOMF_CARBOHYDRATE_TRANSMEMBRANE_TRANSPORTER_ACTIVITY | -1.35 | 0.158 |
| GOBP_MITOTIC_SISTER_CHROMATID_COHESION | -1.35 | 0.159 |
| GOBP_NEUROMUSCULAR_JUNCTION_DEVELOPMENT | -1.35 | 0.159 |
| GOCC_COPI_COATED_VESICLE_MEMBRANE | -1.35 | 0.159 |
| GOBP_RESPONSE_TO_TEMPERATURE_STIMULUS | -1.35 | 0.160 |
| GOBP_INTRACELLULAR_PH_REDUCTION | -1.35 | 0.160 |
| GOMF_C_C_CHEMOKINE_BINDING | -1.35 | 0.160 |
| GOCC_COPI_COATED_VESICLE | -1.35 | 0.160 |
| GOMF_METALLOAMINOPEPTIDASE_ACTIVITY | -1.35 | 0.161 |
| GOBP_ANTIMICROBIAL_HUMORAL_IMMUNE_RESPONSE_MEDIATED_BY_ANTIMICROBIAL_PEPTIDE | -1.35 | 0.161 |
| GOBP_DETECTION_OF_STIMULUS | -1.35 | 0.161 |
| GOBP_CALCINEURIN_MEDIATED_SIGNALING | -1.35 | 0.161 |
| GOBP_NEGATIVE_REGULATION_OF_ESTABLISHMENT_OF_PROTEIN_LOCALIZATION | -1.35 | 0.161 |
| GOBP_RESPONSE_TO_FATTY_ACID | -1.35 | 0.161 |
| GOMF_PROTEIN_SEQUESTERING_ACTIVITY | -1.35 | 0.161 |
| GOBP_NEGATIVE_REGULATION_OF_EXTRINSIC_APOPTOTIC_SIGNALING_PATHWAY_VIA_DEATH_DOMAIN_RECEPTORS | -1.35 | 0.161 |
| GOBP_VITAMIN_TRANSPORT | -1.35 | 0.161 |
| GOBP_AMINO_ACID_TRANSPORT | -1.35 | 0.161 |
| GOMF_CHLORIDE_TRANSMEMBRANE_TRANSPORTER_ACTIVITY | -1.35 | 0.162 |
| GOBP_ENDOPLASMIC_RETICULUM_ORGANIZATION | -1.35 | 0.163 |
| GOBP_RETINOL_METABOLIC_PROCESS | -1.35 | 0.164 |
| GOBP_MONOATOMIC_ANION_TRANSPORT | -1.35 | 0.165 |
| GOMF_MHC_CLASS_II_PROTEIN_COMPLEX_BINDING | -1.35 | 0.165 |
| GOBP_NEGATIVE_REGULATION_OF_MUSCLE_CELL_APOPTOTIC_PROCESS | -1.34 | 0.166 |
| GOBP_CHLORIDE_TRANSPORT | -1.34 | 0.166 |
| GOBP_POSITIVE_REGULATION_OF_T_CELL_MIGRATION | -1.34 | 0.166 |
| GOBP_REGULATION_OF_CYSTEINE_TYPE_ENDOPEPTIDASE_ACTIVITY | -1.34 | 0.166 |
| GOBP_RESPONSE_TO_INSULIN | -1.34 | 0.166 |
| GOBP_OVULATION_CYCLE_PROCESS | -1.34 | 0.166 |
| GOBP_REGULATION_OF_ADENYLATE_CYCLASE_ACTIVITY | -1.34 | 0.166 |
| GOBP_CELLULAR_RESPONSE_TO_VITAMIN | -1.34 | 0.166 |
| GOCC_GOLGI_CISTERNA | -1.34 | 0.166 |
| GOMF_GLYCOLIPID_BINDING | -1.34 | 0.166 |
| GOBP_REGULATION_OF_LEUKOCYTE_APOPTOTIC_PROCESS | -1.34 | 0.166 |
| GOBP_REGULATION_OF_EXTENT_OF_CELL_GROWTH | -1.34 | 0.166 |
| GOBP_PANCREAS_DEVELOPMENT | -1.34 | 0.167 |
| GOBP_NEGATIVE_REGULATION_OF_PEPTIDE_SECRETION | -1.34 | 0.167 |
| GOBP_MULTICELLULAR_ORGANISMAL_LEVEL_WATER_HOMEOSTASIS | -1.34 | 0.167 |
| GOBP_EMBRYONIC_HEMOPOIESIS | -1.34 | 0.168 |
| GOMF_PROTEIN_SERINE_THREONINE_PHOSPHATASE_ACTIVITY | -1.34 | 0.167 |
| GOBP_PHOSPHATIDYLCHOLINE_BIOSYNTHETIC_PROCESS | -1.34 | 0.168 |
| GOCC_COATED_VESICLE_MEMBRANE | -1.34 | 0.168 |
| GOBP_THIOESTER_BIOSYNTHETIC_PROCESS | -1.34 | 0.168 |
| GOBP_MAINTENANCE_OF_PROTEIN_LOCATION_IN_CELL | -1.34 | 0.168 |
| GOBP_PROTEIN_LOCALIZATION_TO_CELL_PERIPHERY | -1.34 | 0.169 |
| GOBP_POSITIVE_REGULATION_OF_NATURAL_KILLER_CELL_ACTIVATION | -1.34 | 0.168 |
| GOBP_FATTY_ACID_TRANSPORT | -1.34 | 0.169 |
| GOBP_LEUKOCYTE_ADHESION_TO_VASCULAR_ENDOTHELIAL_CELL | -1.34 | 0.169 |
| GOBP_CELLULAR_RESPONSE_TO_CADMIUM_ION | -1.34 | 0.170 |
| GOBP_POSITIVE_REGULATION_OF_PEPTIDYL_SERINE_PHOSPHORYLATION | -1.34 | 0.170 |
| GOBP_APOPTOTIC_PROCESS_INVOLVED_IN_MORPHOGENESIS | -1.34 | 0.170 |
| GOBP_PROTEIN_INSERTION_INTO_ER_MEMBRANE | -1.34 | 0.170 |
| GOBP_REGULATION_OF_ALPHA_BETA_T_CELL_DIFFERENTIATION | -1.34 | 0.170 |
| GOBP_MODIFIED_AMINO_ACID_TRANSPORT | -1.34 | 0.170 |
| GOMF_MONOATOMIC_ANION_TRANSMEMBRANE_TRANSPORTER_ACTIVITY | -1.34 | 0.171 |
| GOBP_POLYOL_BIOSYNTHETIC_PROCESS | -1.34 | 0.171 |
| GOMF_HORMONE_BINDING | -1.34 | 0.172 |
| GOBP_NEGATIVE_REGULATION_OF_PROTEIN_SERINE_THREONINE_KINASE_ACTIVITY | -1.34 | 0.172 |
| GOCC_MITOCHONDRIAL_MATRIX | -1.34 | 0.172 |
| GOBP_NEGATIVE_REGULATION_OF_LYMPHOCYTE_MEDIATED_IMMUNITY | -1.34 | 0.172 |
| GOBP_RESPONSE_TO_SALT | -1.33 | 0.174 |
| GOMF_GDP_BINDING | -1.33 | 0.174 |
| GOBP_POSITIVE_REGULATION_OF_EXOCYTOSIS | -1.33 | 0.174 |
| GOBP_SULFUR_AMINO_ACID_METABOLIC_PROCESS | -1.33 | 0.174 |
| GOBP_CELLULAR_PIGMENTATION | -1.33 | 0.174 |
| GOBP_CELLULAR_RESPONSE_TO_HYDROGEN_PEROXIDE | -1.33 | 0.174 |
| GOBP_PROTEIN_LOCALIZATION_TO_PLASMA_MEMBRANE | -1.33 | 0.174 |
| GOBP_NEGATIVE_REGULATION_OF_LEUKOCYTE_PROLIFERATION | -1.33 | 0.174 |
| GOBP_BLOOD_VESSEL_REMODELING | -1.33 | 0.175 |
| GOBP_REGULATION_OF_SKELETAL_MUSCLE_CELL_DIFFERENTIATION | -1.33 | 0.175 |
| GOCC_SARCOPLASMIC_RETICULUM | -1.33 | 0.175 |
| GOBP_RESPONSE_TO_MANGANESE_ION | -1.33 | 0.175 |
| GOBP_LEUKOCYTE_APOPTOTIC_PROCESS | -1.33 | 0.175 |
| GOBP_POSITIVE_REGULATION_OF_PROGRAMMED_CELL_DEATH | -1.33 | 0.175 |
| GOBP_NEURON_PROJECTION_EXTENSION | -1.33 | 0.176 |
| GOBP_PARAXIAL_MESODERM_DEVELOPMENT | -1.33 | 0.176 |
| GOBP_GLYCOLIPID_BIOSYNTHETIC_PROCESS | -1.33 | 0.178 |
| GOBP_PYRIDINE_CONTAINING_COMPOUND_METABOLIC_PROCESS | -1.33 | 0.178 |
| GOMF_5_3_EXONUCLEASE_ACTIVITY | -1.33 | 0.178 |
| GOBP_RENAL_WATER_HOMEOSTASIS | -1.33 | 0.179 |
| GOBP_NEGATIVE_REGULATION_OF_OSTEOBLAST_DIFFERENTIATION | -1.33 | 0.179 |
| GOMF_CARBOXYLIC_ESTER_HYDROLASE_ACTIVITY | -1.33 | 0.179 |
| GOBP_SIGNAL_TRANSDUCTION_IN_ABSENCE_OF_LIGAND | -1.33 | 0.179 |
| GOBP_ESTABLISHMENT_OR_MAINTENANCE_OF_CELL_POLARITY | -1.33 | 0.180 |
| GOBP_DEPHOSPHORYLATION | -1.33 | 0.181 |
| GOBP_MESENCHYMAL_CELL_MIGRATION | -1.32 | 0.182 |
| GOBP_CEREBELLAR_CORTEX_DEVELOPMENT | -1.32 | 0.182 |
| GOBP_MICROTUBULE_DEPOLYMERIZATION | -1.32 | 0.182 |
| GOBP_SULFUR_COMPOUND_TRANSPORT | -1.32 | 0.182 |
| GOBP_LIVER_REGENERATION | -1.32 | 0.182 |
| GOMF_ADENYL_NUCLEOTIDE_EXCHANGE_FACTOR_ACTIVITY | -1.32 | 0.182 |
| GOBP_ADAPTIVE_THERMOGENESIS | -1.32 | 0.183 |
| GOCC_TRANSPORT_VESICLE | -1.32 | 0.183 |
| GOBP_REGULATION_OF_APOPTOTIC_SIGNALING_PATHWAY | -1.32 | 0.183 |
| GOBP_SOMATIC_DIVERSIFICATION_OF_IMMUNE_RECEPTORS | -1.32 | 0.183 |
| GOBP_PROTEIN_O_LINKED_GLYCOSYLATION | -1.32 | 0.183 |
| GOBP_SYNAPTIC_TRANSMISSION_DOPAMINERGIC | -1.32 | 0.183 |
| GOBP_CENTRAL_NERVOUS_SYSTEM_NEURON_DIFFERENTIATION | -1.32 | 0.184 |
| GOBP_BODY_FLUID_SECRETION | -1.32 | 0.184 |
| GOBP_RESPONSE_TO_ENDOPLASMIC_RETICULUM_STRESS | -1.32 | 0.184 |
| GOBP_MEMBRANE_LIPID_CATABOLIC_PROCESS | -1.32 | 0.184 |
| GOBP_REGULATION_OF_AXON_EXTENSION | -1.32 | 0.184 |
| GOBP_REGULATION_OF_NOTCH_SIGNALING_PATHWAY | -1.32 | 0.185 |
| GOBP_REGULATION_OF_SYNAPTIC_TRANSMISSION_GABAERGIC | -1.32 | 0.185 |
| GOBP_RETINOIC_ACID_METABOLIC_PROCESS | -1.32 | 0.185 |
| GOBP_STRIATED_MUSCLE_CELL_APOPTOTIC_PROCESS | -1.32 | 0.185 |
| GOBP_DIGESTIVE_SYSTEM_PROCESS | -1.32 | 0.185 |
| GOMF_PENTOSYLTRANSFERASE_ACTIVITY | -1.32 | 0.185 |
| GOBP_POSITIVE_REGULATION_OF_REGULATED_SECRETORY_PATHWAY | -1.32 | 0.185 |
| GOBP_CYTOSOLIC_TRANSPORT | -1.32 | 0.184 |
| GOMF_DISULFIDE_OXIDOREDUCTASE_ACTIVITY | -1.32 | 0.185 |
| GOMF_POTASSIUM_ION_TRANSMEMBRANE_TRANSPORTER_ACTIVITY | -1.32 | 0.185 |
| GOMF_MEMBRANE_INSERTASE_ACTIVITY | -1.32 | 0.185 |
| GOMF_INSULIN_RECEPTOR_BINDING | -1.32 | 0.186 |
| GOMF_ADP_BINDING | -1.32 | 0.186 |
| GOBP_GOLGI_VESICLE_TRANSPORT | -1.32 | 0.186 |
| GOBP_REGULATION_OF_POLYSACCHARIDE_METABOLIC_PROCESS | -1.32 | 0.186 |
| GOMF_MONOCARBOXYLIC_ACID_BINDING | -1.32 | 0.186 |
| GOBP_CELL_SURFACE_PATTERN_RECOGNITION_RECEPTOR_SIGNALING_PATHWAY | -1.32 | 0.186 |
| GOBP_COCHLEA_DEVELOPMENT | -1.32 | 0.187 |
| GOBP_POSITIVE_REGULATION_OF_RECEPTOR_SIGNALING_PATHWAY_VIA_STAT | -1.32 | 0.187 |
| GOBP_LYMPHOCYTE_HOMEOSTASIS | -1.32 | 0.187 |
| GOBP_REGULATION_OF_SYNAPSE_STRUCTURE_OR_ACTIVITY | -1.32 | 0.188 |
| GOBP_REGULATION_OF_SMALL_MOLECULE_METABOLIC_PROCESS | -1.32 | 0.188 |
| GOBP_MAINTENANCE_OF_CELL_POLARITY | -1.32 | 0.188 |
| GOBP_POSITIVE_REGULATION_OF_DEVELOPMENTAL_GROWTH | -1.32 | 0.188 |
| GOBP_POSITIVE_REGULATION_OF_REPRODUCTIVE_PROCESS | -1.32 | 0.188 |
| GOBP_POSITIVE_REGULATION_OF_SMALL_GTPASE_MEDIATED_SIGNAL_TRANSDUCTION | -1.32 | 0.188 |
| GOBP_NEGATIVE_REGULATION_OF_STEM_CELL_POPULATION_MAINTENANCE | -1.32 | 0.188 |
| GOBP_PROTEIN_TETRAMERIZATION | -1.32 | 0.188 |
| GOBP_ANIMAL_ORGAN_REGENERATION | -1.31 | 0.189 |
| GOCC_LAMELLIPODIUM | -1.31 | 0.189 |
| GOBP_POSITIVE_REGULATION_OF_BIOMINERAL_TISSUE_DEVELOPMENT | -1.31 | 0.190 |
| GOBP_ENDOLYSOSOMAL_TOLL_LIKE_RECEPTOR_SIGNALING_PATHWAY | -1.31 | 0.190 |
| GOBP_POSITIVE_REGULATION_OF_NEUTROPHIL_MIGRATION | -1.31 | 0.190 |
| GOBP_RHYTHMIC_BEHAVIOR | -1.31 | 0.190 |
| GOBP_EPITHELIAL_TO_MESENCHYMAL_TRANSITION_INVOLVED_IN_ENDOCARDIAL_CUSHION_FORMATION | -1.31 | 0.190 |
| GOCC_CATENIN_COMPLEX | -1.31 | 0.191 |
| GOBP_REGULATION_OF_RESPONSE_TO_BIOTIC_STIMULUS | -1.31 | 0.191 |
| GOBP_AMINO_ACID_TRANSMEMBRANE_TRANSPORT | -1.31 | 0.192 |
| GOBP_POSITIVE_REGULATION_OF_LYMPHOCYTE_MIGRATION | -1.31 | 0.191 |
| GOBP_CELL_CELL_JUNCTION_ORGANIZATION | -1.31 | 0.192 |
| GOBP_BEHAVIOR | -1.31 | 0.192 |
| GOMF_ACETYLGLUCOSAMINYLTRANSFERASE_ACTIVITY | -1.31 | 0.192 |
| GOBP_POSITIVE_REGULATION_OF_CELL_CYCLE | -1.31 | 0.193 |
| GOBP_TONGUE_DEVELOPMENT | -1.31 | 0.193 |
| GOBP_REGULATION_OF_TOLERANCE_INDUCTION | -1.31 | 0.193 |
| GOBP_COLUMNAR_CUBOIDAL_EPITHELIAL_CELL_DEVELOPMENT | -1.31 | 0.193 |
| GOBP_MUSCLE_CELL_CELLULAR_HOMEOSTASIS | -1.31 | 0.193 |
| GOBP_REGULATION_OF_PROTEIN_POLYMERIZATION | -1.31 | 0.193 |
| GOBP_HIPPOCAMPUS_DEVELOPMENT | -1.31 | 0.193 |
| GOBP_NEGATIVE_REGULATION_OF_HYDROLASE_ACTIVITY | -1.31 | 0.194 |
| GOBP_REGULATION_OF_MYELOID_CELL_APOPTOTIC_PROCESS | -1.31 | 0.194 |
| GOMF_ENDOPEPTIDASE_ACTIVATOR_ACTIVITY | -1.31 | 0.195 |
| GOMF_HYDROLASE_ACTIVITY_ACTING_ON_CARBON_NITROGEN_BUT_NOT_PEPTIDE_BONDS | -1.31 | 0.195 |
| GOBP_RESPONSE_TO_STEROL_DEPLETION | -1.31 | 0.195 |
| GOMF_LIPID_TRANSPORTER_ACTIVITY | -1.31 | 0.195 |
| GOBP_POSITIVE_REGULATION_OF_ACTIN_FILAMENT_BUNDLE_ASSEMBLY | -1.31 | 0.195 |
| GOBP_REGULATION_OF_PROTEIN_MATURATION | -1.31 | 0.195 |
| GOCC_GABA_ERGIC_SYNAPSE | -1.31 | 0.196 |
| GOBP_ENSHEATHMENT_OF_NEURONS | -1.31 | 0.196 |
| GOBP_POSITIVE_REGULATION_OF_PROTEIN_CATABOLIC_PROCESS | -1.31 | 0.196 |
| GOCC_DISTAL_AXON | -1.31 | 0.196 |
| GOBP_ESTABLISHMENT_OF_SPINDLE_ORIENTATION | -1.31 | 0.196 |
| GOBP_CELLULAR_RESPONSE_TO_EXTERNAL_STIMULUS | -1.31 | 0.196 |
| GOBP_NEGATIVE_REGULATION_OF_SYSTEMIC_ARTERIAL_BLOOD_PRESSURE | -1.31 | 0.196 |
| GOBP_NEGATIVE_REGULATION_OF_TOLL_LIKE_RECEPTOR_SIGNALING_PATHWAY | -1.31 | 0.196 |
| GOBP_BRANCHING_INVOLVED_IN_BLOOD_VESSEL_MORPHOGENESIS | -1.31 | 0.196 |
| GOBP_ANDROGEN_RECEPTOR_SIGNALING_PATHWAY | -1.31 | 0.196 |
| GOCC_MITOCHONDRIAL_PROTEIN_CONTAINING_COMPLEX | -1.31 | 0.197 |
| GOCC_LIPID_DROPLET | -1.30 | 0.197 |
| GOBP_SEMAPHORIN_PLEXIN_SIGNALING_PATHWAY | -1.30 | 0.197 |
| GOMF_TRANSFORMING_GROWTH_FACTOR_BETA_BINDING | -1.30 | 0.197 |
| GOBP_NEGATIVE_REGULATION_OF_ORGANIC_ACID_TRANSPORT | -1.30 | 0.197 |
| GOBP_LEUKOTRIENE_METABOLIC_PROCESS | -1.30 | 0.197 |
| GOMF_GDP_DISSOCIATION_INHIBITOR_ACTIVITY | -1.30 | 0.197 |
| GOCC_PRESYNAPSE | -1.30 | 0.197 |
| GOCC_PORE_COMPLEX | -1.30 | 0.197 |
| GOBP_NEGATIVE_REGULATION_OF_ENDOCYTOSIS | -1.30 | 0.197 |
| GOBP_NATURAL_KILLER_CELL_ACTIVATION | -1.30 | 0.197 |
| GOCC_POSTSYNAPSE | -1.30 | 0.197 |
| GOBP_ECTOPIC_GERM_CELL_PROGRAMMED_CELL_DEATH | -1.30 | 0.198 |
| GOMF_PROTEIN_TRANSMEMBRANE_TRANSPORTER_ACTIVITY | -1.30 | 0.198 |
| GOBP_CELL_CYCLE_G1_S_PHASE_TRANSITION | -1.30 | 0.198 |
| GOBP_SERTOLI_CELL_DIFFERENTIATION | -1.30 | 0.198 |
| GOBP_EMBRYONIC_PLACENTA_DEVELOPMENT | -1.30 | 0.199 |
| GOBP_POSITIVE_REGULATION_OF_T_CELL_MEDIATED_IMMUNITY | -1.30 | 0.199 |
| GOMF_UNFOLDED_PROTEIN_BINDING | -1.30 | 0.199 |
| GOBP_REGULATION_OF_PHOSPHOPROTEIN_PHOSPHATASE_ACTIVITY | -1.30 | 0.199 |
| GOBP_FOCAL_ADHESION_ASSEMBLY | -1.30 | 0.199 |
| GOBP_GOLGI_TO_ENDOSOME_TRANSPORT | -1.30 | 0.200 |
| GOMF_QUINONE_BINDING | -1.30 | 0.200 |
| GOBP_HINDBRAIN_DEVELOPMENT | -1.30 | 0.201 |
| GOBP_PEPTIDE_TRANSPORT | -1.30 | 0.201 |
| GOMF_AMINO_ACID_TRANSMEMBRANE_TRANSPORTER_ACTIVITY | -1.30 | 0.201 |
| GOBP_CELLULAR_RESPONSE_TO_GLUCOCORTICOID_STIMULUS | -1.30 | 0.201 |
| GOBP_VESICLE_MEDIATED_TRANSPORT_IN_SYNAPSE | -1.30 | 0.201 |
| GOCC_CELL_SUBSTRATE_JUNCTION | -1.30 | 0.201 |
| GOBP_AROMATIC_AMINO_ACID_METABOLIC_PROCESS | -1.30 | 0.202 |
| GOBP_CYCLIC_NUCLEOTIDE_BIOSYNTHETIC_PROCESS | -1.30 | 0.203 |
| GOBP_NEGATIVE_REGULATION_OF_UBIQUITIN_PROTEIN_TRANSFERASE_ACTIVITY | -1.30 | 0.203 |
| GOBP_AGGREPHAGY | -1.30 | 0.204 |
| GOBP_POSITIVE_REGULATION_OF_ORGANELLE_ORGANIZATION | -1.30 | 0.205 |
| GOBP_POSITIVE_REGULATION_OF_COLD_INDUCED_THERMOGENESIS | -1.30 | 0.205 |
| GOMF_PEPTIDASE_REGULATOR_ACTIVITY | -1.30 | 0.205 |
| GOBP_NEGATIVE_REGULATION_OF_PRODUCTION_OF_MOLECULAR_MEDIATOR_OF_IMMUNE_RESPONSE | -1.30 | 0.205 |
| GOMF_RIBOSOMAL_SMALL_SUBUNIT_BINDING | -1.30 | 0.205 |
| GOMF_VITAMIN_TRANSMEMBRANE_TRANSPORTER_ACTIVITY | -1.30 | 0.205 |
| GOBP_NEGATIVE_REGULATION_OF_TRANSFORMING_GROWTH_FACTOR_BETA_RECEPTOR_SIGNALING_PATHWAY | -1.29 | 0.206 |
| GOBP_CEREBELLAR_CORTEX_FORMATION | -1.29 | 0.206 |
| GOBP_REGULATION_OF_COLLATERAL_SPROUTING | -1.29 | 0.206 |
| GOBP_REGULATION_OF_PROTEIN_NEDDYLATION | -1.29 | 0.206 |
| GOBP_PIGMENT_GRANULE_ORGANIZATION | -1.29 | 0.207 |
| GOBP_REGULATION_OF_GLYCOGEN_METABOLIC_PROCESS | -1.29 | 0.207 |
| GOBP_NEGATIVE_REGULATION_OF_OSSIFICATION | -1.29 | 0.207 |
| GOMF_NAD_BINDING | -1.29 | 0.207 |
| GOBP_POSITIVE_REGULATION_OF_SMOOTH_MUSCLE_CONTRACTION | -1.29 | 0.208 |
| GOBP_NEGATIVE_REGULATION_OF_TRANSFERASE_ACTIVITY | -1.29 | 0.208 |
| GOBP_CELLULAR_RESPONSE_TO_STEROID_HORMONE_STIMULUS | -1.29 | 0.208 |
| GOBP_PROTEIN_AUTOPHOSPHORYLATION | -1.29 | 0.208 |
| GOMF_LYSOPHOSPHOLIPID_ACYLTRANSFERASE_ACTIVITY | -1.29 | 0.209 |
| GOBP_PROTEIN_HOMOOLIGOMERIZATION | -1.29 | 0.209 |
| GOBP_PROTEIN_POLYMERIZATION | -1.29 | 0.209 |
| GOMF_OXIDOREDUCTASE_ACTIVITY_ACTING_ON_A_SULFUR_GROUP_OF_DONORS | -1.29 | 0.209 |
| GOBP_FATTY_ACID_TRANSMEMBRANE_TRANSPORT | -1.29 | 0.209 |
| GOBP_REGULATION_OF_NON_CANONICAL_NF_KAPPAB_SIGNAL_TRANSDUCTION | -1.29 | 0.209 |
| GOCC_TIGHT_JUNCTION | -1.29 | 0.210 |
| GOBP_HYALURONAN_METABOLIC_PROCESS | -1.29 | 0.210 |
| GOMF_MYOSIN_PHOSPHATASE_ACTIVITY | -1.29 | 0.211 |
| GOMF_SMALL_MOLECULE_SENSOR_ACTIVITY | -1.29 | 0.211 |
| GOMF_PHOSPHATIDYLINOSITOL_PHOSPHATE_BINDING | -1.29 | 0.211 |
| GOBP_POSITIVE_REGULATION_OF_GRANULOCYTE_CHEMOTAXIS | -1.29 | 0.211 |
| GOBP_G_PROTEIN_COUPLED_RECEPTOR_SIGNALING_PATHWAY_COUPLED_TO_CYCLIC_NUCLEOTIDE_SECOND_MESSENGER | -1.29 | 0.212 |
| GOMF_HISTONE_H4_METHYLTRANSFERASE_ACTIVITY | -1.29 | 0.212 |
| GOBP_ADULT_LOCOMOTORY_BEHAVIOR | -1.29 | 0.212 |
| GOBP_REGULATION_OF_CYTOSKELETON_ORGANIZATION | -1.29 | 0.213 |
| GOBP_REGULATION_OF_PROTEIN_UBIQUITINATION | -1.29 | 0.213 |
| GOBP_RESPONSE_TO_ZINC_ION | -1.29 | 0.213 |
| GOBP_PROTEIN_KINASE_C_SIGNALING | -1.29 | 0.213 |
| GOBP_RESPONSE_TO_MISFOLDED_PROTEIN | -1.29 | 0.213 |
| GOBP_POSITIVE_REGULATION_OF_ENDOTHELIAL_CELL_APOPTOTIC_PROCESS | -1.29 | 0.214 |
| GOBP_PROTEIN_TARGETING | -1.29 | 0.214 |
| GOBP_REGULATION_OF_INNATE_IMMUNE_RESPONSE | -1.29 | 0.214 |
| GOBP_GLIAL_CELL_PROLIFERATION | -1.29 | 0.215 |
| GOBP_NEGATIVE_REGULATION_OF_MAP_KINASE_ACTIVITY | -1.28 | 0.215 |
| GOBP_HYDROGEN_PEROXIDE_METABOLIC_PROCESS | -1.28 | 0.215 |
| GOBP_EXIT_FROM_MITOSIS | -1.28 | 0.215 |
| GOMF_PROTEIN_HORMONE_RECEPTOR_ACTIVITY | -1.28 | 0.215 |
| GOBP_CELL_SUBSTRATE_JUNCTION_ORGANIZATION | -1.28 | 0.215 |
| GOBP_NEGATIVE_REGULATION_OF_SMOOTHENED_SIGNALING_PATHWAY | -1.28 | 0.215 |
| GOMF_GTPASE_ACTIVITY | -1.28 | 0.216 |
| GOCC_POSTSYNAPTIC_SPECIALIZATION_MEMBRANE | -1.28 | 0.216 |
| GOBP_ASPARTATE_FAMILY_AMINO_ACID_BIOSYNTHETIC_PROCESS | -1.28 | 0.217 |
| GOCC_DENDRITIC_SHAFT | -1.28 | 0.217 |
| GOBP_MEMORY | -1.28 | 0.217 |
| GOBP_MITOCHONDRIAL_GENE_EXPRESSION | -1.28 | 0.217 |
| GOBP_ACTOMYOSIN_STRUCTURE_ORGANIZATION | -1.28 | 0.218 |
| GOCC_ROUGH_ENDOPLASMIC_RETICULUM | -1.28 | 0.219 |
| GOBP_INTERFERON_ALPHA_PRODUCTION | -1.28 | 0.220 |
| GOBP_AORTA_DEVELOPMENT | -1.28 | 0.220 |
| GOBP_NUCLEOSIDE_BISPHOSPHATE_METABOLIC_PROCESS | -1.28 | 0.220 |
| GOBP_NEGATIVE_REGULATION_OF_VASCULATURE_DEVELOPMENT | -1.28 | 0.221 |
| GOBP_NEGATIVE_REGULATION_OF_KINASE_ACTIVITY | -1.28 | 0.221 |
| GOCC_SARCOPLASMIC_RETICULUM_MEMBRANE | -1.28 | 0.221 |
| GOBP_SARCOMERE_ORGANIZATION | -1.28 | 0.221 |
| GOMF_LIPASE_ACTIVITY | -1.28 | 0.222 |
| GOMF_PHOSPHATIDYLCHOLINE_TRANSPORTER_ACTIVITY | -1.28 | 0.222 |
| GOCC_MICROBODY | -1.28 | 0.222 |
| GOBP_VASCULAR_WOUND_HEALING | -1.28 | 0.222 |
| GOMF_MONOOXYGENASE_ACTIVITY | -1.28 | 0.222 |
| GOBP_TUMOR_NECROSIS_FACTOR_MEDIATED_SIGNALING_PATHWAY | -1.28 | 0.222 |
| GOBP_NEGATIVE_REGULATION_OF_BLOOD_VESSEL_ENDOTHELIAL_CELL_MIGRATION | -1.28 | 0.222 |
| GOBP_VIRAL_LIFE_CYCLE | -1.28 | 0.222 |
| GOBP_EXOCYTIC_PROCESS | -1.28 | 0.222 |
| GOMF_RRNA_BINDING | -1.28 | 0.224 |
| GOBP_RESPONSE_TO_HEAT | -1.28 | 0.224 |
| GOBP_PROTEIN_STABILIZATION | -1.28 | 0.224 |
| GOBP_GAMMA_DELTA_T_CELL_ACTIVATION | -1.28 | 0.224 |
| GOBP_REGULATION_OF_POST_TRANSLATIONAL_PROTEIN_MODIFICATION | -1.27 | 0.225 |
| GOBP_REGULATION_OF_HORMONE_METABOLIC_PROCESS | -1.27 | 0.225 |
| GOBP_PRODUCTION_OF_MOLECULAR_MEDIATOR_INVOLVED_IN_INFLAMMATORY_RESPONSE | -1.27 | 0.226 |
| GOBP_NEPHRON_MORPHOGENESIS | -1.27 | 0.226 |
| GOBP_PURINE_CONTAINING_COMPOUND_METABOLIC_PROCESS | -1.27 | 0.226 |
| GOBP_NEGATIVE_REGULATION_OF_RESPONSE_TO_ENDOPLASMIC_RETICULUM_STRESS | -1.27 | 0.226 |
| GOMF_PROTEIN_PHOSPHORYLATED_AMINO_ACID_BINDING | -1.27 | 0.226 |
| GOBP_CD4_POSITIVE_OR_CD8_POSITIVE_ALPHA_BETA_T_CELL_LINEAGE_COMMITMENT | -1.27 | 0.226 |
| GOBP_T_CELL_MEDIATED_IMMUNITY | -1.27 | 0.227 |
| GOBP_REGULATION_OF_FATTY_ACID_OXIDATION | -1.27 | 0.227 |
| GOBP_RETINA_LAYER_FORMATION | -1.27 | 0.228 |
| GOBP_LEUKOCYTE_TETHERING_OR_ROLLING | -1.27 | 0.228 |
| GOBP_POSITIVE_REGULATION_OF_LEUKOCYTE_APOPTOTIC_PROCESS | -1.27 | 0.228 |
| GOMF_LIPID_TRANSFER_ACTIVITY | -1.27 | 0.230 |
| GOBP_REGULATION_OF_GLIAL_CELL_PROLIFERATION | -1.27 | 0.233 |
| GOCC_CONTRACTILE_MUSCLE_FIBER | -1.27 | 0.232 |
| GOBP_CAMP_METABOLIC_PROCESS | -1.27 | 0.232 |
| GOMF_LIGASE_ACTIVITY_FORMING_CARBON_NITROGEN_BONDS | -1.27 | 0.233 |
| GOBP_HEMATOPOIETIC_PROGENITOR_CELL_DIFFERENTIATION | -1.27 | 0.233 |
| GOMF_GLUCOSYLTRANSFERASE_ACTIVITY | -1.27 | 0.233 |
| GOMF_CALMODULIN_BINDING | -1.27 | 0.234 |
| GOBP_MEMBRANE_DOCKING | -1.27 | 0.234 |
| GOBP_POSITIVE_REGULATION_OF_CELL_CYCLE_PHASE_TRANSITION | -1.27 | 0.234 |
| GOBP_SYNAPTIC_VESICLE_LUMEN_ACIDIFICATION | -1.27 | 0.235 |
| GOCC_EXCITATORY_SYNAPSE | -1.26 | 0.236 |
| GOMF_PURINE_RIBONUCLEOTIDE_TRANSMEMBRANE_TRANSPORTER_ACTIVITY | -1.26 | 0.236 |
| GOMF_VOLTAGE_GATED_CHANNEL_ACTIVITY | -1.26 | 0.236 |
| GOBP_HINDBRAIN_MORPHOGENESIS | -1.26 | 0.236 |
| GOBP_VIRAL_TRANSLATION | -1.26 | 0.237 |
| GOMF_FLIPPASE_ACTIVITY | -1.26 | 0.237 |
| GOBP_NEURAL_NUCLEUS_DEVELOPMENT | -1.26 | 0.238 |
| GOMF_VOLTAGE_GATED_MONOATOMIC_CATION_CHANNEL_ACTIVITY | -1.26 | 0.238 |
| GOMF_MONOATOMIC_ANION_CHANNEL_ACTIVITY | -1.26 | 0.238 |
| GOBP_REGULATION_OF_FATTY_ACID_METABOLIC_PROCESS | -1.26 | 0.238 |
| GOBP_GLUCAN_METABOLIC_PROCESS | -1.26 | 0.239 |
| GOBP_NEGATIVE_REGULATION_OF_LEUKOCYTE_CELL_CELL_ADHESION | -1.26 | 0.239 |
| GOBP_TYPE_2_IMMUNE_RESPONSE | -1.26 | 0.241 |
| GOBP_REGULATION_OF_ERYTHROCYTE_DIFFERENTIATION | -1.26 | 0.242 |
| GOMF_LIGAND_GATED_CHANNEL_ACTIVITY | -1.26 | 0.241 |
| GOBP_METANEPHRIC_NEPHRON_MORPHOGENESIS | -1.26 | 0.241 |
| GOMF_AMINO_ACID_MONOATOMIC_CATION_SYMPORTER_ACTIVITY | -1.26 | 0.242 |
| GOBP_REGULATION_OF_FIBROBLAST_GROWTH_FACTOR_RECEPTOR_SIGNALING_PATHWAY | -1.26 | 0.242 |
| GOBP_POSITIVE_REGULATION_OF_PROTEIN_SECRETION | -1.26 | 0.242 |
| GOCC_U5_SNRNP | -1.26 | 0.242 |
| GOBP_MITOCHONDRIAL_FUSION | -1.26 | 0.243 |
| GOBP_CYTOPLASMIC_PATTERN_RECOGNITION_RECEPTOR_SIGNALING_PATHWAY | -1.26 | 0.243 |
| GOBP_POSITIVE_REGULATION_OF_COLLAGEN_METABOLIC_PROCESS | -1.26 | 0.243 |
| GOBP_REGULATION_OF_TRANSCRIPTION_REGULATORY_REGION_DNA_BINDING | -1.26 | 0.244 |
| GOBP_NEGATIVE_REGULATION_OF_CELL_SUBSTRATE_JUNCTION_ORGANIZATION | -1.26 | 0.244 |
| GOBP_NEGATIVE_REGULATION_OF_AMIDE_METABOLIC_PROCESS | -1.26 | 0.244 |
| GOBP_POSITIVE_REGULATION_OF_LYMPHOCYTE_APOPTOTIC_PROCESS | -1.26 | 0.243 |
| GOBP_PROTEIN_LOCALIZATION_TO_CELL_SURFACE | -1.26 | 0.243 |
| GOBP_REGULATION_OF_HAIR_FOLLICLE_DEVELOPMENT | -1.26 | 0.244 |
| GOCC_EXTRINSIC_COMPONENT_OF_PLASMA_MEMBRANE | -1.26 | 0.244 |
| GOBP_SMOOTH_MUSCLE_CELL_APOPTOTIC_PROCESS | -1.26 | 0.244 |
| GOMF_MODIFIED_AMINO_ACID_BINDING | -1.26 | 0.244 |
| GOBP_DENTATE_GYRUS_DEVELOPMENT | -1.26 | 0.244 |
| GOBP_GLYCEROPHOSPHOLIPID_CATABOLIC_PROCESS | -1.26 | 0.244 |
| GOBP_POSITIVE_REGULATION_OF_GLUCOSE_TRANSMEMBRANE_TRANSPORT | -1.26 | 0.244 |
| GOBP_CELLULAR_RESPONSE_TO_FATTY_ACID | -1.26 | 0.244 |
| GOBP_SENSORY_PERCEPTION_OF_MECHANICAL_STIMULUS | -1.26 | 0.244 |
| GOBP_NEGATIVE_REGULATION_OF_HEMOPOIESIS | -1.26 | 0.244 |
| GOBP_COTRANSLATIONAL_PROTEIN_TARGETING_TO_MEMBRANE | -1.26 | 0.244 |
| GOBP_REGULATION_OF_PROTEIN_IMPORT_INTO_NUCLEUS | -1.26 | 0.244 |
| GOMF_UBIQUITIN_LIKE_PROTEIN_LIGASE_BINDING | -1.26 | 0.244 |
| GOBP_REGULATION_OF_HORMONE_SECRETION | -1.25 | 0.244 |
| GOBP_ESTABLISHMENT_OF_MITOCHONDRION_LOCALIZATION_MICROTUBULE_MEDIATED | -1.25 | 0.245 |
| GOCC_GOLGI_STACK | -1.25 | 0.246 |
| GOBP_POSITIVE_REGULATION_OF_PROTEIN_EXPORT_FROM_NUCLEUS | -1.25 | 0.246 |
| GOMF_INORGANIC_ANION_TRANSMEMBRANE_TRANSPORTER_ACTIVITY | -1.25 | 0.246 |
| GOBP_NEGATIVE_REGULATION_OF_ENDOTHELIAL_CELL_PROLIFERATION | -1.25 | 0.246 |
| GOMF_HYDROLASE_ACTIVITY_HYDROLYZING_O_GLYCOSYL_COMPOUNDS | -1.25 | 0.247 |
| GOBP_MYD88_DEPENDENT_TOLL_LIKE_RECEPTOR_SIGNALING_PATHWAY | -1.25 | 0.247 |
| GOMF_COPPER_ION_BINDING | -1.25 | 0.247 |
| GOBP_HORMONE_BIOSYNTHETIC_PROCESS | -1.25 | 0.247 |
| GOBP_GLIAL_CELL_DEVELOPMENT | -1.25 | 0.247 |
| GOCC_EXOCYTIC_VESICLE | -1.25 | 0.247 |
| GOBP_GLUTAMATE_METABOLIC_PROCESS | -1.25 | 0.247 |
| GOBP_INORGANIC_ANION_TRANSMEMBRANE_TRANSPORT | -1.25 | 0.247 |
| GOMF_MACROMOLECULE_TRANSMEMBRANE_TRANSPORTER_ACTIVITY | -1.25 | 0.247 |
| GOMF_OXIDOREDUCTASE_ACTIVITY_ACTING_ON_PAIRED_DONORS_WITH_INCORPORATION_OR_REDUCTION_OF_MOLECULAR_OXYGEN | -1.25 | 0.247 |
| GOBP_REGULATION_OF_CALCIUM_ION_TRANSMEMBRANE_TRANSPORT | -1.25 | 0.247 |
| GOBP_COLLATERAL_SPROUTING | -1.25 | 0.248 |
| GOBP_RESPONSE_TO_CADMIUM_ION | -1.25 | 0.248 |
| GOBP_EPHRIN_RECEPTOR_SIGNALING_PATHWAY | -1.25 | 0.248 |
| GOBP_ACTIN_FILAMENT_ORGANIZATION | -1.25 | 0.248 |
| GOBP_ESTABLISHMENT_OF_PROTEIN_LOCALIZATION_TO_MEMBRANE | -1.25 | 0.248 |
| GOBP_NEGATIVE_REGULATION_OF_MUSCLE_HYPERTROPHY | -1.25 | 0.248 |
| GOBP_ESTABLISHMENT_OF_PROTEIN_LOCALIZATION_TO_ORGANELLE | -1.25 | 0.248 |
| GOBP_CERAMIDE_METABOLIC_PROCESS | -1.25 | 0.249 |
| GOBP_LIMBIC_SYSTEM_DEVELOPMENT | -1.25 | 0.249 |
| GOBP_REGULATION_OF_SKELETAL_MUSCLE_TISSUE_DEVELOPMENT | -1.25 | 0.249 |

**NES: Normalized Enrichment Score, the enrichment score for the gene set after it has been normalized across analyzed gene sets. FDR q-value: False discovery rate, the estimated probability that the normalized enrichment score represents a false positive finding. Only gene sets with an FDR q-value ≤ 25% were included.**
