# Supplementary material for: Placental transcriptome profiling in congenital Chagas disease: gene networks associated with transmission
Source: Front Cell Infect Microbiol. 2026 Mar 18;16:1749307. doi: 10.3389/fcimb.2026.1749307 (PMC13038943; doi:10.3389/fcimb.2026.1749307)
Supplement: Supplementary file 7 [file Table3.docx]

**Supplementary Table 3. GSEA results using GO library for M+B- vs M-B- clinical groups.**

| Enriched gene sets in M+B- | NES | FDR q-val |
| --- | --- | --- |
| GOMF_CHEMOREPELLENT_ACTIVITY | 1.94 | 0.232 |
| GOMF_SEMAPHORIN_RECEPTOR_BINDING | 1.92 | 0.188 |
| GOBP_SENSORY_PERCEPTION_OF_SMELL | 1.91 | 0.170 |
| GOBP_FOREBRAIN_NEURON_DEVELOPMENT | 1.89 | 0.180 |
| GOBP_GAMMA_AMINOBUTYRIC_ACID_SIGNALING_PATHWAY | 1.89 | 0.153 |
| GOBP_SENSORY_PERCEPTION_OF_CHEMICAL_STIMULUS | 1.86 | 0.169 |

| Enriched gene sets in M-B- | NES | FDR q-val |
| --- | --- | --- |
| GOBP_ANTIMICROBIAL_HUMORAL_IMMUNE_RESPONSE_MEDIATED_BY_ANTIMICROBIAL_PEPTIDE | -2.49 | 0.000 |
| GOBP_ANTIMICROBIAL_HUMORAL_RESPONSE | -2.42 | 0.000 |
| GOCC_CYTOSOLIC_LARGE_RIBOSOMAL_SUBUNIT | -2.36 | 0.000 |
| GOBP_HUMORAL_IMMUNE_RESPONSE | -2.36 | 0.000 |
| GOCC_RIBOSOMAL_SUBUNIT | -2.34 | 0.000 |
| GOMF_STRUCTURAL_CONSTITUENT_OF_RIBOSOME | -2.29 | 0.000 |
| GOCC_TERTIARY_GRANULE_LUMEN | -2.27 | 0.000 |
| GOCC_CYTOSOLIC_RIBOSOME | -2.26 | 0.001 |
| GOCC_LARGE_RIBOSOMAL_SUBUNIT | -2.24 | 0.001 |
| GOBP_RESPONSE_TO_FUNGUS | -2.17 | 0.002 |
| GOBP_DEFENSE_RESPONSE_TO_GRAM_POSITIVE_BACTERIUM | -2.16 | 0.002 |
| GOBP_DEFENSE_RESPONSE_TO_FUNGUS | -2.16 | 0.002 |
| GOMF_CHEMOKINE_RECEPTOR_BINDING | -2.15 | 0.002 |
| GOBP_ACUTE_INFLAMMATORY_RESPONSE | -2.14 | 0.003 |
| GOMF_IMMUNE_RECEPTOR_ACTIVITY | -2.13 | 0.003 |
| GOBP_ANTIBACTERIAL_HUMORAL_RESPONSE | -2.10 | 0.006 |
| GOBP_REGULATION_OF_MACROPHAGE_ACTIVATION | -2.10 | 0.006 |
| GOBP_ANTIGEN_PROCESSING_AND_PRESENTATION_OF_PEPTIDE_ANTIGEN | -2.09 | 0.006 |
| GOMF_STRUCTURAL_CONSTITUENT_OF_CHROMATIN | -2.07 | 0.009 |
| GOCC_RIBOSOME | -2.07 | 0.010 |
| GOBP_REGULATION_OF_ACUTE_INFLAMMATORY_RESPONSE | -2.05 | 0.010 |
| GOBP_REGULATION_OF_MONOCYTE_CHEMOTAXIS | -2.05 | 0.010 |
| GOBP_ANTIGEN_PROCESSING_AND_PRESENTATION_OF_ENDOGENOUS_ANTIGEN | -2.05 | 0.010 |
| GOBP_POSITIVE_REGULATION_OF_ACUTE_INFLAMMATORY_RESPONSE | -2.05 | 0.010 |
| GOMF_LIPOPOLYSACCHARIDE_BINDING | -2.04 | 0.010 |
| GOBP_MACROPHAGE_CYTOKINE_PRODUCTION | -2.04 | 0.010 |
| GOBP_ANTIGEN_PROCESSING_AND_PRESENTATION | -2.04 | 0.010 |
| GOCC_SPECIFIC_GRANULE_LUMEN | -2.04 | 0.010 |
| GOBP_POSITIVE_REGULATION_OF_LIPID_STORAGE | -2.03 | 0.010 |
| GOCC_LUMENAL_SIDE_OF_MEMBRANE | -2.03 | 0.010 |
| GOCC_SMALL_RIBOSOMAL_SUBUNIT | -2.03 | 0.009 |
| GOBP_REGULATION_OF_LEUKOCYTE_MIGRATION | -2.03 | 0.009 |
| GOBP_FOAM_CELL_DIFFERENTIATION | -2.02 | 0.011 |
| GOBP_POSITIVE_REGULATION_OF_INFLAMMATORY_RESPONSE | -2.02 | 0.011 |
| GOCC_MHC_PROTEIN_COMPLEX | -2.01 | 0.012 |
| GOBP_NEGATIVE_REGULATION_OF_IMMUNE_EFFECTOR_PROCESS | -2.00 | 0.012 |
| GOBP_POSITIVE_REGULATION_OF_MACROPHAGE_ACTIVATION | -2.00 | 0.012 |
| GOBP_CELL_KILLING | -2.00 | 0.012 |
| GOCC_LUMENAL_SIDE_OF_ENDOPLASMIC_RETICULUM_MEMBRANE | -2.00 | 0.013 |
| GOBP_NEGATIVE_REGULATION_OF_HORMONE_SECRETION | -1.99 | 0.014 |
| GOBP_CELLULAR_RESPONSE_TO_MOLECULE_OF_BACTERIAL_ORIGIN | -1.97 | 0.017 |
| GOBP_REGULATION_OF_MONONUCLEAR_CELL_MIGRATION | -1.97 | 0.019 |
| GOBP_ANTIGEN_PROCESSING_AND_PRESENTATION_OF_ENDOGENOUS_PEPTIDE_ANTIGEN | -1.97 | 0.019 |
| GOBP_MYELOID_LEUKOCYTE_CYTOKINE_PRODUCTION | -1.96 | 0.019 |
| GOBP_CYTOPLASMIC_TRANSLATION | -1.96 | 0.020 |
| GOCC_CYTOSOLIC_SMALL_RIBOSOMAL_SUBUNIT | -1.96 | 0.020 |
| GOBP_ENDOCRINE_HORMONE_SECRETION | -1.96 | 0.020 |
| GOMF_MHC_PROTEIN_COMPLEX_BINDING | -1.95 | 0.020 |
| GOBP_REGULATION_OF_LEUKOCYTE_CHEMOTAXIS | -1.95 | 0.022 |
| GOBP_RESPONSE_TO_BACTERIUM | -1.95 | 0.022 |
| GOBP_ANTIGEN_PROCESSING_AND_PRESENTATION_OF_EXOGENOUS_ANTIGEN | -1.95 | 0.022 |
| GOBP_MONOCYTE_CHEMOTAXIS | -1.94 | 0.022 |
| GOBP_DEFENSE_RESPONSE_TO_BACTERIUM | -1.94 | 0.022 |
| GOBP_CELL_JUNCTION_DISASSEMBLY | -1.94 | 0.022 |
| GOBP_REGULATION_OF_MACROPHAGE_DERIVED_FOAM_CELL_DIFFERENTIATION | -1.94 | 0.023 |
| GOBP_LIPID_STORAGE | -1.93 | 0.023 |
| GOBP_PROTEIN_LOCALIZATION_TO_CENP_A_CONTAINING_CHROMATIN | -1.93 | 0.023 |
| GOBP_REGULATION_OF_LIPID_STORAGE | -1.93 | 0.024 |
| GOBP_NEGATIVE_REGULATION_OF_MONONUCLEAR_CELL_MIGRATION | -1.92 | 0.025 |
| GOBP_CYTOKINE_PRODUCTION_INVOLVED_IN_IMMUNE_RESPONSE | -1.92 | 0.025 |
| GOBP_B_CELL_MEDIATED_IMMUNITY | -1.92 | 0.025 |
| GOBP_ANTIGEN_PROCESSING_AND_PRESENTATION_OF_PEPTIDE_OR_POLYSACCHARIDE_ANTIGEN_VIA_MHC_CLASS_II | -1.92 | 0.025 |
| GOBP_DETECTION_OF_BIOTIC_STIMULUS | -1.91 | 0.026 |
| GOMF_CHEMOKINE_ACTIVITY | -1.91 | 0.027 |
| GOBP_NEGATIVE_REGULATION_OF_PEPTIDE_SECRETION | -1.91 | 0.028 |
| GOBP_NEGATIVE_REGULATION_OF_LEUKOCYTE_MIGRATION | -1.90 | 0.029 |
| GOBP_REGULATION_OF_PRODUCTION_OF_MOLECULAR_MEDIATOR_OF_IMMUNE_RESPONSE | -1.90 | 0.029 |
| GOBP_TOLL_LIKE_RECEPTOR_2_SIGNALING_PATHWAY | -1.90 | 0.029 |
| GOBP_REGULATION_OF_P38MAPK_CASCADE | -1.90 | 0.028 |
| GOBP_ANTIGEN_PROCESSING_AND_PRESENTATION_OF_EXOGENOUS_PEPTIDE_ANTIGEN | -1.90 | 0.030 |
| GOCC_SPECIFIC_GRANULE | -1.89 | 0.031 |
| GOBP_REGULATION_OF_MACROPHAGE_MIGRATION | -1.89 | 0.030 |
| GOBP_CELLULAR_RESPONSE_TO_BIOTIC_STIMULUS | -1.89 | 0.032 |
| GOBP_REGULATION_OF_COAGULATION | -1.89 | 0.032 |
| GOBP_ANTIGEN_PROCESSING_AND_PRESENTATION_OF_EXOGENOUS_PEPTIDE_ANTIGEN_VIA_MHC_CLASS_II | -1.89 | 0.032 |
| GOBP_CHOLESTEROL_STORAGE | -1.88 | 0.033 |
| GOBP_RESPONSE_TO_MOLECULE_OF_BACTERIAL_ORIGIN | -1.88 | 0.032 |
| GOBP_NEGATIVE_REGULATION_OF_CELL_ACTIVATION | -1.88 | 0.032 |
| GOBP_ACUTE_INFLAMMATORY_RESPONSE_TO_ANTIGENIC_STIMULUS | -1.88 | 0.035 |
| GOBP_INTERLEUKIN_4_PRODUCTION | -1.87 | 0.035 |
| GOBP_NEGATIVE_REGULATION_OF_MEGAKARYOCYTE_DIFFERENTIATION | -1.87 | 0.035 |
| GOBP_CELL_FATE_COMMITMENT_INVOLVED_IN_FORMATION_OF_PRIMARY_GERM_LAYER | -1.87 | 0.037 |
| GOBP_REGULATION_OF_BIOMINERAL_TISSUE_DEVELOPMENT | -1.87 | 0.037 |
| GOBP_ENDODERM_FORMATION | -1.86 | 0.038 |
| GOCC_PROTEIN_LIPID_COMPLEX | -1.86 | 0.039 |
| GOBP_NEGATIVE_REGULATION_OF_PRODUCTION_OF_MOLECULAR_MEDIATOR_OF_IMMUNE_RESPONSE | -1.86 | 0.039 |
| GOBP_POSITIVE_REGULATION_OF_MONOCYTE_CHEMOTAXIS | -1.86 | 0.039 |
| GOBP_INFLAMMATORY_RESPONSE_TO_ANTIGENIC_STIMULUS | -1.86 | 0.040 |
| GOBP_LEUKOCYTE_CHEMOTAXIS | -1.86 | 0.040 |
| GOMF_SERINE_HYDROLASE_ACTIVITY | -1.86 | 0.040 |
| GOBP_DISRUPTION_OF_ANATOMICAL_STRUCTURE_IN_ANOTHER_ORGANISM | -1.85 | 0.040 |
| GOBP_REGULATION_OF_CELL_KILLING | -1.85 | 0.040 |
| GOBP_TYPE_II_INTERFERON_PRODUCTION | -1.85 | 0.040 |
| GOBP_REGULATION_OF_BIOLOGICAL_PROCESS_INVOLVED_IN_SYMBIOTIC_INTERACTION | -1.85 | 0.040 |
| GOBP_MACROPHAGE_ACTIVATION | -1.85 | 0.042 |
| GOCC_VESICLE_LUMEN | -1.85 | 0.042 |
| GOBP_NEGATIVE_REGULATION_OF_CYTOKINE_PRODUCTION_INVOLVED_IN_IMMUNE_RESPONSE | -1.85 | 0.042 |
| GOBP_MAINTENANCE_OF_LOCATION | -1.84 | 0.044 |
| GOBP_CALCIUM_ION_TRANSPORT_INTO_CYTOSOL | -1.84 | 0.044 |
| GOBP_ANTIGEN_PROCESSING_AND_PRESENTATION_OF_PEPTIDE_ANTIGEN_VIA_MHC_CLASS_I | -1.83 | 0.047 |
| GOBP_NEGATIVE_REGULATION_OF_ADAPTIVE_IMMUNE_RESPONSE | -1.83 | 0.047 |
| GOBP_ADAPTIVE_IMMUNE_RESPONSE | -1.83 | 0.049 |
| GOBP_DETECTION_OF_EXTERNAL_BIOTIC_STIMULUS | -1.83 | 0.049 |
| GOBP_NEGATIVE_REGULATION_OF_LEUKOCYTE_MEDIATED_IMMUNITY | -1.82 | 0.051 |
| GOBP_POSITIVE_REGULATION_OF_PRODUCTION_OF_MOLECULAR_MEDIATOR_OF_IMMUNE_RESPONSE | -1.82 | 0.050 |
| GOBP_REGULATION_OF_ADAPTIVE_IMMUNE_RESPONSE | -1.82 | 0.052 |
| GOBP_TUMOR_NECROSIS_FACTOR_SUPERFAMILY_CYTOKINE_PRODUCTION | -1.82 | 0.052 |
| GOBP_COMPLEMENT_ACTIVATION | -1.82 | 0.053 |
| GOBP_NEGATIVE_REGULATION_OF_TYPE_II_INTERFERON_PRODUCTION | -1.82 | 0.052 |
| GOBP_ACYLGLYCEROL_HOMEOSTASIS | -1.82 | 0.052 |
| GOBP_REGULATION_OF_NITRIC_OXIDE_METABOLIC_PROCESS | -1.81 | 0.052 |
| GOMF_PEPTIDE_ANTIGEN_BINDING | -1.81 | 0.052 |
| GOBP_ENDOCRINE_PROCESS | -1.81 | 0.052 |
| GOBP_CELL_ADHESION_MEDIATED_BY_INTEGRIN | -1.81 | 0.052 |
| GOCC_PLATELET_ALPHA_GRANULE | -1.81 | 0.054 |
| GOCC_T_TUBULE | -1.81 | 0.054 |
| GOBP_MYELOID_LEUKOCYTE_ACTIVATION | -1.81 | 0.054 |
| GOMF_MHC_CLASS_II_PROTEIN_COMPLEX_BINDING | -1.80 | 0.055 |
| GOBP_REGULATION_OF_T_CELL_MEDIATED_CYTOTOXICITY | -1.80 | 0.056 |
| GOBP_REGULATION_OF_CELL_ADHESION_MEDIATED_BY_INTEGRIN | -1.80 | 0.056 |
| GOBP_B_CELL_HOMEOSTASIS | -1.80 | 0.058 |
| GOBP_REGULATION_OF_INFLAMMATORY_RESPONSE | -1.79 | 0.059 |
| GOBP_REGULATION_OF_IMMUNE_EFFECTOR_PROCESS | -1.79 | 0.059 |
| GOMF_PHOSPHATIDYLETHANOLAMINE_BINDING | -1.79 | 0.061 |
| GOBP_HUMORAL_IMMUNE_RESPONSE_MEDIATED_BY_CIRCULATING_IMMUNOGLOBULIN | -1.79 | 0.062 |
| GOBP_ADAPTIVE_IMMUNE_RESPONSE_BASED_ON_SOMATIC_RECOMBINATION_OF_IMMUNE_RECEPTORS_BUILT_FROM_IMMUNOGLOBULIN_SUPERFAMILY_DOMAINS | -1.78 | 0.063 |
| GOBP_NEGATIVE_REGULATION_OF_B_CELL_ACTIVATION | -1.78 | 0.065 |
| GOCC_NUCLEOSOME | -1.78 | 0.066 |
| GOBP_MYELOID_LEUKOCYTE_MIGRATION | -1.78 | 0.065 |
| GOBP_POSITIVE_REGULATION_OF_CYTOSOLIC_CALCIUM_ION_CONCENTRATION | -1.78 | 0.066 |
| GOBP_NEGATIVE_REGULATION_OF_COAGULATION | -1.78 | 0.066 |
| GOBP_NEGATIVE_REGULATION_OF_MYELOID_CELL_DIFFERENTIATION | -1.78 | 0.067 |
| GOCC_CHROMOSOME_CENTROMERIC_CORE_DOMAIN | -1.77 | 0.070 |
| GOBP_POSITIVE_REGULATION_OF_MYELOID_LEUKOCYTE_CYTOKINE_PRODUCTION_INVOLVED_IN_IMMUNE_RESPONSE | -1.77 | 0.072 |
| GOBP_LEUKOCYTE_MIGRATION | -1.76 | 0.076 |
| GOBP_PHASIC_SMOOTH_MUSCLE_CONTRACTION | -1.76 | 0.076 |
| GOMF_ANTIGEN_BINDING | -1.76 | 0.076 |
| GOBP_POSITIVE_REGULATION_OF_MACROPHAGE_CYTOKINE_PRODUCTION | -1.76 | 0.076 |
| GOBP_REGULATION_OF_ENDOCRINE_PROCESS | -1.76 | 0.075 |
| GOCC_TERTIARY_GRANULE | -1.76 | 0.075 |
| GOBP_REGULATION_OF_LYMPHOCYTE_CHEMOTAXIS | -1.76 | 0.075 |
| GOBP_POSITIVE_REGULATION_OF_T_CELL_MEDIATED_CYTOTOXICITY | -1.76 | 0.075 |
| GOBP_REGULATION_OF_INFLAMMATORY_RESPONSE_TO_ANTIGENIC_STIMULUS | -1.76 | 0.074 |
| GOBP_DENDRITIC_CELL_DIFFERENTIATION | -1.75 | 0.077 |
| GOCC_EXTERNAL_SIDE_OF_PLASMA_MEMBRANE | -1.75 | 0.079 |
| GOBP_REGULATION_OF_HUMORAL_IMMUNE_RESPONSE | -1.75 | 0.079 |
| GOBP_POSITIVE_REGULATION_OF_CYTOKINE_PRODUCTION_INVOLVED_IN_IMMUNE_RESPONSE | -1.75 | 0.080 |
| GOBP_P38MAPK_CASCADE | -1.75 | 0.080 |
| GOBP_ADIPOSE_TISSUE_DEVELOPMENT | -1.75 | 0.080 |
| GOCC_BLOOD_MICROPARTICLE | -1.75 | 0.080 |
| GOBP_NEGATIVE_REGULATION_OF_TOLL_LIKE_RECEPTOR_SIGNALING_PATHWAY | -1.75 | 0.079 |
| GOBP_PRODUCTION_OF_MOLECULAR_MEDIATOR_OF_IMMUNE_RESPONSE | -1.74 | 0.082 |
| GOBP_GLUCOSAMINE_CONTAINING_COMPOUND_METABOLIC_PROCESS | -1.74 | 0.083 |
| GOBP_REGULATION_OF_CARDIOCYTE_DIFFERENTIATION | -1.74 | 0.083 |
| GOBP_AMINE_BIOSYNTHETIC_PROCESS | -1.74 | 0.084 |
| GOBP_ENDOTHELIAL_CELL_APOPTOTIC_PROCESS | -1.74 | 0.084 |
| GOBP_REGULATION_OF_B_CELL_MEDIATED_IMMUNITY | -1.74 | 0.084 |
| GOBP_PROTEIN_CONTAINING_COMPLEX_REMODELING | -1.73 | 0.085 |
| GOBP_POSITIVE_REGULATION_OF_CELL_KILLING | -1.73 | 0.087 |
| GOBP_NEGATIVE_REGULATION_OF_IMMUNE_SYSTEM_PROCESS | -1.73 | 0.088 |
| GOBP_INNERVATION | -1.73 | 0.090 |
| GOBP_REGULATION_OF_WOUND_HEALING | -1.73 | 0.090 |
| GOBP_IMMUNE_EFFECTOR_PROCESS | -1.73 | 0.090 |
| GOBP_CELL_CHEMOTAXIS | -1.72 | 0.091 |
| GOBP_POSITIVE_REGULATION_OF_TUMOR_NECROSIS_FACTOR_SUPERFAMILY_CYTOKINE_PRODUCTION | -1.72 | 0.090 |
| GOBP_POSITIVE_REGULATION_OF_SPROUTING_ANGIOGENESIS | -1.72 | 0.091 |
| GOBP_REGULATION_OF_LIPOPOLYSACCHARIDE_MEDIATED_SIGNALING_PATHWAY | -1.72 | 0.090 |
| GOBP_CARDIAC_NEURAL_CREST_CELL_DIFFERENTIATION_INVOLVED_IN_HEART_DEVELOPMENT | -1.72 | 0.090 |
| GOCC_STRIATED_MUSCLE_THIN_FILAMENT | -1.72 | 0.094 |
| GOBP_INTRACELLULAR_ZINC_ION_HOMEOSTASIS | -1.72 | 0.095 |
| GOBP_T_CELL_MEDIATED_CYTOTOXICITY | -1.72 | 0.095 |
| GOBP_REGULATION_OF_CHEMOTAXIS | -1.71 | 0.095 |
| GOBP_NEGATIVE_REGULATION_OF_WOUND_HEALING | -1.71 | 0.095 |
| GOBP_NEGATIVE_REGULATION_OF_SMOOTH_MUSCLE_CELL_PROLIFERATION | -1.71 | 0.095 |
| GOBP_POSITIVE_REGULATION_OF_T_CELL_MEDIATED_IMMUNITY | -1.71 | 0.095 |
| GOBP_POSITIVE_REGULATION_OF_CHEMOKINE_PRODUCTION | -1.71 | 0.095 |
| GOBP_POSITIVE_REGULATION_OF_COAGULATION | -1.71 | 0.098 |
| GOBP_REGULATION_OF_LYMPHOCYTE_MEDIATED_IMMUNITY | -1.71 | 0.099 |
| GOBP_NEGATIVE_REGULATION_OF_SMAD_PROTEIN_SIGNAL_TRANSDUCTION | -1.71 | 0.099 |
| GOBP_COMPLEMENT_ACTIVATION_CLASSICAL_PATHWAY | -1.71 | 0.099 |
| GOBP_POSITIVE_REGULATION_OF_P38MAPK_CASCADE | -1.70 | 0.101 |
| GOCC_U5_SNRNP | -1.70 | 0.101 |
| GOBP_T_CELL_CHEMOTAXIS | -1.70 | 0.101 |
| GOBP_REGULATION_OF_LEUKOCYTE_ADHESION_TO_VASCULAR_ENDOTHELIAL_CELL | -1.70 | 0.100 |
| GOBP_NEGATIVE_REGULATION_OF_LIPID_CATABOLIC_PROCESS | -1.70 | 0.100 |
| GOMF_E_BOX_BINDING | -1.70 | 0.101 |
| GOBP_RESPONSE_TO_AUDITORY_STIMULUS | -1.70 | 0.101 |
| GOBP_POSITIVE_REGULATION_OF_VIRAL_LIFE_CYCLE | -1.70 | 0.102 |
| GOBP_POSITIVE_REGULATION_OF_LEUKOCYTE_CHEMOTAXIS | -1.70 | 0.103 |
| GOBP_MAINTENANCE_OF_PROTEIN_LOCATION | -1.69 | 0.104 |
| GOBP_MUSCLE_HYPERTROPHY_IN_RESPONSE_TO_STRESS | -1.69 | 0.104 |
| GOBP_REGULATION_OF_BONE_MINERALIZATION | -1.69 | 0.104 |
| GOBP_POSITIVE_REGULATION_OF_LEUKOCYTE_MIGRATION | -1.69 | 0.105 |
| GOMF_MOLECULAR_SEQUESTERING_ACTIVITY | -1.69 | 0.106 |
| GOMF_STRUCTURAL_CONSTITUENT_OF_MUSCLE | -1.69 | 0.107 |
| GOBP_NEGATIVE_REGULATION_OF_IMMUNE_RESPONSE | -1.69 | 0.109 |
| GOMF_POLYSACCHARIDE_BINDING | -1.69 | 0.109 |
| GOBP_LEUKOCYTE_PROLIFERATION | -1.68 | 0.110 |
| GOMF_CYTOKINE_ACTIVITY | -1.68 | 0.111 |
| GOBP_NEGATIVE_REGULATION_OF_VASCULAR_ASSOCIATED_SMOOTH_MUSCLE_CELL_PROLIFERATION | -1.68 | 0.112 |
| GOBP_CALCIUM_INDEPENDENT_CELL_CELL_ADHESION_VIA_PLASMA_MEMBRANE_CELL_ADHESION_MOLECULES | -1.68 | 0.112 |
| GOBP_REGULATION_OF_CHOLESTEROL_STORAGE | -1.68 | 0.112 |
| GOBP_REGULATION_OF_CELLULAR_EXTRAVASATION | -1.68 | 0.114 |
| GOBP_REGULATION_OF_T_CELL_MEDIATED_IMMUNITY | -1.68 | 0.114 |
| GOBP_LABYRINTHINE_LAYER_BLOOD_VESSEL_DEVELOPMENT | -1.68 | 0.114 |
| GOBP_COLLAGEN_CATABOLIC_PROCESS | -1.68 | 0.116 |
| GOBP_POSITIVE_REGULATION_OF_MONONUCLEAR_CELL_MIGRATION | -1.67 | 0.116 |
| GOBP_POSITIVE_REGULATION_OF_CYTOKINE_PRODUCTION | -1.67 | 0.119 |
| GOBP_MOTOR_NEURON_APOPTOTIC_PROCESS | -1.67 | 0.118 |
| GOCC_PLATELET_ALPHA_GRANULE_LUMEN | -1.67 | 0.120 |
| GOBP_NEGATIVE_REGULATION_OF_SECRETION | -1.67 | 0.120 |
| GOCC_SECONDARY_LYSOSOME | -1.67 | 0.121 |
| GOBP_ORGAN_OR_TISSUE_SPECIFIC_IMMUNE_RESPONSE | -1.67 | 0.122 |
| GOBP_MESENCHYME_MORPHOGENESIS | -1.66 | 0.123 |
| GOBP_NEGATIVE_REGULATION_OF_RESPONSE_TO_EXTERNAL_STIMULUS | -1.66 | 0.125 |
| GOBP_B_CELL_PROLIFERATION | -1.66 | 0.127 |
| GOMF_GLYCOSAMINOGLYCAN_BINDING | -1.66 | 0.127 |
| GOBP_LYMPHOCYTE_MEDIATED_IMMUNITY | -1.66 | 0.127 |
| GOBP_OVULATION_CYCLE | -1.66 | 0.128 |
| GOBP_HOMOTYPIC_CELL_CELL_ADHESION | -1.66 | 0.129 |
| GOBP_CHEMOKINE_PRODUCTION | -1.66 | 0.129 |
| GOBP_HEMOGLOBIN_METABOLIC_PROCESS | -1.66 | 0.129 |
| GOBP_POSITIVE_REGULATION_OF_T_CELL_PROLIFERATION | -1.65 | 0.129 |
| GOBP_NEGATIVE_REGULATION_OF_LYMPHOCYTE_ACTIVATION | -1.65 | 0.129 |
| GOBP_PLATELET_AGGREGATION | -1.65 | 0.130 |
| GOBP_POSITIVE_REGULATION_OF_INTERLEUKIN_4_PRODUCTION | -1.65 | 0.133 |
| GOMF_CYTOKINE_BINDING | -1.65 | 0.133 |
| GOBP_DEFENSE_RESPONSE_TO_GRAM_NEGATIVE_BACTERIUM | -1.65 | 0.132 |
| GOBP_REGULATION_OF_PLASMA_LIPOPROTEIN_PARTICLE_LEVELS | -1.65 | 0.136 |
| GOBP_INTERLEUKIN_10_PRODUCTION | -1.65 | 0.136 |
| GOCC_MYOFILAMENT | -1.65 | 0.135 |
| GOBP_NEGATIVE_REGULATION_OF_CHEMOTAXIS | -1.64 | 0.136 |
| GOCC_ER_TO_GOLGI_TRANSPORT_VESICLE_MEMBRANE | -1.64 | 0.136 |
| GOBP_CYTOPLASMIC_SEQUESTERING_OF_PROTEIN | -1.64 | 0.135 |
| GOMF_RRNA_BINDING | -1.64 | 0.136 |
| GOBP_REGULATION_OF_DIGESTIVE_SYSTEM_PROCESS | -1.64 | 0.137 |
| GOBP_NEGATIVE_REGULATION_OF_TUMOR_NECROSIS_FACTOR_SUPERFAMILY_CYTOKINE_PRODUCTION | -1.64 | 0.137 |
| GOBP_NEGATIVE_REGULATION_OF_LIPID_STORAGE | -1.64 | 0.137 |
| GOMF_PEPTIDE_BINDING | -1.64 | 0.137 |
| GOBP_POSITIVE_REGULATION_OF_RECEPTOR_SIGNALING_PATHWAY_VIA_STAT | -1.64 | 0.137 |
| GOBP_HYPERSENSITIVITY | -1.64 | 0.139 |
| GOBP_NEGATIVE_REGULATION_OF_LYMPHOCYTE_MEDIATED_IMMUNITY | -1.64 | 0.140 |
| GOMF_CARBOHYDRATE_TRANSMEMBRANE_TRANSPORTER_ACTIVITY | -1.63 | 0.143 |
| GOBP_STRIATED_MUSCLE_ADAPTATION | -1.63 | 0.143 |
| GOBP_PHENOL_CONTAINING_COMPOUND_BIOSYNTHETIC_PROCESS | -1.63 | 0.144 |
| GOBP_MULTI_MULTICELLULAR_ORGANISM_PROCESS | -1.63 | 0.144 |
| GOBP_POSITIVE_REGULATION_OF_CHEMOTAXIS | -1.63 | 0.144 |
| GOBP_TYROSINE_PHOSPHORYLATION_OF_STAT_PROTEIN | -1.63 | 0.146 |
| GOBP_REGULATION_OF_MIRNA_METABOLIC_PROCESS | -1.63 | 0.147 |
| GOBP_REGULATION_OF_FAT_CELL_DIFFERENTIATION | -1.63 | 0.146 |
| GOBP_REGULATION_OF_CELL_CELL_ADHESION_MEDIATED_BY_CADHERIN | -1.63 | 0.146 |
| GOBP_REGULATION_OF_COMPLEMENT_ACTIVATION | -1.63 | 0.146 |
| GOBP_REGULATION_OF_RELEASE_OF_SEQUESTERED_CALCIUM_ION_INTO_CYTOSOL | -1.63 | 0.146 |
| GOBP_POSITIVE_REGULATION_OF_G_PROTEIN_COUPLED_RECEPTOR_SIGNALING_PATHWAY | -1.63 | 0.146 |
| GOBP_T_HELPER_17_TYPE_IMMUNE_RESPONSE | -1.63 | 0.146 |
| GOBP_OVULATION_CYCLE_PROCESS | -1.62 | 0.147 |
| GOBP_NEGATIVE_REGULATION_OF_CELL_KILLING | -1.62 | 0.147 |
| GOBP_POSITIVE_REGULATION_OF_CELL_ADHESION_MEDIATED_BY_INTEGRIN | -1.62 | 0.148 |
| GOBP_REGULATION_OF_LIPID_CATABOLIC_PROCESS | -1.62 | 0.148 |
| GOBP_POSITIVE_REGULATION_OF_TYROSINE_PHOSPHORYLATION_OF_STAT_PROTEIN | -1.62 | 0.147 |
| GOBP_REGULATION_OF_LEUKOCYTE_PROLIFERATION | -1.62 | 0.147 |
| GOBP_NUCLEOTIDE_TRANSMEMBRANE_TRANSPORT | -1.62 | 0.147 |
| GOBP_POSITIVE_REGULATION_OF_ERK1_AND_ERK2_CASCADE | -1.62 | 0.147 |
| GOMF_TRANSCRIPTION_REGULATOR_INHIBITOR_ACTIVITY | -1.62 | 0.148 |
| GOBP_PROTEIN_N_LINKED_GLYCOSYLATION_VIA_ASPARAGINE | -1.62 | 0.149 |
| GOBP_POSITIVE_REGULATION_OF_VASCULATURE_DEVELOPMENT | -1.62 | 0.148 |
| GOBP_POSITIVE_REGULATION_OF_HUMORAL_IMMUNE_RESPONSE | -1.62 | 0.148 |
| GOBP_REGULATION_OF_TOLL_LIKE_RECEPTOR_4_SIGNALING_PATHWAY | -1.62 | 0.150 |
| GOBP_DEVELOPMENT_OF_PRIMARY_FEMALE_SEXUAL_CHARACTERISTICS | -1.62 | 0.151 |
| GOBP_POSITIVE_REGULATION_OF_IMMUNE_EFFECTOR_PROCESS | -1.62 | 0.150 |
| GOBP_POSITIVE_REGULATION_OF_CIRCADIAN_RHYTHM | -1.62 | 0.150 |
| GOBP_NEGATIVE_REGULATION_OF_INTRINSIC_APOPTOTIC_SIGNALING_PATHWAY | -1.61 | 0.152 |
| GOBP_REACTIVE_NITROGEN_SPECIES_METABOLIC_PROCESS | -1.61 | 0.152 |
| GOBP_REGULATION_OF_FATTY_ACID_BIOSYNTHETIC_PROCESS | -1.61 | 0.153 |
| GOCC_I_BAND | -1.61 | 0.153 |
| GOBP_POSITIVE_REGULATION_OF_LIPID_LOCALIZATION | -1.61 | 0.153 |
| GOBP_STEROID_HORMONE_SECRETION | -1.61 | 0.152 |
| GOBP_BIOMINERAL_TISSUE_DEVELOPMENT | -1.61 | 0.153 |
| GOBP_RESPONSE_TO_INTERLEUKIN_1 | -1.61 | 0.152 |
| GOBP_POSITIVE_REGULATION_OF_LEUKOCYTE_PROLIFERATION | -1.61 | 0.156 |
| GOBP_MONONUCLEAR_CELL_MIGRATION | -1.61 | 0.158 |
| GOBP_REGULATION_OF_MYELOID_CELL_DIFFERENTIATION | -1.61 | 0.157 |
| GOBP_REGULATION_OF_TOLL_LIKE_RECEPTOR_SIGNALING_PATHWAY | -1.60 | 0.158 |
| GOBP_LYMPHOCYTE_ACTIVATION_INVOLVED_IN_IMMUNE_RESPONSE | -1.60 | 0.158 |
| GOMF_CYTOKINE_RECEPTOR_ACTIVITY | -1.60 | 0.160 |
| GOBP_REGULATION_OF_CELL_ACTIVATION | -1.60 | 0.159 |
| GOBP_LEUKOCYTE_MEDIATED_CYTOTOXICITY | -1.60 | 0.159 |
| GOBP_REGULATION_OF_B_CELL_APOPTOTIC_PROCESS | -1.60 | 0.159 |
| GOBP_POSITIVE_REGULATION_OF_LEUKOCYTE_MEDIATED_IMMUNITY | -1.60 | 0.160 |
| GOBP_POSITIVE_REGULATION_OF_BIOMINERAL_TISSUE_DEVELOPMENT | -1.60 | 0.159 |
| GOBP_LEUKOCYTE_TETHERING_OR_ROLLING | -1.60 | 0.159 |
| GOBP_MACROPHAGE_MIGRATION | -1.60 | 0.159 |
| GOBP_MYELOID_DENDRITIC_CELL_ACTIVATION | -1.60 | 0.159 |
| GOBP_POSITIVE_REGULATION_OF_ADAPTIVE_IMMUNE_RESPONSE | -1.60 | 0.161 |
| GOBP_POSITIVE_REGULATION_OF_TYPE_II_INTERFERON_PRODUCTION | -1.60 | 0.161 |
| GOBP_REGULATION_OF_MEMBRANE_PROTEIN_ECTODOMAIN_PROTEOLYSIS | -1.60 | 0.160 |
| GOCC_FICOLIN_1_RICH_GRANULE_LUMEN | -1.60 | 0.162 |
| GOBP_T_HELPER_17_CELL_DIFFERENTIATION | -1.59 | 0.164 |
| GOBP_REGULATION_OF_RESPONSE_TO_WOUNDING | -1.59 | 0.165 |
| GOBP_CELLULAR_OXIDANT_DETOXIFICATION | -1.59 | 0.165 |
| GOBP_POSITIVE_REGULATION_OF_B_CELL_MEDIATED_IMMUNITY | -1.59 | 0.167 |
| GOMF_FIBRONECTIN_BINDING | -1.59 | 0.169 |
| GOBP_DIGESTIVE_SYSTEM_PROCESS | -1.59 | 0.170 |
| GOBP_CELLULAR_RESPONSE_TO_CADMIUM_ION | -1.59 | 0.169 |
| GOBP_ACUTE_PHASE_RESPONSE | -1.59 | 0.170 |
| GOBP_CELL_SURFACE_PATTERN_RECOGNITION_RECEPTOR_SIGNALING_PATHWAY | -1.59 | 0.170 |
| GOBP_CELLULAR_RESPONSE_TO_OXYGEN_LEVELS | -1.59 | 0.170 |
| GOBP_REGULATION_OF_SMAD_PROTEIN_SIGNAL_TRANSDUCTION | -1.59 | 0.169 |
| GOBP_REGULATION_OF_MEGAKARYOCYTE_DIFFERENTIATION | -1.58 | 0.169 |
| GOBP_VASOCONSTRICTION | -1.58 | 0.169 |
| GOBP_NEGATIVE_REGULATION_OF_FAT_CELL_DIFFERENTIATION | -1.58 | 0.170 |
| GOBP_VASCULAR_ASSOCIATED_SMOOTH_MUSCLE_CELL_DIFFERENTIATION | -1.58 | 0.171 |
| GOMF_PROTEIN_HETERODIMERIZATION_ACTIVITY | -1.58 | 0.171 |
| GOBP_B_CELL_APOPTOTIC_PROCESS | -1.58 | 0.171 |
| GOBP_STRIATED_MUSCLE_CELL_DEVELOPMENT | -1.58 | 0.171 |
| GOBP_SMOOTH_MUSCLE_CONTRACTION | -1.58 | 0.171 |
| GOBP_REGULATION_OF_ALPHA_BETA_T_CELL_ACTIVATION | -1.58 | 0.171 |
| GOBP_REGULATION_OF_LEUKOCYTE_MEDIATED_IMMUNITY | -1.58 | 0.172 |
| GOBP_ANTERIOR_POSTERIOR_AXIS_SPECIFICATION | -1.58 | 0.171 |
| GOBP_IMMUNE_RESPONSE_REGULATING_CELL_SURFACE_RECEPTOR_SIGNALING_PATHWAY | -1.58 | 0.171 |
| GOBP_INTEGRATED_STRESS_RESPONSE_SIGNALING | -1.58 | 0.171 |
| GOBP_NEGATIVE_REGULATION_OF_B_CELL_PROLIFERATION | -1.58 | 0.170 |
| GOMF_SOLUTE_INORGANIC_ANION_ANTIPORTER_ACTIVITY | -1.58 | 0.171 |
| GOCC_ENDOCYTIC_VESICLE_LUMEN | -1.58 | 0.171 |
| GOBP_NEGATIVE_REGULATION_OF_NATURAL_KILLER_CELL_MEDIATED_IMMUNITY | -1.58 | 0.171 |
| GOBP_REGULATION_OF_OSTEOBLAST_DIFFERENTIATION | -1.58 | 0.171 |
| GOBP_NEGATIVE_REGULATION_OF_LOCOMOTION | -1.58 | 0.171 |
| GOBP_CELL_SURFACE_RECEPTOR_SIGNALING_PATHWAY_VIA_STAT | -1.58 | 0.171 |
| GOBP_NEGATIVE_REGULATION_OF_REPRODUCTIVE_PROCESS | -1.57 | 0.174 |
| GOBP_NEUROTRANSMITTER_REUPTAKE | -1.57 | 0.173 |
| GOBP_REGULATION_OF_DEFENSE_RESPONSE_TO_BACTERIUM | -1.57 | 0.173 |
| GOBP_POSITIVE_REGULATION_OF_NITRIC_OXIDE_METABOLIC_PROCESS | -1.57 | 0.173 |
| GOBP_BONE_MINERALIZATION | -1.57 | 0.174 |
| GOBP_REGULATION_OF_OSSIFICATION | -1.57 | 0.176 |
| GOBP_LEUKOCYTE_ADHESION_TO_VASCULAR_ENDOTHELIAL_CELL | -1.57 | 0.176 |
| GOBP_MOTOR_NEURON_AXON_GUIDANCE | -1.57 | 0.175 |
| GOBP_ZYMOGEN_ACTIVATION | -1.57 | 0.175 |
| GOMF_LIPASE_ACTIVATOR_ACTIVITY | -1.57 | 0.176 |
| GOCC_SARCOPLASMIC_RETICULUM_MEMBRANE | -1.57 | 0.176 |
| GOBP_CELLULAR_RESPONSE_TO_TOXIC_SUBSTANCE | -1.57 | 0.176 |
| GOBP_ARTERY_MORPHOGENESIS | -1.57 | 0.176 |
| GOCC_FICOLIN_1_RICH_GRANULE | -1.57 | 0.176 |
| GOBP_POSITIVE_REGULATION_OF_CELL_ADHESION | -1.57 | 0.176 |
| GOBP_PRODUCTION_OF_MOLECULAR_MEDIATOR_INVOLVED_IN_INFLAMMATORY_RESPONSE | -1.57 | 0.176 |
| GOBP_NEUTROPHIL_CHEMOTAXIS | -1.57 | 0.177 |
| GOBP_POSITIVE_REGULATION_OF_REACTIVE_OXYGEN_SPECIES_METABOLIC_PROCESS | -1.57 | 0.176 |
| GOBP_TELOMERASE_RNA_LOCALIZATION | -1.56 | 0.177 |
| GOBP_T_CELL_MEDIATED_IMMUNITY | -1.56 | 0.178 |
| GOBP_DETOXIFICATION | -1.56 | 0.178 |
| GOBP_PROTEIN_LIPID_COMPLEX_ORGANIZATION | -1.56 | 0.178 |
| GOBP_POSITIVE_REGULATION_OF_PEPTIDYL_SERINE_PHOSPHORYLATION | -1.56 | 0.179 |
| GOCC_SPECIFIC_GRANULE_MEMBRANE | -1.56 | 0.179 |
| GOBP_LYMPHOCYTE_CHEMOTAXIS | -1.56 | 0.180 |
| GOBP_OSTEOBLAST_PROLIFERATION | -1.56 | 0.180 |
| GOBP_NUCLEOTIDE_SUGAR_METABOLIC_PROCESS | -1.56 | 0.180 |
| GOCC_COPII_COATED_ER_TO_GOLGI_TRANSPORT_VESICLE | -1.56 | 0.181 |
| GOBP_REGULATION_OF_LIPID_LOCALIZATION | -1.56 | 0.181 |
| GOCC_ENDOPLASMIC_RETICULUM_PROTEIN_CONTAINING_COMPLEX | -1.56 | 0.183 |
| GOCC_ORGANELLAR_RIBOSOME | -1.55 | 0.187 |
| GOBP_PHAGOCYTOSIS_RECOGNITION | -1.55 | 0.187 |
| GOBP_PLASMINOGEN_ACTIVATION | -1.55 | 0.186 |
| GOBP_TOLL_LIKE_RECEPTOR_4_SIGNALING_PATHWAY | -1.55 | 0.188 |
| GOBP_NEGATIVE_REGULATION_OF_ENDOTHELIAL_CELL_APOPTOTIC_PROCESS | -1.55 | 0.188 |
| GOBP_MYELOID_CELL_DIFFERENTIATION | -1.55 | 0.189 |
| GOBP_NEGATIVE_REGULATION_OF_LEUKOCYTE_PROLIFERATION | -1.55 | 0.190 |
| GOBP_POSITIVE_REGULATION_OF_INTERLEUKIN_17_PRODUCTION | -1.55 | 0.191 |
| GOBP_REGULATION_OF_HEMOPOIESIS | -1.55 | 0.190 |
| GOBP_CELLULAR_RESPONSE_TO_LIPID | -1.55 | 0.191 |
| GOCC_SMN_SM_PROTEIN_COMPLEX | -1.55 | 0.191 |
| GOBP_REGULATION_OF_PHOSPHOLIPASE_C_ACTIVITY | -1.55 | 0.192 |
| GOBP_REGULATION_OF_PLATELET_AGGREGATION | -1.55 | 0.194 |
| GOBP_NUCLEOTIDE_SUGAR_BIOSYNTHETIC_PROCESS | -1.54 | 0.193 |
| GOCC_PLATELET_ALPHA_GRANULE_MEMBRANE | -1.54 | 0.194 |
| GOBP_POSITIVE_REGULATION_OF_PHAGOCYTOSIS | -1.54 | 0.194 |
| GOBP_ONE_CARBON_METABOLIC_PROCESS | -1.54 | 0.197 |
| GOBP_SMOOTH_MUSCLE_TISSUE_DEVELOPMENT | -1.54 | 0.197 |
| GOBP_ENDODERM_DEVELOPMENT | -1.54 | 0.197 |
| GOBP_REGULATION_OF_CARTILAGE_DEVELOPMENT | -1.54 | 0.198 |
| GOMF_CYTOKINE_RECEPTOR_BINDING | -1.54 | 0.197 |
| GOBP_NEGATIVE_REGULATION_OF_BMP_SIGNALING_PATHWAY | -1.54 | 0.197 |
| GOBP_POSITIVE_REGULATION_OF_MIRNA_METABOLIC_PROCESS | -1.54 | 0.199 |
| GOMF_COMPLEMENT_BINDING | -1.54 | 0.199 |
| GOCC_PROTEASOME_ACCESSORY_COMPLEX | -1.54 | 0.199 |
| GOBP_HYDROGEN_PEROXIDE_CATABOLIC_PROCESS | -1.54 | 0.199 |
| GOBP_VASCULAR_WOUND_HEALING | -1.53 | 0.204 |
| GOBP_POSITIVE_REGULATION_OF_DEFENSE_RESPONSE | -1.53 | 0.204 |
| GOBP_RESPONSE_TO_MANGANESE_ION | -1.53 | 0.204 |
| GOBP_POSITIVE_REGULATION_OF_BEHAVIOR | -1.53 | 0.204 |
| GOMF_HORMONE_ACTIVITY | -1.53 | 0.204 |
| GOBP_RESPONSE_TO_CHEMOKINE | -1.53 | 0.204 |
| GOBP_NEGATIVE_REGULATION_OF_SIGNAL_TRANSDUCTION_BY_P53_CLASS_MEDIATOR | -1.53 | 0.205 |
| GOMF_ANTIOXIDANT_ACTIVITY | -1.53 | 0.208 |
| GOBP_CELLULAR_RESPONSE_TO_INTERLEUKIN_1 | -1.53 | 0.210 |
| GOCC_MITOCHONDRIAL_LARGE_RIBOSOMAL_SUBUNIT | -1.53 | 0.211 |
| GOBP_CHEMOKINE_C_X_C_MOTIF_LIGAND_2_PRODUCTION | -1.53 | 0.212 |
| GOBP_ICOSANOID_BIOSYNTHETIC_PROCESS | -1.52 | 0.212 |
| GOBP_CARDIAC_EPITHELIAL_TO_MESENCHYMAL_TRANSITION | -1.52 | 0.214 |
| GOCC_SNO_S_RNA_CONTAINING_RIBONUCLEOPROTEIN_COMPLEX | -1.52 | 0.214 |
| GOBP_REGULATION_OF_CYTOSOLIC_CALCIUM_ION_CONCENTRATION | -1.52 | 0.214 |
| GOBP_LEUKOCYTE_MEDIATED_IMMUNITY | -1.52 | 0.214 |
| GOBP_REGULATION_OF_SYNCYTIUM_FORMATION_BY_PLASMA_MEMBRANE_FUSION | -1.52 | 0.214 |
| GOMF_G_PROTEIN_COUPLED_RECEPTOR_BINDING | -1.52 | 0.216 |
| GOBP_POSITIVE_REGULATION_OF_REGULATORY_T_CELL_DIFFERENTIATION | -1.52 | 0.216 |
| GOBP_REGULATION_OF_PLATELET_ACTIVATION | -1.52 | 0.217 |
| GOBP_NEGATIVE_REGULATION_OF_INTRINSIC_APOPTOTIC_SIGNALING_PATHWAY_IN_RESPONSE_TO_DNA_DAMAGE | -1.52 | 0.217 |
| GOBP_REGULATION_OF_T_CELL_PROLIFERATION | -1.52 | 0.217 |
| GOBP_POSITIVE_REGULATION_OF_LIPASE_ACTIVITY | -1.52 | 0.218 |
| GOBP_REGULATION_OF_PHAGOCYTOSIS | -1.52 | 0.220 |
| GOCC_U1_SNRNP | -1.51 | 0.221 |
| GOBP_CELL_ACTIVATION_INVOLVED_IN_IMMUNE_RESPONSE | -1.51 | 0.222 |
| GOBP_MYELOID_LEUKOCYTE_DIFFERENTIATION | -1.51 | 0.222 |
| GOBP_CELL_AGGREGATION | -1.51 | 0.223 |
| GOBP_T_CELL_DIFFERENTIATION_INVOLVED_IN_IMMUNE_RESPONSE | -1.51 | 0.223 |
| GOBP_POSITIVE_REGULATION_OF_CELL_CELL_ADHESION | -1.51 | 0.223 |
| GOMF_PROTEIN_LIPID_COMPLEX_BINDING | -1.51 | 0.223 |
| GOMF_ZINC_ION_TRANSMEMBRANE_TRANSPORTER_ACTIVITY | -1.51 | 0.225 |
| GOBP_REGULATION_OF_B_CELL_PROLIFERATION | -1.51 | 0.224 |
| GOBP_INTERMEDIATE_FILAMENT_BASED_PROCESS | -1.51 | 0.224 |
| GOCC_CLATHRIN_COATED_ENDOCYTIC_VESICLE_MEMBRANE | -1.51 | 0.224 |
| GOBP_INNATE_IMMUNE_RESPONSE_ACTIVATING_CELL_SURFACE_RECEPTOR_SIGNALING_PATHWAY | -1.51 | 0.225 |
| GOBP_NEUTROPHIL_MIGRATION | -1.51 | 0.225 |
| GOBP_T_CELL_ACTIVATION_INVOLVED_IN_IMMUNE_RESPONSE | -1.51 | 0.225 |
| GOBP_POSITIVE_REGULATION_OF_PROTEIN_DEPOLYMERIZATION | -1.51 | 0.226 |
| GOBP_POSITIVE_REGULATION_OF_LYMPHOCYTE_MIGRATION | -1.51 | 0.226 |
| GOCC_EXTERNAL_ENCAPSULATING_STRUCTURE | -1.51 | 0.228 |
| GOCC_CONTRACTILE_MUSCLE_FIBER | -1.51 | 0.228 |
| GOBP_REGULATION_OF_HOMOTYPIC_CELL_CELL_ADHESION | -1.51 | 0.227 |
| GOBP_POSITIVE_REGULATION_OF_ENDOTHELIAL_CELL_APOPTOTIC_PROCESS | -1.51 | 0.227 |
| GOMF_OXIDOREDUCTASE_ACTIVITY_ACTING_ON_PEROXIDE_AS_ACCEPTOR | -1.50 | 0.227 |
| GOBP_NEGATIVE_REGULATION_OF_GLIOGENESIS | -1.50 | 0.228 |
| GOMF_HEPARIN_BINDING | -1.50 | 0.227 |
| GOBP_POSITIVE_REGULATION_OF_TELOMERASE_RNA_LOCALIZATION_TO_CAJAL_BODY | -1.50 | 0.227 |
| GOBP_REGULATION_OF_EPITHELIAL_CELL_DIFFERENTIATION | -1.50 | 0.229 |
| GOBP_GLYCEROLIPID_CATABOLIC_PROCESS | -1.50 | 0.228 |
| GOBP_CHAPERONE_MEDIATED_PROTEIN_FOLDING | -1.50 | 0.228 |
| GOBP_REGULATION_OF_MIRNA_TRANSCRIPTION | -1.50 | 0.228 |
| GOBP_FORMATION_OF_PRIMARY_GERM_LAYER | -1.50 | 0.228 |
| GOBP_REGULATION_OF_LIPASE_ACTIVITY | -1.50 | 0.228 |
| GOBP_MAINTENANCE_OF_LOCATION_IN_CELL | -1.50 | 0.228 |
| GOBP_BIOLOGICAL_PROCESS_INVOLVED_IN_INTRASPECIES_INTERACTION_BETWEEN_ORGANISMS | -1.50 | 0.228 |
| GOBP_STEM_CELL_DIVISION | -1.50 | 0.228 |
| GOBP_PLASMA_LIPOPROTEIN_PARTICLE_CLEARANCE | -1.50 | 0.227 |
| GOBP_REGULATION_OF_CD4_POSITIVE_ALPHA_BETA_T_CELL_DIFFERENTIATION | -1.50 | 0.227 |
| GOBP_REGULATION_OF_PEPTIDYL_SERINE_PHOSPHORYLATION | -1.50 | 0.227 |
| GOBP_REGULATION_OF_NEUTROPHIL_MIGRATION | -1.50 | 0.226 |
| GOMF_CCR_CHEMOKINE_RECEPTOR_BINDING | -1.50 | 0.227 |
| GOBP_REGULATION_OF_CELL_FATE_SPECIFICATION | -1.50 | 0.228 |
| GOBP_POSITIVE_REGULATION_OF_ALPHA_BETA_T_CELL_ACTIVATION | -1.50 | 0.229 |
| GOBP_PROTEIN_LOCALIZATION_TO_CHROMATIN | -1.50 | 0.229 |
| GOCC_COSTAMERE | -1.50 | 0.231 |
| GOBP_PURINE_DEOXYRIBONUCLEOTIDE_METABOLIC_PROCESS | -1.50 | 0.231 |
| GOBP_REGULATION_OF_ALPHA_BETA_T_CELL_DIFFERENTIATION | -1.50 | 0.230 |
| GOBP_REGULATION_OF_MACROPHAGE_CHEMOTAXIS | -1.50 | 0.231 |
| GOBP_REGULATION_OF_CD8_POSITIVE_ALPHA_BETA_T_CELL_ACTIVATION | -1.49 | 0.233 |
| GOBP_TOLERANCE_INDUCTION | -1.49 | 0.232 |
| GOCC_ENDOPLASMIC_RETICULUM_LUMEN | -1.49 | 0.233 |
| GOBP_REGULATION_OF_LYMPHOCYTE_MIGRATION | -1.49 | 0.233 |
| GOMF_MONOSACCHARIDE_BINDING | -1.49 | 0.233 |
| GOBP_LIPOPOLYSACCHARIDE_MEDIATED_SIGNALING_PATHWAY | -1.49 | 0.233 |
| GOBP_NEUROTRANSMITTER_UPTAKE | -1.49 | 0.234 |
| GOBP_NEGATIVE_REGULATION_OF_OSTEOBLAST_DIFFERENTIATION | -1.49 | 0.235 |
| GOBP_NEUTRAL_LIPID_CATABOLIC_PROCESS | -1.49 | 0.237 |
| GOBP_MAINTENANCE_OF_PROTEIN_LOCATION_IN_CELL | -1.49 | 0.237 |
| GOBP_POSITIVE_REGULATION_OF_INTERLEUKIN_8_PRODUCTION | -1.49 | 0.238 |
| GOBP_COLLAGEN_METABOLIC_PROCESS | -1.49 | 0.238 |
| GOBP_INTERLEUKIN_6_PRODUCTION | -1.49 | 0.239 |
| GOBP_REGULATION_OF_CELL_FATE_COMMITMENT | -1.48 | 0.241 |
| GOBP_INTERLEUKIN_17_PRODUCTION | -1.48 | 0.241 |
| GOBP_POSITIVE_REGULATION_OF_POTASSIUM_ION_TRANSMEMBRANE_TRANSPORT | -1.48 | 0.243 |
| GOBP_TRIGLYCERIDE_CATABOLIC_PROCESS | -1.48 | 0.242 |
| GOMF_CARBOHYDRATE_BINDING | -1.48 | 0.242 |
| GOBP_FEMALE_SEX_DIFFERENTIATION | -1.48 | 0.245 |
| GOMF_OXIDOREDUCTASE_ACTIVITY_ACTING_ON_THE_CH_NH_GROUP_OF_DONORS | -1.48 | 0.246 |
| GOBP_REGULATION_OF_GLIAL_CELL_MIGRATION | -1.48 | 0.246 |
| GOBP_RESPONSE_TO_RETINOIC_ACID | -1.48 | 0.246 |
| GOMF_ORGANOPHOSPHATE_ESTER_TRANSMEMBRANE_TRANSPORTER_ACTIVITY | -1.48 | 0.246 |
| GOBP_FAT_CELL_DIFFERENTIATION | -1.48 | 0.247 |
| GOCC_VACUOLAR_LUMEN | -1.48 | 0.247 |
| GOBP_NEGATIVE_REGULATION_OF_INFLAMMATORY_RESPONSE | -1.48 | 0.248 |
| GOBP_PROTEIN_LOCALIZATION_TO_CHROMOSOME_CENTROMERIC_REGION | -1.48 | 0.248 |
| GOBP_RENAL_ABSORPTION | -1.48 | 0.248 |
| GOBP_POSITIVE_REGULATION_OF_CATION_CHANNEL_ACTIVITY | -1.48 | 0.248 |
| GOBP_ANGIOGENESIS_INVOLVED_IN_WOUND_HEALING | -1.48 | 0.249 |
| GOBP_POSITIVE_REGULATION_OF_TELOMERASE_ACTIVITY | -1.48 | 0.249 |
| GOCC_INTEGRIN_COMPLEX | -1.48 | 0.249 |
| GOBP_NEGATIVE_REGULATION_OF_BLOOD_CIRCULATION | -1.47 | 0.249 |
| GOBP_POSITIVE_REGULATION_OF_STEM_CELL_DIFFERENTIATION | -1.47 | 0.250 |
| GOBP_PROGRAMMED_CELL_DEATH_INVOLVED_IN_CELL_DEVELOPMENT | -1.47 | 0.250 |
| GOBP_REGULATION_OF_CARDIAC_MUSCLE_CONTRACTION_BY_REGULATION_OF_THE_RELEASE_OF_SEQUESTERED_CALCIUM_ION | -1.47 | 0.250 |
| GOBP_RESPONSE_TO_ISCHEMIA | -1.47 | 0.250 |

**NES: Normalized Enrichment Score, the enrichment score for the gene set after it has been normalized across analyzed gene sets. FDR q-value: False discovery rate, the estimated probability that the normalized enrichment score represents a false positive finding. Only gene sets with an FDR q-value ≤ 25% were included.**
