# Supplementary material for: Placental transcriptome profiling in congenital Chagas disease: gene networks associated with transmission
Source: Front Cell Infect Microbiol. 2026 Mar 18;16:1749307. doi: 10.3389/fcimb.2026.1749307 (PMC13038943; doi:10.3389/fcimb.2026.1749307)
Supplement: Supplementary file 8 [file Table4.docx]

**Supplementary Table 4. GSEA results using GO library for M+B+ vs M+B- clinical groups.**

| Enriched gene sets in M+B+ | NES | FDR q-val |
| --- | --- | --- |
| GOMF_STRUCTURAL_CONSTITUENT_OF_CHROMATIN | 2.28 | 0.000 |
| GOBP_ANTIGEN_PROCESSING_AND_PRESENTATION_OF_ENDOGENOUS_ANTIGEN | 2.14 | 0.007 |
| GOCC_MHC_PROTEIN_COMPLEX | 2.09 | 0.010 |
| GOBP_ANTIGEN_PROCESSING_AND_PRESENTATION_OF_ENDOGENOUS_PEPTIDE_ANTIGEN | 2.08 | 0.009 |
| GOCC_LUMENAL_SIDE_OF_ENDOPLASMIC_RETICULUM_MEMBRANE | 2.06 | 0.012 |
| GOCC_NUCLEOSOME | 2.01 | 0.025 |
| GOCC_CYTOSOLIC_LARGE_RIBOSOMAL_SUBUNIT | 1.96 | 0.043 |
| GOCC_LUMENAL_SIDE_OF_MEMBRANE | 1.93 | 0.060 |
| GOCC_TERTIARY_GRANULE_LUMEN | 1.88 | 0.095 |
| GOMF_CYSTEINE_TYPE_ENDOPEPTIDASE_INHIBITOR_ACTIVITY | 1.84 | 0.144 |
| GOBP_ANTIBACTERIAL_HUMORAL_RESPONSE | 1.81 | 0.187 |
| GOCC_SPECIFIC_GRANULE_LUMEN | 1.81 | 0.177 |
| GOMF_NUCLEOSOMAL_DNA_BINDING | 1.80 | 0.179 |
| GOBP_POSITIVE_REGULATION_OF_T_CELL_MEDIATED_CYTOTOXICITY | 1.78 | 0.211 |
| GOCC_ER_TO_GOLGI_TRANSPORT_VESICLE_MEMBRANE | 1.75 | 0.249 |
| GOMF_PEPTIDE_ANTIGEN_BINDING | 1.75 | 0.236 |
| GOBP_HEMOGLOBIN_METABOLIC_PROCESS | 1.75 | 0.230 |
| GOMF_OXYGEN_BINDING | 1.74 | 0.232 |
| GOBP_REGULATION_OF_T_HELPER_17_TYPE_IMMUNE_RESPONSE | 1.74 | 0.227 |
|  |  |  |

| Enriched gene sets in M+B- | NES | FDR q-val |
| --- | --- | --- |
| GOMF_EXTRACELLULAR_MATRIX_BINDING | -2.21 | 0.001 |
| GOMF_EXTRACELLULAR_MATRIX_STRUCTURAL_CONSTITUENT | -2.21 | 0.001 |
| GOBP_EXTERNAL_ENCAPSULATING_STRUCTURE_ORGANIZATION | -2.16 | 0.004 |
| GOBP_SALIVARY_GLAND_DEVELOPMENT | -2.15 | 0.004 |
| GOBP_EXOCRINE_SYSTEM_DEVELOPMENT | -2.13 | 0.004 |
| GOBP_NEGATIVE_CHEMOTAXIS | -2.13 | 0.004 |
| GOBP_COLLAGEN_FIBRIL_ORGANIZATION | -2.12 | 0.004 |
| GOBP_REGULATION_OF_EXTRACELLULAR_MATRIX_ORGANIZATION | -2.09 | 0.006 |
| GOBP_BASEMENT_MEMBRANE_ORGANIZATION | -2.08 | 0.006 |
| GOBP_EXTRACELLULAR_MATRIX_ASSEMBLY | -2.07 | 0.007 |
| GOCC_COLLAGEN_CONTAINING_EXTRACELLULAR_MATRIX | -2.07 | 0.006 |
| GOBP_BRANCHING_INVOLVED_IN_SALIVARY_GLAND_MORPHOGENESIS | -2.05 | 0.009 |
| GOBP_REGULATION_OF_ANIMAL_ORGAN_MORPHOGENESIS | -2.04 | 0.011 |
| GOCC_COLLAGEN_TRIMER | -2.03 | 0.012 |
| GOBP_PROXIMAL_DISTAL_PATTERN_FORMATION | -1.99 | 0.018 |
| GOBP_CARDIAC_MUSCLE_CELL_CONTRACTION | -1.98 | 0.020 |
| GOMF_CHEMOREPELLENT_ACTIVITY | -1.98 | 0.019 |
| GOMF_COLLAGEN_BINDING | -1.98 | 0.019 |
| GOCC_EXTERNAL_ENCAPSULATING_STRUCTURE | -1.97 | 0.020 |
| GOBP_PHAGOLYSOSOME_ASSEMBLY | -1.97 | 0.020 |
| GOBP_SPECIFICATION_OF_ANIMAL_ORGAN_IDENTITY | -1.97 | 0.019 |
| GOBP_APOPTOTIC_PROCESS_INVOLVED_IN_DEVELOPMENT | -1.97 | 0.020 |
| GOBP_ROOF_OF_MOUTH_DEVELOPMENT | -1.96 | 0.021 |
| GOBP_POSITIVE_REGULATION_OF_STEM_CELL_PROLIFERATION | -1.96 | 0.020 |
| GOBP_AORTIC_VALVE_MORPHOGENESIS | -1.95 | 0.021 |
| GOBP_PHAGOSOME_MATURATION | -1.95 | 0.021 |
| GOBP_SEMAPHORIN_PLEXIN_SIGNALING_PATHWAY | -1.95 | 0.020 |
| GOBP_PERICARDIUM_DEVELOPMENT | -1.95 | 0.020 |
| GOBP_NEGATIVE_REGULATION_OF_NEURON_PROJECTION_DEVELOPMENT | -1.95 | 0.020 |
| GOBP_ACTIN_MEDIATED_CELL_CONTRACTION | -1.94 | 0.023 |
| GOBP_POSITIVE_REGULATION_OF_ANIMAL_ORGAN_MORPHOGENESIS | -1.93 | 0.024 |
| GOBP_REGULATION_OF_ODONTOGENESIS | -1.93 | 0.025 |
| GOBP_MESENCHYMAL_CELL_PROLIFERATION | -1.93 | 0.025 |
| GOBP_DIGESTIVE_SYSTEM_DEVELOPMENT | -1.92 | 0.026 |
| GOBP_EMBRYONIC_DIGESTIVE_TRACT_DEVELOPMENT | -1.91 | 0.028 |
| GOBP_GENITALIA_DEVELOPMENT | -1.90 | 0.033 |
| GOBP_CELL_JUNCTION_DISASSEMBLY | -1.90 | 0.033 |
| GOBP_NADP_METABOLIC_PROCESS | -1.90 | 0.032 |
| GOMF_SULFUR_COMPOUND_BINDING | -1.90 | 0.032 |
| GOBP_CELLULAR_RESPONSE_TO_VASCULAR_ENDOTHELIAL_GROWTH_FACTOR_STIMULUS | -1.90 | 0.031 |
| GOBP_CHONDROCYTE_DIFFERENTIATION | -1.90 | 0.031 |
| GOBP_FEMALE_GENITALIA_DEVELOPMENT | -1.90 | 0.031 |
| GOCC_GABA_ERGIC_SYNAPSE | -1.89 | 0.030 |
| GOBP_CARDIAC_MUSCLE_CELL_ACTION_POTENTIAL_INVOLVED_IN_CONTRACTION | -1.89 | 0.031 |
| GOBP_CELL_MIGRATION_INVOLVED_IN_HEART_DEVELOPMENT | -1.89 | 0.031 |
| GOBP_NEGATIVE_REGULATION_OF_AXONOGENESIS | -1.89 | 0.032 |
| GOCC_BASEMENT_MEMBRANE | -1.89 | 0.032 |
| GOMF_CHEMOATTRACTANT_ACTIVITY | -1.88 | 0.033 |
| GOBP_MORPHOGENESIS_OF_A_BRANCHING_STRUCTURE | -1.88 | 0.033 |
| GOBP_DEVELOPMENTAL_GROWTH_INVOLVED_IN_MORPHOGENESIS | -1.88 | 0.032 |
| GOBP_GAMMA_AMINOBUTYRIC_ACID_SIGNALING_PATHWAY | -1.88 | 0.032 |
| GOBP_MESENCHYME_DEVELOPMENT | -1.88 | 0.033 |
| GOBP_APPENDAGE_MORPHOGENESIS | -1.88 | 0.033 |
| GOBP_CALCIUM_DEPENDENT_CELL_CELL_ADHESION_VIA_PLASMA_MEMBRANE_CELL_ADHESION_MOLECULES | -1.87 | 0.034 |
| GOBP_SYNAPSE_ASSEMBLY | -1.87 | 0.036 |
| GOMF_GLYCOSAMINOGLYCAN_BINDING | -1.87 | 0.036 |
| GOBP_MORPHOGENESIS_OF_AN_EPITHELIUM | -1.87 | 0.036 |
| GOBP_ADENYLATE_CYCLASE_MODULATING_G_PROTEIN_COUPLED_RECEPTOR_SIGNALING_PATHWAY | -1.86 | 0.036 |
| GOBP_CARDIAC_MUSCLE_CELL_ACTION_POTENTIAL | -1.86 | 0.036 |
| GOMF_HEPARIN_BINDING | -1.86 | 0.037 |
| GOBP_AMYLOID_BETA_CLEARANCE | -1.86 | 0.037 |
| GOBP_APPENDAGE_DEVELOPMENT | -1.85 | 0.039 |
| GOBP_DEVELOPMENTAL_INDUCTION | -1.85 | 0.040 |
| GOBP_TAXIS | -1.85 | 0.041 |
| GOMF_SEMAPHORIN_RECEPTOR_BINDING | -1.85 | 0.041 |
| GOBP_REGULATION_OF_MORPHOGENESIS_OF_AN_EPITHELIUM | -1.84 | 0.041 |
| GOBP_MACROPHAGE_ACTIVATION_INVOLVED_IN_IMMUNE_RESPONSE | -1.84 | 0.042 |
| GOBP_HEART_VALVE_MORPHOGENESIS | -1.84 | 0.042 |
| GOMF_EXTRACELLULAR_MATRIX_STRUCTURAL_CONSTITUENT_CONFERRING_TENSILE_STRENGTH | -1.84 | 0.042 |
| GOBP_TISSUE_MORPHOGENESIS | -1.84 | 0.043 |
| GOCC_ENDOPLASMIC_RETICULUM_LUMEN | -1.83 | 0.045 |
| GOBP_SEMI_LUNAR_VALVE_DEVELOPMENT | -1.83 | 0.047 |
| GOBP_BILE_ACID_BIOSYNTHETIC_PROCESS | -1.83 | 0.046 |
| GOBP_POSITIVE_REGULATION_OF_EXTRACELLULAR_MATRIX_ORGANIZATION | -1.83 | 0.046 |
| GOMF_SODIUM_CHANNEL_REGULATOR_ACTIVITY | -1.83 | 0.046 |
| GOBP_HOMOPHILIC_CELL_ADHESION_VIA_PLASMA_MEMBRANE_ADHESION_MOLECULES | -1.82 | 0.050 |
| GOBP_MONOATOMIC_ANION_HOMEOSTASIS | -1.82 | 0.051 |
| GOBP_GLIAL_CELL_APOPTOTIC_PROCESS | -1.82 | 0.051 |
| GOBP_RESPONSE_TO_HEPATOCYTE_GROWTH_FACTOR | -1.81 | 0.055 |
| GOBP_DEVELOPMENTAL_CELL_GROWTH | -1.81 | 0.055 |
| GOBP_REGULATION_OF_VESICLE_MEDIATED_TRANSPORT | -1.81 | 0.056 |
| GOBP_POSITIVE_REGULATION_OF_CANONICAL_WNT_SIGNALING_PATHWAY | -1.80 | 0.060 |
| GOMF_O_ACYLTRANSFERASE_ACTIVITY | -1.80 | 0.059 |
| GOBP_POSITIVE_REGULATION_OF_SODIUM_ION_TRANSPORT | -1.80 | 0.059 |
| GOBP_NEURON_PROJECTION_GUIDANCE | -1.80 | 0.060 |
| GOBP_METANEPHROS_DEVELOPMENT | -1.80 | 0.060 |
| GOBP_SYMPATHETIC_NERVOUS_SYSTEM_DEVELOPMENT | -1.79 | 0.060 |
| GOBP_APOPTOTIC_CELL_CLEARANCE | -1.79 | 0.060 |
| GOBP_NATURAL_KILLER_CELL_DIFFERENTIATION | -1.79 | 0.062 |
| GOBP_EMBRYONIC_APPENDAGE_MORPHOGENESIS | -1.79 | 0.063 |
| GOBP_CIRCULATORY_SYSTEM_PROCESS | -1.79 | 0.062 |
| GOBP_CARDIAC_MUSCLE_CELL_MEMBRANE_REPOLARIZATION | -1.79 | 0.062 |
| GOBP_PHYSIOLOGICAL_CARDIAC_MUSCLE_HYPERTROPHY | -1.79 | 0.062 |
| GOBP_DIGESTIVE_TRACT_MORPHOGENESIS | -1.78 | 0.062 |
| GOBP_NEGATIVE_REGULATION_OF_AXON_EXTENSION | -1.78 | 0.063 |
| GOBP_CELL_SUBSTRATE_ADHESION | -1.78 | 0.062 |
| GOBP_REGULATION_OF_ANIMAL_ORGAN_FORMATION | -1.78 | 0.063 |
| GOBP_ACTIN_FILAMENT_BASED_MOVEMENT | -1.78 | 0.062 |
| GOBP_NEGATIVE_REGULATION_OF_CELL_PROJECTION_ORGANIZATION | -1.78 | 0.062 |
| GOMF_CARGO_RECEPTOR_ACTIVITY | -1.78 | 0.062 |
| GOBP_RESPONSE_TO_XENOBIOTIC_STIMULUS | -1.78 | 0.063 |
| GOBP_REGULATION_OF_STEM_CELL_PROLIFERATION | -1.78 | 0.062 |
| GOBP_REGULATION_OF_ENDOCYTOSIS | -1.78 | 0.062 |
| GOBP_REGULATION_OF_SODIUM_ION_TRANSMEMBRANE_TRANSPORTER_ACTIVITY | -1.77 | 0.064 |
| GOCC_MEMBRANE_MICRODOMAIN | -1.77 | 0.064 |
| GOBP_XENOBIOTIC_TRANSMEMBRANE_TRANSPORT | -1.77 | 0.063 |
| GOBP_VENTRICULAR_CARDIAC_MUSCLE_CELL_MEMBRANE_REPOLARIZATION | -1.77 | 0.063 |
| GOBP_EPITHELIAL_TUBE_MORPHOGENESIS | -1.77 | 0.063 |
| GOBP_POSITIVE_CHEMOTAXIS | -1.77 | 0.063 |
| GOBP_POSITIVE_REGULATION_OF_CHOLESTEROL_EFFLUX | -1.77 | 0.062 |
| GOBP_ADRENAL_GLAND_DEVELOPMENT | -1.77 | 0.062 |
| GOBP_REGULATION_OF_GRANULOCYTE_CHEMOTAXIS | -1.77 | 0.063 |
| GOBP_GLIAL_CELL_MIGRATION | -1.76 | 0.064 |
| GOBP_CALCIUM_MEDIATED_SIGNALING | -1.76 | 0.064 |
| GOBP_CARTILAGE_DEVELOPMENT | -1.76 | 0.064 |
| GOBP_EPITHELIAL_TUBE_BRANCHING_INVOLVED_IN_LUNG_MORPHOGENESIS | -1.76 | 0.064 |
| GOBP_RENAL_SYSTEM_DEVELOPMENT | -1.76 | 0.063 |
| GOBP_VITAMIN_TRANSMEMBRANE_TRANSPORT | -1.76 | 0.064 |
| GOCC_COMPLEX_OF_COLLAGEN_TRIMERS | -1.76 | 0.063 |
| GOBP_SYNAPTIC_MEMBRANE_ADHESION | -1.76 | 0.064 |
| GOBP_REGULATION_OF_PLATELET_ACTIVATION | -1.76 | 0.065 |
| GOBP_REGULATION_OF_CHOLESTEROL_METABOLIC_PROCESS | -1.76 | 0.064 |
| GOBP_RESPONSE_TO_ESTROGEN | -1.76 | 0.064 |
| GOBP_POSITIVE_REGULATION_OF_CHEMOTAXIS | -1.76 | 0.064 |
| GOBP_STEM_CELL_DEVELOPMENT | -1.76 | 0.064 |
| GOBP_NEURON_PROJECTION_EXTENSION | -1.76 | 0.063 |
| GOBP_POSITIVE_REGULATION_OF_WNT_SIGNALING_PATHWAY | -1.75 | 0.066 |
| GOBP_POSITIVE_REGULATION_OF_ERK1_AND_ERK2_CASCADE | -1.75 | 0.066 |
| GOBP_REGULATION_OF_HEART_RATE_BY_CARDIAC_CONDUCTION | -1.75 | 0.066 |
| GOMF_TRANSPORTER_REGULATOR_ACTIVITY | -1.75 | 0.067 |
| GOBP_LYMPH_NODE_DEVELOPMENT | -1.75 | 0.067 |
| GOBP_NEURON_RECOGNITION | -1.75 | 0.068 |
| GOBP_CD4_POSITIVE_ALPHA_BETA_T_CELL_PROLIFERATION | -1.74 | 0.069 |
| GOBP_RESPONSE_TO_PH | -1.74 | 0.069 |
| GOBP_BRANCHING_MORPHOGENESIS_OF_AN_EPITHELIAL_TUBE | -1.74 | 0.068 |
| GOMF_INTEGRIN_BINDING | -1.74 | 0.068 |
| GOBP_ORGAN_INDUCTION | -1.74 | 0.068 |
| GOBP_REGULATION_OF_SODIUM_ION_TRANSMEMBRANE_TRANSPORT | -1.74 | 0.068 |
| GOBP_CORONARY_VASCULATURE_DEVELOPMENT | -1.74 | 0.069 |
| GOBP_NEURON_MIGRATION | -1.74 | 0.071 |
| GOBP_PROTEIN_KINASE_A_SIGNALING | -1.74 | 0.071 |
| GOMF_CORECEPTOR_ACTIVITY | -1.73 | 0.072 |
| GOBP_MAINTENANCE_OF_SYNAPSE_STRUCTURE | -1.73 | 0.072 |
| GOBP_DEVELOPMENTAL_PIGMENTATION | -1.73 | 0.073 |
| GOCC_CATENIN_COMPLEX | -1.73 | 0.073 |
| GOMF_STEROL_BINDING | -1.73 | 0.073 |
| GOMF_VOLTAGE_GATED_SODIUM_CHANNEL_ACTIVITY | -1.73 | 0.073 |
| GOBP_SKELETAL_SYSTEM_DEVELOPMENT | -1.73 | 0.072 |
| GOMF_SIGNALING_RECEPTOR_REGULATOR_ACTIVITY | -1.73 | 0.073 |
| GOBP_FIBROBLAST_PROLIFERATION | -1.73 | 0.074 |
| GOBP_FOREBRAIN_CELL_MIGRATION | -1.72 | 0.075 |
| GOBP_SECONDARY_METABOLIC_PROCESS | -1.72 | 0.075 |
| GOBP_REGULATION_OF_CANONICAL_WNT_SIGNALING_PATHWAY | -1.72 | 0.075 |
| GOBP_RESPIRATORY_SYSTEM_DEVELOPMENT | -1.72 | 0.075 |
| GOBP_AXIS_ELONGATION | -1.72 | 0.075 |
| GOBP_CONNECTIVE_TISSUE_DEVELOPMENT | -1.72 | 0.076 |
| GOBP_MYELOID_CELL_ACTIVATION_INVOLVED_IN_IMMUNE_RESPONSE | -1.72 | 0.075 |
| GOBP_CHONDROITIN_SULFATE_METABOLIC_PROCESS | -1.72 | 0.075 |
| GOMF_ATPASE_COUPLED_TRANSMEMBRANE_TRANSPORTER_ACTIVITY | -1.72 | 0.076 |
| GOBP_CENTRAL_NERVOUS_SYSTEM_NEURON_AXONOGENESIS | -1.72 | 0.076 |
| GOMF_INTRACELLULARLY_GATED_CALCIUM_CHANNEL_ACTIVITY | -1.72 | 0.076 |
| GOMF_SCAVENGER_RECEPTOR_ACTIVITY | -1.72 | 0.077 |
| GOBP_REGULATION_OF_CHEMOTAXIS | -1.71 | 0.078 |
| GOMF_ABC_TYPE_TRANSPORTER_ACTIVITY | -1.71 | 0.079 |
| GOBP_NEURON_PROJECTION_EXTENSION_INVOLVED_IN_NEURON_PROJECTION_GUIDANCE | -1.71 | 0.080 |
| GOBP_LYMPH_VESSEL_DEVELOPMENT | -1.71 | 0.080 |
| GOBP_MOLTING_CYCLE_PROCESS | -1.71 | 0.079 |
| GOBP_CHONDROITIN_SULFATE_BIOSYNTHETIC_PROCESS | -1.71 | 0.080 |
| GOBP_NEGATIVE_REGULATION_OF_SIGNAL_TRANSDUCTION_IN_ABSENCE_OF_LIGAND | -1.71 | 0.080 |
| GOBP_REGENERATION | -1.71 | 0.081 |
| GOBP_ODONTOGENESIS | -1.71 | 0.081 |
| GOBP_PROTEIN_O_LINKED_MANNOSYLATION | -1.71 | 0.081 |
| GOMF_CALCIUM_ION_BINDING | -1.70 | 0.081 |
| GOMF_CHEMOKINE_BINDING | -1.70 | 0.083 |
| GOBP_ADENYLATE_CYCLASE_ACTIVATING_G_PROTEIN_COUPLED_RECEPTOR_SIGNALING_PATHWAY | -1.70 | 0.082 |
| GOBP_CELL_DIFFERENTIATION_INVOLVED_IN_METANEPHROS_DEVELOPMENT | -1.70 | 0.082 |
| GOBP_FIBROBLAST_GROWTH_FACTOR_RECEPTOR_SIGNALING_PATHWAY | -1.70 | 0.083 |
| GOBP_GRANULOCYTE_CHEMOTAXIS | -1.70 | 0.083 |
| GOBP_CEREBRAL_CORTEX_CELL_MIGRATION | -1.70 | 0.084 |
| GOBP_POSITIVE_REGULATION_OF_MACROPHAGE_MIGRATION | -1.70 | 0.085 |
| GOBP_NEURAL_CREST_CELL_DIFFERENTIATION | -1.69 | 0.086 |
| GOBP_REGULATION_OF_LEUKOCYTE_DEGRANULATION | -1.69 | 0.086 |
| GOBP_HEART_MORPHOGENESIS | -1.69 | 0.086 |
| GOBP_CANONICAL_WNT_SIGNALING_PATHWAY | -1.69 | 0.086 |
| GOCC_PROTEIN_COMPLEX_INVOLVED_IN_CELL_MATRIX_ADHESION | -1.69 | 0.086 |
| GOMF_INSULIN_LIKE_GROWTH_FACTOR_BINDING | -1.69 | 0.085 |
| GOBP_REGULATION_OF_STEROL_TRANSPORT | -1.69 | 0.086 |
| GOBP_POSITIVE_REGULATION_OF_MACROPHAGE_CHEMOTAXIS | -1.69 | 0.086 |
| GOBP_CELL_PROLIFERATION_IN_FOREBRAIN | -1.69 | 0.087 |
| GOCC_MAIN_AXON | -1.69 | 0.087 |
| GOBP_STEM_CELL_PROLIFERATION | -1.69 | 0.086 |
| GOBP_REGULATION_OF_NEURON_PROJECTION_DEVELOPMENT | -1.69 | 0.087 |
| GOBP_CARDIAC_CHAMBER_DEVELOPMENT | -1.69 | 0.088 |
| GOBP_CELL_MORPHOGENESIS_INVOLVED_IN_NEURON_DIFFERENTIATION | -1.69 | 0.087 |
| GOBP_CHOLESTEROL_EFFLUX | -1.69 | 0.087 |
| GOBP_KERATINOCYTE_MIGRATION | -1.68 | 0.088 |
| GOBP_MORPHOGENESIS_OF_AN_ENDOTHELIUM | -1.68 | 0.088 |
| GOBP_POSITIVE_REGULATION_OF_STEROL_TRANSPORT | -1.68 | 0.088 |
| GOBP_AMEBOIDAL_TYPE_CELL_MIGRATION | -1.68 | 0.087 |
| GOBP_REGULATION_OF_PROTEIN_KINASE_A_SIGNALING | -1.68 | 0.087 |
| GOBP_RESPONSE_TO_AMYLOID_BETA | -1.68 | 0.087 |
| GOBP_PHAGOCYTOSIS_ENGULFMENT | -1.68 | 0.087 |
| GOBP_POSITIVE_REGULATION_OF_LIPID_TRANSPORT | -1.68 | 0.087 |
| GOBP_AXON_DEVELOPMENT | -1.68 | 0.087 |
| GOBP_NADPH_REGENERATION | -1.68 | 0.087 |
| GOBP_I_KAPPAB_PHOSPHORYLATION | -1.68 | 0.087 |
| GOBP_POSITIVE_REGULATION_OF_MICROTUBULE_POLYMERIZATION_OR_DEPOLYMERIZATION | -1.68 | 0.087 |
| GOBP_REGULATION_OF_SMOOTHENED_SIGNALING_PATHWAY | -1.68 | 0.088 |
| GOMF_STEROL_TRANSFER_ACTIVITY | -1.68 | 0.088 |
| GOBP_SYNAPSE_ORGANIZATION | -1.68 | 0.088 |
| GOCC_ENDOCYTIC_VESICLE_MEMBRANE | -1.68 | 0.088 |
| GOBP_MESENCHYMAL_CELL_DIFFERENTIATION | -1.68 | 0.087 |
| GOBP_CELL_CELL_ADHESION_VIA_PLASMA_MEMBRANE_ADHESION_MOLECULES | -1.68 | 0.087 |
| GOBP_CHONDROITIN_SULFATE_PROTEOGLYCAN_METABOLIC_PROCESS | -1.68 | 0.088 |
| GOBP_CHONDROITIN_SULFATE_PROTEOGLYCAN_BIOSYNTHETIC_PROCESS | -1.68 | 0.088 |
| GOBP_METANEPHROS_MORPHOGENESIS | -1.68 | 0.088 |
| GOBP_NEUROTRANSMITTER_SECRETION | -1.67 | 0.089 |
| GOBP_CARDIAC_CONDUCTION | -1.67 | 0.089 |
| GOBP_HEART_PROCESS | -1.67 | 0.089 |
| GOBP_AXON_EXTENSION | -1.67 | 0.089 |
| GOBP_LYMPH_VESSEL_MORPHOGENESIS | -1.67 | 0.089 |
| GOBP_REGULATION_OF_KERATINOCYTE_PROLIFERATION | -1.67 | 0.090 |
| GOBP_EMBRYONIC_CRANIAL_SKELETON_MORPHOGENESIS | -1.67 | 0.091 |
| GOBP_REGULATION_OF_VESICLE_FUSION | -1.67 | 0.091 |
| GOBP_LYTIC_VACUOLE_ORGANIZATION | -1.67 | 0.090 |
| GOBP_MYOBLAST_DIFFERENTIATION | -1.67 | 0.090 |
| GOBP_POSITIVE_REGULATION_OF_ENDOCYTOSIS | -1.67 | 0.091 |
| GOBP_VASCULAR_TRANSPORT | -1.67 | 0.091 |
| GOBP_POSITIVE_REGULATION_OF_RECEPTOR_INTERNALIZATION | -1.67 | 0.090 |
| GOBP_VASCULAR_PROCESS_IN_CIRCULATORY_SYSTEM | -1.67 | 0.090 |
| GOBP_STRIATED_MUSCLE_CONTRACTION | -1.67 | 0.090 |
| GOBP_POSITIVE_REGULATION_OF_MESENCHYMAL_CELL_PROLIFERATION | -1.66 | 0.092 |
| GOBP_RETINAL_GANGLION_CELL_AXON_GUIDANCE | -1.66 | 0.093 |
| GOBP_CARDIAC_VENTRICLE_DEVELOPMENT | -1.66 | 0.092 |
| GOBP_SODIUM_ION_TRANSMEMBRANE_TRANSPORT | -1.66 | 0.093 |
| GOBP_CELL_MATRIX_ADHESION | -1.66 | 0.093 |
| GOBP_MALE_GENITALIA_DEVELOPMENT | -1.66 | 0.094 |
| GOMF_NUCLEAR_ANDROGEN_RECEPTOR_BINDING | -1.66 | 0.094 |
| GOBP_LUNG_MORPHOGENESIS | -1.66 | 0.095 |
| GOBP_CARDIOCYTE_DIFFERENTIATION | -1.66 | 0.094 |
| GOBP_REGULATED_EXOCYTOSIS | -1.66 | 0.094 |
| GOBP_CARDIAC_MUSCLE_CELL_DIFFERENTIATION | -1.66 | 0.095 |
| GOBP_REGULATION_OF_EXTRINSIC_APOPTOTIC_SIGNALING_PATHWAY_IN_ABSENCE_OF_LIGAND | -1.66 | 0.095 |
| GOBP_PATTERN_SPECIFICATION_PROCESS | -1.66 | 0.095 |
| GOBP_RESPONSE_TO_FIBROBLAST_GROWTH_FACTOR | -1.65 | 0.095 |
| GOBP_CELL_JUNCTION_ASSEMBLY | -1.65 | 0.095 |
| GOBP_CELLULAR_RESPONSE_TO_CAMP | -1.65 | 0.095 |
| GOBP_FAT_SOLUBLE_VITAMIN_METABOLIC_PROCESS | -1.65 | 0.095 |
| GOBP_REGULATION_OF_REGULATED_SECRETORY_PATHWAY | -1.65 | 0.095 |
| GOBP_AMINO_ACID_BETAINE_METABOLIC_PROCESS | -1.65 | 0.095 |
| GOBP_GRANULOCYTE_MIGRATION | -1.65 | 0.095 |
| GOBP_POSITIVE_REGULATION_OF_CALCIUM_MEDIATED_SIGNALING | -1.65 | 0.095 |
| GOMF_3_5_CYCLIC_AMP_PHOSPHODIESTERASE_ACTIVITY | -1.65 | 0.096 |
| GOBP_CELL_CELL_SIGNALING_INVOLVED_IN_CARDIAC_CONDUCTION | -1.65 | 0.097 |
| GOBP_POSITIVE_REGULATION_OF_FIBROBLAST_PROLIFERATION | -1.65 | 0.097 |
| GOBP_REGULATION_OF_CARDIAC_MUSCLE_CELL_DIFFERENTIATION | -1.65 | 0.097 |
| GOBP_GLAND_DEVELOPMENT | -1.65 | 0.097 |
| GOBP_RESPONSE_TO_TRANSFORMING_GROWTH_FACTOR_BETA | -1.65 | 0.098 |
| GOBP_TISSUE_HOMEOSTASIS | -1.65 | 0.098 |
| GOMF_OXYSTEROL_BINDING | -1.65 | 0.098 |
| GOBP_RESPONSE_TO_KETONE | -1.65 | 0.098 |
| GOBP_EXCITATORY_SYNAPSE_ASSEMBLY | -1.64 | 0.098 |
| GOBP_SODIUM_ION_HOMEOSTASIS | -1.64 | 0.098 |
| GOBP_REGULATION_OF_MESENCHYMAL_CELL_PROLIFERATION | -1.64 | 0.098 |
| GOBP_ODONTOGENESIS_OF_DENTIN_CONTAINING_TOOTH | -1.64 | 0.098 |
| GOCC_PROTEIN_COMPLEX_INVOLVED_IN_CELL_ADHESION | -1.64 | 0.098 |
| GOBP_CHEMOKINE_PRODUCTION | -1.64 | 0.099 |
| GOBP_NEGATIVE_REGULATION_OF_CELL_SUBSTRATE_ADHESION | -1.64 | 0.098 |
| GOBP_METANEPHRIC_NEPHRON_DEVELOPMENT | -1.64 | 0.100 |
| GOBP_NEURON_FATE_COMMITMENT | -1.64 | 0.099 |
| GOBP_MODIFIED_AMINO_ACID_TRANSPORT | -1.64 | 0.099 |
| GOBP_MUCOPOLYSACCHARIDE_METABOLIC_PROCESS | -1.64 | 0.100 |
| GOBP_REGULATION_OF_CELL_SIZE | -1.64 | 0.100 |
| GOMF_TAU_PROTEIN_BINDING | -1.64 | 0.102 |
| GOBP_CARDIAC_MUSCLE_CONTRACTION | -1.64 | 0.103 |
| GOBP_CARDIAC_SEPTUM_DEVELOPMENT | -1.63 | 0.104 |
| GOBP_VENTRICULAR_CARDIAC_MUSCLE_CELL_ACTION_POTENTIAL | -1.63 | 0.104 |
| GOMF_UDP_GLYCOSYLTRANSFERASE_ACTIVITY | -1.63 | 0.103 |
| GOBP_CELL_CHEMOTAXIS | -1.63 | 0.103 |
| GOBP_REGULATION_OF_FATTY_ACID_TRANSPORT | -1.63 | 0.104 |
| GOBP_REGULATION_OF_LIPID_TRANSPORT | -1.63 | 0.104 |
| GOBP_SYNAPTIC_VESICLE_EXOCYTOSIS | -1.63 | 0.104 |
| GOBP_MEMBRANE_INVAGINATION | -1.63 | 0.104 |
| GOBP_INFLAMMASOME_MEDIATED_SIGNALING_PATHWAY | -1.63 | 0.104 |
| GOBP_LAMELLIPODIUM_MORPHOGENESIS | -1.63 | 0.105 |
| GOBP_VERY_LONG_CHAIN_FATTY_ACID_METABOLIC_PROCESS | -1.63 | 0.105 |
| GOBP_STEROL_TRANSPORT | -1.63 | 0.105 |
| GOBP_OUTFLOW_TRACT_SEPTUM_MORPHOGENESIS | -1.63 | 0.105 |
| GOBP_MANNOSYLATION | -1.63 | 0.107 |
| GOBP_POSITIVE_REGULATION_OF_ORGAN_GROWTH | -1.62 | 0.107 |
| GOBP_TISSUE_MIGRATION | -1.62 | 0.107 |
| GOBP_CELLULAR_RESPONSE_TO_LIPOPROTEIN_PARTICLE_STIMULUS | -1.62 | 0.107 |
| GOCC_SYNAPTIC_MEMBRANE | -1.62 | 0.107 |
| GOBP_MYOBLAST_FUSION | -1.62 | 0.108 |
| GOBP_NEGATIVE_REGULATION_OF_PHAGOCYTOSIS | -1.62 | 0.108 |
| GOBP_B_CELL_RECEPTOR_SIGNALING_PATHWAY | -1.62 | 0.108 |
| GOBP_STRIATED_MUSCLE_TISSUE_DEVELOPMENT | -1.62 | 0.108 |
| GOBP_HEART_VALVE_DEVELOPMENT | -1.62 | 0.110 |
| GOBP_NEGATIVE_REGULATION_OF_EXTRINSIC_APOPTOTIC_SIGNALING_PATHWAY | -1.62 | 0.113 |
| GOBP_POTASSIUM_ION_HOMEOSTASIS | -1.61 | 0.114 |
| GOBP_PRESYNAPSE_ORGANIZATION | -1.61 | 0.115 |
| GOBP_RESPONSE_TO_STEROL | -1.61 | 0.114 |
| GOBP_ESTABLISHMENT_OF_CELL_POLARITY | -1.61 | 0.115 |
| GOBP_NEURON_MATURATION | -1.61 | 0.115 |
| GOBP_NEURON_PROJECTION_REGENERATION | -1.61 | 0.114 |
| GOBP_AXONAL_FASCICULATION | -1.61 | 0.114 |
| GOBP_ANIMAL_ORGAN_FORMATION | -1.61 | 0.114 |
| GOBP_FOREBRAIN_DEVELOPMENT | -1.61 | 0.114 |
| GOBP_REGULATION_OF_VASCULAR_ENDOTHELIAL_GROWTH_FACTOR_RECEPTOR_SIGNALING_PATHWAY | -1.61 | 0.114 |
| GOMF_CYCLIC_NUCLEOTIDE_PHOSPHODIESTERASE_ACTIVITY | -1.61 | 0.115 |
| GOBP_TRANSPOSITION | -1.61 | 0.116 |
| GOBP_EPIBOLY | -1.61 | 0.115 |
| GOCC_PORE_COMPLEX | -1.61 | 0.116 |
| GOBP_STEM_CELL_DIFFERENTIATION | -1.61 | 0.116 |
| GOBP_RENAL_SYSTEM_VASCULATURE_DEVELOPMENT | -1.61 | 0.117 |
| GOBP_MUSCLE_TISSUE_DEVELOPMENT | -1.61 | 0.117 |
| GOBP_RESPONSE_TO_AXON_INJURY | -1.61 | 0.117 |
| GOBP_HINDLIMB_MORPHOGENESIS | -1.61 | 0.116 |
| GOBP_POSITIVE_REGULATION_OF_INFLAMMASOME_MEDIATED_SIGNALING_PATHWAY | -1.60 | 0.117 |
| GOBP_REGULATION_OF_WNT_SIGNALING_PATHWAY | -1.60 | 0.118 |
| GOBP_PLATELET_DERIVED_GROWTH_FACTOR_RECEPTOR_SIGNALING_PATHWAY | -1.60 | 0.118 |
| GOBP_WNT_SIGNALING_PATHWAY | -1.60 | 0.118 |
| GOBP_STEROID_CATABOLIC_PROCESS | -1.60 | 0.117 |
| GOBP_REGULATION_OF_RECEPTOR_INTERNALIZATION | -1.60 | 0.120 |
| GOCC_PLASMA_MEMBRANE_RAFT | -1.60 | 0.120 |
| GOMF_GROWTH_FACTOR_RECEPTOR_BINDING | -1.60 | 0.120 |
| GOMF_SODIUM_CHANNEL_ACTIVITY | -1.60 | 0.120 |
| GOBP_REGULATION_OF_SYSTEM_PROCESS | -1.60 | 0.119 |
| GOBP_UTERUS_DEVELOPMENT | -1.60 | 0.120 |
| GOBP_REGULATION_OF_SUPEROXIDE_METABOLIC_PROCESS | -1.60 | 0.121 |
| GOMF_POTASSIUM_CHANNEL_REGULATOR_ACTIVITY | -1.60 | 0.120 |
| GOBP_DENDRITIC_CELL_CHEMOTAXIS | -1.60 | 0.120 |
| GOBP_REGULATION_OF_PHAGOCYTOSIS | -1.60 | 0.122 |
| GOMF_CELL_ADHESION_MEDIATOR_ACTIVITY | -1.59 | 0.125 |
| GOBP_NEGATIVE_REGULATION_OF_EPITHELIAL_CELL_MIGRATION | -1.59 | 0.125 |
| GOBP_NEGATIVE_REGULATION_OF_LOCOMOTION | -1.59 | 0.126 |
| GOBP_PHOSPHOLIPASE_C_ACTIVATING_G_PROTEIN_COUPLED_RECEPTOR_SIGNALING_PATHWAY | -1.59 | 0.127 |
| GOBP_HETEROPHILIC_CELL_CELL_ADHESION_VIA_PLASMA_MEMBRANE_CELL_ADHESION_MOLECULES | -1.59 | 0.128 |
| GOBP_CELL_FATE_DETERMINATION | -1.59 | 0.128 |
| GOBP_CELL_RECOGNITION | -1.59 | 0.128 |
| GOBP_RAB_PROTEIN_SIGNAL_TRANSDUCTION | -1.59 | 0.129 |
| GOBP_PULMONARY_VALVE_MORPHOGENESIS | -1.59 | 0.129 |
| GOMF_GROWTH_FACTOR_ACTIVITY | -1.59 | 0.129 |
| GOBP_MONOAMINE_TRANSPORT | -1.59 | 0.129 |
| GOBP_POSITIVE_REGULATION_OF_NUCLEAR_DIVISION | -1.58 | 0.129 |
| GOBP_SENSORY_ORGAN_MORPHOGENESIS | -1.58 | 0.129 |
| GOBP_LUNG_EPITHELIUM_DEVELOPMENT | -1.58 | 0.131 |
| GOBP_MAINTENANCE_OF_CELL_POLARITY | -1.58 | 0.131 |
| GOBP_EMBRYONIC_MORPHOGENESIS | -1.58 | 0.131 |
| GOBP_SPECIFICATION_OF_SYMMETRY | -1.58 | 0.132 |
| GOBP_EXOCYTOSIS | -1.58 | 0.132 |
| GOMF_LAMININ_BINDING | -1.58 | 0.132 |
| GOMF_ALDEHYDE_DEHYDROGENASE_NAD_P_PLUS_ACTIVITY | -1.58 | 0.131 |
| GOBP_FOREBRAIN_NEURON_DEVELOPMENT | -1.58 | 0.131 |
| GOBP_EMBRYONIC_SKELETAL_SYSTEM_DEVELOPMENT | -1.58 | 0.131 |
| GOBP_RECEPTOR_METABOLIC_PROCESS | -1.58 | 0.131 |
| GOCC_GLUTAMATERGIC_SYNAPSE | -1.58 | 0.132 |
| GOCC_FILOPODIUM | -1.58 | 0.132 |
| GOBP_CELL_FATE_COMMITMENT | -1.58 | 0.131 |
| GOBP_PHAGOCYTOSIS | -1.58 | 0.131 |
| GOBP_VENTRAL_SPINAL_CORD_DEVELOPMENT | -1.58 | 0.131 |
| GOBP_POSITIVE_REGULATION_OF_LIPID_LOCALIZATION | -1.58 | 0.132 |
| GOBP_ORGANIC_HYDROXY_COMPOUND_BIOSYNTHETIC_PROCESS | -1.58 | 0.132 |
| GOBP_LEUKOCYTE_DEGRANULATION | -1.58 | 0.132 |
| GOBP_REGULATION_OF_RECEPTOR_MEDIATED_ENDOCYTOSIS | -1.58 | 0.132 |
| GOBP_EMBRYONIC_DIGIT_MORPHOGENESIS | -1.58 | 0.132 |
| GOBP_NEGATIVE_REGULATION_OF_PROTEIN_LOCALIZATION_TO_NUCLEUS | -1.58 | 0.132 |
| GOMF_GDP_DISSOCIATION_INHIBITOR_ACTIVITY | -1.57 | 0.132 |
| GOBP_ERK1_AND_ERK2_CASCADE | -1.57 | 0.132 |
| GOBP_NEGATIVE_REGULATION_OF_STEM_CELL_PROLIFERATION | -1.57 | 0.133 |
| GOBP_REGULATION_OF_NEURON_PROJECTION_REGENERATION | -1.57 | 0.133 |
| GOBP_OUTFLOW_TRACT_MORPHOGENESIS | -1.57 | 0.134 |
| GOBP_ATRIAL_CARDIAC_MUSCLE_CELL_TO_AV_NODE_CELL_COMMUNICATION | -1.57 | 0.133 |
| GOBP_CRANIAL_SKELETAL_SYSTEM_DEVELOPMENT | -1.57 | 0.133 |
| GOBP_WNT_SIGNALING_PATHWAY_PLANAR_CELL_POLARITY_PATHWAY | -1.57 | 0.133 |
| GOCC_LAMELLIPODIUM_MEMBRANE | -1.57 | 0.134 |
| GOBP_RECEPTOR_MEDIATED_ENDOCYTOSIS | -1.57 | 0.134 |
| GOBP_NUCLEOSIDE_BISPHOSPHATE_BIOSYNTHETIC_PROCESS | -1.57 | 0.135 |
| GOBP_PHENOL_CONTAINING_COMPOUND_METABOLIC_PROCESS | -1.57 | 0.135 |
| GOBP_SODIUM_ION_TRANSPORT | -1.57 | 0.138 |
| GOCC_PRESYNAPTIC_MEMBRANE | -1.57 | 0.137 |
| GOBP_SULFUR_COMPOUND_BIOSYNTHETIC_PROCESS | -1.57 | 0.137 |
| GOBP_SECONDARY_PALATE_DEVELOPMENT | -1.56 | 0.138 |
| GOBP_CELL_CELL_ADHESION_MEDIATED_BY_CADHERIN | -1.56 | 0.138 |
| GOBP_RECEPTOR_CATABOLIC_PROCESS | -1.56 | 0.138 |
| GOBP_RESPIRATORY_GASEOUS_EXCHANGE_BY_RESPIRATORY_SYSTEM | -1.56 | 0.141 |
| GOBP_PIGMENT_CELL_DIFFERENTIATION | -1.56 | 0.140 |
| GOBP_EYE_MORPHOGENESIS | -1.56 | 0.141 |
| GOBP_CENTRAL_NERVOUS_SYSTEM_PROJECTION_NEURON_AXONOGENESIS | -1.56 | 0.141 |
| GOBP_NEGATIVE_REGULATION_OF_G_PROTEIN_COUPLED_RECEPTOR_SIGNALING_PATHWAY | -1.56 | 0.141 |
| GOBP_VASCULAR_ASSOCIATED_SMOOTH_MUSCLE_CELL_PROLIFERATION | -1.56 | 0.141 |
| GOBP_REGULATION_OF_CARDIAC_MUSCLE_CELL_MEMBRANE_REPOLARIZATION | -1.56 | 0.141 |
| GOBP_RESPONSE_TO_HYDROPEROXIDE | -1.56 | 0.141 |
| GOBP_EMBRYONIC_HINDLIMB_MORPHOGENESIS | -1.56 | 0.141 |
| GOBP_CARDIAC_CHAMBER_MORPHOGENESIS | -1.56 | 0.141 |
| GOBP_POSITIVE_REGULATION_OF_INTERLEUKIN_2_PRODUCTION | -1.56 | 0.142 |
| GOBP_CELLULAR_RESPONSE_TO_XENOBIOTIC_STIMULUS | -1.56 | 0.142 |
| GOMF_VITAMIN_TRANSMEMBRANE_TRANSPORTER_ACTIVITY | -1.56 | 0.144 |
| GOBP_REGULATION_OF_CHOLESTEROL_BIOSYNTHETIC_PROCESS | -1.55 | 0.144 |
| GOBP_ORGANIC_HYDROXY_COMPOUND_TRANSPORT | -1.55 | 0.144 |
| GOBP_NEGATIVE_REGULATION_OF_SMOOTH_MUSCLE_CELL_MIGRATION | -1.55 | 0.144 |
| GOBP_REGULATION_OF_HEART_CONTRACTION | -1.55 | 0.143 |
| GOBP_LYMPHANGIOGENESIS | -1.55 | 0.144 |
| GOMF_ATPASE_COUPLED_MONOATOMIC_CATION_TRANSMEMBRANE_TRANSPORTER_ACTIVITY | -1.55 | 0.144 |
| GOBP_POSITIVE_REGULATION_OF_VASCULAR_ASSOCIATED_SMOOTH_MUSCLE_CELL_PROLIFERATION | -1.55 | 0.144 |
| GOBP_REGULATION_OF_EXTENT_OF_CELL_GROWTH | -1.55 | 0.144 |
| GOBP_RESPONSE_TO_INTERLEUKIN_7 | -1.55 | 0.144 |
| GOBP_BRANCH_ELONGATION_OF_AN_EPITHELIUM | -1.55 | 0.144 |
| GOMF_TRANSMEMBRANE_TRANSPORTER_BINDING | -1.55 | 0.144 |
| GOBP_REGULATION_OF_EXOCYTOSIS | -1.55 | 0.144 |
| GOBP_NEGATIVE_REGULATION_OF_SYSTEMIC_ARTERIAL_BLOOD_PRESSURE | -1.55 | 0.144 |
| GOBP_PROTEOGLYCAN_METABOLIC_PROCESS | -1.55 | 0.144 |
| GOBP_MYELOID_LEUKOCYTE_MIGRATION | -1.55 | 0.145 |
| GOBP_REGULATION_OF_VASCULAR_PERMEABILITY | -1.55 | 0.145 |
| GOBP_CAMP_MEDIATED_SIGNALING | -1.55 | 0.145 |
| GOBP_ESTABLISHMENT_OR_MAINTENANCE_OF_CELL_POLARITY | -1.55 | 0.145 |
| GOBP_ORGANIC_HYDROXY_COMPOUND_METABOLIC_PROCESS | -1.55 | 0.146 |
| GOBP_RESPIRATORY_BURST | -1.55 | 0.147 |
| GOMF_OPSONIN_BINDING | -1.55 | 0.146 |
| GOBP_VASCULAR_ENDOTHELIAL_GROWTH_FACTOR_SIGNALING_PATHWAY | -1.55 | 0.147 |
| GOBP_REGULATION_OF_BLOOD_CIRCULATION | -1.55 | 0.147 |
| GOBP_POSITIVE_REGULATION_OF_RECEPTOR_MEDIATED_ENDOCYTOSIS | -1.55 | 0.147 |
| GOBP_EMBRYONIC_FORELIMB_MORPHOGENESIS | -1.55 | 0.147 |
| GOCC_EXOCYST | -1.54 | 0.148 |
| GOBP_NEGATIVE_REGULATION_OF_SUPRAMOLECULAR_FIBER_ORGANIZATION | -1.54 | 0.148 |
| GOBP_VENTRICULAR_SEPTUM_DEVELOPMENT | -1.54 | 0.148 |
| GOBP_INTERLEUKIN_2_PRODUCTION | -1.54 | 0.147 |
| GOBP_FATTY_ACYL_COA_BIOSYNTHETIC_PROCESS | -1.54 | 0.147 |
| GOMF_LIPOPROTEIN_PARTICLE_RECEPTOR_ACTIVITY | -1.54 | 0.150 |
| GOBP_REGULATION_OF_ALCOHOL_BIOSYNTHETIC_PROCESS | -1.54 | 0.153 |
| GOBP_MEMBRANE_REPOLARIZATION_DURING_ACTION_POTENTIAL | -1.54 | 0.152 |
| GOBP_RESPONSE_TO_ELECTRICAL_STIMULUS | -1.54 | 0.153 |
| GOMF_INTRAMOLECULAR_OXIDOREDUCTASE_ACTIVITY | -1.54 | 0.153 |
| GOBP_REGULATION_OF_SUPEROXIDE_ANION_GENERATION | -1.54 | 0.153 |
| GOMF_BETA_CATENIN_BINDING | -1.54 | 0.153 |
| GOBP_CELL_VOLUME_HOMEOSTASIS | -1.54 | 0.153 |
| GOBP_HEART_GROWTH | -1.54 | 0.153 |
| GOBP_POSITIVE_REGULATION_OF_HEART_GROWTH | -1.54 | 0.153 |
| GOBP_OSSIFICATION | -1.53 | 0.154 |
| GOBP_MORPHOGENESIS_OF_AN_EPITHELIAL_SHEET | -1.53 | 0.156 |
| GOMF_LIGAND_GATED_CALCIUM_CHANNEL_ACTIVITY | -1.53 | 0.155 |
| GOBP_DENDRITE_DEVELOPMENT | -1.53 | 0.155 |
| GOBP_MESENCHYMAL_CELL_MIGRATION | -1.53 | 0.155 |
| GOBP_CELLULAR_RESPONSE_TO_CHOLESTEROL | -1.53 | 0.155 |
| GOBP_MAMMARY_GLAND_EPITHELIUM_DEVELOPMENT | -1.53 | 0.155 |
| GOBP_REGULATION_OF_SODIUM_ION_TRANSPORT | -1.53 | 0.156 |
| GOBP_TRICARBOXYLIC_ACID_CYCLE | -1.53 | 0.156 |
| GOBP_MAMMARY_GLAND_DEVELOPMENT | -1.53 | 0.157 |
| GOCC_CLATHRIN_COATED_ENDOCYTIC_VESICLE_MEMBRANE | -1.53 | 0.157 |
| GOBP_CENTRAL_NERVOUS_SYSTEM_NEURON_DIFFERENTIATION | -1.53 | 0.157 |
| GOCC_POSTSYNAPTIC_MEMBRANE | -1.53 | 0.157 |
| GOBP_CELL_GROWTH | -1.53 | 0.157 |
| GOBP_LEUKOCYTE_CHEMOTAXIS | -1.53 | 0.156 |
| GOBP_CELLULAR_RESPONSE_TO_STEROL | -1.53 | 0.157 |
| GOBP_NEPHRON_DEVELOPMENT | -1.53 | 0.157 |
| GOBP_HYPOTHALAMUS_DEVELOPMENT | -1.53 | 0.157 |
| GOBP_ADENYLATE_CYCLASE_INHIBITING_G_PROTEIN_COUPLED_RECEPTOR_SIGNALING_PATHWAY | -1.53 | 0.157 |
| GOBP_REGULATION_OF_VENTRICULAR_CARDIAC_MUSCLE_CELL_MEMBRANE_REPOLARIZATION | -1.53 | 0.158 |
| GOBP_CARDIAC_CELL_DEVELOPMENT | -1.53 | 0.158 |
| GOBP_REGULATION_OF_CELLULAR_COMPONENT_SIZE | -1.52 | 0.160 |
| GOBP_REGULATION_OF_LAMELLIPODIUM_ORGANIZATION | -1.52 | 0.160 |
| GOBP_REGULATION_OF_AXON_EXTENSION | -1.52 | 0.160 |
| GOMF_NOTCH_BINDING | -1.52 | 0.160 |
| GOBP_CENTRAL_NERVOUS_SYSTEM_NEURON_DEVELOPMENT | -1.52 | 0.160 |
| GOBP_ENDOTHELIAL_CELL_CHEMOTAXIS | -1.52 | 0.161 |
| GOBP_SENSORY_ORGAN_DEVELOPMENT | -1.52 | 0.162 |
| GOBP_THIOESTER_BIOSYNTHETIC_PROCESS | -1.52 | 0.162 |
| GOBP_REGULATION_OF_CELLULAR_RESPONSE_TO_VASCULAR_ENDOTHELIAL_GROWTH_FACTOR_STIMULUS | -1.52 | 0.162 |
| GOBP_INTRACELLULAR_PH_REDUCTION | -1.52 | 0.162 |
| GOBP_RESPONSE_TO_GONADOTROPIN | -1.52 | 0.163 |
| GOBP_NEUROBLAST_PROLIFERATION | -1.52 | 0.162 |
| GOBP_ENDOTHELIAL_CELL_MIGRATION | -1.52 | 0.162 |
| GOBP_ORGAN_GROWTH | -1.52 | 0.162 |
| GOBP_NEGATIVE_REGULATION_OF_PROTEIN_POLYMERIZATION | -1.52 | 0.162 |
| GOBP_REGULATION_OF_PLATELET_AGGREGATION | -1.52 | 0.162 |
| GOMF_CARGO_ADAPTOR_ACTIVITY | -1.52 | 0.162 |
| GOMF_ISOPRENOID_BINDING | -1.52 | 0.162 |
| GOBP_STEROID_METABOLIC_PROCESS | -1.52 | 0.162 |
| GOBP_REGULATION_OF_CALCIUM_MEDIATED_SIGNALING | -1.52 | 0.162 |
| GOBP_LEUKOCYTE_ACTIVATION_INVOLVED_IN_INFLAMMATORY_RESPONSE | -1.52 | 0.163 |
| GOBP_KERATINOCYTE_PROLIFERATION | -1.52 | 0.163 |
| GOBP_TELENCEPHALON_GLIAL_CELL_MIGRATION | -1.52 | 0.163 |
| GOBP_EMBRYONIC_HEART_TUBE_DEVELOPMENT | -1.52 | 0.163 |
| GOBP_MUSCLE_CONTRACTION | -1.52 | 0.163 |
| GOBP_CELL_DIFFERENTIATION_INVOLVED_IN_KIDNEY_DEVELOPMENT | -1.52 | 0.163 |
| GOBP_ENDOCARDIAL_CUSHION_DEVELOPMENT | -1.51 | 0.164 |
| GOBP_SPHINGOLIPID_BIOSYNTHETIC_PROCESS | -1.51 | 0.165 |
| GOBP_NEUTROPHIL_HOMEOSTASIS | -1.51 | 0.165 |
| GOBP_RENAL_TUBULE_DEVELOPMENT | -1.51 | 0.166 |
| GOBP_POSITIVE_REGULATION_OF_NEURON_PROJECTION_DEVELOPMENT | -1.51 | 0.166 |
| GOBP_REGULATION_OF_NEURON_MIGRATION | -1.51 | 0.166 |
| GOBP_NEUROINFLAMMATORY_RESPONSE | -1.51 | 0.166 |
| GOBP_MACROPHAGE_MIGRATION | -1.51 | 0.166 |
| GOBP_POSITIVE_REGULATION_OF_STEROID_METABOLIC_PROCESS | -1.51 | 0.165 |
| GOBP_VESICLE_MEDIATED_TRANSPORT_IN_SYNAPSE | -1.51 | 0.166 |
| GOCC_SCHAFFER_COLLATERAL_CA1_SYNAPSE | -1.51 | 0.165 |
| GOBP_SYNAPTIC_TRANSMISSION_GABAERGIC | -1.51 | 0.166 |
| GOBP_REGULATION_OF_HOMOTYPIC_CELL_CELL_ADHESION | -1.51 | 0.167 |
| GOBP_REGULATION_OF_STRIATED_MUSCLE_CONTRACTION | -1.51 | 0.166 |
| GOBP_EAR_DEVELOPMENT | -1.51 | 0.168 |
| GOBP_INOSITOL_PHOSPHATE_METABOLIC_PROCESS | -1.51 | 0.168 |
| GOBP_POSITIVE_REGULATION_OF_CHEMOKINE_PRODUCTION | -1.51 | 0.168 |
| GOBP_PULMONARY_VALVE_DEVELOPMENT | -1.51 | 0.168 |
| GOBP_POSITIVE_REGULATION_OF_PHAGOCYTOSIS | -1.51 | 0.168 |
| GOBP_RESPONSE_TO_ALCOHOL | -1.51 | 0.168 |
| GOMF_LIPID_TRANSFER_ACTIVITY | -1.51 | 0.168 |
| GOBP_INNER_EAR_MORPHOGENESIS | -1.51 | 0.169 |
| GOMF_OXIDOREDUCTASE_ACTIVITY_ACTING_ON_THE_CH_NH2_GROUP_OF_DONORS | -1.51 | 0.169 |
| GOBP_BONE_DEVELOPMENT | -1.50 | 0.170 |
| GOBP_LEUKOCYTE_MIGRATION | -1.50 | 0.172 |
| GOBP_NEGATIVE_REGULATION_OF_MUSCLE_CELL_DIFFERENTIATION | -1.50 | 0.172 |
| GOBP_EMBRYONIC_ORGAN_DEVELOPMENT | -1.50 | 0.171 |
| GOBP_POSITIVE_REGULATION_OF_CARDIAC_MUSCLE_CELL_PROLIFERATION | -1.50 | 0.171 |
| GOBP_PROTEIN_LIPID_COMPLEX_ASSEMBLY | -1.50 | 0.171 |
| GOBP_NEUROTRANSMITTER_TRANSPORT | -1.50 | 0.171 |
| GOBP_NEUTROPHIL_ACTIVATION_INVOLVED_IN_IMMUNE_RESPONSE | -1.50 | 0.171 |
| GOCC_CELL_CELL_JUNCTION | -1.50 | 0.171 |
| GOBP_POSITIVE_REGULATION_OF_NEURAL_PRECURSOR_CELL_PROLIFERATION | -1.50 | 0.171 |
| GOMF_FRIZZLED_BINDING | -1.50 | 0.171 |
| GOBP_RESPONSE_TO_ESTRADIOL | -1.50 | 0.171 |
| GOBP_MONONUCLEAR_CELL_MIGRATION | -1.50 | 0.172 |
| GOBP_COMPLEMENT_ACTIVATION_CLASSICAL_PATHWAY | -1.50 | 0.172 |
| GOBP_STEROID_BIOSYNTHETIC_PROCESS | -1.50 | 0.171 |
| GOBP_TRANSPORT_OF_VIRUS | -1.50 | 0.171 |
| GOBP_VASODILATION | -1.50 | 0.172 |
| GOMF_PROTEOGLYCAN_BINDING | -1.50 | 0.172 |
| GOBP_GANGLIOSIDE_METABOLIC_PROCESS | -1.50 | 0.172 |
| GOMF_DEACETYLASE_ACTIVITY | -1.50 | 0.172 |
| GOBP_REGULATION_OF_CELL_SUBSTRATE_ADHESION | -1.50 | 0.172 |
| GOBP_POSITIVE_REGULATION_OF_MITOTIC_NUCLEAR_DIVISION | -1.50 | 0.175 |
| GOBP_FOREBRAIN_GENERATION_OF_NEURONS | -1.50 | 0.174 |
| GOBP_AMINOGLYCAN_METABOLIC_PROCESS | -1.50 | 0.175 |
| GOBP_PITUITARY_GLAND_DEVELOPMENT | -1.50 | 0.175 |
| GOBP_RETINA_LAYER_FORMATION | -1.49 | 0.175 |
| GOBP_HEART_FORMATION | -1.49 | 0.175 |
| GOBP_CELL_COMMUNICATION_INVOLVED_IN_CARDIAC_CONDUCTION | -1.49 | 0.175 |
| GOBP_DOPAMINE_TRANSPORT | -1.49 | 0.175 |
| GOBP_POSITIVE_REGULATION_OF_CELL_DEVELOPMENT | -1.49 | 0.175 |
| GOBP_ENDOTHELIUM_DEVELOPMENT | -1.49 | 0.177 |
| GOBP_POSITIVE_REGULATION_OF_MUSCLE_HYPERTROPHY | -1.49 | 0.177 |
| GOBP_RESPONSE_TO_WOUNDING | -1.49 | 0.178 |
| GOBP_SKELETAL_MUSCLE_CELL_DIFFERENTIATION | -1.49 | 0.179 |
| GOBP_CELL_ADHESION_MEDIATED_BY_INTEGRIN | -1.49 | 0.179 |
| GOBP_ORGANIC_ACID_BIOSYNTHETIC_PROCESS | -1.49 | 0.178 |
| GOBP_REGULATION_OF_MEMBRANE_DEPOLARIZATION | -1.49 | 0.179 |
| GOBP_GLOMERULUS_DEVELOPMENT | -1.49 | 0.179 |
| GOBP_AXONAL_TRANSPORT_OF_MITOCHONDRION | -1.49 | 0.179 |
| GOBP_KIDNEY_EPITHELIUM_DEVELOPMENT | -1.49 | 0.179 |
| GOBP_VASCULAR_ENDOTHELIAL_GROWTH_FACTOR_RECEPTOR_SIGNALING_PATHWAY | -1.49 | 0.179 |
| GOBP_WALKING_BEHAVIOR | -1.49 | 0.179 |
| GOMF_GLYCOLIPID_BINDING | -1.49 | 0.180 |
| GOBP_POST_ANAL_TAIL_MORPHOGENESIS | -1.49 | 0.180 |
| GOBP_POSITIVE_REGULATION_OF_NOTCH_SIGNALING_PATHWAY | -1.49 | 0.180 |
| GOBP_EMBRYONIC_ORGAN_MORPHOGENESIS | -1.49 | 0.180 |
| GOBP_CELLULAR_ALDEHYDE_METABOLIC_PROCESS | -1.49 | 0.179 |
| GOBP_NEGATIVE_REGULATION_OF_EPITHELIAL_CELL_APOPTOTIC_PROCESS | -1.49 | 0.179 |
| GOCC_PIGMENT_GRANULE | -1.49 | 0.180 |
| GOBP_LENS_FIBER_CELL_DIFFERENTIATION | -1.49 | 0.179 |
| GOBP_EPITHELIAL_CELL_MORPHOGENESIS | -1.49 | 0.179 |
| GOBP_REGULATION_OF_STEROID_BIOSYNTHETIC_PROCESS | -1.49 | 0.180 |
| GOBP_REGULATION_OF_MORPHOGENESIS_OF_A_BRANCHING_STRUCTURE | -1.48 | 0.180 |
| GOBP_REGULATION_OF_MYOBLAST_DIFFERENTIATION | -1.48 | 0.180 |
| GOBP_PIGMENTATION | -1.48 | 0.180 |
| GOBP_LUNG_ALVEOLUS_DEVELOPMENT | -1.48 | 0.180 |
| GOBP_SMOOTHENED_SIGNALING_PATHWAY | -1.48 | 0.180 |
| GOBP_NEGATIVE_REGULATION_OF_SIGNALING_RECEPTOR_ACTIVITY | -1.48 | 0.181 |
| GOBP_NEGATIVE_REGULATION_OF_CYTOSKELETON_ORGANIZATION | -1.48 | 0.181 |
| GOBP_REGULATION_OF_ANATOMICAL_STRUCTURE_SIZE | -1.48 | 0.181 |
| GOCC_CHITOSOME | -1.48 | 0.181 |
| GOBP_ACTION_POTENTIAL | -1.48 | 0.181 |
| GOBP_GLAND_MORPHOGENESIS | -1.48 | 0.181 |
| GOBP_NEGATIVE_REGULATION_OF_PLASMA_MEMBRANE_BOUNDED_CELL_PROJECTION_ASSEMBLY | -1.48 | 0.181 |
| GOBP_UROGENITAL_SYSTEM_DEVELOPMENT | -1.48 | 0.180 |
| GOBP_REGULATION_OF_INFLAMMASOME_MEDIATED_SIGNALING_PATHWAY | -1.48 | 0.182 |
| GOBP_MONOCARBOXYLIC_ACID_BIOSYNTHETIC_PROCESS | -1.48 | 0.182 |
| GOBP_SEGMENTATION | -1.48 | 0.182 |
| GOCC_LAMELLIPODIUM | -1.48 | 0.182 |
| GOBP_MACROPHAGE_ACTIVATION | -1.48 | 0.182 |
| GOBP_REGULATION_OF_ACTIN_FILAMENT_BASED_MOVEMENT | -1.48 | 0.181 |
| GOBP_REGULATION_OF_MUSCLE_CELL_DIFFERENTIATION | -1.48 | 0.182 |
| GOBP_REGULATION_OF_G_PROTEIN_COUPLED_RECEPTOR_SIGNALING_PATHWAY | -1.48 | 0.182 |
| GOBP_INTRACELLULAR_SODIUM_ION_HOMEOSTASIS | -1.48 | 0.182 |
| GOMF_CHOLESTEROL_BINDING | -1.48 | 0.182 |
| GOBP_PLATELET_ACTIVATION | -1.48 | 0.182 |
| GOBP_PHOSPHATIDYLCHOLINE_METABOLIC_PROCESS | -1.48 | 0.183 |
| GOBP_SENSORY_SYSTEM_DEVELOPMENT | -1.48 | 0.183 |
| GOBP_POSITIVE_REGULATION_OF_SMOOTHENED_SIGNALING_PATHWAY | -1.48 | 0.183 |
| GOBP_REGULATION_OF_SYNAPTIC_VESICLE_EXOCYTOSIS | -1.47 | 0.185 |
| GOBP_REGULATION_OF_NERVOUS_SYSTEM_DEVELOPMENT | -1.47 | 0.185 |
| GOMF_OXIDOREDUCTASE_ACTIVITY_ACTING_ON_THE_CH_NH2_GROUP_OF_DONORS_OXYGEN_AS_ACCEPTOR | -1.47 | 0.185 |
| GOBP_CELLULAR_RESPONSE_TO_ALCOHOL | -1.47 | 0.185 |
| GOBP_ENDOCARDIAL_CUSHION_MORPHOGENESIS | -1.47 | 0.185 |
| GOBP_UNSATURATED_FATTY_ACID_BIOSYNTHETIC_PROCESS | -1.47 | 0.185 |
| GOBP_MESODERM_DEVELOPMENT | -1.47 | 0.185 |
| GOBP_ARTERY_DEVELOPMENT | -1.47 | 0.185 |
| GOBP_SYNCYTIUM_FORMATION | -1.47 | 0.186 |
| GOBP_RELAXATION_OF_MUSCLE | -1.47 | 0.186 |
| GOBP_RETINA_VASCULATURE_DEVELOPMENT_IN_CAMERA_TYPE_EYE | -1.47 | 0.187 |
| GOBP_POSITIVE_REGULATION_OF_BONE_RESORPTION | -1.47 | 0.189 |
| GOBP_POSITIVE_REGULATION_OF_NEUROBLAST_PROLIFERATION | -1.47 | 0.189 |
| GOBP_NEGATIVE_REGULATION_OF_CELL_GROWTH | -1.47 | 0.189 |
| GOBP_MUSCLE_CELL_DIFFERENTIATION | -1.47 | 0.190 |
| GOBP_STEROL_BIOSYNTHETIC_PROCESS | -1.47 | 0.192 |
| GOBP_REGULATION_OF_CHOLESTEROL_EFFLUX | -1.47 | 0.192 |
| GOBP_EPITHELIAL_CELL_DEVELOPMENT | -1.47 | 0.192 |
| GOBP_ORGANIC_HYDROXY_COMPOUND_CATABOLIC_PROCESS | -1.47 | 0.192 |
| GOBP_APOPTOTIC_PROCESS_INVOLVED_IN_MORPHOGENESIS | -1.46 | 0.193 |
| GOBP_REGULATION_OF_POSITIVE_CHEMOTAXIS | -1.46 | 0.193 |
| GOBP_REGULATION_OF_ACTIN_FILAMENT_BASED_PROCESS | -1.46 | 0.192 |
| GOBP_REGULATION_OF_CHONDROCYTE_DIFFERENTIATION | -1.46 | 0.192 |
| GOBP_REGULATION_OF_SUPRAMOLECULAR_FIBER_ORGANIZATION | -1.46 | 0.192 |
| GOMF_INTRACELLULARLY_LIGAND_GATED_MONOATOMIC_ION_CHANNEL_ACTIVITY | -1.46 | 0.193 |
| GOBP_REGULATION_OF_MYELOID_LEUKOCYTE_MEDIATED_IMMUNITY | -1.46 | 0.193 |
| GOBP_SULFUR_COMPOUND_METABOLIC_PROCESS | -1.46 | 0.194 |
| GOBP_HEMATOPOIETIC_OR_LYMPHOID_ORGAN_DEVELOPMENT | -1.46 | 0.193 |
| GOMF_P_TYPE_TRANSMEMBRANE_TRANSPORTER_ACTIVITY | -1.46 | 0.194 |
| GOMF_CYTOKINE_ACTIVITY | -1.46 | 0.194 |
| GOMF_WNT_PROTEIN_BINDING | -1.46 | 0.194 |
| GOMF_TRANSFORMING_GROWTH_FACTOR_BETA_BINDING | -1.46 | 0.194 |
| GOBP_ARTERY_MORPHOGENESIS | -1.46 | 0.195 |
| GOBP_REGULATION_OF_SYSTEMIC_ARTERIAL_BLOOD_PRESSURE | -1.46 | 0.196 |
| GOMF_AMIDE_BINDING | -1.46 | 0.196 |
| GOBP_MYELOID_LEUKOCYTE_ACTIVATION | -1.46 | 0.196 |
| GOBP_SMOOTH_MUSCLE_CELL_MIGRATION | -1.46 | 0.196 |
| GOMF_STRUCTURAL_CONSTITUENT_OF_SYNAPSE | -1.46 | 0.200 |
| GOBP_TISSUE_REGENERATION | -1.46 | 0.200 |
| GOBP_EMBRYONIC_PLACENTA_MORPHOGENESIS | -1.46 | 0.200 |
| GOBP_REGULATION_OF_ENDOTHELIAL_CELL_CHEMOTAXIS | -1.46 | 0.200 |
| GOBP_POSITIVE_REGULATION_OF_NERVOUS_SYSTEM_DEVELOPMENT | -1.46 | 0.200 |
| GOMF_PHOSPHORIC_ESTER_HYDROLASE_ACTIVITY | -1.45 | 0.200 |
| GOBP_NEGATIVE_REGULATION_OF_ENDOCYTOSIS | -1.45 | 0.199 |
| GOMF_RECEPTOR_SERINE_THREONINE_KINASE_BINDING | -1.45 | 0.200 |
| GOBP_LABYRINTHINE_LAYER_MORPHOGENESIS | -1.45 | 0.200 |
| GOCC_LATERAL_PLASMA_MEMBRANE | -1.45 | 0.200 |
| GOBP_NEGATIVE_REGULATION_OF_DEVELOPMENTAL_GROWTH | -1.45 | 0.200 |
| GOBP_MORPHOGENESIS_OF_A_POLARIZED_EPITHELIUM | -1.45 | 0.199 |
| GOBP_REGULATION_OF_PLATELET_DERIVED_GROWTH_FACTOR_RECEPTOR_SIGNALING_PATHWAY | -1.45 | 0.199 |
| GOBP_MUSCLE_CELL_MIGRATION | -1.45 | 0.199 |
| GOBP_SMALL_MOLECULE_BIOSYNTHETIC_PROCESS | -1.45 | 0.199 |
| GOBP_REGULATION_OF_SMOOTH_MUSCLE_CELL_DIFFERENTIATION | -1.45 | 0.200 |
| GOBP_REGULATION_OF_EPITHELIAL_CELL_MIGRATION | -1.45 | 0.200 |
| GOMF_PHOSPHOLIPID_BINDING | -1.45 | 0.200 |
| GOBP_REGULATION_OF_MAST_CELL_DEGRANULATION | -1.45 | 0.200 |
| GOBP_CATECHOL_CONTAINING_COMPOUND_METABOLIC_PROCESS | -1.45 | 0.201 |
| GOBP_VASCULAR_ASSOCIATED_SMOOTH_MUSCLE_CELL_MIGRATION | -1.45 | 0.201 |
| GOBP_POSITIVE_REGULATION_OF_HEMOPOIESIS | -1.45 | 0.201 |
| GOBP_SYNAPTIC_VESICLE_RECYCLING | -1.45 | 0.201 |
| GOBP_REGULATION_OF_CELL_CELL_ADHESION | -1.45 | 0.201 |
| GOBP_NEGATIVE_REGULATION_OF_EPITHELIAL_CELL_DIFFERENTIATION | -1.45 | 0.202 |
| GOBP_REGULATION_OF_FIBROBLAST_PROLIFERATION | -1.45 | 0.202 |
| GOBP_SULFUR_AMINO_ACID_METABOLIC_PROCESS | -1.45 | 0.202 |
| GOBP_SMOOTH_MUSCLE_CELL_APOPTOTIC_PROCESS | -1.45 | 0.202 |
| GOBP_REGULATION_OF_ADENYLATE_CYCLASE_ACTIVITY | -1.45 | 0.203 |
| GOBP_CELLULAR_RESPONSE_TO_CALCIUM_ION | -1.45 | 0.203 |
| GOBP_COLLAGEN_METABOLIC_PROCESS | -1.45 | 0.203 |
| GOBP_ACID_SECRETION | -1.45 | 0.203 |
| GOBP_AMINOGLYCAN_BIOSYNTHETIC_PROCESS | -1.45 | 0.202 |
| GOBP_MELANOCYTE_DIFFERENTIATION | -1.45 | 0.202 |
| GOBP_NEGATIVE_REGULATION_OF_LIPID_TRANSPORT | -1.45 | 0.202 |
| GOBP_OLFACTORY_LOBE_DEVELOPMENT | -1.45 | 0.202 |
| GOBP_INTRACELLULAR_STEROL_TRANSPORT | -1.45 | 0.202 |
| GOBP_REGULATION_OF_MEMBRANE_POTENTIAL | -1.45 | 0.202 |
| GOBP_REGULATION_OF_HEART_GROWTH | -1.45 | 0.203 |
| GOBP_CARDIAC_SEPTUM_MORPHOGENESIS | -1.44 | 0.204 |
| GOBP_POSITIVE_REGULATION_OF_MAPK_CASCADE | -1.44 | 0.204 |
| GOBP_REGULATION_OF_STEROID_METABOLIC_PROCESS | -1.44 | 0.204 |
| GOBP_CYCLIC_NUCLEOTIDE_METABOLIC_PROCESS | -1.44 | 0.204 |
| GOBP_FOREBRAIN_NEURON_DIFFERENTIATION | -1.44 | 0.205 |
| GOBP_REGULATION_OF_CELLULAR_RESPONSE_TO_GROWTH_FACTOR_STIMULUS | -1.44 | 0.206 |
| GOBP_CELLULAR_RESPONSE_TO_ORGANIC_CYCLIC_COMPOUND | -1.44 | 0.206 |
| GOBP_XENOBIOTIC_METABOLIC_PROCESS | -1.44 | 0.206 |
| GOBP_NEGATIVE_REGULATION_OF_NERVOUS_SYSTEM_DEVELOPMENT | -1.44 | 0.207 |
| GOCC_EXOCYTIC_VESICLE | -1.44 | 0.208 |
| GOBP_RECEPTOR_CLUSTERING | -1.44 | 0.209 |
| GOBP_ESTABLISHMENT_OF_MITOCHONDRION_LOCALIZATION | -1.44 | 0.208 |
| GOBP_MESENCHYME_MORPHOGENESIS | -1.44 | 0.209 |
| GOMF_MODIFIED_AMINO_ACID_BINDING | -1.44 | 0.208 |
| GOMF_MODIFIED_AMINO_ACID_TRANSMEMBRANE_TRANSPORTER_ACTIVITY | -1.44 | 0.210 |
| GOBP_POSITIVE_REGULATION_OF_DNA_DAMAGE_RESPONSE_SIGNAL_TRANSDUCTION_BY_P53_CLASS_MEDIATOR | -1.44 | 0.210 |
| GOBP_MOLTING_CYCLE | -1.44 | 0.211 |
| GOBP_REGULATION_OF_CELL_GROWTH | -1.44 | 0.212 |
| GOBP_CARDIAC_VENTRICLE_MORPHOGENESIS | -1.44 | 0.212 |
| GOCC_ENDOCYTIC_VESICLE | -1.43 | 0.212 |
| GOBP_ADHERENS_JUNCTION_ORGANIZATION | -1.43 | 0.213 |
| GOBP_POSITIVE_REGULATION_OF_SYNAPSE_ASSEMBLY | -1.43 | 0.213 |
| GOCC_EXTERNAL_SIDE_OF_PLASMA_MEMBRANE | -1.43 | 0.213 |
| GOBP_CELLULAR_RESPONSE_TO_KETONE | -1.43 | 0.213 |
| GOMF_EXOGENOUS_PROTEIN_BINDING | -1.43 | 0.214 |
| GOCC_POSTSYNAPTIC_SPECIALIZATION_MEMBRANE | -1.43 | 0.214 |
| GOMF_WNT_RECEPTOR_ACTIVITY | -1.43 | 0.214 |
| GOBP_REGULATION_OF_ORGAN_GROWTH | -1.43 | 0.215 |
| GOBP_ORGANELLE_MEMBRANE_FUSION | -1.43 | 0.215 |
| GOCC_PLASMA_MEMBRANE_PROTEIN_COMPLEX | -1.43 | 0.215 |
| GOBP_POSITIVE_REGULATION_OF_DOUBLE_STRAND_BREAK_REPAIR_VIA_NONHOMOLOGOUS_END_JOINING | -1.43 | 0.215 |
| GOBP_GLYCEROLIPID_CATABOLIC_PROCESS | -1.43 | 0.216 |
| GOCC_LYSOSOMAL_LUMEN | -1.43 | 0.216 |
| GOBP_ESTABLISHMENT_OF_TISSUE_POLARITY | -1.43 | 0.216 |
| GOMF_PHOSPHORIC_DIESTER_HYDROLASE_ACTIVITY | -1.43 | 0.216 |
| GOBP_NEUTROPHIL_CHEMOTAXIS | -1.43 | 0.217 |
| GOBP_AMIDE_BIOSYNTHETIC_PROCESS | -1.43 | 0.218 |
| GOBP_KIDNEY_MORPHOGENESIS | -1.43 | 0.218 |
| GOCC_POSTSYNAPSE | -1.43 | 0.218 |
| GOMF_ADENYL_NUCLEOTIDE_EXCHANGE_FACTOR_ACTIVITY | -1.43 | 0.218 |
| GOBP_POSITIVE_REGULATION_OF_ENDOTHELIAL_CELL_PROLIFERATION | -1.43 | 0.218 |
| GOBP_RESPONSE_TO_ACIDIC_PH | -1.43 | 0.218 |
| GOBP_POSITIVE_REGULATION_OF_SMOOTH_MUSCLE_CELL_PROLIFERATION | -1.42 | 0.221 |
| GOBP_POSITIVE_REGULATION_OF_EPITHELIAL_CELL_PROLIFERATION | -1.42 | 0.222 |
| GOBP_NEURAL_PRECURSOR_CELL_PROLIFERATION | -1.42 | 0.222 |
| GOCC_NEURON_PROJECTION_MEMBRANE | -1.42 | 0.222 |
| GOBP_ENDOCRINE_SYSTEM_DEVELOPMENT | -1.42 | 0.222 |
| GOBP_REGULATION_OF_HEART_RATE | -1.42 | 0.222 |
| GOCC_DENDRITIC_TREE | -1.42 | 0.222 |
| GOBP_POSITIVE_REGULATION_OF_PROTEIN_DEPOLYMERIZATION | -1.42 | 0.222 |
| GOBP_LEUKOCYTE_HOMEOSTASIS | -1.42 | 0.222 |
| GOBP_MESODERM_MORPHOGENESIS | -1.42 | 0.225 |
| GOBP_NEGATIVE_REGULATION_OF_BLOOD_PRESSURE | -1.42 | 0.225 |
| GOBP_TRANSFORMING_GROWTH_FACTOR_BETA_RECEPTOR_SIGNALING_PATHWAY | -1.42 | 0.225 |
| GOBP_REGULATION_OF_CATECHOLAMINE_METABOLIC_PROCESS | -1.42 | 0.227 |
| GOBP_BONE_RESORPTION | -1.42 | 0.226 |
| GOBP_NON_CANONICAL_WNT_SIGNALING_PATHWAY | -1.42 | 0.227 |
| GOMF_CALCIUM_DEPENDENT_PHOSPHOLIPID_BINDING | -1.42 | 0.227 |
| GOBP_EPIDERMIS_MORPHOGENESIS | -1.42 | 0.227 |
| GOBP_POSITIVE_REGULATION_OF_SMAD_PROTEIN_SIGNAL_TRANSDUCTION | -1.42 | 0.226 |
| GOBP_EAR_MORPHOGENESIS | -1.42 | 0.227 |
| GOBP_CAMP_METABOLIC_PROCESS | -1.42 | 0.227 |
| GOBP_MESONEPHROS_DEVELOPMENT | -1.42 | 0.228 |
| GOBP_POSITIVE_REGULATION_OF_POTASSIUM_ION_TRANSPORT | -1.42 | 0.228 |
| GOBP_MUSCLE_SYSTEM_PROCESS | -1.42 | 0.228 |
| GOBP_OLEFINIC_COMPOUND_BIOSYNTHETIC_PROCESS | -1.42 | 0.228 |
| GOMF_CELL_CELL_ADHESION_MEDIATOR_ACTIVITY | -1.42 | 0.228 |
| GOBP_MEMBRANE_REPOLARIZATION | -1.42 | 0.228 |
| GOCC_APICAL_PART_OF_CELL | -1.42 | 0.228 |
| GOBP_PROTEIN_LIPID_COMPLEX_ORGANIZATION | -1.41 | 0.228 |
| GOMF_PHOSPHORUS_OXYGEN_LYASE_ACTIVITY | -1.41 | 0.229 |
| GOBP_CATECHOLAMINE_TRANSPORT | -1.41 | 0.229 |
| GOBP_MORPHOGENESIS_OF_EMBRYONIC_EPITHELIUM | -1.41 | 0.230 |
| GOBP_AUTONOMIC_NERVOUS_SYSTEM_DEVELOPMENT | -1.41 | 0.230 |
| GOMF_PASSIVE_TRANSMEMBRANE_TRANSPORTER_ACTIVITY | -1.41 | 0.231 |
| GOCC_EXTRINSIC_COMPONENT_OF_PLASMA_MEMBRANE | -1.41 | 0.232 |
| GOBP_PYRIMIDINE_NUCLEOBASE_METABOLIC_PROCESS | -1.41 | 0.232 |
| GOBP_REGULATION_OF_CARDIAC_MUSCLE_CELL_ACTION_POTENTIAL | -1.41 | 0.232 |
| GOMF_PRIMARY_ACTIVE_TRANSMEMBRANE_TRANSPORTER_ACTIVITY | -1.41 | 0.232 |
| GOMF_TRANSMEMBRANE_RECEPTOR_PROTEIN_TYROSINE_KINASE_ACTIVITY | -1.41 | 0.232 |
| GOBP_PROTEIN_LOCALIZATION_TO_SITE_OF_DOUBLE_STRAND_BREAK | -1.41 | 0.232 |
| GOBP_MONOATOMIC_ION_HOMEOSTASIS | -1.41 | 0.232 |
| GOMF_TUMOR_NECROSIS_FACTOR_RECEPTOR_BINDING | -1.41 | 0.232 |
| GOBP_LONG_CHAIN_FATTY_ACYL_COA_BIOSYNTHETIC_PROCESS | -1.41 | 0.232 |
| GOBP_EMBRYONIC_PATTERN_SPECIFICATION | -1.41 | 0.234 |
| GOBP_MESODERMAL_CELL_DIFFERENTIATION | -1.41 | 0.234 |
| GOBP_CALCIUM_ION_REGULATED_EXOCYTOSIS | -1.41 | 0.234 |
| GOBP_NEUROMUSCULAR_SYNAPTIC_TRANSMISSION | -1.41 | 0.235 |
| GOBP_NEGATIVE_REGULATION_OF_STRIATED_MUSCLE_CELL_DIFFERENTIATION | -1.41 | 0.235 |
| GOBP_MUSCLE_ORGAN_DEVELOPMENT | -1.41 | 0.235 |
| GOMF_PHOSPHATIDYLSERINE_BINDING | -1.41 | 0.235 |
| GOBP_CAMERA_TYPE_EYE_MORPHOGENESIS | -1.41 | 0.235 |
| GOBP_REGULATION_OF_RECEPTOR_RECYCLING | -1.40 | 0.237 |
| GOMF_PROTEIN_SERINE_THREONINE_PHOSPHATASE_ACTIVITY | -1.40 | 0.237 |
| GOBP_TOLL_LIKE_RECEPTOR_9_SIGNALING_PATHWAY | -1.40 | 0.238 |
| GOCC_EARLY_ENDOSOME | -1.40 | 0.238 |
| GOBP_RESPONSE_TO_NUTRIENT | -1.40 | 0.239 |
| GOCC_AXON | -1.40 | 0.239 |
| GOBP_SECONDARY_METABOLITE_BIOSYNTHETIC_PROCESS | -1.40 | 0.239 |
| GOBP_PROSTANOID_BIOSYNTHETIC_PROCESS | -1.40 | 0.240 |
| GOMF_ALCOHOL_BINDING | -1.40 | 0.240 |
| GOBP_NEGATIVE_REGULATION_OF_CELL_ADHESION | -1.40 | 0.241 |
| GOMF_AMYLOID_BETA_BINDING | -1.40 | 0.242 |
| GOBP_FORMATION_OF_PRIMARY_GERM_LAYER | -1.40 | 0.242 |
| GOBP_RESPONSE_TO_CORTICOSTEROID | -1.40 | 0.242 |
| GOBP_HYALURONAN_METABOLIC_PROCESS | -1.40 | 0.242 |
| GOBP_RENAL_TUBULAR_SECRETION | -1.40 | 0.243 |
| GOBP_RESPONSE_TO_MECHANICAL_STIMULUS | -1.40 | 0.243 |
| GOBP_REGULATION_OF_CLATHRIN_DEPENDENT_ENDOCYTOSIS | -1.40 | 0.243 |
| GOBP_ORGANIC_ACID_TRANSPORT | -1.40 | 0.244 |
| GOCC_DENDRITIC_SHAFT | -1.40 | 0.244 |
| GOMF_G_PROTEIN_ALPHA_SUBUNIT_BINDING | -1.40 | 0.245 |
| GOBP_REGULATION_OF_COLLATERAL_SPROUTING | -1.40 | 0.245 |
| GOBP_UNSATURATED_FATTY_ACID_METABOLIC_PROCESS | -1.39 | 0.247 |
| GOBP_FATTY_ACID_METABOLIC_PROCESS | -1.39 | 0.247 |
| GOBP_TISSUE_REMODELING | -1.39 | 0.248 |
| GOBP_POSITIVE_REGULATION_OF_VIRAL_LIFE_CYCLE | -1.39 | 0.247 |
| GOBP_CENTRIOLE_CENTRIOLE_COHESION | -1.39 | 0.247 |
| GOBP_ICOSANOID_BIOSYNTHETIC_PROCESS | -1.39 | 0.249 |
| GOMF_GATED_CHANNEL_ACTIVITY | -1.39 | 0.250 |
| GOBP_AMIDE_TRANSPORT | -1.39 | 0.250 |
| GOMF_GROWTH_FACTOR_BINDING | -1.39 | 0.249 |
| GOMF_G_PROTEIN_COUPLED_RECEPTOR_ACTIVITY | -1.39 | 0.249 |
| GOBP_POSITIVE_REGULATION_OF_GLYCOPROTEIN_METABOLIC_PROCESS | -1.39 | 0.249 |
| GOBP_REGULATION_OF_MUSCLE_CONTRACTION | -1.39 | 0.249 |
| GOBP_REGULATION_OF_DENDRITIC_SPINE_DEVELOPMENT | -1.39 | 0.250 |
| GOBP_CELLULAR_RESPONSE_TO_AMYLOID_BETA | -1.39 | 0.250 |
| GOMF_FATTY_ACID_DERIVATIVE_BINDING | -1.39 | 0.250 |
| GOBP_DENDRITIC_SPINE_DEVELOPMENT | -1.39 | 0.250 |
| GOBP_PROSTATE_GLAND_DEVELOPMENT | -1.39 | 0.250 |

**NES: Normalized Enrichment Score, the enrichment score for the gene set after it has been normalized across analyzed gene sets. FDR q-value: False discovery rate, the estimated probability that the normalized enrichment score represents a false positive finding. Only gene sets with an FDR q-value ≤ 25% were included.**
