# Supplementary material for: Placental transcriptome profiling in congenital Chagas disease: gene networks associated with transmission
Source: Front Cell Infect Microbiol. 2026 Mar 18;16:1749307. doi: 10.3389/fcimb.2026.1749307 (PMC13038943; doi:10.3389/fcimb.2026.1749307)
Supplement: Supplementary file 11 [file Table7.docx]

**Supplementary Table 7. GSEA results using Cell Type Signature Gene Sets library for M+B+ vs M+ B- clinical groups.**

| Enriched gene sets in M+B+ | NES | FDR q-val |
| --- | --- | --- |
| DESCARTES_FETAL_PLACENTA_EXTRAVILLOUS_TROPHOBLASTS | 2.65 | 0.000 |
| DESCARTES_MAIN_FETAL_EXTRAVILLOUS_TROPHOBLASTS | 2.23 | 0.000 |
| HE_LIM_SUN_FETAL_LUNG_C3_DEFINITIVE_ERYTHROCYTE | 2.18 | 0.000 |
| DESCARTES_FETAL_ADRENAL_ERYTHROBLASTS | 2.08 | 0.001 |
| DESCARTES_FETAL_HEART_LYMPHATIC_ENDOTHELIAL_CELLS | 2.06 | 0.001 |
| DESCARTES_FETAL_KIDNEY_ERYTHROBLASTS | 1.93 | 0.005 |
| TRAVAGLINI_LUNG_SEROUS_CELL | 1.87 | 0.010 |
| HE_LIM_SUN_FETAL_LUNG_C3_PRIMITIVE_ERYTHROCYTE | 1.87 | 0.009 |
| DURANTE_ADULT_OLFACTORY_NEUROEPITHELIUM_SUSTENTACULAR_CELLS | 1.85 | 0.010 |
| DESCARTES_FETAL_INTESTINE_ERYTHROBLASTS | 1.82 | 0.012 |
| DESCARTES_FETAL_HEART_MEGAKARYOCYTES | 1.78 | 0.018 |
| GAO_LARGE_INTESTINE_24W_C8_GOBLET_CELL | 1.77 | 0.018 |
| DESCARTES_FETAL_STOMACH_ERYTHROBLASTS | 1.76 | 0.018 |
| DESCARTES_FETAL_SPLEEN_ERYTHROBLASTS | 1.76 | 0.018 |
| DESCARTES_FETAL_STOMACH_SQUAMOUS_EPITHELIAL_CELLS | 1.76 | 0.017 |
| DESCARTES_FETAL_MUSCLE_ERYTHROBLASTS | 1.75 | 0.017 |
| DESCARTES_FETAL_PANCREAS_ERYTHROBLASTS | 1.75 | 0.017 |
| HE_LIM_SUN_FETAL_LUNG_C3_DEFINITIVE_RETICULOCYTE | 1.71 | 0.023 |
| BUSSLINGER_DUODENAL_MATURE_ENTEROCYTES | 1.64 | 0.043 |
| DESCARTES_FETAL_LUNG_MEGAKARYOCYTES | 1.61 | 0.053 |
| DESCARTES_FETAL_CEREBRUM_ASTROCYTES | 1.58 | 0.067 |
| BUSSLINGER_GASTRIC_REG3A_POSITIVE_CELLS | 1.58 | 0.066 |
| DESCARTES_FETAL_ADRENAL_LYMPHOID_CELLS | 1.58 | 0.064 |
| ZHENG_CORD_BLOOD_C5_SIMILAR_TO_HSC_C6_PUTATIVE_ALTERED_METABOLIC_STATE | 1.57 | 0.066 |
| DESCARTES_FETAL_PANCREAS_LYMPHOID_CELLS | 1.56 | 0.067 |
| DESCARTES_FETAL_MUSCLE_SKELETAL_MUSCLE_CELLS | 1.56 | 0.067 |
| DESCARTES_FETAL_KIDNEY_URETERIC_BUD_CELLS | 1.56 | 0.065 |
| DESCARTES_FETAL_SPLEEN_MEGAKARYOCYTES | 1.50 | 0.104 |
| DESCARTES_MAIN_FETAL_ERYTHROBLASTS | 1.50 | 0.101 |
| DESCARTES_MAIN_FETAL_OLIGODENDROCYTES | 1.46 | 0.126 |
| DESCARTES_FETAL_LUNG_CILIATED_EPITHELIAL_CELLS | 1.46 | 0.123 |
| AIZARANI_LIVER_C8_RESIDENT_B_CELLS_1 | 1.46 | 0.119 |
| DESCARTES_FETAL_PLACENTA_AFP_ALB_POSITIVE_CELLS | 1.45 | 0.124 |
| HE_LIM_SUN_FETAL_LUNG_C3_HMOX1_POS_PRIMITIVE_ERYTHROBLAST | 1.44 | 0.131 |
| DESCARTES_FETAL_EYE_SKELETAL_MUSCLE_CELLS | 1.44 | 0.127 |
| HU_FETAL_RETINA_PHOTORECEPTOR | 1.44 | 0.127 |
| DESCARTES_FETAL_PANCREAS_LYMPHATIC_ENDOTHELIAL_CELLS | 1.44 | 0.125 |
| AIZARANI_LIVER_C22_RESIDENT_B_CELLS_2 | 1.43 | 0.135 |
| DESCARTES_FETAL_PLACENTA_PAEP_MECOM_POSITIVE_CELLS | 1.42 | 0.135 |
| DESCARTES_FETAL_THYMUS_THYMOCYTES | 1.40 | 0.157 |
| DESCARTES_FETAL_PLACENTA_TROPHOBLAST_GIANT_CELLS | 1.39 | 0.161 |
| DESCARTES_MAIN_FETAL_ASTROCYTES | 1.39 | 0.160 |
| DESCARTES_FETAL_STOMACH_CILIATED_EPITHELIAL_CELLS | 1.39 | 0.160 |
| BUSSLINGER_GASTRIC_IMMATURE_PIT_CELLS | 1.37 | 0.176 |
| DESCARTES_MAIN_FETAL_SQUAMOUS_EPITHELIAL_CELLS | 1.37 | 0.177 |
| TRAVAGLINI_LUNG_NATURAL_KILLER_CELL | 1.37 | 0.175 |
| DURANTE_ADULT_OLFACTORY_NEUROEPITHELIUM_RESPIRATORY_SECRETORY_CELLS | 1.36 | 0.187 |
| FAN_EMBRYONIC_CTX_BRAIN_EFFECTOR_T_CELL | 1.34 | 0.206 |
| DESCARTES_MAIN_FETAL_INTESTINAL_EPITHELIAL_CELLS | 1.34 | 0.207 |
| DESCARTES_FETAL_LIVER_MEGAKARYOCYTES | 1.33 | 0.206 |
| HE_LIM_SUN_FETAL_LUNG_C7_PCP4_POS_NEURON_CELL | 1.32 | 0.226 |
| TRAVAGLINI_LUNG_NATURAL_KILLER_T_CELL | 1.31 | 0.229 |
| DESCARTES_FETAL_EYE_CORNEAL_AND_CONJUNCTIVAL_EPITHELIAL_CELLS | 1.31 | 0.234 |
| DURANTE_ADULT_OLFACTORY_NEUROEPITHELIUM_RESPIRATORY_CILIATED_CELLS | 1.30 | 0.244 |
| DESCARTES_FETAL_STOMACH_LYMPHATIC_ENDOTHELIAL_CELLS | 1.30 | 0.239 |
| DESCARTES_FETAL_ADRENAL_MEGAKARYOCYTES | 1.30 | 0.244 |
| DESCARTES_FETAL_KIDNEY_MEGAKARYOCYTES | 1.29 | 0.244 |
| BUSSLINGER_GASTRIC_MATURE_PIT_CELLS | 1.29 | 0.241 |
| DESCARTES_FETAL_INTESTINE_CHROMAFFIN_CELLS | 1.28 | 0.249 |

| Enriched gene sets in M+B- | NES | FDR q-val |
| --- | --- | --- |
| DESCARTES_FETAL_PLACENTA_STROMAL_CELLS | -2.78 | 0.000 |
| CUI_DEVELOPING_HEART_C3_FIBROBLAST_LIKE_CELL | -2.52 | 0.000 |
| DESCARTES_FETAL_ADRENAL_STROMAL_CELLS | -2.37 | 0.000 |
| DESCARTES_FETAL_THYMUS_STROMAL_CELLS | -2.35 | 0.000 |
| DESCARTES_FETAL_HEART_STROMAL_CELLS | -2.34 | 0.000 |
| TRAVAGLINI_LUNG_ADVENTITIAL_FIBROBLAST_CELL | -2.34 | 0.000 |
| TRAVAGLINI_LUNG_ALVEOLAR_FIBROBLAST_CELL | -2.32 | 0.000 |
| DESCARTES_MAIN_FETAL_MYELOID_CELLS | -2.21 | 0.000 |
| DESCARTES_FETAL_LUNG_STROMAL_CELLS | -2.20 | 0.000 |
| DESCARTES_FETAL_PLACENTA_SYNCYTIOTROPHOBLASTS_AND_VILLOUS_CYTOTROPHOBLASTS | -2.19 | 0.000 |
| GAO_LARGE_INTESTINE_ADULT_CJ_IMMUNE_CELLS | -2.18 | 0.000 |
| JONES_OVARY_STROMAL | -2.18 | 0.000 |
| DESCARTES_FETAL_EYE_MICROGLIA | -2.18 | 0.000 |
| DESCARTES_FETAL_KIDNEY_STROMAL_CELLS | -2.18 | 0.000 |
| DESCARTES_FETAL_EYE_STROMAL_CELLS | -2.17 | 0.000 |
| DURANTE_ADULT_OLFACTORY_NEUROEPITHELIUM_FIBROBLASTS_STROMAL_CELLS | -2.16 | 0.000 |
| GAO_LARGE_INTESTINE_24W_C11_PANETH_LIKE_CELL | -2.16 | 0.000 |
| HE_LIM_SUN_FETAL_LUNG_C2_SPP1_POS_MACROPHAGE_CELL | -2.16 | 0.000 |
| DESCARTES_FETAL_STOMACH_STROMAL_CELLS | -2.14 | 0.000 |
| GAO_LARGE_INTESTINE_24W_C1_DCLK1POS_PROGENITOR | -2.13 | 0.000 |
| ZHONG_PFC_MAJOR_TYPES_MICROGLIA | -2.12 | 0.000 |
| TRAVAGLINI_LUNG_IGSF21_DENDRITIC_CELL | -2.11 | 0.000 |
| DESCARTES_FETAL_ADRENAL_CHROMAFFIN_CELLS | -2.10 | 0.000 |
| RUBENSTEIN_SKELETAL_MUSCLE_FAP_CELLS | -2.07 | 0.000 |
| FAN_EMBRYONIC_CTX_BIG_GROUPS_MICROGLIA | -2.07 | 0.000 |
| DESCARTES_FETAL_ADRENAL_MYELOID_CELLS | -2.06 | 0.000 |
| AIZARANI_LIVER_C6_KUPFFER_CELLS_2 | -2.04 | 0.000 |
| DESCARTES_FETAL_LIVER_STELLATE_CELLS | -2.02 | 0.000 |
| HE_LIM_SUN_FETAL_LUNG_C3_VENOUS_ENDOTHELIAL_CELL | -2.01 | 0.000 |
| HU_FETAL_RETINA_FIBROBLAST | -2.01 | 0.000 |
| DESCARTES_FETAL_PLACENTA_MYELOID_CELLS | -2.00 | 0.000 |
| HE_LIM_SUN_FETAL_LUNG_C0_MESENCHYMAL_3_CELL | -2.00 | 0.000 |
| HE_LIM_SUN_FETAL_LUNG_C3_OMD_POS_ENDOTHELIAL_CELL | -1.98 | 0.001 |
| DESCARTES_FETAL_CEREBRUM_MICROGLIA | -1.97 | 0.001 |
| DESCARTES_FETAL_PANCREAS_CCL19_CCL21_POSITIVE_CELLS | -1.97 | 0.001 |
| CUI_DEVELOPING_HEART_COMPACT_VENTRICULAR_CARDIOMYOCYTE | -1.97 | 0.001 |
| DESCARTES_FETAL_MUSCLE_MYELOID_CELLS | -1.96 | 0.001 |
| DESCARTES_FETAL_LUNG_MYELOID_CELLS | -1.95 | 0.001 |
| HE_LIM_SUN_FETAL_LUNG_C0_MID_AIRWAY_SMC_1_CELL | -1.95 | 0.001 |
| CUI_DEVELOPING_HEART_C8_MACROPHAGE | -1.94 | 0.001 |
| LAKE_ADULT_KIDNEY_C13_THICK_ASCENDING_LIMB | -1.94 | 0.001 |
| AIZARANI_LIVER_C21_STELLATE_CELLS_1 | -1.93 | 0.001 |
| DESCARTES_FETAL_INTESTINE_MYELOID_CELLS | -1.93 | 0.001 |
| DESCARTES_FETAL_MUSCLE_STROMAL_CELLS | -1.91 | 0.001 |
| HE_LIM_SUN_FETAL_LUNG_C7_COL20A1_POS_SCHWANN_CELL | -1.91 | 0.001 |
| MANNO_MIDBRAIN_NEUROTYPES_HPROGM | -1.89 | 0.002 |
| LAKE_ADULT_KIDNEY_C4_PROXIMAL_TUBULE_EPITHELIAL_CELLS_S2 | -1.87 | 0.002 |
| HE_LIM_SUN_FETAL_LUNG_C1_EARLY_AIRWAY_PROGENITOR_CELL | -1.85 | 0.003 |
| DESCARTES_FETAL_EYE_ASTROCYTES | -1.85 | 0.003 |
| RUBENSTEIN_SKELETAL_MUSCLE_FBN1_FAP_CELLS | -1.85 | 0.003 |
| AIZARANI_LIVER_C2_KUPFFER_CELLS_1 | -1.84 | 0.003 |
| DESCARTES_MAIN_FETAL_SYNCYTIOTROPHOBLASTS_AND_VILLOUS_CYTOTROPHOBLASTS | -1.84 | 0.003 |
| LAKE_ADULT_KIDNEY_C1_EPITHELIAL_CELLS_UNASSIGNED | -1.84 | 0.003 |
| HE_LIM_SUN_FETAL_LUNG_C0_LATE_AIRWAY_SMC_CELL | -1.84 | 0.003 |
| FAN_EMBRYONIC_CTX_BRAIN_ENDOTHELIAL_2 | -1.83 | 0.004 |
| JONES_OVARY_MACROPHAGE | -1.82 | 0.004 |
| HU_FETAL_RETINA_MICROGLIA | -1.81 | 0.005 |
| HE_LIM_SUN_FETAL_LUNG_C1_PULMONARY_NEUROENDOCRINE_CELL | -1.80 | 0.005 |
| DESCARTES_FETAL_PANCREAS_MYELOID_CELLS | -1.80 | 0.005 |
| CUI_DEVELOPING_HEART_5TH_WEEK_ATRIAL_CARDIOMYOCYTE | -1.79 | 0.005 |
| HE_LIM_SUN_FETAL_LUNG_C0_RESTING_CHONDROCYTE | -1.79 | 0.006 |
| HE_LIM_SUN_FETAL_LUNG_C0_ASPN_POS_CHONDROCYTE | -1.77 | 0.007 |
| HE_LIM_SUN_FETAL_LUNG_C0_PERICYTE | -1.77 | 0.007 |
| DESCARTES_FETAL_KIDNEY_MYELOID_CELLS | -1.77 | 0.007 |
| MENON_FETAL_KIDNEY_3_STROMAL_CELLS | -1.77 | 0.007 |
| MANNO_MIDBRAIN_NEUROTYPES_HNBGABA | -1.76 | 0.008 |
| HE_LIM_SUN_FETAL_LUNG_C0_ADVENTITIAL_FIBROBLAST | -1.75 | 0.008 |
| DESCARTES_FETAL_INTESTINE_ENS_NEURONS | -1.74 | 0.009 |
| AIZARANI_LIVER_C18_NK_NKT_CELLS_5 | -1.74 | 0.009 |
| JONES_OVARY_THECA | -1.74 | 0.009 |
| GAUTAM_EYE_IRIS_CILIARY_BODY_WIF1_HIGH_FIBROBLASTS | -1.73 | 0.009 |
| TRAVAGLINI_LUNG_MYOFIBROBLAST_CELL | -1.73 | 0.009 |
| DESCARTES_FETAL_STOMACH_MYELOID_CELLS | -1.73 | 0.009 |
| DESCARTES_MAIN_FETAL_MICROGLIA | -1.73 | 0.009 |
| DESCARTES_FETAL_SPLEEN_MESOTHELIAL_CELLS | -1.73 | 0.009 |
| BUSSLINGER_DUODENAL_TUFT_CELLS | -1.73 | 0.009 |
| HE_LIM_SUN_FETAL_LUNG_C1_LATE_AIRWAY_PROGENITOR_CELL | -1.73 | 0.009 |
| CUI_DEVELOPING_HEART_LEFT_VENTRICULAR_CARDIOMYOCYTE | -1.72 | 0.010 |
| AIZARANI_LIVER_C25_KUPFFER_CELLS_4 | -1.72 | 0.010 |
| FAN_OVARY_CL5_HEALTHY_SELECTABLE_FOLLICLE_THECAL_CELL | -1.72 | 0.010 |
| AIZARANI_LIVER_C4_EPCAM_POS_BILE_DUCT_CELLS_1 | -1.71 | 0.011 |
| HE_LIM_SUN_FETAL_LUNG_C1_AT1_CELL | -1.71 | 0.011 |
| HU_FETAL_RETINA_RPE | -1.71 | 0.011 |
| LAKE_ADULT_KIDNEY_C29_UNKNOWN_NOVEL_PT_CFH_POS_SUBPOPULATION_S2 | -1.71 | 0.011 |
| HE_LIM_SUN_FETAL_LUNG_C3_LYMPHATIC_ENDOTHELIAL_CELL | -1.70 | 0.011 |
| DESCARTES_FETAL_LIVER_MYELOID_CELLS | -1.70 | 0.011 |
| GAO_LARGE_INTESTINE_ADULT_CG_GOBLET_CELL_SUBTYPE_2 | -1.69 | 0.012 |
| DESCARTES_FETAL_THYMUS_VASCULAR_ENDOTHELIAL_CELLS | -1.69 | 0.013 |
| HE_LIM_SUN_FETAL_LUNG_C3_ARTERIAL_ENDOTHELIAL_CELL | -1.69 | 0.013 |
| CUI_DEVELOPING_HEART_C5_VALVAR_CELL | -1.68 | 0.013 |
| MANNO_MIDBRAIN_NEUROTYPES_HRGL2C | -1.68 | 0.014 |
| MANNO_MIDBRAIN_NEUROTYPES_HRGL1 | -1.68 | 0.014 |
| BUSSLINGER_GASTRIC_D_CELLS | -1.67 | 0.014 |
| MANNO_MIDBRAIN_NEUROTYPES_HOMTN | -1.67 | 0.014 |
| ZHONG_PFC_C5_BCL11B_CALB2_POS_INP | -1.67 | 0.014 |
| HE_LIM_SUN_FETAL_LUNG_C7_MID_SCHWANN_CELL | -1.66 | 0.015 |
| TRAVAGLINI_LUNG_VEIN_CELL | -1.66 | 0.015 |
| LAKE_ADULT_KIDNEY_C21_COLLECTING_DUCT_INTERCALATED_CELLS_TYPE_B | -1.66 | 0.015 |
| HE_LIM_SUN_FETAL_LUNG_C1_PULMONARY_NE_PRECURSOR_CELL | -1.66 | 0.015 |
| DESCARTES_FETAL_CEREBELLUM_ASTROCYTES | -1.66 | 0.015 |
| AIZARANI_LIVER_C23_KUPFFER_CELLS_3 | -1.66 | 0.015 |
| DESCARTES_FETAL_INTESTINE_MESOTHELIAL_CELLS | -1.65 | 0.016 |
| JONES_OVARY_T_CELL | -1.65 | 0.015 |
| DESCARTES_MAIN_FETAL_BIPOLAR_CELLS | -1.65 | 0.016 |
| HE_LIM_SUN_FETAL_LUNG_C0_INTERM_CHONDROCYTE | -1.65 | 0.016 |
| LAKE_ADULT_KIDNEY_C2_PODOCYTES | -1.65 | 0.016 |
| HE_LIM_SUN_FETAL_LUNG_C0_MESENCHYMAL_1_CELL | -1.64 | 0.017 |
| GAUTAM_EYE_IRIS_CILIARY_BODY_MGP_HIGH_FIBROBLASTS | -1.64 | 0.017 |
| GAO_LARGE_INTESTINE_ADULT_CA_ENTEROENDOCRINE_CELLS | -1.64 | 0.017 |
| HE_LIM_SUN_FETAL_LUNG_C0_MYOFIBROBLAST_3_CELL | -1.64 | 0.017 |
| MANNO_MIDBRAIN_NEUROTYPES_HRGL3 | -1.64 | 0.017 |
| MANNO_MIDBRAIN_NEUROTYPES_HDA2 | -1.63 | 0.018 |
| FAN_EMBRYONIC_CTX_OPC | -1.63 | 0.019 |
| MANNO_MIDBRAIN_NEUROTYPES_HDA | -1.63 | 0.018 |
| AIZARANI_LIVER_C17_HEPATOCYTES_3 | -1.62 | 0.019 |
| DESCARTES_FETAL_CEREBELLUM_OLIGODENDROCYTES | -1.62 | 0.019 |
| LAKE_ADULT_KIDNEY_C3_PROXIMAL_TUBULE_EPITHELIAL_CELLS_S1_S2 | -1.62 | 0.020 |
| HE_LIM_SUN_FETAL_LUNG_C0_MYL4_POS_SMC_CELL | -1.61 | 0.020 |
| CUI_DEVELOPING_HEART_SMOOTH_MUSCLE_CELL | -1.61 | 0.021 |
| HE_LIM_SUN_FETAL_LUNG_C0_MYOFIBROBLAST_1_CELL | -1.60 | 0.022 |
| LAKE_ADULT_KIDNEY_C28_INTERSTITIUM | -1.60 | 0.023 |
| DESCARTES_FETAL_THYMUS_ANTIGEN_PRESENTING_CELLS | -1.60 | 0.024 |
| FAN_OVARY_CL6_PUTATIVE_EARLY_ATRETIC_FOLLICLE_THECAL_CELL_2 | -1.59 | 0.024 |
| TRAVAGLINI_LUNG_CLASSICAL_MONOCYTE_CELL | -1.59 | 0.024 |
| MANNO_MIDBRAIN_NEUROTYPES_HNBML1 | -1.59 | 0.024 |
| BUSSLINGER_GASTRIC_OXYNTIC_ENTEROCHROMAFFIN_LIKE_CELLS | -1.59 | 0.024 |
| HE_LIM_SUN_FETAL_LUNG_C0_VASCULAR_SMC_1_CELL | -1.59 | 0.024 |
| HE_LIM_SUN_FETAL_LUNG_C3_INTERMEDIATE_LYMPHATIC_ENDO_CELL | -1.59 | 0.025 |
| LAKE_ADULT_KIDNEY_C15_CONNECTING_TUBULE | -1.58 | 0.025 |
| HE_LIM_SUN_FETAL_LUNG_C3_GRIA2_POS_ARTERIAL_ENDO_CELL | -1.58 | 0.025 |
| TRAVAGLINI_LUNG_LYMPHATIC_CELL | -1.58 | 0.025 |
| MANNO_MIDBRAIN_NEUROTYPES_HRN | -1.58 | 0.025 |
| DESCARTES_FETAL_SPLEEN_MYELOID_CELLS | -1.58 | 0.026 |
| CUI_DEVELOPING_HEART_CARDIAC_FIBROBLASTS | -1.58 | 0.026 |
| HE_LIM_SUN_FETAL_LUNG_C1_INTERM_NEUROENDOCRINE_CELL | -1.56 | 0.030 |
| CUI_DEVELOPING_HEART_VASCULAR_ENDOTHELIAL_CELL | -1.56 | 0.030 |
| LAKE_ADULT_KIDNEY_C14_DISTAL_CONVOLUTED_TUBULE | -1.56 | 0.031 |
| ZHONG_PFC_C9_ORG_OTHER | -1.56 | 0.031 |
| AIZARANI_LIVER_C13_LSECS_2 | -1.55 | 0.031 |
| DESCARTES_FETAL_LUNG_MESOTHELIAL_CELLS | -1.55 | 0.032 |
| MENON_FETAL_KIDNEY_5_PROXIMAL_TUBULE_CELLS | -1.55 | 0.032 |
| HE_LIM_SUN_FETAL_LUNG_C1_SMG_BASAL_CELL | -1.55 | 0.032 |
| AIZARANI_LIVER_C29_MVECS_2 | -1.55 | 0.033 |
| CUI_DEVELOPING_HEART_C1_5TH_WEEK_CARDIAC_CELLS | -1.54 | 0.033 |
| MURARO_PANCREAS_PANCREATIC_POLYPEPTIDE_CELL | -1.54 | 0.032 |
| DESCARTES_FETAL_HEART_MYELOID_CELLS | -1.54 | 0.032 |
| ZHONG_PFC_MAJOR_TYPES_OPC | -1.54 | 0.032 |
| HE_LIM_SUN_FETAL_LUNG_C0_MID_AIRWAY_SMC_2_CELL | -1.54 | 0.032 |
| JONES_OVARY_NK_CELL | -1.54 | 0.032 |
| DESCARTES_FETAL_PLACENTA_VASCULAR_ENDOTHELIAL_CELLS | -1.54 | 0.033 |
| DESCARTES_MAIN_FETAL_PDE11A_FAM19A2_POSITIVE_CELLS | -1.54 | 0.033 |
| GAO_ESOPHAGUS_25W_C4_FGFR1HIGH_EPITHELIAL_CELLS | -1.53 | 0.035 |
| DESCARTES_FETAL_HEART_ENDOCARDIAL_CELLS | -1.53 | 0.036 |
| MANNO_MIDBRAIN_NEUROTYPES_HRGL2A | -1.53 | 0.036 |
| DURANTE_ADULT_OLFACTORY_NEUROEPITHELIUM_DENDRITIC_CELLS | -1.53 | 0.035 |
| HE_LIM_SUN_FETAL_LUNG_C0_MESENCHYMAL_2_CELL | -1.53 | 0.036 |
| DURANTE_ADULT_OLFACTORY_NEUROEPITHELIUM_OLFACTORY_MICROVILLAR_CELLS | -1.53 | 0.036 |
| HE_LIM_SUN_FETAL_LUNG_C0_VASCULAR_SMC_2_CELL | -1.52 | 0.037 |
| DESCARTES_MAIN_FETAL_SMOOTH_MUSCLE_CELLS | -1.51 | 0.039 |
| BUSSLINGER_GASTRIC_G_CELLS | -1.51 | 0.039 |
| DESCARTES_FETAL_PLACENTA_IGFBP1_DKK1_POSITIVE_CELLS | -1.51 | 0.040 |
| DESCARTES_MAIN_FETAL_CARDIOMYOCYTES | -1.51 | 0.040 |
| HE_LIM_SUN_FETAL_LUNG_C0_AIRWAY_FIBROBLAST | -1.51 | 0.041 |
| FAN_EMBRYONIC_CTX_ASTROCYTE_2 | -1.50 | 0.041 |
| CUI_DEVELOPING_HEART_C6_EPICARDIAL_CELL | -1.50 | 0.042 |
| MANNO_MIDBRAIN_NEUROTYPES_HDA1 | -1.50 | 0.042 |
| DESCARTES_MAIN_FETAL_VASCULAR_ENDOTHELIAL_CELLS | -1.50 | 0.043 |
| HE_LIM_SUN_FETAL_LUNG_C7_KCNIP4_POS_NEURON_CELL | -1.49 | 0.044 |
| MANNO_MIDBRAIN_NEUROTYPES_HRGL2B | -1.49 | 0.045 |
| HE_LIM_SUN_FETAL_LUNG_C1_MID_BASAL_CELL | -1.49 | 0.045 |
| DESCARTES_FETAL_LIVER_MESOTHELIAL_CELLS | -1.49 | 0.045 |
| DESCARTES_MAIN_FETAL_ENDOCARDIAL_CELLS | -1.48 | 0.048 |
| GAO_LARGE_INTESTINE_ADULT_CE_OLFM4HIGH_STEM_CELL | -1.48 | 0.049 |
| ZHONG_PFC_C2_SOX5_BCL11B_POS_EXCITATORY_NEURON | -1.48 | 0.049 |
| HE_LIM_SUN_FETAL_LUNG_C0_EARLY_MESOTHELIAL_CELL | -1.48 | 0.049 |
| AIZARANI_LIVER_C33_STELLATE_CELLS_2 | -1.48 | 0.049 |
| HAY_BONE_MARROW_CD34_POS_HSC | -1.47 | 0.049 |
| HE_LIM_SUN_FETAL_LUNG_C7_LATE_SCHWANN_CELL | -1.47 | 0.049 |
| DURANTE_ADULT_OLFACTORY_NEUROEPITHELIUM_MACROPHAGES | -1.47 | 0.049 |
| MANNO_MIDBRAIN_NEUROTYPES_HPROGFPL | -1.47 | 0.049 |
| TRAVAGLINI_LUNG_PERICYTE_CELL | -1.47 | 0.050 |
| DESCARTES_FETAL_INTESTINE_STROMAL_CELLS | -1.47 | 0.050 |
| MANNO_MIDBRAIN_NEUROTYPES_HOPC | -1.47 | 0.051 |
| GAO_SMALL_INTESTINE_24W_C2_PROCRPOS_PROGENITOR | -1.47 | 0.051 |
| GAO_LARGE_INTESTINE_ADULT_CI_MESENCHYMAL_CELLS | -1.46 | 0.052 |
| DESCARTES_FETAL_LIVER_VASCULAR_ENDOTHELIAL_CELLS | -1.46 | 0.052 |
| HE_LIM_SUN_FETAL_LUNG_C1_MUC5AC_POS_ASCL1_POS_PROGENITOR_CELL | -1.46 | 0.052 |
| AIZARANI_LIVER_C34_MHC_II_POS_B_CELLS | -1.46 | 0.053 |
| DESCARTES_FETAL_EYE_RETINAL_PIGMENT_CELLS | -1.46 | 0.053 |
| DESCARTES_FETAL_STOMACH_ENS_NEURONS | -1.45 | 0.055 |
| DURANTE_ADULT_OLFACTORY_NEUROEPITHELIUM_OLFACTORY_ENSHEATHING_GLIA | -1.45 | 0.057 |
| DESCARTES_FETAL_MUSCLE_SATELLITE_CELLS | -1.45 | 0.057 |
| CUI_DEVELOPING_HEART_VALVAR_ENDOTHELIAL_CELL | -1.45 | 0.057 |
| MURARO_PANCREAS_DELTA_CELL | -1.45 | 0.057 |
| DESCARTES_FETAL_SPLEEN_AFP_ALB_POSITIVE_CELLS | -1.44 | 0.058 |
| CUI_DEVELOPING_HEART_ENDOCARDIAL_CELL | -1.44 | 0.058 |
| HE_LIM_SUN_FETAL_LUNG_C2_DC1_CELL | -1.43 | 0.064 |
| DESCARTES_FETAL_HEART_SATB2_LRRC7_POSITIVE_CELLS | -1.43 | 0.064 |
| AIZARANI_LIVER_C14_HEPATOCYTES_2 | -1.43 | 0.064 |
| MANNO_MIDBRAIN_NEUROTYPES_HNBM | -1.43 | 0.064 |
| TRAVAGLINI_LUNG_LIPOFIBROBLAST_CELL | -1.42 | 0.067 |
| DESCARTES_FETAL_KIDNEY_METANEPHRIC_CELLS | -1.42 | 0.068 |
| DURANTE_ADULT_OLFACTORY_NEUROEPITHELIUM_CD4_T_CELLS | -1.42 | 0.068 |
| DESCARTES_FETAL_PLACENTA_SMOOTH_MUSCLE_CELLS | -1.42 | 0.068 |
| TRAVAGLINI_LUNG_PLASMACYTOID_DENDRITIC_CELL | -1.42 | 0.068 |
| AIZARANI_LIVER_C11_HEPATOCYTES_1 | -1.42 | 0.069 |
| MURARO_PANCREAS_ENDOTHELIAL_CELL | -1.42 | 0.069 |
| FAN_EMBRYONIC_CTX_MICROGLIA_3 | -1.41 | 0.070 |
| HAY_BONE_MARROW_NEUTROPHIL | -1.41 | 0.070 |
| TRAVAGLINI_LUNG_BASAL_CELL | -1.41 | 0.071 |
| TRAVAGLINI_LUNG_B_CELL | -1.41 | 0.071 |
| DESCARTES_FETAL_MUSCLE_VASCULAR_ENDOTHELIAL_CELLS | -1.41 | 0.071 |
| LAKE_ADULT_KIDNEY_C16_COLLECTING_SYSTEM_PRINCIPAL_CELLS_CORTEX | -1.41 | 0.071 |
| BUSSLINGER_GASTRIC_X_CELLS | -1.40 | 0.074 |
| LAKE_ADULT_KIDNEY_C20_COLLECTING_DUCT_INTERCALATED_CELLS_TYPE_A_CORTEX | -1.40 | 0.074 |
| DESCARTES_MAIN_FETAL_SCHWANN_CELLS | -1.40 | 0.074 |
| DESCARTES_MAIN_FETAL_MESOTHELIAL_CELLS | -1.40 | 0.074 |
| DESCARTES_FETAL_PANCREAS_ENS_NEURONS | -1.40 | 0.074 |
| HE_LIM_SUN_FETAL_LUNG_C7_TM4SF4_POS_PENK_POS_NEURON_CELL | -1.40 | 0.076 |
| ZHONG_PFC_C3_MICROGLIA | -1.39 | 0.080 |
| DURANTE_ADULT_OLFACTORY_NEUROEPITHELIUM_MAST_CELLS | -1.39 | 0.080 |
| MURARO_PANCREAS_EPSILON_CELL | -1.39 | 0.082 |
| HAY_BONE_MARROW_EARLY_ERYTHROBLAST | -1.38 | 0.087 |
| FAN_EMBRYONIC_CTX_BIG_GROUPS_EXCITATORY_NEURON | -1.38 | 0.087 |
| GAO_SMALL_INTESTINE_24W_C3_ENTEROCYTE_PROGENITOR_SUBTYPE_1 | -1.38 | 0.086 |
| HE_LIM_SUN_FETAL_LUNG_C1_LATE_BASAL_CELL | -1.38 | 0.086 |
| HE_LIM_SUN_FETAL_LUNG_C0_MYOFIBROBLAST_2_CELL | -1.38 | 0.086 |
| VANGURP_PANCREATIC_GAMMA_CELL | -1.38 | 0.086 |
| HU_FETAL_RETINA_AMACRINE | -1.37 | 0.088 |
| HE_LIM_SUN_FETAL_LUNG_C2_APOE_POS_M2_MACROPHAGE_CELL | -1.37 | 0.088 |
| HE_LIM_SUN_FETAL_LUNG_C1_PROXIMAL_SECRETORY_2_CELL | -1.37 | 0.090 |
| DESCARTES_MAIN_FETAL_LYMPHOID_CELLS | -1.37 | 0.091 |
| DESCARTES_MAIN_FETAL_SATELLITE_CELLS | -1.37 | 0.090 |
| HE_LIM_SUN_FETAL_LUNG_C4_ILCP_CELL | -1.37 | 0.090 |
| BUSSLINGER_ESOPHAGEAL_DENDRITIC_CELLS | -1.36 | 0.093 |
| AIZARANI_LIVER_C7_EPCAM_POS_BILE_DUCT_CELLS_2 | -1.36 | 0.093 |
| HE_LIM_SUN_FETAL_LUNG_C1_PROXIMAL_BASAL_CELL | -1.36 | 0.095 |
| DESCARTES_FETAL_LUNG_VISCERAL_NEURONS | -1.36 | 0.096 |
| TRAVAGLINI_LUNG_MACROPHAGE_CELL | -1.36 | 0.096 |
| JONES_OVARY_PERICYTE | -1.35 | 0.096 |
| LAKE_ADULT_KIDNEY_C11_THIN_ASCENDING_LIMB | -1.35 | 0.096 |
| HE_LIM_SUN_FETAL_LUNG_C1_CLUB_CELL | -1.35 | 0.097 |
| DESCARTES_MAIN_FETAL_CCL19_CCL21_POSITIVE_CELLS | -1.35 | 0.097 |
| DESCARTES_MAIN_FETAL_IGFBP1_DKK1_POSITIVE_CELLS | -1.35 | 0.100 |
| HU_FETAL_RETINA_RPC | -1.35 | 0.101 |
| GAUTAM_EYE_CHOROID_SCLERA_FIBROBLASTS | -1.35 | 0.101 |
| MANNO_MIDBRAIN_NEUROTYPES_HNBML5 | -1.34 | 0.101 |
| MANNO_MIDBRAIN_NEUROTYPES_HSERT | -1.34 | 0.102 |
| ZHONG_PFC_C7_ORG_UNDERGOING_NEURONAL_DIFFERENTIATION | -1.34 | 0.102 |
| HE_LIM_SUN_FETAL_LUNG_C6_MUC16_POS_CILIATED_CELL | -1.34 | 0.103 |
| FAN_OVARY_CL1_GPRC5A_TNFRS12A_HIGH_SELECTABLE_FOLLICLE_STROMAL_CELL | -1.34 | 0.103 |
| DESCARTES_FETAL_THYMUS_THYMIC_EPITHELIAL_CELLS | -1.34 | 0.106 |
| FAN_EMBRYONIC_CTX_NSC_1 | -1.34 | 0.105 |
| HE_LIM_SUN_FETAL_LUNG_C1_GHRL_POS_NE_PRECURSOR_CELL | -1.33 | 0.106 |
| DESCARTES_FETAL_KIDNEY_MESANGIAL_CELLS | -1.33 | 0.110 |
| VANGURP_PANCREATIC_ALPHA_CELL | -1.33 | 0.110 |
| DESCARTES_FETAL_CEREBRUM_VASCULAR_ENDOTHELIAL_CELLS | -1.33 | 0.111 |
| DESCARTES_FETAL_KIDNEY_VASCULAR_ENDOTHELIAL_CELLS | -1.33 | 0.112 |
| TRAVAGLINI_LUNG_BASOPHIL_MAST_1_CELL | -1.32 | 0.112 |
| AIZARANI_LIVER_C32_MVECS_3 | -1.32 | 0.113 |
| DESCARTES_FETAL_CEREBRUM_OLIGODENDROCYTES | -1.32 | 0.113 |
| DESCARTES_FETAL_LIVER_HEPATOBLASTS | -1.32 | 0.114 |
| ZHENG_CORD_BLOOD_C4_PUTATIVE_EARLY_ERYTHROID_COMMITMENT | -1.31 | 0.120 |
| CUI_DEVELOPING_HEART_C7_MAST_CELL | -1.31 | 0.124 |
| HAY_BONE_MARROW_FOLLICULAR_B_CELL | -1.31 | 0.125 |
| MURARO_PANCREAS_ALPHA_CELL | -1.31 | 0.125 |
| DESCARTES_FETAL_SPLEEN_STROMAL_CELLS | -1.30 | 0.126 |
| VANGURP_PANCREATIC_BETA_CELL | -1.30 | 0.129 |
| GAO_LARGE_INTESTINE_24W_C6_SECRETORY_PROGENITOR | -1.30 | 0.131 |
| TRAVAGLINI_LUNG_NONCLASSICAL_MONOCYTE_CELL | -1.29 | 0.136 |
| GAUTAM_EYE_IRIS_CILIARY_BODY_CRYAA_HIGH_CILIARY_BODY_CELLS | -1.29 | 0.135 |
| LAKE_ADULT_KIDNEY_C5_PROXIMAL_TUBULE_EPITHELIAL_CELLS_STRESS_INFLAM | -1.29 | 0.136 |
| DESCARTES_FETAL_INTESTINE_INTESTINAL_EPITHELIAL_CELLS | -1.29 | 0.138 |
| DESCARTES_FETAL_ADRENAL_VASCULAR_ENDOTHELIAL_CELLS | -1.28 | 0.141 |
| HE_LIM_SUN_FETAL_LUNG_C5_CD5_POS_CCL22_NEG_MATURE_B_CELL | -1.28 | 0.141 |
| AIZARANI_LIVER_C12_NK_NKT_CELLS_4 | -1.28 | 0.144 |
| CUI_DEVELOPING_HEART_TRABECULAR_VENTRICULAR_CARDIOMYOCYTE | -1.28 | 0.144 |
| DURANTE_ADULT_OLFACTORY_NEUROEPITHELIUM_IMMATURE_NEURONS | -1.27 | 0.152 |
| HAY_BONE_MARROW_MONOCYTE | -1.27 | 0.152 |
| HAY_BONE_MARROW_CD34_POS_MKP | -1.27 | 0.152 |
| TRAVAGLINI_LUNG_ARTERY_CELL | -1.27 | 0.155 |
| AIZARANI_LIVER_C9_LSECS_1 | -1.27 | 0.156 |
| LAKE_ADULT_KIDNEY_C25_ENDOTHELIAL_CELLS_UNASSIGNED | -1.26 | 0.158 |
| DESCARTES_MAIN_FETAL_DUCTAL_CELLS | -1.26 | 0.161 |
| LAKE_ADULT_KIDNEY_C7_PROXIMAL_TUBULE_EPITHELIAL_CELLS_S3 | -1.26 | 0.163 |
| AIZARANI_LIVER_C10_MVECS_1 | -1.26 | 0.165 |
| TRAVAGLINI_LUNG_SIGNALING_ALVEOLAR_EPITHELIAL_TYPE_2_CELL | -1.25 | 0.167 |
| DESCARTES_FETAL_PANCREAS_DUCTAL_CELLS | -1.25 | 0.167 |
| ZHONG_PFC_MAJOR_TYPES_ASTROCYTES | -1.25 | 0.167 |
| HE_LIM_SUN_FETAL_LUNG_C2_S100A12_HI_CLASSICAL_MONOCYTE | -1.25 | 0.167 |
| HE_LIM_SUN_FETAL_LUNG_C1_GHRL_POS_NEUROENDOCRINE_CELL | -1.25 | 0.167 |
| MANNO_MIDBRAIN_NEUROTYPES_HPROGBP | -1.25 | 0.169 |
| FAN_EMBRYONIC_CTX_BRAIN_ENDOTHELIAL_1 | -1.25 | 0.172 |
| MENON_FETAL_KIDNEY_2_NEPHRON_PROGENITOR_CELLS | -1.25 | 0.173 |
| DESCARTES_FETAL_STOMACH_MESOTHELIAL_CELLS | -1.24 | 0.175 |
| AIZARANI_LIVER_C5_NK_NKT_CELLS_3 | -1.24 | 0.179 |
| MANNO_MIDBRAIN_NEUROTYPES_HNPROG | -1.24 | 0.181 |
| CUI_DEVELOPING_HEART_RIGHT_VENTRICULAR_CARDIOMYOCYTE | -1.24 | 0.185 |
| DESCARTES_FETAL_INTESTINE_VASCULAR_ENDOTHELIAL_CELLS | -1.23 | 0.185 |
| FAN_EMBRYONIC_CTX_BIG_GROUPS_BRAIN_ENDOTHELIAL | -1.23 | 0.185 |
| TRAVAGLINI_LUNG_ALVEOLAR_EPITHELIAL_TYPE_1_CELL | -1.23 | 0.185 |
| DURANTE_ADULT_OLFACTORY_NEUROEPITHELIUM_CD8_T_CELLS | -1.23 | 0.185 |
| DESCARTES_MAIN_FETAL_ADRENOCORTICAL_CELLS | -1.23 | 0.185 |
| GAUTAM_EYE_IRIS_CILIARY_BODY_FIBROBLASTS | -1.23 | 0.185 |
| FAN_OVARY_CL2_PUTATIVE_EARLY_ATRETIC_FOLLICLE_THECAL_CELL_1 | -1.23 | 0.187 |
| DURANTE_ADULT_OLFACTORY_NEUROEPITHELIUM_B_CELLS | -1.23 | 0.187 |
| HE_LIM_SUN_FETAL_LUNG_C3_AEROCYTE | -1.23 | 0.188 |
| CUI_DEVELOPING_HEART_C4_ENDOTHELIAL_CELL | -1.23 | 0.188 |
| FAN_OVARY_CL11_MURAL_GRANULOSA_CELL | -1.23 | 0.190 |
| BUSSLINGER_GASTRIC_PARIETAL_CELLS | -1.22 | 0.194 |
| HE_LIM_SUN_FETAL_LUNG_C5_CD5_NEG_MATURE_B_CELL | -1.22 | 0.199 |
| JONES_OVARY_GRANULOSA | -1.21 | 0.204 |
| DESCARTES_FETAL_LUNG_BRONCHIOLAR_AND_ALVEOLAR_EPITHELIAL_CELLS | -1.21 | 0.204 |
| DESCARTES_MAIN_FETAL_STELLATE_CELLS | -1.21 | 0.211 |
| GAUTAM_EYE_IRIS_CILIARY_BODY_CILIARY_BODY_CELLS | -1.21 | 0.212 |
| HE_LIM_SUN_FETAL_LUNG_C1_EARLY_TIP_CELL | -1.21 | 0.212 |
| DESCARTES_FETAL_HEART_VASCULAR_ENDOTHELIAL_CELLS | -1.20 | 0.218 |
| HU_FETAL_RETINA_RGC | -1.20 | 0.219 |
| FAN_EMBRYONIC_CTX_BRAIN_MYELOID | -1.20 | 0.221 |
| MANNO_MIDBRAIN_NEUROTYPES_HPROGFPM | -1.20 | 0.221 |
| DESCARTES_FETAL_MUSCLE_LYMPHATIC_ENDOTHELIAL_CELLS | -1.20 | 0.222 |
| HE_LIM_SUN_FETAL_LUNG_C7_SCHWANN_PRECURSOR_CELL | -1.19 | 0.228 |
| DESCARTES_FETAL_CEREBRUM_INHIBITORY_NEURONS | -1.19 | 0.230 |
| DESCARTES_FETAL_HEART_SMOOTH_MUSCLE_CELLS | -1.19 | 0.233 |
| DESCARTES_FETAL_HEART_VISCERAL_NEURONS | -1.18 | 0.241 |
| DESCARTES_MAIN_FETAL_RETINAL_PIGMENT_CELLS | -1.18 | 0.242 |
| DESCARTES_FETAL_PANCREAS_MESOTHELIAL_CELLS | -1.18 | 0.247 |

**NES: Normalized Enrichment Score, the enrichment score for the gene set after it has been normalized across analyzed gene sets. FDR q-value: False discovery rate, the estimated probability that the normalized enrichment score represents a false positive finding. Only gene sets with an FDR q-value ≤ 25% were included.**
